# Multitargeted Caffeic Acid Derivatives Inhibit Cardiac RyR2- and Na_V_1.5- Channels but Stimulate SERCA2a Pump Activity

**DOI:** 10.1021/acsomega.5c04399

**Published:** 2025-09-17

**Authors:** Gyuzel Y. Mitronova, Christine Quentin, Vladimir N. Belov, Kamila A. Kiszka, Jörg W. Wegener, Stephan E. Lehnart

**Affiliations:** † Department of NanoBiophotonics, 28282 Max Planck Institute for Multidisciplinary Sciences, Am Fassberg 11, 37077 Göttingen, Germany; ‡ Department of Cardiology & Pulmonology, Heart Research Center Göttingen, 27177 University Medical Center Göttingen, Robert-Koch-Strasse 42a, 37075 Göttingen, Germany; § DZHK (German Centre for Cardiovascular Research), Partner Site Lower Saxony, 37075 Göttingen, Germany; ∥ Department of Translational Pharmacology, Medical School OWL, Bielefeld University, Universitätsstraße 25, 33615 Bielefeld, Germany

## Abstract

Heart beat relies on coordinated calcium regulation through RyR2 and SERCA2a activity, as well as impulse propagation mediated by Na_V_1.5 activity. Here, we present small molecules combining a 1,4-benzothia- or 1,4-benzoxazepine scaffold and a 3-(3,4-dihydroxyphenyl)-2-propenoic acid residue of which five compounds (**4**, **9**, **10**, **12**, and **13**) reduced cardiac RyR2 activity and stimulated SERCA2a activity in our cell systems. The acceleration of SERCA2 activity was particularly enhanced by cyanoborane derivatives **11**–**13**. The substantial impact on SERCA2 activation relates to the presence of polar (zwitterionic) N–B fragments, which can increase the binding affinity to SERCA2 and result in a more efficient activation. Compounds **12** and **13** at a concentration of 10 μM additionally reduced Na_V_1.5 activity in Chinese hamster ovary (CHO) cells, indicating a multitargeted drug property. We propose that the multitargeted actions of these novel compounds have the potential to enhance heart failure therapy, particularly in personalized treatment approaches.

## Graphical abstract

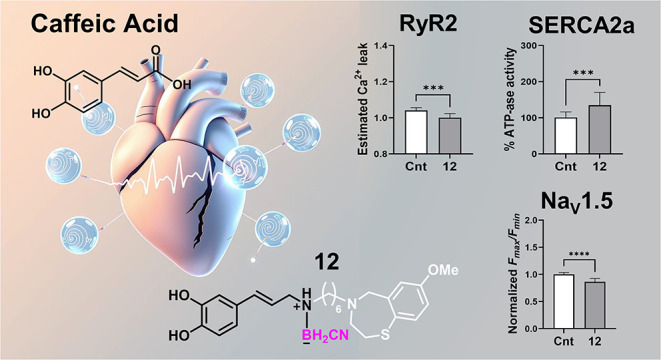

## Introduction

Heart beat depends on coordinated contraction and relaxation of cardiac muscles. Ryanodine receptor 2 (RyR2) and sarco/endoplasmic reticulum Ca^2+^-dependent ATPase 2a (SERCA2a) are abundant proteins essential for this process in cardiac myocytes.^1^ They work together to regulate Ca^2+^ cycling in cardiac myocytes. RyR2 mediates the rapid release of Ca^2+^ from the specialized sarcoendoplasmic reticulum (SER) into the cytoplasm. On the other hand, SERCA2a, transports Ca^2+^ from the cytoplasm back into SER. These cycles of SER Ca^2+^ release and reuptake enable repeated muscle contraction and relaxation. Alterations in the functions of RyR2 or SERCA2a can lead to impaired heart function and various pathological conditions including heart failure (HF) and cardiac arrhythmias.^2–6^ In HF, for instance, RyR2 channels are hyperphosphorylated by protein kinase A (PKA) and Ca^2+^-calmodulin-dependent kinase II (CaMKII), impairing binding of the stabilizing subunit, calstabin2, and rendering the channels leaky for SER Ca^2+^.^2,7,8^ Along this, SERCA2a and RyR2 are sensitive to oxidative modifications, especially on cysteine residues, leading to a reduction of SERCA2a and increase of RyR2 activities.^9,10^ Thus, a higher level of oxidative stress in HF causes impaired Ca^2+^ replenishment in the SER of cardiomyocytes and results in delayed diastolic relaxation.^11–13^

The importance of regulating the net balance of both proteins, RyR2 and SERCA2a, in maintaining heart health has inspired us to design and synthesize compounds that can simultaneously influence their critical functions in both desired directions.^14,15^ The dual activity of the new agents, which simultaneously inhibit Ca^2+^ leak through RyR2 and activate Ca^2+^ uptake through SERCA2a, was achieved by attaching cyclopropanol groups to the structures of 1,4-benzothiazepines or their analogs.^14^ 1,4-Benzothiazepine compounds, known as Rycals, are able to bind dysfunctional RyR2, reducing Ca^2+^ leakage and improving heart function.^3,16–18^ Single-particle cryo-electron microscopy revealed that the binding of Rycal ARM210 induces a conformational change in the RY1&2 domain of dysfunctional RyR2 tetrameric channels enhancing its interaction with ATP and promoting a closed channel conformation.^19^ This cooperative binding of ARM210 and ATP helps to revert the receptor from a primed state, reducing Ca^2+^ leak and improving cardiac function. By supplementing the structures of 1,4-benzothiazepines with one or two cyclopropanol groups, we were able to obtain compounds with SERCA activities comparable or superior to the activity of the known SERCA activator CDN1163.^14^

To expand the repertoire of such dual compounds, we searched for new small molecules as candidates for hybrid dual-activity drugs. One such molecule that has been reported to activate SERCA2a pumps by direct binding is 3-(3,4-dihydroxyphenyl)-2-propenoic acid or caffeic acid (CA, Figure 1a).^20^

**Figure 1:**
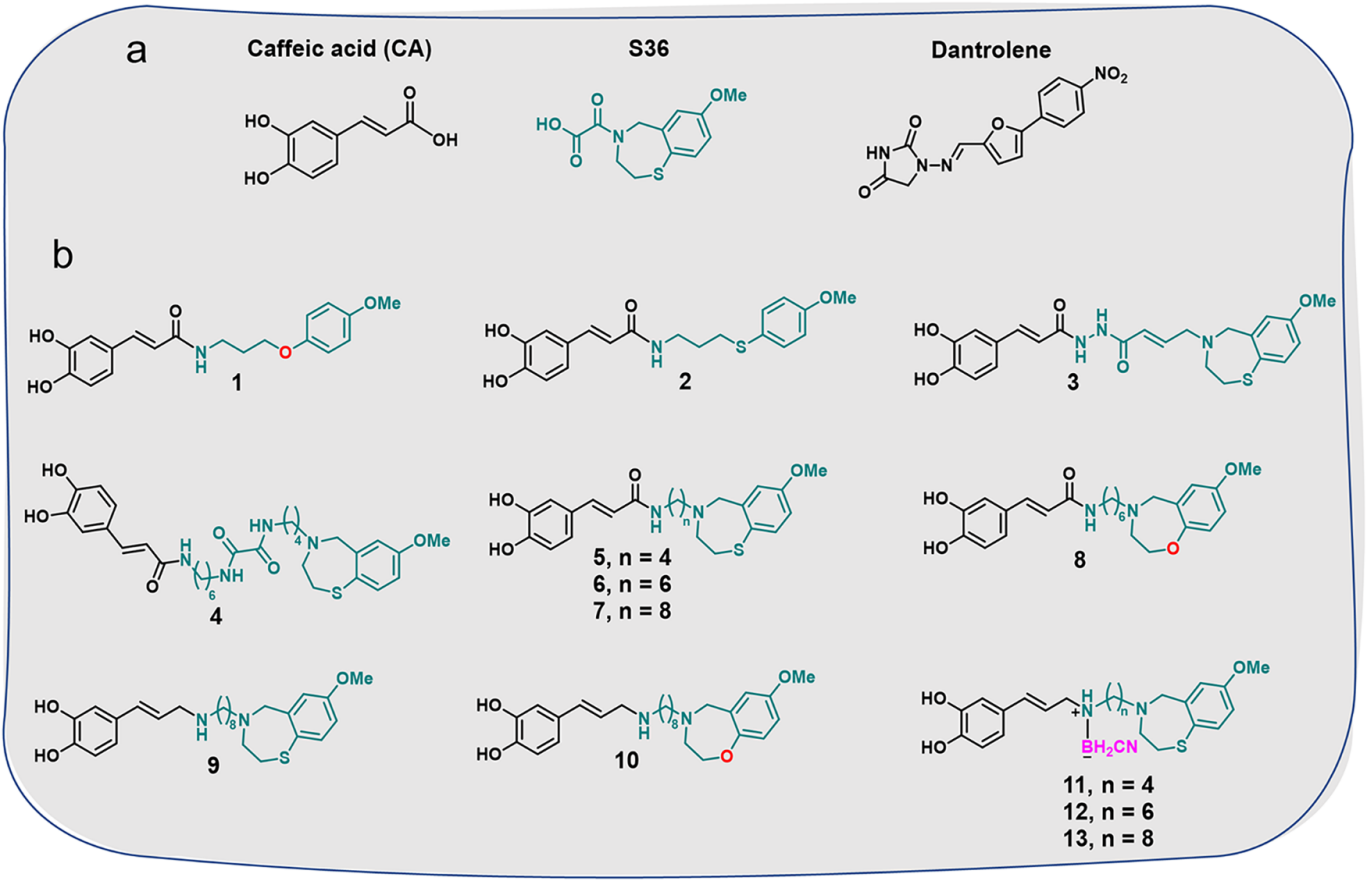
(a) Structures of CA and known RyR2 inhibitors, S36 and dantrolene; (b) synthesized 1,4-benzothia- and 1,4-benzoxazepines and related compounds.

CA counteracted thapsigargin-induced increases in intracellular Ca^2+^ levels in a concentration dependent manner and improved SR Ca^2+^ uptake.^21^ This activation of SERCA has been suggested as an additional mechanism underlying some of the cardiovascular benefits of CA. There is no direct evidence that CA can functionally affect RyR2, but its interaction with human ryanodine receptor 1 (hRyR1) has been suggested by using homology modeling together with molecular docking analysis.^22^ The authors found that CA is likely to form a relatively stable ligand-protein complex with hRyR1. In rabbits’ tracheal smooth muscle cells, CA significantly increased intracellular Ca^2+^ levels treated by the specific RyR inhibitor ryanodine, indicating that CA might act on RyR channels as an agonist.^22^

Based on this evidence, we synthesized several 1,4-benzothiazepines containing CA residues, and analyzed their effects on RyR2 and SERCA2a activities (Figure 1b). Because 1,4-benzoxazepines and 1,4-benzothiazepines are structurally related, we synthesized some benzoxazepines containing a CA moiety to test whether replacing the sulfur atom with oxygen could affect the RyR2 and/or SERCA2 activities. Two “open” analogs of 1,4-benzothia- or 1,4-benzoxazepines, 3-[(4-methoxyphenyl)­oxy]- and 3-[(4-methoxyphenyl)­thio]­propane-1-amines containing CA moiety, were synthesized as well, and their (dual) activities were studied.

Heart beat is initiated by depolarization activating the cellular Ca^2+^ cycling cascade described above. In more detail, the cardiac excitation-contraction coupling is carried by sodium influx through voltage-gated Na_V_1.5 channels that drives the membrane depolarization, which activates L-type calcium channels, leading to Ca^2+^ entry into the cell.^23^ This activates RyR2 by Ca^2+^-induced Ca^2+^ release (CICR) from the SER leading to contraction. Besides this classical cascade, nondesired activity of Na_V_1.5 was shown to directly influence cardiac Ca^2+^ homeostasis.^24^ Acquired Na_V_1.5 dysfunction/late Na^+^ current can increase the intracellular Ca^2+^ concentration during cardiac relaxation, a common cause of contractile and arrhythmogenic dysfunction in the impaired heart.^25^ Interestingly, single-particle cryo-EM has identified close structural similarity between Na_V_1.5 and RyR channels.^26^ Proteins with similar 3D structures might share functional or binding site similarities, which would potentially allow compounds active on one protein to interact with structurally similar proteins.^27^ In line with this notion, the classical Na_V_1.5 channel blocker flecainide may also directly affect RyR2 channel activity indicating drug binding similarities between both proteins,^28^ although this connection was recently questioned.^29^ We tested our compounds for their possible effects on Na_V_1.5 voltage-gated sodium channels by examining the veratridine-induced responses according to a published protocol.^30^ Consequently, we propose that several of our derivatives exhibited multitargeted drug actions on RyR2, SERCA2a, and Na_V_1.5.

## Results

### Chemistry

To prepare the caffeic acid derivatives, we used the following sequence of steps: *tert*-butyldimethylsilyl (TBDMS) protected “caffeic alcohol” **14** was synthesized from CA (ABCR GmbH, Karlsruhe, Germany) using the procedure described by Roifman et al.^31^ 3,4-Dihydroxycinnamaldehyd **15** was further obtained by the method of Dess & Martin (Scheme 1).^32^ In our previous publication, we found that “open” structural analogues of 1,4-benzothia- and 1,4-benzoxazepines can also function as RyR2 stabilizers.^14^ To test whether this conclusion is also true for CA derivatives, we prepared compounds **1** and **2** by coupling TBDMS protected CA (**16**) with 3-[(4-methoxyphenyl)­oxy]­propan-1-amine or freshly prepared 3-[(4-methoxyphenyl)­thio]­propan-1-amine (Scheme 1). Both target compounds **1** and **2** were obtained by stepwise cleavage of *tert*-butyldimethylsilyl groups, which was carried out in MeOH with KF and triethylamine hydrochloride at room temperature. Compound **3** was obtained in several steps. Alkylation of commercially available 7-methoxy-2,3,4,5-tetrahydrobenzo­[1,4-*f*]­thiazepine (BLD Pharm) with ethyl *trans*-4-bromo-2-butenoate followed by saponification of ethyl ester gave compound **17** (Scheme 1B). Then acid **17** was coupled with *tert*-butyl hydrazinecarboxylate. After cleavage of *tert*-butyl group, hydrazide **18** was acylated by TBDMS protected CA (**16**). We synthesized compounds **4**–**7** having linkers of different lengths, which connect 1,4-benzothiazepine and the caffeic acid residues. We wanted to test whether the linker length could modulate RyR2 and SERCA2 activities. Amines **19**, **22**, **23** (Scheme 2) were synthesized in two steps using *N*-(ω-bromoalkyl)­phthalimides. Compound **4** (Scheme 1C) was prepared in several steps starting from amine **19** and methyl 2-chloro-2-oxoacetate. The coupling of acid **20** with *N*-*tert*-butoxycarbonyl-1,6-hexanediamine followed by cleavage of *tert*-butyl group afforded amine **21**. Amidation of TBDMS-protected CA (**16**) with amine **21** followed by cleavage of the TBDMS groups afforded triamide **4**. Compounds **5**–**7** were obtained from amines **19**, **22**, and **23** and TBDMS-protected CA (**16**) using 2-(1*H*-benzotriazol-1-yl)-1,1,3,3-tetramethyluronium hexafluorophosphate (HBTU) as a coupling agent followed by TBDMS cleavage. Compound **8** was prepared in several steps starting from *N*-(2-bromo-5-methoxybenzyl)-2-aminoethanol **36** (Scheme S1). Compounds **9** was prepared by reductive amination of 8-(7-methoxy-2,3-dihydrobenzo­[1,4-*f*]­thiazepin-4­(5*H*)-yl)­octan-1-amine (**23**) with cinnamic aldehyde **15** (Scheme 2) in the presence of sodium triacetoxyborohydride followed by cleavage of *tert-*butyldimethylsilyl group.

**Scheme 1:**
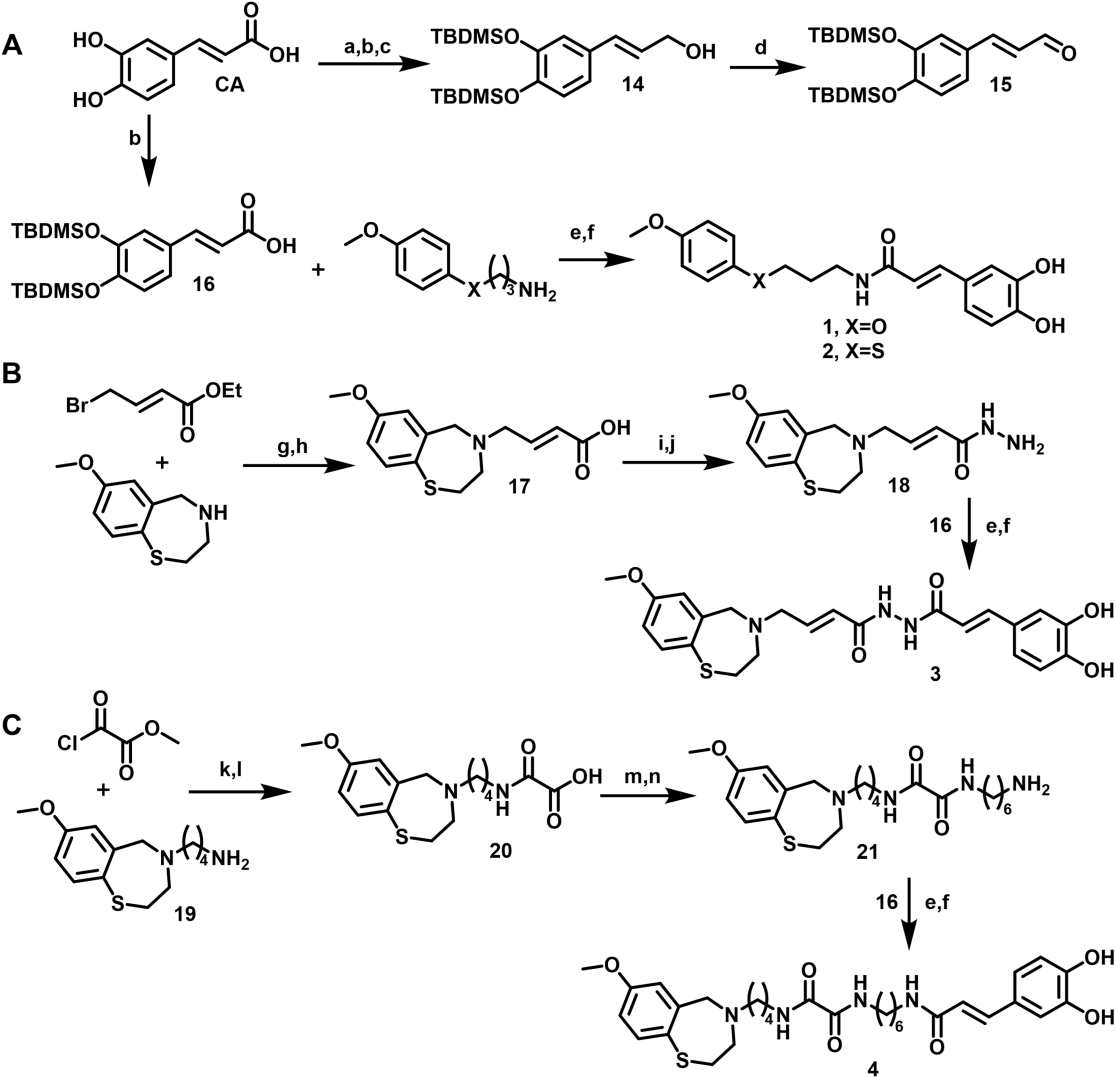
Synthesis of Compounds **1**–**4** ^a^

a Reagents and conditions: **A**. (a) SOCl_2_, MeOH; (b) TBDMS-Cl, imidazole, DMF; (c) DIBAL, THF; (d) Dess-Martin reagent, DCM; (e) HBTU, Et_3_N, THF; (f) KF, Et_3_N × HCl, MeOH. **B**. (e–f) same as in A; (g) pyridine, DCM; (h) NaOH, EtOH; (i) NH_2_NHBoc, EDCI, DMSO; (j) TFA, DCM. **C**. (e–f) same as in A; (k) methyl 2-chloro-2-oxoacetate, DCM; (l) NaOH, MeOH; (m) *N*-*tert*-butoxycarbonyl-1,6-hexanediamine, HATU, Et_3_N, DMSO; (n) TFA in DCM.

**Scheme 2:**
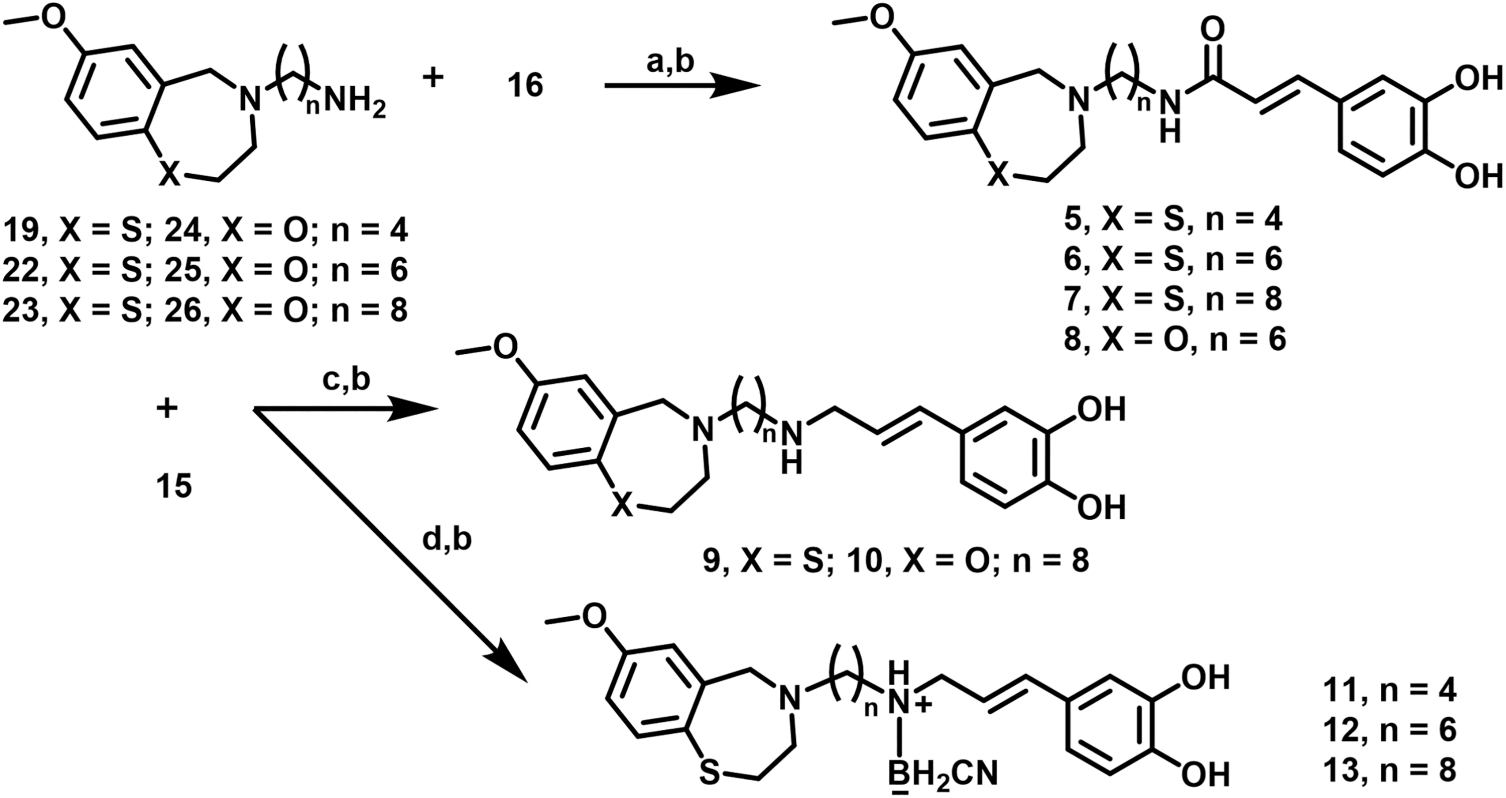
Synthesis of Compounds **5**–**13** ^a^

a Reagents and conditions: (a) HBTU, Et_3_N, THF; (b) KF, Et_3_N × HCl, MeOH; (c) NaBH­(OAc)_3_, 1,2-dichloroethane; (d) NaBH_3_CN, 1,2-dichloroethane.

Compound **10** was obtained from freshly prepared 8-(7-methoxy-2,3-dihydrobenzo­[1,4-*f*]­oxazepin-4­(5*H*)-yl)­octan-1-amine (**26**) using a procedure similar to the preparation of **9**. The attempted synthesis of compounds similar to **9** and **10** with a shorter linker (*n* = 4) was performed according to the same scheme, but did not yield the expected products.

Alternatively, we performed reductive alkylation of amines **19**, **22**, **23** with cinnamic aldehyde **15** using two equivalents of sodium cyanoborohydride (Scheme 2). Surprisingly, this method afforded TBDMS protected intermediates (**43**–**45** in Supporting information (SI)) as boran complexes stable in air and on silica gel. After cleavage of TBDMS groups, we obtained cyanoboranes **11**–**13**, which were characterized by NMR spectroscopy, including ^11^B-NMR, and additionally by LC-MS, ESI-MS and HR-MS (see SI). Zwitterionic *N*-(cyanoborane)-*N*-alkoxyamines obtained by reduction of *O*-alkyloximes with sodium cyanoborohydride and plausible reaction mechanism were reported by Màrquez et al.^33^ Stable cyano-substituted amine borane complexes obtained by the reaction of propargyl amine hydrochlorides with sodium cyanoborohydride in THF in the presence of MgSO_4_, were used in the synthesis of cyclic amine boranes.^34,35^

### Modulation of RyR2 Activity by New CA Derivatives

CA and its derivatives have numerous biological activities including antioxidant, antibacterial, anti-inflammatory, anticarcinogenic, and cardioprotective.^36–39^ The key target of CA in the heart is Keap1 (Kelch-like ECH-associated protein 1), which activation with CA leads to enhanced antioxidant defenses in cardiomyocytes and protection against oxidative damage.^40^ CA and its derivatives inhibits proteins in the renin-angiotensin-aldosterone system, contributing to blood pressure regulation, and influence cyclogenase (COX) and lipoxygenase (LOX) enzymes, reducing inflammation in cardiac tissue.^41^ CA targets transforming growth factor-β receptor 1 (TGFBR1) in infarcted hearts and inhibit TGF-β1-induced proliferation and collagen synthesis.^42^ There have been no publications on caffeic acid derivatives specifically acting as modulators of RyR2. We have examined the action of CA and its derivatives as RyR2 activators by monitoring changes in luminal ER Ca^2+^ levels in HEK-293 cells with inducible expression of wild type mouse RyR2 (WT RyR2) in which the heterologous expression of WT RyR2 leads to additional spontaneous Ca^2+^ leak from the ER.^43^ Compounds, which are able to reduce or increase the Ca^2+^ leak can be considered as modulators of RyR2 channel activity. The investigation of functional ability of CA derivatives to modulate the RyR2 channel activity was possible because the cells additionally stably expressed the Ca^2+^ fluorescence indicator R-CEPIA1*er*, which is a genetically encoded Ca^2+^ sensory protein in the ER lumen.^44,45^ Measuring ER fluorescence before and after the compound addition allowed us to identify RyR2 modulators. The presence of RyR2 was confirmed by Western blotting (Figure S1, for details see Experimental Section). The R-CEPIA1*er* fluorescence is easily detected using confocal microscopy (Figure 2a).

**Figure 2:**
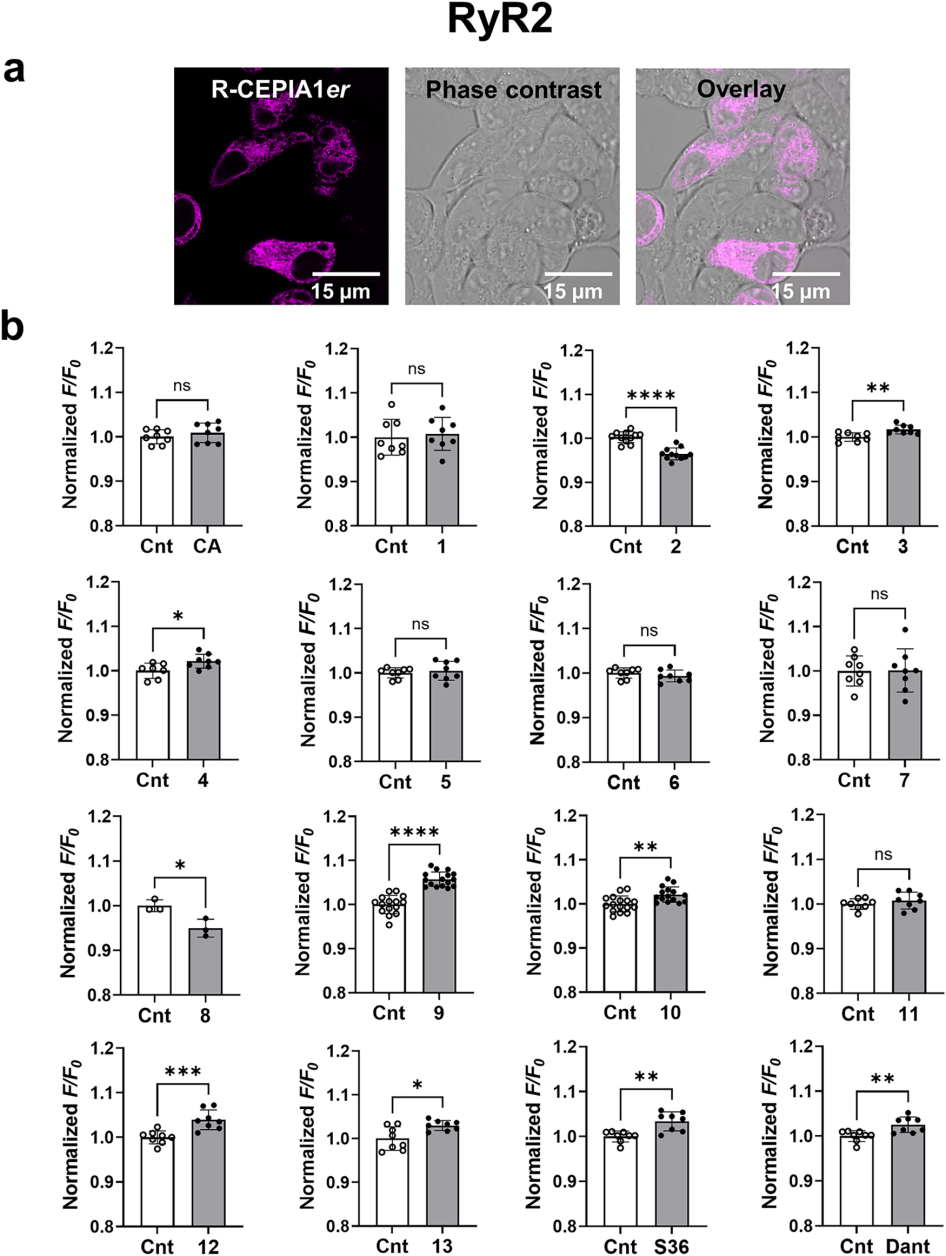
(a) Confocal images of living HEK-293 cells expressing WT RyR2 and ER Ca^2+^ indicator R-CEPIA1er. Scale bar 15 μm. The cells were imaged on confocal Leica SP8 microscope as described in the Experimental Section. (b) RyR2 activity assay in HEK-293 RyR2 R-CEPIA1*er* cells. The ratio between the average fluorescence before and after injection of 25 μM solution of a test compound (*F*/*F*_0_) was normalized to the control (Cnt, 0.1 v/v% DMSO). Values represent the mean ± SD, * *P* < 0.05, ** *P* < 0.01, **** *P* < 0.0001 vs control by unpaired *t* test *n* = 3–16 of individual measurements. CA, Caffeic acid; numbers correspond to structures of test compounds in Figure 1; Dant, Dantrolene; S36—a known RyR stabilizer (ref 18).

We performed time-resolved ER fluorescence measurements using a FlexStation 3 Multimode Microplate Reader (Molecular Devices, San Jose, CA, USA). Upon addition of tested compounds we observed changes in the concentration of Ca^2+^ that correspond to RyR2 channel activity in the ER ([Ca^2+^]_ER_) in HEK-293 RyR2 R-CEPIA1*er* cells.^46^ The calculation of the sample fluorescence ratio (normalized to control *F*/*F*_0_, where *F*_0_ is the average fluorescence over the first 90 s and *F* is the average fluorescence over the last 100 s), allowed us to compare the signals obtained from the tested compounds with those from the control sample (vehicle 0.1 v/v% DMSO). We have successfully used HEK-293 RyR2 R-CEPIA1*er* cells to identify potential RyR2 stabilizers in our previous publications.^14,15^ A hit substance, a RyR2 stabilizer, will reduce Ca^2+^ leak, resulting in an increase in R-CEPIA1*er* fluorescence. The CA derivative **1**, which with its “open” structure mimics 1,4-benzoxazepines, showed no stabilizing effect on RyR2 in this assay (Figure 2b). Compounds **3**, **4**, **9, 10, 12**, and **13** significantly increased [Ca^2+^]_ER_ in the RyR2 R-CEPIA1*er* assay, with compound **9** being most efficacious (Figure 2b). As positive controls, we used the RyR2 inhibitor dantrolene and the RyR stabilizer S36.^18^ The addition of both standard drugs to the HEK-293 RyR2 R-CEPIA1*er* cells led to an increase in R-CEPIA1*er* fluorescence validating our system. The use of 25 μM CA, as well as compounds **1**, **5, 6**, and **11**, did not affect the RyR2 activity in this model system. Compounds **2** and **8** appeared to be RyR2 activators because they significantly initiated additional Ca^2+^ leakage from the ER (Figure 2b).

### SERCA2 Activity in HL-1 Cells

In high concentrations caffeine binds to RyR2 and causes a mass Ca^2+^ release from the SER, which is indicative of a complete emptying of the intracellular Ca^2+^ store.^47^ We used this ability of caffeine to activate cardiac RyR2 in HL-1 cells treated with cytosolic Ca^2+^ indicator FLIPR Calcium 6 (Molecular Devices) to estimate intracellular SER calcium content. HL-1 cells originate from mouse atrial myocytes and retain the ability to beat and cycle Ca^2+^ similar to adult mouse cardiomyocytes.^48^ The presence of SERCA2a was confirmed by Western blotting (Figure S2, for details see Experimental section). The addition of 10 mM caffeine solution causes Ca^2+^ depletion from the SER, which can be detected by rapid increase in cytosolic fluorescence (see Figure S3 for caffeine-induced ER depletion and the controls in the cells expressing the RyR2 channel). We used CDN1163, a low molecular weight activator of SERCA,^49^ as a positive control, and incubated HL-1 cells in CDN1163 solutions of different concentrations together with cytoplasmic FLIPR Calcium 6 indicator (see Figure 3a for the experimental plan and Figure 3b for confocal microscopy of HL-1 cells stained with FLIPR Calcium 6 dye). Treatment of cells with CDN1163 solution should result in increased Ca^2+^ uptake into the SER via SERCA2 compared to untreated cells and, thus, to an increased SER Ca^2+^ load. Indeed, after caffeine application the cells pretreated with CDN1163 showed a much larger increase in fluorescence signal compared to the control (0.1 v/v% DMSO) because more Ca^2+^ was released from the SER confirming that CDN1163 increases SERCA2 activity. The half-maximal effective concentration of CDN1163 (EC_50_) is 21 μM, which is consistent with the literature value (Figure 3c).^50^

**Figure 3:**
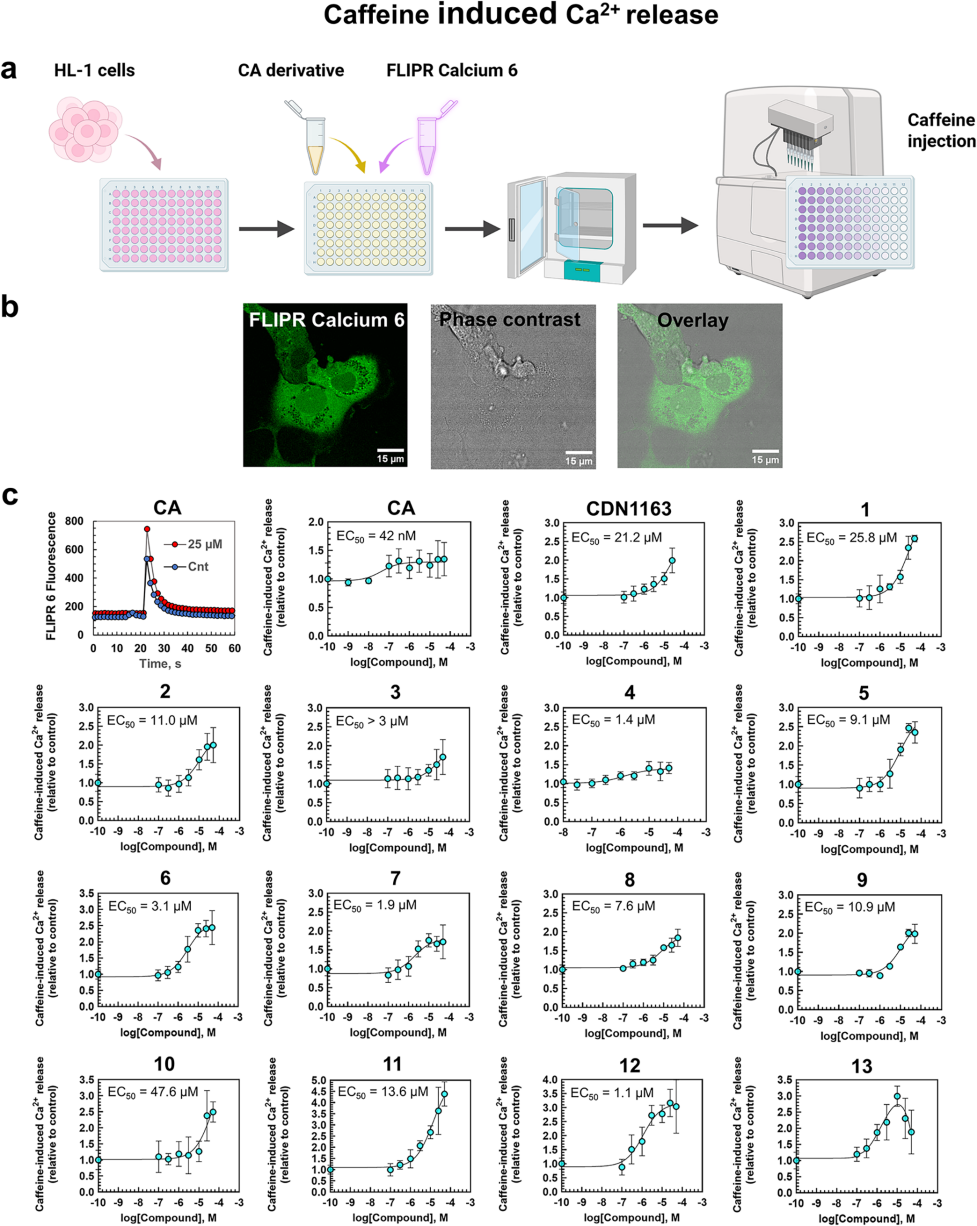
Caffeine induced calcium release assay on live HL-1 cardiomyocytes. (a) Experimental plan. HL-1 cells were incubated with the FLIPR 6 Ca^2+^ indicator in the presence of various concentrations of CA or test compounds. Ca^2+^ influx was initiated by the addition of 10 mM caffeine. The chart is created with BioRender.com. (b) Confocal images of living HL-1 cells stained with FLIPR Calcium 6 dye. Scale bar 15 μm. The cells were imaged on confocal Leica SP8 microscope as described in the Experimental Section. (c) The representative curve of the fluorescence change after caffeine addition (upper left) and the dose–response curves of CA, SERCA activator CDN1163, and compounds **1**– **13**. The data were normalized to the control (Cnt, 0.1 v/v% DMSO). Values represent the mean ± SD, *n* = 8–16 of individual measurements.

As in the case of CDN1163, the effect of caffeine on HL-1 cells was enhanced compared to the control (0.1 v/v% DMSO), when the cells were preincubated with CA. CA improved SERCA2 activity even in the nanomolar concentration range, albeit with much lower efficacy than CDN1163 (Figure 3c). CA molecules emit light with the peak intensity at 460 nm. The intensity of this fluorescence is influenced by pH, primary due to the dissociation of its phenolic hydroxyl groups (Figure S4). To ensure the accuracy of our results and avoid false positives, we evaluated the fluorescence of CA in HL-1 cells without the FLIPR Calcium 6 dye (Figure S5). This step was crucial in distinguishing the true fluorescence signal from potential background emission. However, since we preincubated the cells with CA 2 h before starting the Ca^2+^ recordings, we noticed an increase in background fluorescence which left the effect of caffeine unchanged. When attached to 1,4-benzothiazepine or 1,4-benzoxazepine, the CA residue largely lost its fluorescence. For example, compound **9** showed almost no fluorescence compared to the CA (Figure S6).

An increase in concentration led to stronger caffeine-induced signals for all the synthesized compounds. Their EC_50_ values are in the micromolar range, and the efficacies of some compounds are superior to that of CDN1163. The EC_50_ values for **1**–**12** are in the micromolar range, and borane complex **12** provides the largest potency of all synthesized caffeic acid derivatives (EC_50_ = 1.1 μM). Compound **11** is the most active of all compounds and demonstrates up to 450% increase in the signaling response to caffeine stimulation compared to the control sample. Boron complex **13** displayed a bell-shaped dose response; it has shown a good stimulatory effect from 1 to 10 μM (up to 300% compared to the control) that decreased at higher doses (Figure 3c).

### SERCA2a Activity in Microsomal Membrane Vesicles

So far, we monitored cellular SERCA2a activity indirectly by assessing the SER Ca^2+^ content that is filled up by SERCA2a mediated Ca^2+^ uptake. Since the SERCA2a mediated Ca^2+^ transport is ATP dependent (due to its endogenous ATPase Ca^2+^ pump activity), we additionally studied whether CA and the synthesized derivatives could directly modulate the ATPase activity in the SERCA2a containing vesicles.^51^

To this end, we tracked the rate of nicotinamide adenine dinucleotide (NADH) oxidation linked to ATP hydrolysis by observing changes in NADH fluorescence over time. This was done after addition of ATP to solutions containing microsomal membranes enriched with SER vesicles isolated from adult mouse heart ventricles (mouse SER).^14^ The NADH fluorescence decrease was recorded on a FlexStation 3 Multimode Microplate Reader. Consistent with our previous experiment on HL-1 cells, 10 μM CA accelerated consumption of NADH, indicating enhanced SERCA2a activity in mouse SER (*n* = 5) (Figure 4a). The treatment of cardiac SER vesicles with compounds **1**, **3**, and **5** did not evoke a significant increase in ATPase activity, while compounds **2**, **4**, **6**–**13** showed significant activation of SERCA2a ATPase (*n* = 3–6) (Figure 4a). We detected the highest increase in ATPase activity (up to 118%), when the vesicles were treated with borate complex **11**–**13** (Table 1). We tested compounds **11**–**13** in the NADH assay buffer to see whether these compounds could contribute to the rate of NADH fluorescence reduction (Figures S7–S9). No significant deviations were found in the rate of NADH consumption compared to the control sample (0.1 v/v% DMSO). Compounds **9**, **11**–**13** evoked an enhancement in the kinetic rate of NADH–NAD^+^ conversion in a dose-dependent manner, the EC_50_ values lie in the nanomolar range: 41, 30, 2.3, and 23 nM respectively (Figure S10).

**Figure 4:**
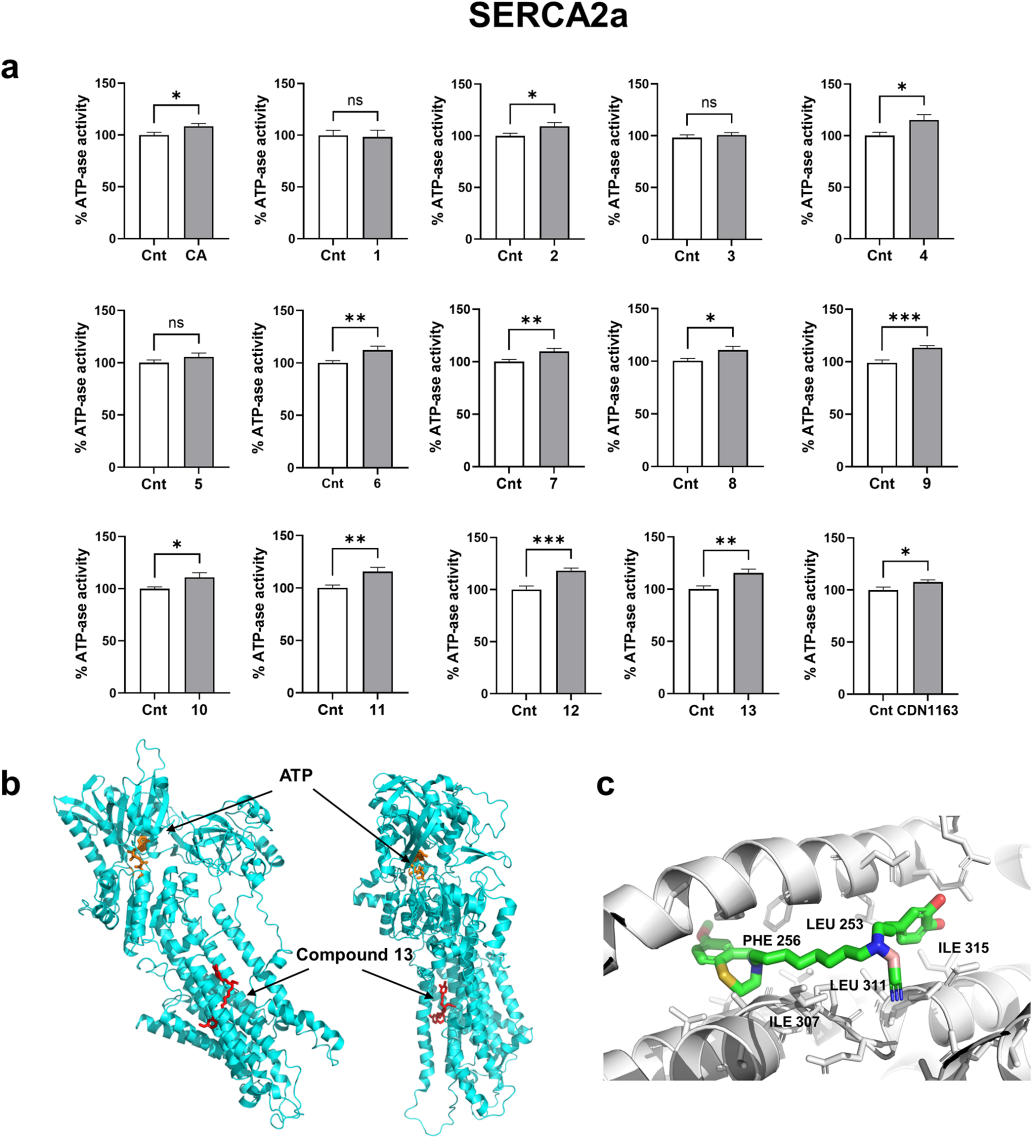
(a) Effect of 10 μM CA and its derivatives as well as CDN1163 on SERCA2a activity in mouse SER. The data were normalized to the control (Cnt, 0.1 v/v% DMSO). Values represent the mean ± SEM, * *P* < 0.05, ** *P* < 0.005, *** *P* < 0.001 vs control by Student’s *t* test; *n* = 12–31/3–6 mice. (b) Compound **13** (red) in the human SERCA2a in E2.ATP state (pdb 7BT2, ref 52). ATP molecule in the N domain of SERCA2a is indicated in orange. Molecular docking was done using DiffDock-L (Neurosnap Inc., Delaware, USA). The DiffDock confidence is −1.3 (moderate); the SMINA Minimized Affinity is −7.32; the minimized RMSD is 1.62 (good). Data visualization was done using Pymol, Molecular Graphics System, Version 3.1.2 (Schrödinger, LLC, New York, USA). (c) Predicted binding mode of compound **13**. Carbon atoms are indicated in gray (for SERCA2a) or green (for compound **13**), nitrogen atoms are in blue, oxygens in red and sulfur in yellow.

**Table 1:** Properties of S36, Caffeic Acid (CA), and Compounds **1**–**13**

compound	MW	log*P* ^a^	*P*_e_ ^b^ 10^–6^ cm/s	RyR2 activity^c^, %	activation of SERCA2 in HL-1^c^, % (EC_50_, μM)	ATPase activity of SERCA2a in mouse SER^c^, % (EC_50_, nM)	Na_V_1.5 activity in CHO cells^c^
S36	267	1.214	1.0 (low, ref 14)	103.4 ± 0.7	–	n.d.	n.d.
CA	180	1.196	n.d.	100.9 ± 0.8	123.7 ± 6.0 (0.04)	108.5 ± 2.5	91.8 ± 2.4
1	343	2.705	1.8 (medium)	100.8 ± 1.3	157.7 ± 7.1 (14.9)	98.2 ± 6.5	91.8 ± 1.0
2	359	3.418	<1.0 (low)	96.5 ± 0.4	161.5 ± 9.5 (11.0)	109.2 ± 3.6	89.7 ± 1.1
3	455	2.431	3.4 (medium)	101.7 ± 0.3	135.4 ± 3.8 (>3.0)	100.6 ± 2.5	94.8 ± 2.1
4	598	3.417	2.7 (medium)	102.2 ± 0.6	140.3 ± 6.4 (1.4)	110.8 ± 4.5	108.0 ± 1.6
5	428	3.624	0.9 (low)	100.5 ± 0.8	190.6 ± 6.6 (9.1)	109.0 ± 4.0	97.7 ± 1.9
6	456	4.404	0.9 (low)	99.4 ± 0.4	235.4 ± 8.5 (3.1)	112.3 ± 3.5	88.8 ± 0.9
7	484	5.184	0.9 (low)	100.1 ± 1.7	175.3 ± 7.2 (1.9)	109.8 ± 2.9	87.4 ± 1.1
8	440	3.691	1.1 (low)	95.0 ± 1.2	158.1 ± 2.4 (7.6)	110.5 ± 3.5	96.6 ± 3.6
9	470	5.658	0.4 (low)	105.7 ± 0.4	164.0 ± 3.0 (10.9)	113.1 ± 2.1 (41.2)	106.0 ± 1.0
10	454	4.944	0.2 (low)	102.1 ± 0.4	126.4 ± 13.0 (47.6)	112.3 ± 4.6	103.0 ± 1.4
11	453	2.225	2.6 (medium)	100.8 ± 0.7	267.3 ± 11.7 (13.6)	115.7 ± 4.0 (29.6)	83.8 ± 1.7
12	442	3.005	0.4 (low)	103.9 ± 0.8	277.8 ± 12.7 (1.1)	118.2 ± 2.5 (2.3)	86.2 ± 2.6
13	509	3.786	0.1 (low)	103.0 ± 0.4	299.1 ± 12.8 (2.5)	115.5 ± 3.7 (23.1)	83.3 ± 0.9

a Wildman-Crippen log*P* values were evaluated using ADMETlab.

b *P*_*e*_ permeability rate.

c Percentage from the control sample (100%) at a given concentration: 25 μM in RyR2 R-CEPIA1*er* assay; 10 μM in caffeine, membrane potential assays and NADH assay on mouse SER. Values represent the mean ± SEM.

Figure 4b represents two views of the human SERCA2a Ca^2+^ pump in the E2.ATP form (pdb 7BT2)^52^ with compound **13**. The Ca^2+^ -free E2 SERCA2a form is a binding state with a low affinity for Ca^2+^ and faces the SER lumen. ATP, which binds to the nucleotide-binding (N) domain and near the phosphorylation (P) domain of SERCA2a, arranges the cytoplasmic domains into the configuration suited for phosphoryl transfer.^52^ We performed AI-assisted protein–ligand blind docking for E2.ATP SERCA2a and compound **13** using DiffDock-L (Neurosnap Inc., Delaware, USA).^53–56^ Neurosnap DiffDock-L uses diffusion generative models to predict ligand poses. Out of 100 proposed ligand-protein visualizations we selected those that exhibited root mean squared deviation (RMSD) of less than 2 Å, ensuring a higher degree of structural alignment. RMSD reflects the geometric similarities between the predicted pose and a reference structure, which in blind docking is the 3D structure of the target protein.^54^ From these preselected visualizations, we have chosen the one with lowest affinity, prioritizing the most favorable binding interaction. The calculated RMSD for compound **13** in the selected conformation and location is 1.62 Å; the minimized affinity, calculated using SMINA AutoDock Vina algorithm, is −7.32 kcal/mol, which suggests a stable protein–ligand complex. Another factor, which we took into consideration, is a DiffDock confidence. This is a score that evaluates the reliability of predicted ligand-protein docking pose. The moderate Diff-Dock confidence of −1.3 calculated for the proposed binding mode suggests a reasonable likelihood of successful docking. The proposed binding mode of compound **13** in the transmembrane domain site of E2.ATP SERCA2a is given in Figure 4c. The AI-assisted molecular docking for CDN1163, compounds **9, 11**, and **12** in the E2.ATP state of SERCA2a gave similar results (Figures S11 and S12). The identified potential activator binding site is in the transmembrane domain of SERCA2a. The SMINA Minimized Affinities for the selected visualizations for compounds **9** and **11** are −7.49 and −7.45 kcal/mol indicating that the potential ligand-protein complexes are stable. The SMINA minimized affinity for the selected visualization of compound **12** in E2.ATP SERCA2a is −8.15 kcal/mol, which means that this complex can be highly stable. The assessed DiffDock confidences are moderate: the largest for compound **9** (−0.64) followed by −0.99 for compound **11** and −1.2 for compound **12**. The resulting RMSDs of 1.99 Å for compounds **9**, 1.45 Å for **11** and 1.56 Å for **12** meet the criterion for the accurate prediction. Earlier, Paula et al. identified three potential SERCA protein sites that can be occupied by CDN1163 to accelerate the SERCA catalytic cycle: in the transmembrane domain, in the A domain of the enzyme, close to the cytosol/membrane interface, and in the close proximity to the ATP binding pocket.^57^ The highest binding affinity was computed for CDN1163 in the transmembrane domain site. Given the very lipophilic nature of this compound (calculated log*P* = 5.08 ± 0.57, ACD Laboratories), it is highly likely that the CDN1163 site of action is located within the transmembrane domain, as also proposed for our compounds **9**, **11**, **12**, and **13**.

### Na_V_1.5 Activities in CHO Cells

Sodium channel Na_V_1.5, encoded by the *SCN5A* gene, is the predominant cardiac Na^+^ channel and plays a central role in generating and propagating action potentials in cardiac myocytes and muscles, respectively.^58,59^ Like other voltage-gated sodium channels, it mediates a rapid influx of Na^+^ ions that initiates the cardiac action potential in phase 0.^23,60,61^ Due to its main function, namely the orderly conduction of electrical activity through the heart, Na_V_1.5 has become an important target for antiarrhythmic drugs. For example, flecainide and quinidine are known to directly block this ion channel (class I antiarrhythmic agents).^23,62^ Flecainide is also found to reduce cardiac RyR2-mediated SER Ca^2+^ release when used in concentrations above 5 μM.^28^

To test whether the synthesized CA derivatives can affect cardiac sodium channels, we used a fluorescence-based sodium flux assay on CHO Na_V_1.5 Duo cells, which stably express human Na_V_1.5 (B’SYS GmbH, Switzerland). According to the manufacturer, the human Na_V_1.5 cDNA was cloned and transfected into CHO cells, and the functional properties of the Na_V_1.5 channels were subsequently confirmed. The Na_V_1.5 expression was proved by Western blotting and immunostaining (Figures S2 and S13). We performed the FLIPR membrane potential assay (Molecular Devices, USA) using a Blue Assay Kit and recorded changes in fluorescence, as the cell membranes depolarized after veratridine injection. Veratridine is a neurotoxin that acts on voltage-gated Na^+^ channels preventing their inactivation and leading to increased Na^+^ influx.^63^ Depolarization of cell membranes, which occurs due to the rapid entry of Na^+^ ions into cells, results in increased fluorescence. As a positive control we used tetracaine as an intracellular Na^+^ channel blocker; the maximal inhibition was reached at 100 μM (Figure 5).

**Figure 5:**
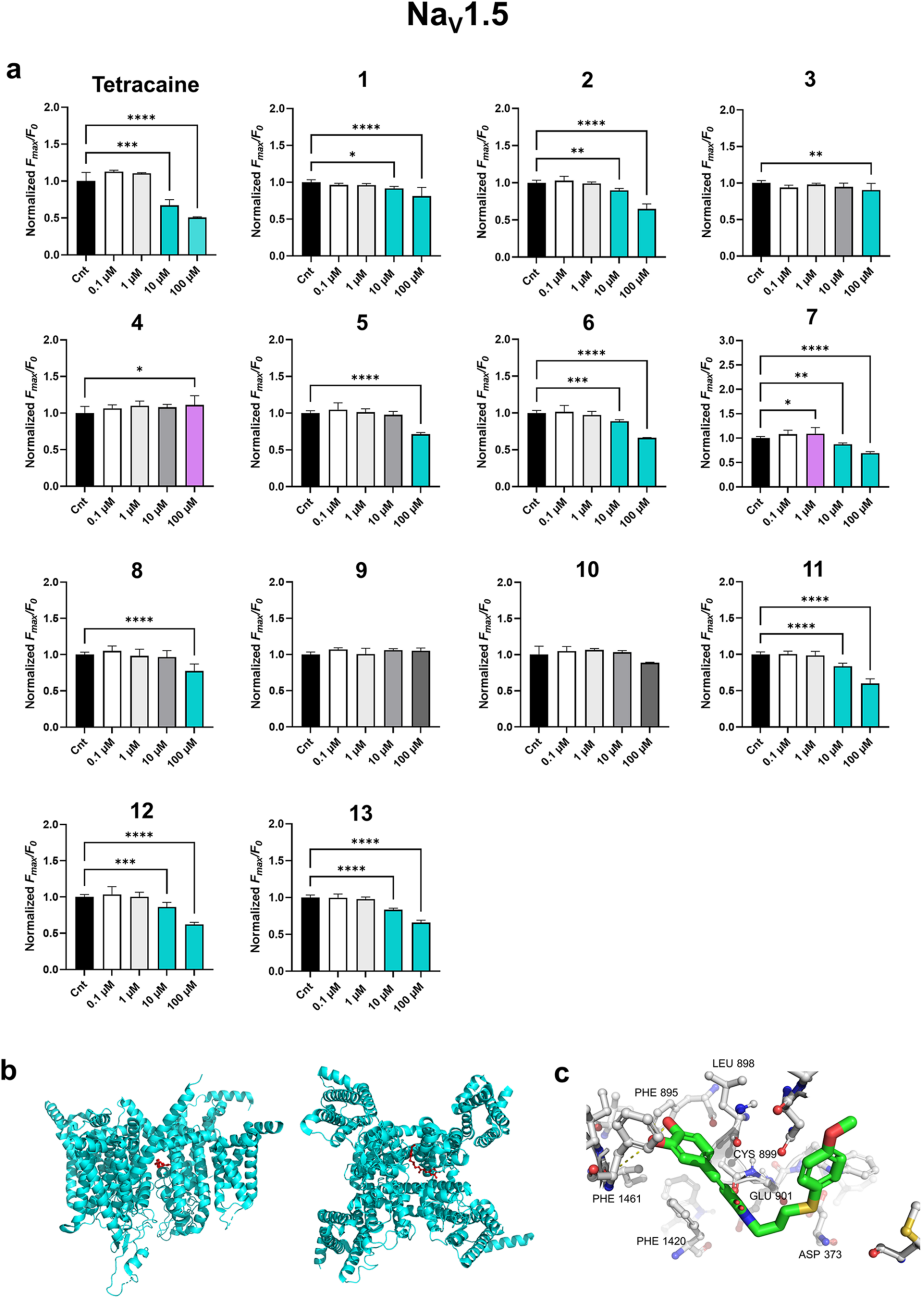
(a) FLIPR membrane potential assay on CHO Na_V_1.5 Duo cells. The data were normalized to the control (Cnt, 0.1 v/v% DMSO). Values represent the mean ± SD * *P* < 0.05, ** *P* < 0.005, *** *P* < 0.001, **** *P* < 0.0001 vs control by one-way ANOVA test, *n* = 3–6 of individual measurements. Cyan bars correspond to the Na_V_1.5 blocking activities; magenta bars—to stimulating activities. (b) Compound **2** (red) in the cardiac Na_V_1.5 from rat heart (α-subunit, pdb 6UZ0, ref 23). Molecular docking was done using DiffDock-L (Neurosnap Inc., Delaware, USA), DiffDock Confidence: −0.97 (moderate). Data visualization was done using Pymol, Molecular Graphics System, Version 3.1.2 (Schrödinger, LLC, New York, USA). (c) Predicted binding mode of compound **2**. Carbon atoms are indicated in gray (for Na_V_1.5) or green (for compound **2**), nitrogen atoms are in blue, oxygens in red and sulfur in yellow.

In the concentration range from 0.1 to 100 μM, both CA and the RyR stabilizer S36 had no effect on Na_V_1.5 activity (Figure S14). To our surprise, under the given experimental conditions, CDN1163 blocked Na_V_1.5 in in a concentration-dependent manner (Figure S14, Table 1). The effect of 100 μM CDN1163 was comparable to the maximal effect of tetracaine. Thus, in addition to the beneficial effect of improving SERCA2a activity, CDN1163 may also act as a mild class I antiarrhythmic agent.

Likewise, we observed that some of our new compounds, which improve SERCA2a activity, affect Na_V_1.5 functions in our assay system (Figure 5a). We have identified compounds **1**–**3**, **5**–**8**, and **11**–**13** as Na_V_1.5 blockers that inhibit Na^+^ channels at concentrations higher than 10 μM (Figure 5a). Only two of the synthesized CA derivatives—compounds **9** and **10**—had no effect on Na_V_1.5 in the concentration range of 0.1 to 100 μM (Figure 5a). Compound **4** at a concentration of 100 μM had a small but significant activating effect. Compound **7** showed a bell-shaped behavior with an activating effect at a concentration of 1 μM and an inhibitory effect at concentrations in the range of 10–100 μM. Thus, our data suggest that we have identified a new substance class, boron complexes **12** and **13**, with three multitargeted drug activities toward RyR2, SERCA2a and Na_V_1.5.

Figure 5b illustrates the side and front views of the Na_V_1.5 channel with the four domains along with their proposed voltage-sensing and pore forming segments (pdb 6UZ0).^23^ AI-assisted protein–ligand docking for Na_V_1.5 and compound **2** was performed using DiffDock-L (Neurosnap Inc., Delaware, USA).^53^ The DiffDock Confidence is moderate (−0.97). The proposed binding mode of compound **2** is demonstrated in Figure 5c.

### Drug-Related Cell Toxicity and Membrane Permeability

The CA derivatives **1**–**8** and **11**–**12** showed no significant cell death at concentrations up to 10 μM in CytoTox-Glo Cytotoxicity Assay (Promega GmbH, Figure S15). The viability of HL-1 cells treated with compounds **9** and **13** for 24 h remained unchanged at concentrations up to 1 μM.

We determined permeability rates (*P*_*e*_), which are a measure of how fast a compound can cross a membrane, using cell-free transport models (PermeaPad, innoME GmbH) The CA derivatives have medium to low permeability rates in the range 1.0–24.0 nm/s (Table 1). Similar to S36,^14^ despite poor permeability, compounds **2**–**4**, **8**, and **9** acted on live RyR2-HEK-293 R-CEPIA1*er* cells inhibiting RyR2. In addition, all compounds increased a caffeine-induced Ca^2+^ release effect on HL-1 cells and demonstrated Na_V_1.5 blocking in FLIPR membrane potential assay on CHO Na_V_1.5 Duo cells. Since PermeaPad is an artificial model that primarily reflects passive diffusion, it is likely that additional mechanisms contribute to the crossing of cell membranes by these compounds.

The properties of CA and the new compounds together with the properties of the known Rycal S36 are given in Table 1. The Wildman-Crippen values of partition coefficients (log*P*)^64^ for CA and the synthesized derivatives were calculated using ADMETlab online platform (CBDD Group, Xiangya School of Pharmaceutical Sciences & Central South University).^65^

## Discussion and Conclusions

By adding CA residues to the 1,4-benzothia- or 1,4-benzoxazepines, we aimed to obtain dual-function drugs that primarily inhibit RyR2 activity while enhancing SERCA2a action. According to our results, increasing the linker length between its functional moieties (CA and 1,4-benzothia- or 1,4-benzoxazepine) allowed us to obtain RyR inhibitors. 1,4-Benzoxazepine **10** with C_8_H_16_ linker and 1,4-benzothiazepines **9**, **12**, and **13** with C_8_H_16_ and C_6_H_12_ linkers exhibited significant impact on RyR2 activity (Figure 2b, Table 1). The 1,4-benzoxazepine **8**, which is a 1,4-benzoxazepine derivative with C_6_H_12_ linker, as well as compound **2**, which is an “open” analog of 1,4-benzothiazepine directly bound to CA, accelerated the leakage of Ca^2+^ from ER demonstrating activities opposite to the desired ones in respect of RyR2. Compound **3** with the shortest N_2_H_4_ linker has the smallest inhibitory effect on RyR2 followed by compound **4** with the longest C_4_H_8_NH­(CO)_2_NHC_6_H_12_ linker. Among three synthesized cyanoborane derivatives **11**–**13**, only compound **11** with the C_4_H_8_ linker did not show any significant changes in RyR2 activities (Figure 2b, S1). Despite the structural similarity between compounds **7** and **9**, compound **9** containing no carbonyl group showed a clear inhibitory effect on RyR2. The differences in these compounds’ action can be explained by the structural and chemical properties of amide and amine functional groups, which influence how the compounds interact with biological targets. The amine group is more basic, allowing binding with acidic sites in proteins, which can be crucial for biological activity. On the other hand, the amide bond makes the molecule more rigid and it may not fit the binding site as effectively as the similar molecule with amine bond.

Polyphenols are well-known for their antioxidant properties, capable of scavenging free radicals and reducing oxidative stress.^20^ Oxidative stress can impair SERCA function by causing structural damage to the protein, particularly through the irreversible oxidation of key cysteine residues such as Cys 674.^9^ This oxidative modification reduces SERCA activity and impairs muscle relaxation and contractility. Therefore, the antioxidant effects of polyphenols could indirectly enhance SERCA activity by mitigating these damaging effects. However, attachment of bulky substitutions to the side chain of CA, like in our case (molecular masses of 1,4-benzothiazepines/1,4-benzoxazepines are about 2 times larger than that of CA residue), results in compounds with reduced reactivity of catechol group and alters the molecule’s physicochemical properties, decreasing nonspecific interactions and assay interference.^41^ Nonetheless, to investigate SERCA activity and minimize redox-based artifacts, we specifically used caffeine-induced calcium release assay, which is less sensitive to redox modulation and more reflective of functional SR calcium load and SERCA activity. The effects of our compounds were rapid and concentration-dependent, suggesting a pharmacological interaction rather than a delayed antioxidant response.

Our results show that the covalent binding of 1,4-benzothiazepines or their “open-chain” analogues, as well as 1,4-benzoxasepines with CA or its aldehyde ((*E*)-3-(3,4-dihydroxyphenyl)­acryl aldehyde) leads to compounds that increase SERCA2 activity (Figures 3, 4). The increase in SERCA2 activity was particularly clear in the case of cyanoborane derivatives **11**–**13**. Zwitterionic cyanoborane **11** demonstrated superior effect in the caffeine-induced Ca^2+^ release assay on live HL-1 cardiomyocytes, which was greater than that of the known SERCA2 activator CDN1163 by 2.5-fold (Figure 3). Compound **11**, and its homologues **12** and **13**, demonstrated exceptional ability to activate SERCA2a in the mouse cardiomyocyte SER (Figure 4). This result may open new perspectives in biochemical research and drug development. The substantial SERCA2 activation relates to the presence of polar (zwitterionic) N–B fragments, which can enhance the binding affinity to SERCA2 and result in a more efficient activation. Due to their substantial impact on SERCA2 activation, zwitterionic cyanoboranes represent a promising starting point for further studies. Their unique properties not only contribute to our understanding of structure activity relationship but also indicate new routes in drug design.

The AI assisted blind docking of compounds **9**, **11**–**13** indicates that the compounds occupy SERCA2a sites in the transmembrane domain of this protein. The results provide the useful insights by narrowing down potential binding poses for future refinement of validation through additional computational or experimental methods.

HL-1 cells are known to express cardiac SERCA2a.^66^ Compounds **1** and **5** increased SERCA2 activity in HL-1 cells, showing EC_50_ values of 15 and 9 μM, respectively. However, these compounds did not demonstrate the SERCA2a stimulating activity on mouse cardiomyocyte SER, as well as RyR2-stabilizing effect on HEK-293 RyR2 R-CEPIA1*er* cells. HL-1 cells are in fact derived from mouse atrial cardiomyocytes that coexpress SERCA2a and SERCA2b.^67,68^ We assumed that such behavior of compounds **1** and **5** is associated with their selective action on additional SERCA2 isoform present in these cells. To prove it, we performed the NADH fluorescence-coupled ATPase assay on microsomal membranes isolated from HEK-293T cells. Indeed, both compounds significantly increased the ATPase activity, which may indicate that compounds **1** and **5** could be considered as selective activators of SERCA2b (Figure S16).

Among all derivatives, compound **4**, which is a 1,4-benzothiazepine derivative with the longest C_4_H_8_NH­(CO)_2_NHC_6_H_12_ linker, showed the lowest efficacy in the HL-1 assay. The potency in this assay was greater for the compounds with a longer linker between the CA part and the 1,4-benzothiazepine or 1,4-benzoxazepine residues.

The 1,4-benzothiazepine derivatives **4**, **9**, **10, 12**, and **13** showed dual RyR2/SERCA2a activity. By stabilizing the RyR2 closed state, these compounds can inhibit SER Ca^2+^ leak associated with muscle dysfunction and heart failure. Additionally, accelerating SERCA2a improves Ca^2+^ reuptake into the SER, enhancing muscle relaxation and reducing arrhythmogenic risk.

Among the dual RyR2-SERCA2a agents, only compounds **9**, and **10** exhibited no effect on Na_V_1.5 activity over the entire range of concentrations (0.1–100 μM) These compounds have similar structures: in compound **9**, CA is attached via a C_8_H_16_ linker to 1,4-benzothiazepine and in compound **10**—to 1,4-benzoxazepine. Blocking Na_V_1.5 decreases the rate of cardiac depolarization and slows conduction velocity, which may result in, for example, the QRS interval prolongation on the electrocardiogram.^69^ In general, nonselective off-target blockage of the hNa_V_1.5 channel is considered critical for drug candidates.^70^ However, compounds that inhibit Na^+^ current (early) peak can also inhibit the late sodium current (I_NaL_), which occurs when Na_V_1.5 channels slowly open or are inactivated due to altered genetic and acquired Na^+^ channel dysfunction.^71^ The effect on I_NaL_ is generally higher than that on the early Na^+^ current. Because inhibition of I_NaL_ has several therapeutic advantages,^72^ further detailed investigations of how CA derivatives may modulate I_NaL_ are necessary. From a therapeutic viewpoint, no safety concerns were observed by increasing SERCA2a activity in patients treated with AAV1/SERCA2a cardiac gene therapy^73^ or in clinical trials with istaroxime, a combined Na^+^/K^+^ pump inhibitor and SERCA2a activator.^74^ We propose that our compound **12** may represent a lead substance for simultaneously modulating RyR2, SERCA2a, and Na_V_1.5 activity with moderate efficacy (at 10 μM) on RyR2 and Na_V_1.5 inhibition and strong efficacy on SERCA2a activation. However, for therapeutic use, the corresponding efficacies require further validation and fine-tuning in complex systems, i.e., diseased cardiac organoid or animal models.

## Experimental Section

### Chemistry

#### General Materials and Methods

All reagents and solvents were purchased from commercial sources and used without further purification. Flash chromatography was performed using Biotage Selekt (Biotage Sweden AB, Uppsala, Sweden) flash purification system with cartridges and solvent gradients indicated below. NMR spectra were recorded at ambient temperature on Agilent 400-MR spectrometer (MPI NAT Göttingen) at 400 MHz (^1^H) and 101 MHz (^13^C). The chemical shifts are reported in ppm. All spectra are referenced to tetramethylsilane (δ = 0 ppm) using the signals of the residual protons of CHCl_3_ (7.26 ppm) in CDCl_3_, CHD_2_CN (1.94 ppm) in CD_3_CN, CHD_2_OD (3.31 ppm) in CD_3_OD or DMSO-*d*_5_ (2.50 ppm) in DMSO-*d*_6_. ^13^C spectra are referenced to tetramethylsilane (δ = 0 ppm) using the signals of the solvent: CDCl_3_ (77.16 ppm), CD_3_CN (1.32 ppm), CD_3_OD (49.00 ppm) or DMSO-*d*_6_ (39.52 ppm). Multiplicities of signals are described as follows: s = singlet, br. s = broad singlet, d = doublet, q = quartet, dq = doublet of quartets, m = multiplet or overlap of signals. Low resolution mass spectra (50–3500 *m*/*z*) with electrospray ionization (ESI) were recorded on a Varian 500-MS spectrometer (Agilent) at MPI NAT Göttingen. Copies of ^1^H, ^11^B and ^13^C NMR spectra are available in the Supporting Information (Figures S17–S60). High resolution mass spectra (HRMS) were recorded on a MICROTOF spectrometer (Bruker) equipped with ESI ion source (Apollo) and direct injector with LC autosampler Agilent RR 1200 in the Institute of Organic and Biomolecular Chemistry (Georg-August-Universität Göttingen).

Liquid chromatography: analytical HPLC was performed with Knauer AZURA liquid chromatography system. Analytical column: Knauer Eurosphere II 100-5, C18-H, 5 μm, 150 × 4.6 mm (unless stated otherwise); solvent A: H_2_O + 0.1% v/v TFA, solvent B: MeCN + 0.1% v/v TFA; temperature 25 °C. All final compounds display HPLC peak area >95%, Figures S61–S73. UltiMate 3000 Standard (SD) HPLC Systems with ISQ EM Single Quadrupole Mass-Spectrometer were employed in LC-MS. Column: Phenomenex (2.6 μm, 3.0 × 75 mm). Flow rate: 0.5 mL/min. UV detection wavelength: 254 nm. solvent A: H_2_O + 0.1% formic acid, solvent B: MeCN + 0.1% formic acid; temperature 25 °C. Analytical TLC was performed on MERCK ready-to-use plates with silica gel 60 (F_254_).

Compound S36 was obtained in two steps from 7-methoxy-2,3,4,5-tetrahydrobenzo­[1,4-*f*]­thiazepine (BLD Pharm, Kaiserslautern, Germany) as described by Marks et al.^18^

(*E*)-3-(3,4-Bis­((*tert*-butyldimethylsilyl)­oxy)­phenyl)­prop-2-en-1-ol (**14**) was obtained in three steps from 3,4-dihydroxybenzeneacrylic acid as described by Roifman et al.^31^

(*E*)-3-(3,4-Bis­((*tert*-butyldimethylsilyl)­oxy)­phenyl)­acrylaldehyde (**15**) was obtained from (*E*)-3-(3,4-bis­((*tert*-butyldimethylsilyl)­oxy)­phenyl)­prop-2-en-1-ol **14** (261 mg, 0.66 mmol) and Dess–Martin periodinane (TCI, 276 mg, 0.65 mmol) in 1 mL DCM by the method of Dess & Martin.^32^ Yield – 238 mg (92%) of yellow oil. HRMS (*m*/*z*): [*M*+H]^+^ calcd for C_21_H_37_O_3_Si_2_, 393.2276; found, 393.2273.

(*E*)-3-(3,4-Bis­((*tert*-butyldimethylsilyl)­oxy)­phenyl)­acrylic acid (**16**) was obtained from 3-(3,4-dihydroxyphenyl)­acrylic acid (Merck KGaA, Darmstadt, Germany) as described by Barcelos et al.^75^

##### (*E*)-3-(3,4-Bis­((*tert*-butyldimethylsilyl)­oxy)­phenyl)-*N*-(3-(4-methoxyphenyloxy)­propyl)­acrylamide (**27**)

**General procedure 1**. To (*E*)-3-(3,4-bis­((*tert*-butyldimethylsilyl)­oxy)­phenyl)­acrylic acid (102 mg, 0.25 mmol) in 2 mL THF, *N,N,N′,N′*-Tetramethyl-*O-*(1*H*-benzotriazol-1-yl)­uronium hexafluorophosphate (HBTU, 144 mg, 0.38 mmol) and Et_3_N (101 mg, 1.00 mmol) were added. After the reaction mixture was stirred at rt for 15 min, 3-(4-methoxyphenyloxy)­propan-1-amine (69 mg, 0.38 mmol, BLD Pharm) was added. The reaction mixture was stirred at rt overnight and concentrated *in vacuo*. The product was isolated by flash chromatography using Biotage HP-Sfär Silica HC Duo 20 μm, 25 g; gradient 0 to 20% MeOH in DCM. Yield – 134 mg (94%) of yellowish oil. ^1^H NMR (400 MHz, CDCl_3_) δ 7.46 (d, *J* = 15.6 Hz, 1H, CH=), 6.99 (d, *J* = 7.5 Hz, 2H, H_Ar_), 6.85–6.77 (m, 5H, H_Ar_), 6.31–6.14 (m, 2H, CH=), 4.13–3.94 (m, 2H, CH_2_), 3.77–3.73 (s, 3H, OCH_3_), 3.61–3.40 (m, 2H, CH_2_), 2.16–2.01 (m, 2H, CH_2_), 0.98 (2 × s, 18H, CH_3_), 0.21 (2 × s, 12H, CH_3_). ESI-MS, positive mode: *m*/*z* (rel. int., %) = 572 (100) [M + H]^+^.

##### (*E*)-3-(3,4-Dihydroxyphenyl)-*N*-(3-(4-methoxyphenyloxy)­propyl)­acrylamide (**1**)

**General procedure 2**. The cleavage of TBDMS groups in compound 27 (134 mg, 0.23 μmol) was performed in MeOH (5 mL) using 53 mg (0.92 mmol) KF together with 127 mg (0.92 mmol) Et_3_N × HCl. The reaction mixture was stirred overnight at rt. After MeOH was evaporated, the title compound was isolated by preparative HPLC with gradient elution (B/A: 20/80 → 100/0). Yield – 67 mg (85%). ^1^H NMR (400 MHz, DMSO-*d*_6_) δ 8.05 (t, *J* = 5.6 Hz, 1H, NH), 7.23 (d, *J* = 15.7 Hz, 1H, CH=), 6.93 (d, *J* = 2.1 Hz, 1H, H_Ar_), 6.88–6.78 (m, 4H, H_Ar_), 6.73 (d, *J* = 8.1 Hz, 1H, H_Ar_), 6.31 (d, *J* = 15.7 Hz, 1H, CH=), 3.93 (t, *J* = 6.3 Hz, 2H, CH_2_), 3.69 (s, 3H, OCH_3_), 3.32–3.20 (m, 2H, CH_2_), 1.87 (p, *J* = 6.5 Hz, 2H, CH_2_). ^13^C NMR (101 MHz, DMSO-*d*_6_) δ 165.4, 153.3, 152.6, 147.2, 145.5, 139.0, 126.4, 120.3, 118.5, 115.7, 115.4, 114.6, 113.8, 65.7, 55.3, 37.9, 35.7, 29.1. ESI-MS, negative mode: *m*/*z* (rel. int., %) = 342 (100) [M-H]^−^. HRMS (*m*/*z*): [*M*+Na]^+^ calcd for C_19_H_21_N_2_NaO_5_, 366.1312; found, 366.1309.

(*E*)-3-(3,4-Bis­((*tert*-butyldimethylsilyl)­oxy)­phenyl)-*N*-(3-((4-methoxyphenyl)­thio)­propyl)­acrylamide (**28**) was obtained from 3-((4-methoxyphenyl)­thio)­propan-1-amine^14^ (29 mg, 0.15 mmol), *N,N,N′,N′*-tetramethyl-*O*-(1*H*-benzotriazol-1-yl)­uronium hexafluorophosphate (HBTU, 55 mg, 0.15 mmol) and (*E*)-3-(3,4-bis­((*tert*-butyldimethylsilyl)­oxy)­phenyl)­acrylic acid (40 mg, 0.10 mmol) in the presence of Et_3_N (40 mg, 0.39 mmol) using the general procedure 1. The title compound was isolated by flash chromatography using Biotage HP-Sfär Silica HC Duo cartridge 20 μm, 10 g SiO_2_ (gradient: methanol in DCM 2–20%). Yield – 22 mg (38%) of yellowish solid. ^1^H NMR (400 MHz, CDCl_3_) δ 7.48 (d, *J* = 15.6 Hz, 1H, CH=), 7.36 (d, *J* = 8.9 Hz, 1H, H_Ar_), 6.98 (d, *J* = 9.1 Hz, 1H, H_Ar_), 6.86 (s, 1H, H_Ar_), 6.80 (d, *J* = 8.7 Hz, 1H, H_Ar_), 6.17 (d, *J* = 15.6 Hz, 1H, CH=), 3.79 (s, 3H, OCH_3_), 3.48 (q, *J* = 6.6 Hz, 2H, CH_2_), 2.88 (t, *J* = 7.1 Hz, 2H, CH_2_), 1.91–1.79 (m, 2H, CH_2_), 0.99 (2 × s, 18H, CH_3_), 0.21 (2 × s, 12H, CH_3_). ^13^C NMR (101 MHz, CDCl_3_) δ 166.4, 159.2, 148.9, 147.2, 141.0, 133.5, 128.5, 126.1, 121.6, 121.2, 120.6, 118.4, 114.8, 55.4, 53.5, 38.7, 33.5, 29.2, 26.0, 26.0, 18.6, −3.9, −4.0. ESI-MS, positive mode: *m*/*z* (rel. int., %) = 588 (100) [M + H]^+^.

(*E*)-3-(3,4-Dihydroxyphenyl)-*N*-(3-((4-methoxyphenyl)­thio)­propyl)­acrylamide (**2**) was obtained from compound **28** (22 mg, 37 μmol) with 9 mg (0.15 mmol) KF and 21 mg (0.15 mmol) Et_3_N × HCl using the general procedure 2. The title compound was isolated by flash chromatography using Biotage HP-Sfär Silica HC Duo cartridge 20 μm, 10 g SiO_2_ (gradient: methanol in DCM 2–40%). Yield – 9 mg (67%) of colorless solid. HPLC: *t*_R_ = 6.1 min (B/A: 30/70–100/0 in 15 min, flow 1.2 mL/min, 254 nm). ^1^H NMR (400 MHz, CD_3_OD) δ 7.37–7.32 (m, 2H, H_Ar_), 7.00 (d, *J* = 2.1 Hz, 1H, H_Ar_), 6.93–6.83 (m, 3H, CH =, H_Ar_), 6.76 (d, *J* = 8.1 Hz, 1H, H_Ar_), 6.33 (d, *J* = 15.7 Hz, 1H, CH=), 3.76 (s, 3H, OCH_3_), 3.38 (t, *J* = 6.8 Hz, 2H, CH_2_), 2.86 (dd, *J* = 7.7, 6.7 Hz, 2H, CH_2_), 1.78 (p, *J* = 7.0 Hz, 2H, CH_2_). ^13^C NMR (101 MHz, CD_3_OD) δ 169.3, 160.6, 148.7, 146.7, 142.2, 134.5, 128.3, 127.4, 122.1, 118.3, 116.4, 115.6, 115.1, 55.8, 39.4, 34.0, 30.3. ESI-MS, positive mode: *m*/*z* (rel. int., %) = 382 (100) [M + Na]^+^. HRMS (*m*/*z*): [*M*+Na]^+^ calcd for C_19_H_21_NNaO_4_S, 382.1083; found, 382.1080.

##### Ethyl (*E*)-4-(7-Methoxy-2,3-dihydrobenzo­[1,4-*f*]­thiazepin-4­(5*H*)-yl)­but-2-enoate (**29**)

To a solution of 7-methoxy-2,3,4,5-tetrahydrobenzo­[1,4-*f*]­thiazepine (BLD Pharm, 50 mg, 0.26 mmol) in DCM under Ar, ethyl *trans*-4-bromo-2-butenoate (BLD Pharm, 48 mg, 0.38 mmol), and pyridine (39 mg, 0.52 mmol) were added. The reaction mixture was stirred overnight at room temperature. After the solvent was evaporated *in vacuo*, the product was isolated by flash column chromatography (Biotage HP-Sfär Silica HC Duo 20 μm 10 g; gradient 5 to 100% EtOAc in hexane). Yield – 21 mg (26%) of yellowish oil. HPLC: *t*_*R*_ = 11.1 min, B/A: 50/50 → 0/100 in 20 min, 254 nm. ^1^H NMR (400 MHz, CDCl_3_) δ 7.43 (d, *J* = 8.4 Hz, 1H, H-9), 6.91 (dt, *J* = 15.7, 5.9 Hz, 1H, CH=), 6.72 (d, *J* = 2.8 Hz, 1H, H-6), 6.68 (dd, *J* = 8.4, 2.8 Hz, 1H, H-8), 5.95 (dt, *J* = 15.7, 1.7 Hz, 1H, CH=), 4.19 (q, *J* = 7.1 Hz, 2H, CH_2_CH_3_), 4.08 (s, 2H, NCH_2_), 3.76 (s, 3H, OCH_3_), 3.34–3.26 (m, 2H, NCH_2_), 3.16 (dd, *J* = 6.0, 1.7 Hz, 2H, NCH_2_), 2.73–2.62 (m, 2H, SCH_2_), 1.28 (t, *J* = 7.1 Hz, 3H, CH_2_CH_3_). ^13^C NMR (101 MHz, CDCl_3_) δ 166.3 (CO), 159.2, 145.9, 144.5, 133.9, 127.8, 123.2, 116.7, 112.6, 60.4, 59.8, 58.6, 55.4, 53.5, 30.7, 14.3. ESI-MS, positive mode: *m*/*z* (rel. int., %) = 308 (100) [*M*+H]^+^. HRMS (*m*/*z*): [*M*+H]^+^ calcd for C_16_H_21_NO_3_S, 308.1315; found, 308.1316.

(*E*)-4-(7-Methoxy-2,3-dihydrobenzo­[1,4-*f*]­thiazepin-4­(5*H*)-yl)­but-2-enoic acid (**17**) was obtained by saponification of ethyl ester **29** (80 mg, 0.26 mmol). For that, ester **29** was dissolved in 3 mL EtOH and 72 mg (1.8 mmol) NaOH in 1.5 mL H2O was added at 0 °C. The reaction mixture was stirred at rt for 1 h. After pH was adjusted to **2** with 6 M HCl, the title product precipitated and was filtered. Yield – 68 mg (95%) of colorless solid. ^1^H NMR (400 MHz, DMSO-*d*_6_) δ 7.51 (d, *J* = 8.5 Hz, 1H, H-9), 7.23 (d, *J* = 2.9 Hz, 1H, H-6), 6.97 (dd, *J* = 8.5, 2.9 Hz, 1H, H-8), 6.81 (dt, *J* = 15.5, 6.9 Hz, 1H, CH=), 6.19 (d, *J* = 15.5 Hz, 1H, CH=), 4.60 (s, 2H, CH_2_), 3.96 (s, 2H, CH_2_), 3.78 (s, 3H, OCH_3_), 3.68–3.48 (m, 2H, CH_2_), 3.08 (s, 2H, CH_2_). ^13^C NMR (101 MHz, DMSO-*d*_6_) δ 166.0, 159.2, 158.4, 135.5, 133.9, 129.9, 127.9, 119.1, 115.1, 58.1, 56.0, 55.5, 30.5, 15.3. ESI-MS, positive mode: *m*/*z* (rel. int., %) = 280 (100) [*M*+H]^+^. HRMS (*m*/*z*): [*M*+H]^+^ calcd for C_14_H_17_NO_3_S, 280.1002; found, 280.1001.

##### *tert*-Butyl (*E*)-2-(4-(7-Methoxy-2,3-dihydrobenzo­[1,4-*f*]­thiazepin-4­(5*H*)-yl)­but-2-enoyl)­hydrazine-1-carboxylate (**30**)

To the solution of (*E*)-4-(7-methoxy-2,3-dihydrobenzo­[1,4-*f*]­thiazepin-4­(5*H*)-yl)­but-2-enoic acid **17** (272 mg, 1.0 mmol) in 10 mL DMSO, *tert*-butyl hydrazinecarboxylate (136 mg, 1.03 mmol) and 1-ethyl-3-(3-(dimethylamino)­propyl)­carbodiimide (197 mg, 1.27 mmol) were added, and the reaction mixture was stirred overnight at rt. After the solvent was removed *in vacuo*, 10 mL water and 10 mL DCM were added. The organic phase was separated, the aqueous solution extracted with DCM (2 × 5 mL). The combined organic solutions were dried over Na_2_SO_4_. The filtrate was evaporated, and the product isolated by flash chromatography using Biotage SNAP Ultra 10g cartridge (gradient: methanol in DCM 2% → 20%). Yield – 94 mg (24%) of yellowish oil. ^1^H NMR (400 MHz, CDCl_3_) δ 7.43 (d, *J* = 8.3 Hz, 1H), 6.89 (dt, *J* = 15.5, 5.7 Hz, 1H, CH=), 6.74 (d, *J* = 2.8 Hz, 1H), 6.68 (dd, *J* = 8.4, 2.8 Hz, 1H), 6.04 (dt, *J* = 15.6, 1.7 Hz, 1H, CH=), 4.09 (s, 2H), 3.77 (s, 3H, OCH_3_), 3.35–3.25 (m, 2H, CH_2_), 3.16 (dd, *J* = 5.8, 1.7 Hz, 2H, CH_2_), 2.70–2.62 (m, 2H, CH_2_), 1.45 (s, 9H, CH_3_). ^13^C NMR (101 MHz, CDCl_3_) δ 164.9, 159.3, 155.7, 144.1, 143.1, 133.9, 127.8, 122.7, 116.9, 112.7, 82.0, 65.4, 59.8, 58.3, 55.5, 53.6, 53.4, 30.5, 28.3, 28.3. ESI-MS, positive mode: *m*/*z* (rel. int., %) = 394 (100) [*M*+H]^+^.

(*E*)-4-(7-Methoxy-2,3-dihydrobenzo­[1,4-*f*]­thiazepin-4­(5*H*)-yl)­but-2-enoyl-hydrazine (**18**) was obtained from compound **30** (60 mg, 0.15 mmol) in 10% TFA in DCM (2 mL). The reaction mixture was stirred overnight at rt. After the solvent was removed *in vacuo*, the product was isolated by preparative HPLC with gradient elution (B/A: 20/80 → 100/0). Yield – 43 mg (98%) of yellowish oil. ^1^H NMR (400 MHz, CD_3_OD) δ 7.61–7.52 (m, 1H, H_Ar_), 7.19–7.12 (m, 1H, H_Ar_), 7.03–6.97 (m, 1H, H_Ar_), 6.97–6.88 (m, 1H, CH=), 6.42 (d, *J* = 15.5 Hz, 1H, CH=), 4.90 (s, 9H, NH, H_2_O), 4.70 (s, 2H, NCH_2_), 4.07 (d, *J* = 7.1 Hz, 2H, NCH_2_), 3.84 (s, 3H, CH_3_), 3.74 (s, 2H, OCH_2_), 3.09 (t, *J* = 5.2 Hz, 2H, CH_2_). ^13^C NMR (101 MHz, CD_3_OD) δ 161.6 (CO), 136.4 (C), 135.6 (C), 135.5 (CH), 133.3 (CH=), 129.5 (CH=), 120.3 (C), 119.9 (CH), 116.6 (CH), 60.3 (CH_2_), 58.7 (CH_2_), 56.2 (OCH_3_), 56.1 (CH_2_), 30.6 (CH_2_). ESI-MS, positive mode: *m*/*z* (rel. int., %) = 294 (100) [*M*+H]^+^. HRMS (*m*/*z*): [*M*+H]^+^ calcd for C_14_H_19_N_3_O_2_S, 294.1271; found, 294.1269.

(*E*)-*N*-((*E*)-3-(3,4-Bis­((*tert*-butyldimethylsilyl)­oxy)­phenyl)­acryloyl)-*N′*-((*E*)-4-(7-methoxy-2,3-dihydrobenzo­[1,4-*f*]­thiazepin-4­(5*H*)-yl)­but-2-enoyl)­hydrazine (**31**) was obtained from compound **18** (15 mg, 0.05 mmol), (*E*)-3-(3,4-bis­((*tert*-butyldimethylsilyl)­oxy)­phenyl)­acrylic acid (25 mg, 0.06 mmol) using the general procedure 1. The product was isolated by flash chromatography using the cartridge Biotage HP-Sfär Silica HC Duo 20 μm, 10 g; gradient 0 to 20% MeOH in DCM. Yield – 24 mg (70%) of colorless solid. ^1^H NMR (400 MHz, CDCl_3_) δ 7.57 (d, *J* = 15.5 Hz, 1H, CH=), 7.42 (d, *J* = 8.4 Hz, 1H, H_Ar_), 7.02–6.90 (m, 3H, CH =, H_Ar_), 6.82–6.74 (m, 2H, H_Ar_), 6.69 (dd, *J* = 8.5, 2.8 Hz, 1H, H_Ar_), 6.43 (d, *J* = 15.5 Hz, 1H, CH=), 6.25 (d, *J* = 15.9 Hz, 1H, CH=), 4.17 (s, 2H, CH_2_), 3.75 (s, 3H, OCH_3_), 3.37–3.26 (m, 2H, CH_2_), 2.75–2.67 (m, 2H, CH_2_), 1.02–0.94 (m, 18H, CH_3_), 0.25–0.13 (m, 12H, CH_3_). ESI-MS, positive mode: *m*/*z* (rel. int., %) = 684 (100) [M + H]^+^. HRMS (*m*/*z*): [*M*+H]^+^ calcd for C_35_H_54_N_3_O_5_SSi_2_, 684.3317; found, 684.3319.

(*E*)-*N′*-((*E*)-3-(3,4-Dihydroxyphenyl)­acryloyl)-4-(7-methoxy-2,3-dihydrobenzo­[1,4-*f*]­thiazepin-4­(5*H*)-yl)­but-2-enehydrazide (**3**) was obtained from compound **31** (24 mg, 35 μmol) with 8 mg (0.14 mmol) KF and 19 mg (0.14 mmol) Et_3_N × HCl using the general procedure 2. The title compound was isolated by flash chromatography using Biotage HP-Sfär Silica HC Duo cartridge 20 μm, 10 g (gradient: methanol in DCM 2–40%). Yield – 12 mg (75%) of colorless solid. ^1^H NMR (400 MHz, DMSO-*d*_6_) δ 10.23–10.04 (m, 2H, NH), 7.41 (d, *J* = 8.4 Hz, 1H, H_Ar_), 7.33 (d, *J* = 15.7 Hz, 1H, CH=), 6.98 (d, *J* = 2.0 Hz, 1H, H_Ar_), 6.90–6.84 (m, 2H, H_Ar_), 6.78–6.73 (m, 2H, H_Ar_), 6.66 (dt, *J* = 15.6, 5.8 Hz, 1H, CH=), 6.41 (d, *J* = 15.7 Hz, 1H, CH=), 6.14 (d, *J* = 15.6 Hz, 1H, CH=), 4.02 (s, 2H, CH_2_), 3.75 (s, 3H, OCH_3_), 3.23–3.18 (m, 2H, CH_2_), 3.17–3.12 (m, 2H, CH_2_), 2.75–2.65 (m, 2H, CH_2_). ^13^C NMR (101 MHz, CD_3_OD) δ 167.6, 161.6, 149.4, 146.9, 144.1, 135.5, 132.5, 131.5, 129.4, 127.8, 122.4, 119.7, 116.6, 116.4, 115.1, 114.9, 56.1, 29.7, 29.3, 20.5. ESI-MS, positive mode: *m*/*z* (rel. int., %) = 456 (100) [M + H]^+^. HRMS (*m*/*z*): [*M*+H]^+^ calcd for C_23_H_26_N_3_O_5_S, 456.1588; found, 456.1592.

4-(7-Methoxy-2,3-dihydrobenzo­[1,4-*f*]­thiazepin-4­(5*H*)-yl)­butan-1-amine (**19**) was obtained in two steps from 7-methoxy-2,3,4,5-tetrahydrobenzo­[1,4-*f*]­thiazepine (BLD Pharm, Kaiserslautern, Germany) as described in our previous work.^14^

2-((4-(7-Methoxy-2,3-dihydrobenzo­[1,4-*f*]­thiazepin-4­(5*H*)-yl)­butyl)­amino)-2-oxoacetic acid (**20**) was obtained from amine **19** (300 mg, 1.13 mmol) and methyl 2-chloro-2-oxoacetate (136 mg, 1.12 mmol) using the two-step procedure. First, to the compound 19 in DCM (5 mL), pyridine (54 μL) was added; then the reaction mixture was cooled to 0 °C. After that, methyl 2-chloro-2-oxoacetate (221 mg, 1.8 mmol, abcr GmbH) was added dropwise. The reaction mixture was stirred for 1 h at 0 °C. After warming up to room temperature, the reaction mixture was washed with sat. aq. NaHCO_3_, 1 M aq. HCl and water. The organic layer was separated and dried over Na_2_SO_4_. The solvent was evaporated under reduced pressure on a rotary evaporator. The title compound was isolated by means of flash chromatography (cartridge Biotage HP-Sfär Silica HC Duo 20 μm 10 g, gradient 5 to 50% MeOH in DCM) to afford methyl 2-((4-(7-methoxy-2,3-dihydrobenzo­[1,4-*f*]­thiazepin-4­(5*H*)-yl)­butyl)­amino)-2-oxoacetate as a yellowish solid (156 mg, 38% yield). The product was then dissolved in MeOH (2 mL), and 72 mg (1.8 mmol) NaOH in 1.5 mL H_2_O was added at 0 °C. The reaction mixture was stirred at rt for 1 h. After pH was adjusted to 2 with 6 M HCl, the product was extracted with DCM. The organic layer was dried over Na_2_SO_4_ and the solvent evaporated giving 104 mg (71%) of the title compound as a beige solid. ^1^H NMR (400 MHz, CD_3_OD) δ 7.56 (d, *J* = 8.5 Hz, 1H), 7.21 (d, *J* = 2.8 Hz, 1H), 6.98 (dd, *J* = 8.5, 2.7 Hz, 1H), 4.68 (s, 2H), 3.84 (s, 3H), 3.80–3.62 (m, 3H), 3.38–3.31 (m, 2H), 3.09 (d, *J* = 73.5 Hz, 0H), 1.85 (s, 2H), 1.71–1.58 (m, 2H). ^13^C NMR (101 MHz, CD_3_OD) δ 162.6, 161.7, 160.5, 136.6, 135.5, 129.5, 119.8, 116.6, 60.1, 56.2, 53.8, 49.4, 39.6, 27.2, 22.5. ESI-MS, positive mode: *m*/*z* (rel. int., %) = 339 (100) [*M*+H]^+^. HRMS (*m*/*z*): [*M*+H]^+^ calcd for M = C_16_H_22_N_2_O_4_S, 339.1373; found, 339.1378.

##### *N-*(6-*tert*-Butiloxycarbonylamino)­hexyl-*N′*-(4-(7-methoxy-2,3-dihydrobenzo­[1,4-*f*]­thiazepin-4­(5*H*)-yl)­butyl) Oxalic Acid Diamide (**32**)

*N*-(*tert*-Butoxycarbonyl)-1,6-hexanediamine (31 mg, 0.11 mmol), *O*-(7-azabenzotriazol-1-yl)-*N,N,N′,N′*-tetramethyluronium hexafluorphosphat (HATU, 65 mg, 0.17 mmol) and Et_3_N (22 mg, 0.22 mmol) were added to a stirred solution of **20** (100 mg, 0.11 mmol) in DMSO (0.4 mL). The reaction mixture was stirred for 1 h at rt. After the solvent was removed *in vacuo*, the title compound was isolated by means of flash chromatography (cartridge Biotage HP-Sfär Silica HC Duo 20 μm 20 g, gradient 5 to 20% MeOH in DCM). Yield – 33 mg (41%) of white solid. ^1^H NMR (400 MHz, CDCl_3_) δ 7.81 (t, *J* = 4.9 Hz, 1H, NH), 7.53 (t, *J* = 6.1 Hz, 1H, NH), 7.45 (d, *J* = 8.4 Hz, 1H, H_Ar_), 6.89 (s, 1H, H_Ar_), 6.75 (d, *J* = 8.4 Hz, 1H, H_Ar_), 4.58 (s, 1H, NH), 4.28 (s, 2H, CH_2_), 3.79 (s, 3H, OCH_3_), 3.48–3.43 (m, 2H, CH_2_), 3.33–3.24 (m, 2H, CH_2_), 3.20 (q, *J* = 7.3 Hz, 2H, CH_2_), 3.12–3.04 (m, 4H, CH_2_), 2.83–2.78 (m, 2H, CH_2_), 2.63 (br. s, 2H, CH_2_), 1.65–1.52 (m, 4H, CH_2_), 1.43 (s, 13H, CH_2_, CH_3_), 1.38–1.33 (m, 4H, CH_2_). ESI-MS, positive mode: *m*/*z* (rel. int., %) = 537 (100) [*M*+H]^+^. HRMS (*m*/*z*): [*M*+H]^+^ calcd for C_27_H_45_N_4_O_5_S, 537.3105; found, 537.3104.

##### *N*-(6-Aminohexyl)-*N′*-(4-(7-methoxy-2,3-dihydrobenzo­[1,4-*f*]­thiazepin-4­(5*H*)-yl)­butyl)­oxalamide (**21**)

The *t*-butoxycarbamate deprotection of **32** (33 mg, 0.06 mmol) was performed in 10% TFA in DCM (2 mL). After the reaction mixture was stirred for 3 h at rt, the solvents were removed *in vacuo*. The compound was used in the next reaction without further purification. Yield 26 mg (99%) of yellowish oil. ^1^H NMR (400 MHz, CD_3_OD) δ 7.55 (d, *J* = 8.5 Hz, 1H, H_Ar_), 7.19 (d, *J* = 2.8 Hz, 1H, H_Ar_), 6.98 (dd, *J* = 8.5, 2.8 Hz, 1H, H_Ar_), 4.67 (s, 2H, CH_2_), 3.84 (s, 3H, OCH_3_), 3.28–3.15 (m, 6H, CH_2_), 3.00–2.86 (m, 4H, CH_2_), 1.72–1.53 (m, 6H, CH_2_), 1.47–1.34 (m, 4H, CH_2_), 1.34–1.22 (m, 4H, CH_2_). ^13^C NMR (101 MHz, CD_3_OD) δ 161.9, 161.6, 161.5, 161.4, 161.2, 135.3, 119.6, 118.8, 116.4, 56.0, 47.7, 40.5, 40.4, 40.1, 39.3, 30.8, 29.8, 28.2, 27.1, 26.8, 24.0, 22.3, 9.0. ESI-MS, positive mode: *m*/*z* (rel. int., %) = 437 (100) [M + H]^+^. HRMS (*m*/*z*): [*M*+H]^+^ calcd for C_22_H_36_N_4_O_3_S, 437.2581; found, 437.2588.

Compound **33** was obtained from compound **21** (27 mg, 0.06 mmol), TBDMS protected caffeic acid (16) (16 mg, 0.04 mmol) using the general procedure 1. The product was isolated by flash chromatography using the cartridge Biotage HP-Sfär Silica HC Duo 20 μm, 10 g SiO_2_; gradient 0 to 20% MeOH in DCM. Yield – 25 mg (76%) of yellowish solid. ^1^H NMR (400 MHz, CDCl_3_) δ 7.45–7.39 (m, 1H, CH =, H_Ar_), 7.08 (d, *J* = 2.2 Hz, 1H, H_Ar_), 6.99–6.92 (m, 2H, H_Ar_), 6.83 (dd, *J* = 8.7, 2.2 Hz, 1H, H_Ar_), 6.77 (d, *J* = 8.7 Hz, 1H, H_Ar_), 6.38 (d, *J* = 15.6 Hz, 1H, CH=), 4.06 (s, 1H), 3.77 (s, 1H), 3.30 (s, 0H), 3.24–3.15 (m, 2H), 3.10 (s, 2H), 2.81 (s, 4H), 1.62 (s, 0H), 1.51 (s, 0H), 1.34 (t, *J* = 7.3 Hz, 5H), 0.96 (d, *J* = 2.5 Hz, 6H), 0.18 (d, *J* = 7.4 Hz, 3H). ESI-MS, positive mode: *m*/*z* (rel. int., %) = 827 (100) [M + H]^+^.

Compound **4** was obtained from compound **33** (25 mg, 30 μmol), 7 mg (0.12 mmol) KF and 17 mg (0.12 mmol) Et_3_N × HCl using the general procedure 2. Compound 4 was isolated by preparative HPLC with gradient elution (B/A: 20/80 → 100/0). Yield – 4 mg (22%) of white solid. ^1^H NMR (400 MHz, CD_3_OD) δ 7.55 (d, *J* = 8.5 Hz, 1H, H_Ar_), 7.37 (d, *J* = 15.7 Hz, 1H, CH=), 7.20 (d, *J* = 2.8 Hz, 1H, H_Ar_), 7.02–6.94 (m, 2H, H_Ar_), 6.90 (d, *J* = 8.5 Hz, 1H, H_Ar_), 6.76 (d, *J* = 8.2 Hz, 1H, H_Ar_), 6.35 (d, *J* = 15.7 Hz, 1H, CH=), 4.68 (s, 2H, CH_2_), 3.83 (s, 2H, CH_2_), 3.35 (s, 3H, OCH_3_), 3.29–3.23 (m, 4H, CH_2_), 3.23–3.13 (m, 2H, CH_2_), 3.00–2.90 (m, 2H, CH_2_), 1.90–1.73 (m, 2H, CH_2_), 1.68–1.52 (m, 6H, CH_2_), 1.44–1.27 (m, 6H, CH_2_). ESI-MS, positive mode: *m*/*z* (rel. int., %) = 599 (100) [M + H]^+^. HRMS (*m*/*z*): [*M*+H]^+^ calcd for C_31_H_43_N_4_O_6_S, 599.2898; found, 599.2892.

##### Compound **34**

The title compound was obtained from amine **19** (39 mg, 0.15 mmol), (*E*)-3-(3,4-bis­((*tert*-butyldimethylsilyl)­oxy)­phenyl)­acrylic acid (40 mg, 0.10 mmol), HBTU (57 mg, 0.15 mmol) and Et_3_N (30 mg, 0.3 mmol) using the general procedure 1. The product was isolated by flash chromatography using Biotage HP-Sfär Silica HC Duo 20 μm, 10 g; gradient 2 to 20% MeOH in DCM. Yield – 57 mg (88%) of yellow oil. ^1^H NMR (400 MHz, CDCl_3_) δ 7.46 (d, *J* = 8.6 Hz, 1H, H_Ar_), 7.13–6.97 (m, 2H, H_Ar_), 6.97–6.91 (m, 2H, H_Ar_), 6.85–6.75 (m, 2H, CH =, H_Ar_), 6.29 (d, *J* = 15.6 Hz, 1H, CH=), 4.65–4.51 (m, 1H, CH_2_), 3.85–3.74 (m, 2H, CH_2_), 3.66 (s, 3H, OCH_3_), 3.47–3.27 (m, 4H, CH_2_), 3.14–3.00 (m, 2H, CH_2_), 1.97 (br. s, 1H, CH_2_), 1.85–1.74 (m, 1H, CH_2_), 1.73–1.60 (m, 2H, CH_2_), 1.08–0.94 (m, 18H, CH_3_), 0.21 (s, 12H, CH_3_). ^13^C NMR (101 MHz, CDCl_3_) δ 168.1, 165.6, 160.1, 149.1, 147.1, 141.5, 134.3, 127.9, 127.6, 122.0, 121.1, 120.5, 117.5, 116.1, 55.5, 47.5, 38.6, 25.9, 25.6, 18.5, 18.4, 8.7, −3.6, −4.1, −4.2. ESI-MS, positive mode: *m*/*z* (rel. int., %) = 657 (100) [M + H]^+^.

Compound **5** was obtained from compound **34** (55 mg, 84 μmol), 20 mg (0.34 mmol) KF and 46 mg (0.12 mmol) Et_3_N × HCl using the general procedure 2. The title compound was isolated by flash chromatography using cartridge Biotage HP-Sfär Silica HC Duo 20 μm, 10 g SiO_2_; gradient 2 to 40% MeOH in DCM. Yield – 22 mg (61%) of yellowish solid. ^1^H NMR (400 MHz, DMSO-*d*_6_) 7.99–7.92 (m, 1H, NH), 7.39 (d, *J* = 8.4 Hz, 1H, H_Ar_), 7.21 (d, *J* = 15.7 Hz, 1H, CH=), 6.93 (d, *J* = 2.0 Hz, 2H, H_Ar_), 6.82 (dd, *J* = 8.2, 2.0 Hz, 1H, H_Ar_), 6.74 (d, *J* = 8.1 Hz, 2H, H_Ar_), 6.31 (d, *J* = 15.7 Hz, 1H, CH=), 4.12–4.06 (br. s, 1H, NH), 4.05–3.99 (br. s, 2H, CH_2_), 3.72 (s, 3H, OCH_3_), 3.33 (s, 4H, CH_2_), 3.19–3.11 (m, 4H, CH_2_), 2.67 (s, 2H, CH_2_), 1.51–1.36 (m, 2H, CH_2_). ESI-MS, positive mode: *m*/*z* (rel. int., %) = 429 (100) [M + H]^+^. HRMS (*m*/*z*): [*M*+H]^+^ calcd for C_23_H_29_N_2_O_4_S, 429.1843; found, 429.1838.

##### Compound **35**

The reductive amination was performed in nonacidic conditions according to Salvage et al.^28^ Aldehyde **15** (100 mg, 0.31 mmol), amine **19** (87 mg, 0.38 mmol), *N*,*N*-diisopropylethylamine (120 mg, 0.93 mmol) were dissolved in 6 mL THF. Then MgSO_4_ (74 mg, 0.62 mmol) was added and the obtained suspension was stirred for 1 h at rt. Sodium triacetoxyborohydride (131 mg, 0.62 mmol) was added and the reaction mixture was stirred for 24 h at rt. The suspension was filtered, and the filtrate was concentrated *in vacuo* to give a yellow oil. The oil was dissolved in DCM (10 mL), the organic solution was washed with sat. aq. NaHCO_3_ (2 × 5 mL) and dried over Na_2_SO_4_. The title compound was isolated by means of flash chromatography (cartridge Biotage HP-Sfär Silica HC Duo 20 μm 20 g SiO_2_, gradient 2 to 20% MeOH in DCM). Yield – 29 mg (15%) of yellow oil. ^1^H NMR (400 MHz, CDCl_3_) δ 7.45 (d, *J* = 8.4 Hz, 1H, H_Ar_), 6.89 (d, *J* = 2.1 Hz, 1H, H_Ar_), 6.86–6.79 (m, 2H, H_Ar_), 6.77 (d, *J* = 8.2 Hz, 1H, H_Ar_), 6.69 (dd, *J* = 8.4, 2.8 Hz, 1H, H_Ar_), 6.40 (d, *J* = 15.8 Hz, 1H, CH), 6.10 (dt, *J* = 15.8, 6.7 Hz, 1H, CH), 4.11 (s, 2H, NCH_2_), 3.79 (s, 3H, OCH_3_), 3.35–3.23 (m, 4H, CH_2_), 2.69 (d, *J* = 7.7 Hz, 2H, CH_2_), 2.56–2.45 (m, 1H, NH), 2.39–2.31 (m, 2H, CH_2_), 1.56–1.41 (m, 2H, CH_2_), 1.29 (m, 4H), 1.00 (2 × s, *J* = 4.6 Hz, 18H, CH_3_), 0.21 (d, *J* = 1.9 Hz, 12H, CH_3_). ESI-MS, positive mode: *m*/*z* (rel. int., %) = 643 (100) [M + H]^+^. HRMS (*m*/*z*): [*M*+H]^+^ calcd for C_35_H_59_N_2_O_3_SSi_2_, 643.3779; found, 643.3789.

6-(7-Methoxy-2,3-dihydrobenzo­[1,4-*f*]­thiazepin-4­(5*H*)-yl)­hexane-1-amine (**22**) was obtained from 7-methoxy-2,3,4,5-tetrahydrobenzo­[1,4-*f*]­thiazepine (BLD Pharm, 500 mg, 2.5 mmol) and *N*-(6-bromohexyl)­phthalimide (713 mg, 2.3 mmol) using the two-step-procedure as described previously for 4-(7-methoxy-2,3-dihydrobenzo­[1,4-*f*]­thiazepin-4­(5*H*)-yl)­butan-1-amine.^14^ ^1^H NMR (400 MHz, CDCl_3_) δ 7.44 (d, *J* = 8.4 Hz, 1H, H_Ar_), 6.79 (d, *J* = 2.8 Hz, 1H, H_Ar_), 6.68 (dd, *J* = 8.4, 2.8 Hz, 1H, H_Ar_), 4.09 (s, 2H, CH_2_), 3.79 (s, 3H, OCH_3_), 3.40–3.27 (m, 2H, CH_2_), 2.68 (dd, *J* = 8.7, 5.5 Hz, 4H, CH_2_), 2.41–2.31 (m, 2H, CH_2_), 2.07 (s, 2H, NH), 1.55–1.37 (m, 4H, CH_2_), 1.37–1.18 (m, 4H, CH_2_). ^13^C NMR (101 MHz, CDCl_3_) δ 159.0, 159.0, 145.0, 133.5, 127.7, 116.9, 111.9, 59.6, 58.4, 55.3, 55.3, 52.0, 51.9, 41.9, 33.2, 30.2, 27.4, 27.3, 27.1, 27.0, 26.8. ESI-MS, positive mode: *m*/*z* (rel. int., %) = 295 (100) [M + H]^+^. HRMS (*m*/*z*): [*M*+H]^+^ calcd for C_16_H_27_N_2_OS, 295.1839; found, 295.1839.

8-(7-Methoxy-2,3-dihydrobenzo­[1,4-*f*]­thiazepin-4­(5*H*)-yl)­octan-1-amine (**23**) was obtained from 7-methoxy-2,3,4,5-tetrahydrobenzo­[1,4-*f*]­thiazepine (BLD Pharm, 500 mg, 2.5 mmol) and *N*-(8-Bromooctyl)­phthalimide (787 mg, 2.3 mmol) using the two-step-procedure as we described previously for 4-(7-methoxy-2,3-dihydrobenzo­[1,4-*f*]­thiazepin-4­(5*H*)-yl)­butan-1-amine.^14^ ^1^H NMR (400 MHz, CDCl_3_) δ 7.43 (d, *J* = 8.4 Hz, 1H, H_Ar_), 6.79 (d, *J* = 2.8 Hz, 1H, H_Ar_), 6.68 (dd, *J* = 8.4, 2.8 Hz, 1H, H_Ar_), 4.09 (s, 2H, CH_2_), 3.78 (s, 3H, OCH_3_), 3.34–3.29 (m, 2H, CH_2_), 2.69 (t, *J* = 7.1 Hz, 4H, CH_2_), 2.37–2.31 (m, 2H, CH_2_), 2.31–2.11 (m, 2H, NH_2_), 1.46 (dp, *J* = 14.4, 7.6 Hz, 4H, CH_2_), 1.35–1.21 (m, 8H, CH_2_). ^13^C NMR (101 MHz, CDCl_3_) δ 159.2, 145.1, 133.6, 127.9, 117.0, 112.0, 59.7, 58.5, 55.4, 52.2, 42.1, 33.4, 30.3, 29.6, 29.6, 29.5, 27.6, 27.4, 26.9. ESI-MS, positive mode: *m*/*z* (rel. int., %) = 323 (100) [M + H]^+^. HRMS (*m*/*z*): [*M*+H]^+^ calcd for C_18_H_31_N_2_OS, 323.2152; found, 323.2155.

##### 2-((2-Bromo-5-methoxybenzyl)­amino)­ethan-1-ol (**36**)

To a solution of 2-aminoethanol (1.77 g, 29 mmol) in 36 mL THF 1-bromo-2-(bromomethyl)-4-methoxybenzene (BLD Pharm, 3.32 g, 12 mmol) and sodium bicarbonate (2.44 g, 29 mmol) were added. The reaction mixture was stirred overnight at room temperature. After the solvent was removed *in vacuo*, 10 mL water and 10 mL DCM were added. The organic solution was separated, the aqueous solution extracted with CHCl_3_ (2 × 5 mL). The combined organic solutions were washed with brine and dried over Na_2_SO_4_. The filtrate was evaporated, and the product isolated by flash chromatography using the cartridge Biotage HP-Sfär Silica HC Duo 20 μm, 10 g SiO_2_; gradient 5 to 50% methanol in DCM. Yield – 2.11 g (68%) of yellow solid. ^1^H NMR (400 MHz, CDCl_3_) δ 7.42 (d, *J* = 8.7 Hz, 1H, H_Ar_), 6.96 (d, *J* = 3.0 Hz, 1H, H_Ar_), 6.69 (dd, *J* = 8.7, 3.0 Hz, 1H, H_Ar_), 3.84 (s, 2H, NCH_2_), 3.79 (s, 3H, OCH_3_), 3.69–3.64 (m, 2H, CH_2_), 2.82–2.76 (m, 2H, CH_2_), 2.55 (s, 2H, CH_2_).

##### 7-Methoxy-2,3,4,5-tetrahydrobenzo­[1,4-*f*]­oxazepane (**37**)

To the solution of 2-((2-bromo-5-methoxybenzyl)­amino)­ethan-1-ol (500 mg, 1.9 mmol) in *i*PrOH (7 mL), copper­(I) iodide (72 mg, 0.38 mmol) and potassium carbonate (524 mg, 3.8 mmol) were added. The reaction mixture was degassed and refluxed for 16 h. Then it was cooled down to room temperature, and the solid was removed by filtration. The filtrate was evaporated in vacuo and dissolved in DCM. The solution was washed with 1 M HCl, water and brine and dried over Na_2_SO_4_. The filtrate was evaporated, and the product isolated by flash column chromatography using Biotage SNAP Ultra 25 g SiO_2_ cartridge (gradient: methanol in DCM 2% → 20%). Yield – 184 mg (54%) of yellowish solid. ^1^H NMR (400 MHz, CDCl_3_) δ 6.90 (dd, *J* = 8.7, 0.3 Hz, 1H, H_Ar_), 6.75 (d, *J* = 3.0 Hz, 1H, H_Ar_), 6.74–6.66 (m, 1H, H_Ar_), 4.85 (s, 2H, CH_2_), 4.00–3.92 (m, 2H, CH_2_), 3.89–3.79 (br. s., 1H, NH), 3.74 (s, 3H, OCH_3_), 3.12 (s, 2H, CH_2_). ESI-MS, positive mode: *m*/*z* (rel. int., %) = 180 (100) [M + H]^+^. HRMS (*m*/*z*): [*M*+H]^+^ calcd for C_10_H_14_NO_2_, 180.1019; found, 180.1022.

4-(7-Methoxy-2,3-dihydrobenzo­[1,4-*f*]­oxazepin-4­(5*H*)-yl)­butan-1-amine (**24**) was obtained from 7-methoxy-2,3,4,5-tetrahydrobenzo­[1,4-*f*]­oxazepine (567 mg, 3.2 mmol) and *N*-(4-bromobutyl)­phthalimide (BLD Pharm, 169 mg, 0.6 mmol) using the two-step-procedure as described previously for 4-(7-methoxy-2,3-dihydrobenzo­[1,4-*f*]­thiazepin-4­(5*H*)-yl)­butan-1-amine.^14^ Overall yield–126 mg (85%). ^1^H NMR (400 MHz, CD_3_OD) δ 6.89 (d, *J* = 8.6 Hz, 3H), 6.78 (s, 2H), 6.73 (d, *J* = 11.7 Hz, 2H), 3.99 (d, *J* = 9.0 Hz, 3H), 3.78 (s, 6H), 3.75 (s, 8H), 3.04 (d, *J* = 8.8 Hz, 5H), 2.64 (s, 3H), 2.50 (d, *J* = 15.4 Hz, 3H), 1.58 (d, *J* = 18.6 Hz, 2H), 1.46 (d, *J* = 20.9 Hz, 1H). ^13^C NMR (101 MHz, CD_3_OD) δ 155.6, 153.6, 131.9, 120.8, 115.8, 112.8, 69.5, 58.2, 57.6, 54.6, 54.0, 41.0, 30.3, 24.0. ESI-MS, positive mode: *m*/*z* (rel. int., %) = 251 (100) [M + H]^+^. HRMS (*m*/*z*): [*M*+H]^+^ calcd for C_14_H_23_N_2_O_2_, 251.1754; found, 251.1757.

6-(7-Methoxy-2,3-dihydrobenzo­[1,4-*f*]­oxazepin-4­(5*H*)-yl)­hexan-1-amine (**25**) was obtained from 7-methoxy-2,3,4,5-tetrahydrobenzo­[1,4-*f*]­oxazepine (467 mg, 2.6 mmol) and *N*-(6-bromohexyl)­phthalimide (BLD Pharm, 162 mg, 0.52 mmol) using the two-step-procedure as described previously for 4-(7-methoxy-2,3-dihydrobenzo­[1,4-*f*]­thiazepin-4­(5*H*)-yl)­butan-1-amine.^14^ Overall yield–123 mg (76%). ^1^H NMR (400 MHz, CD_3_OD) δ 6.89 (d, *J* = 8.6 Hz, 1H, H_Ar_), 6.79–6.71 (m, 2H, H_Ar_), 4.00–3.97 (m, 2H, CH_2_), 3.78 (s, 2H, CH_2_), 3.75 (s, 3H, OCH_3_), 3.04 (d, *J* = 8.8 Hz, 2H, CH_2_), 2.63 (br. s, 2H, NH_2_), 2.53–2.44 (m, 2H, CH_2_), 1.62–1.52 (m, 2H, CH_2_), 1.51–1.42 (m, 2H, CH_2_), 1.42–1.23 (m, 4H, CH_2_). ^13^C NMR (101 MHz, CD_3_OD) δ 157.0, 155.1, 133.3, 122.3, 117.2, 114.2, 70.8, 59.6, 59.1, 58.3, 56.0, 55.5, 42.4, 28.4, 27.9, 27.9, 18.4.

8-(7-Methoxy-2,3-dihydrobenzo­[1,4-*f*]­oxazepin-4­(5*H*)-yl)­octan-1-amine (**26**) was obtained from 7-methoxy-2,3,4,5-tetrahydrobenzo­[1,4-*f*]­oxazepine (125 mg, 0.7 mmol) and *N*-(8-bromooctyl)­phthalimide (BLD Pharm, 236 mg, 0.7 mmol) using the two-step-procedure described previously for 4-(7-methoxy-2,3-dihydrobenzo­[1,4-*f*]­thiazepin-4­(5*H*)-yl)­butan-1-amine.^14^ Overall yield–58 mg (26%). ^1^H NMR (400 MHz, CDCl_3_) δ 6.93–6.86 (m, 1H, H_Ar_), 6.71–6.61 (m, 2H, H_Ar_), 4.02–3.94 (m, 2H, NCH_2_), 3.76 (s, 2H, NCH_2_), 3.74 (s, 1H, OCH_3_), 3.07–2.98 (m, 2H, CH_2_), 2.71–2.63 (m, 2H, CH_2_), 2.48–2.34 (m, 2H, CH_2_), 2.11 (s, 2H, NH_2_), 1.54–1.37 (m, 4H, CH_2_), 1.34–1.20 (m, 8H, CH_2_). ^13^C NMR (101 MHz, CDCl_3_) δ 155.4, 153.7, 133.0, 121.3, 116.1, 112.8, 70.1, 58.5, 58.4, 55.7, 54.1, 42.1, 33.4, 29.6, 29.5, 27.6, 27.4, 26.9. HRMS (*m*/*z*): [*M*+H]^+^ calcd for C_18_H_31_N_2_O_2_, 307.2380; found, 207.2381.

Compound **38** was obtained from amine **22** (39 mg, 0.15 mmol), (*E*)-3-(3,4-bis­((*tert*-butyldimethylsilyl)­oxy)­phenyl)­acrylic acid (40 mg, 0.10 mmol), HBTU (57 mg, 0.15 mmol) and Et_3_N (30 mg, 0.3 mmol) using the general procedure 1. The product was isolated by flash chromatography using the cartridge Biotage HP-Sfär Silica HC Duo 20 μm, 10 g SiO_2_; gradient 2 to 20% MeOH in DCM. Yield – 57 mg (88%) of yellow oil. ^1^H NMR (400 MHz, CD_3_OD) δ 7.42–7.36 (m, 2H, CH= H_Ar_), 7.05 (d, *J* = 7.7 Hz, 2H, H_Ar_), 6.92 (d, *J* = 2.8 Hz, 1H, H_Ar_), 6.86 (d, *J* = 8.4 Hz, 1H, H_Ar_), 6.75 (dd, *J* = 8.5, 2.8 Hz, 1H, H_Ar_), 6.43 (d, *J* = 15.7 Hz, 1H, CH=), 4.14 (s, 2H, CH_2_), 3.76 (s, 3H, OCH_3_), 3.39–3.22 (m, 4H, CH_2_), 2.73 (d, *J* = 8.6 Hz, 2H, CH_2_), 2.55–2.40 (m, 2H, CH_2_), 1.65–1.47 (m, 4H, CH_2_), 1.35 (dd, *J* = 16.7, 10.8 Hz, 4H, CH_2_), 1.00 (d, *J* = 4.9 Hz, 18H, CH_3_), 0.22 (s, 12H, CH_3_). ^13^C NMR (101 MHz, CD_3_OD) δ 168.8, 160.8, 150.0, 148.4, 144.0, 141.2, 134.6, 130.1, 129.2, 123.2, 122.4, 121.0, 119.9, 118.3, 113.9, 60.3, 59.5, 55.9, 54.1, 40.4, 38.9, 30.8, 30.3, 27.9, 27.4, 26.5, 19.4, −3.8. ESI-MS, positive mode: *m*/*z* (rel. int., %) = 685 (100) [M + H]^+^. HRMS (*m*/*z*): [*M*+H]^+^ calcd for C_37_H_61_N_2_O_4_SSi_2_, 685.3885; found, 685.3876.

Compound **6** was obtained from compound **38** (48 mg, 0.07 mmol), 16 mg (0.28 mmol) KF and 37 mg (0.28 mmol) Et_3_N × HCl using the general procedure 2. The title compound was isolated by flash chromatography using the cartridge Biotage HP-Sfär Silica HC Duo 20 μm, 10 g SiO_2_; gradient 2 to 40% MeOH in DCM. Yield – 22 mg (69%) of white powder. ^1^H NMR (400 MHz, CD_3_OD) δ 7.43 (d, *J* = 8.4 Hz, 1H, H_Ar_), 7.38 (d, *J* = 15.7 Hz, 1H, CH=), 7.00 (d, *J* = 2.1 Hz, 1H, H_Ar_), 6.95 (d, *J* = 2.8 Hz, 1H, H_Ar_), 6.90 (dd, *J* = 8.4, 2.1 Hz, 1H, H_Ar_), 6.79 (dd, *J* = 8.1, 2.8 Hz, 1H, H_Ar_), 6.76 (d, *J* = 8.1 Hz, 1H, H_Ar_), 6.35 (d, *J* = 15.7 Hz, 1H, CH=), 4.20 (s, 2H, CH_2_), 3.78 (s, 3H, OCH_3_), 3.38–3.33 (m, 2H, CH_2_),3.27 (t, *J* = 7.0 Hz, 2H, CH_2_), 2.83–2.70 (m, 2H, CH_2_), 2.59–2.49 (m, 2H, CH_2_), 1.65–1.52 (m, 4H, CH_2_), 1.43–1.22 (m, 4H, CH_2_). ^13^C NMR (101 MHz, CD_3_OD) δ 169.2, 161.0, 148.7, 146.7, 142.1, 134.7, 129.3, 128.3, 122.0, 118.5, 118.4, 116.4, 115.0, 114.1, 60.2, 59.5, 55.9, 41.1, 40.4, 30.8, 30.4, 27.9, 27.7, 27.2. ESI-MS, negative mode: *m*/*z* (rel. int., %) = 455 (100) [M–H]^−^. HRMS (*m*/*z*): [*M*+H]^+^ calcd for C_25_H_33_N_2_O_4_S, 457.2156; found, 457.2165.

Compound **39** was obtained from amine **23** (39 mg, 0.12 mmol), (*E*)-3-(3,4-bis­((*tert*-butyldimethylsilyl)­oxy)­phenyl)­acrylic acid (32 mg, 0.08 mmol), HBTU (45 mg, 0.12 mmol) and Et_3_N (56 mg, 0.3 mmol) using the general procedure 1. The product was isolated by flash chromatography using the cartridge Biotage HP-Sfär Silica HC Duo 20 μm, 10 g SiO_2_; gradient 2 to 20% MeOH in DCM. Yield – 57 mg (88%) of yellow oil. ^1^H NMR (400 MHz, CDCl_3_) δ 7.47 (d, *J* = 8.5 Hz, 1H, H_Ar_), 7.43 (d, *J* = 15.6 Hz, 1H, CH=), 7.02–6.95 (m, 2H, H_Ar_), 6.82–6.77 (m, 2H, H_Ar_), 6.27 (d, *J* = 15.6 Hz, 1H, CH=), 6.00 (s, 1H, H_Ar_), 4.40 (s, 2H, CH_2_), 3.80 (s, 3H, OCH_3_), 3.55–3.50 (m, 2H, CH_2_), 3.33 (q, *J* = 6.9 Hz, 2H, CH_2_), 2.93–2.86 (m, 2H, CH_2_), 2.76–2.67 (m, 2H, CH_2_), 1.73–1.64 (m, 2H, CH_2_), 1.58–1.48 (m, 2H, CH_2_), 1.34–1.24 (m, 4H, CH_2_), 0.98 (2 × s, 18H, CH_3_), 0.20 (2 × s, 12H, CH_3_). ^13^C NMR (101 MHz, CDCl_3_) δ 166.6, 159.9, 148.8, 147.2, 140.6, 134.3, 128.6, 127.6, 121.6, 121.2, 120.6, 118.9, 118.1, 114.5, 59.0, 55.7, 53.6, 46.9, 39.8, 38.7, 29.5, 28.9, 26.6, 26.0, 25.8, 18.6, 8.8, −3.9, −4.0. ESI-MS, negative mode: *m*/*z* (rel. int., %) = 713 (100) [M-H]^−^. HRMS (*m*/*z*): [*M*+H]^+^ calcd for C_39_H_65_N_2_O_4_SSi_2_, 713.4198; found, 713.4195.

Compound **7** was obtained from compound **39** (35 mg, 0.05 mmol), 12 mg (0.21 mmol) KF and 28 mg (0.20 mmol) Et_3_N × HCl using the general procedure 2. The title compound was isolated by flash chromatography using the cartridge Biotage HP-Sfär Silica HC Duo 20 μm, 10 g SiO_2_; gradient 2 to 20% MeOH in DCM. Yield – 13 mg (54%) of white powder. ^1^H NMR (400 MHz, CD_3_OD) δ 7.47 (d, *J* = 8.5 Hz, 1H, H_Ar_), 7.38 (d, *J* = 15.7 Hz, 1H, CH=), 7.08 (d, *J* = 2.4 Hz, 1H, H_Ar_), 7.01 (d, *J* = 2.4 Hz, 1H, H_Ar_), 6.90 (dd, *J* = 8.5, 2.4 Hz, 1H, H_Ar_), 6.84 (dd, *J* = 8.5, 2.4 Hz, 1H, H_Ar_), 6.76 (d, *J* = 8.1 Hz, 1H, H_Ar_), 6.36 (d, *J* = 15.7 Hz, 1H, CH=), 5.49 (s, 1H, NH), 4.32 (s, 2H, CH_2_), 3.80 (s, 3H, OCH_3_), 3.51–3.40 (m, 2H, CH_2_), 3.27 (t, *J* = 7.0 Hz, 2H, CH_2_), 2.90–2.81 (m, 2H, CH_2_), 2.70 (t, *J* = 8.0 Hz, 2H, CH_2_), 1.72–1.60 (m, 2H, CH_2_), 1.55 (t, *J* = 6.9 Hz, 2H, CH_2_), 1.41–1.26 (m, 8H, CH_2_). ^13^C NMR (101 MHz, CD_3_OD) δ 169.21, 161.15, 148.71, 146.72, 142.05, 134.95, 129.31, 128.30, 122.04, 118.89, 118.46, 116.44, 115.04, 114.77, 60.16, 59.37, 55.98, 40.46, 30.65, 30.42, 30.24, 30.15, 27.95, 27.85, 26.68.,ESI-MS, positive mode: *m*/*z* (rel. int., %) = 485 (100) [M + H]^+^.,HRMS (*m*/*z*): [*M*+H]^+^ calcd for C_27_H_36_N_2_O_4_S, 485.2469; found, 485.2469.

Compound **40** was obtained from amine **25** (55 mg, 0.20 mmol), (*E*)-3-(3,4-bis­((*tert*-butyldimethylsilyl)­oxy)­phenyl)­acrylic acid (67 mg, 0.17 mmol), HBTU (96 mg, 0.26 mmol) and Et_3_N (112 mg, 0.6 mmol) using the general procedure 1. The product was isolated by flash chromatography using the cartridge Biotage HP-Sfär Silica HC Duo 20 μm, 10 g SiO_2_; gradient 2 to 20% MeOH in DCM. Yield – 30 mg (26%) of yellow oil. ^1^H NMR (400 MHz, CDCl_3_) δ 7.48 (d, *J* = 15.5 Hz, 1H, CH=), 7.00–6.91 (m, 3H, H_Ar_), 6.80 (d, *J* = 7.7 Hz, 1H, H_Ar_), 6.72–6.68 (m, 2H, H_Ar_), 6.20 (d, *J* = 15.5 Hz, 1H, CH=), 5.73 (s, 1H, NH), 4.14–3.94 (m, 2H, CH_2_), 3.83 (s, 2H, CH_2_), 3.76 (s, 3H, OCH_3_), 3.36 (d, *J* = 6.2 Hz, 2H, CH_2_), 3.17–3.03 (m, 2H, CH_2_), 2.57–2.40 (m, 2H, CH_2_), 1.60–1.48 (m, 4H, CH_2_), 1.44–1.29 (m, 4H, CH_2_), 0.99 (s, 18H, CH_3_), 0.21 (2 × s, 12H, CH_3_). ^13^C NMR (101 MHz, CDCl_3_) δ 166.4, 165.7, 155.6, 153.5, 148.7, 147.0, 140.6, 128.4, 121.5, 121.5, 121.1, 120.5, 118.6, 116.2, 115.1, 113.9, 68.9, 67.9, 57.9, 55.7, 53.8, 53.4, 39.5, 38.6, 29.4, 26.6, 26.4, 25.90, 25.87, 25.7, 18.5, 18.4, −4.08, −4.12. ESI-MS, positive mode: *m*/*z* (rel. int., %) = 679 (100) [M + H]^+^. HRMS (*m*/*z*): [*M*+H]^+^ calcd for C_37_H_61_N_2_O_5_Si_2_, 669.4114; found, 669.4116.

Compound **8** was obtained from compound **40** (36 mg, 0.05 mmol), 10 mg (0.17 mmol) KF and 24 mg (0.17 mmol) Et_3_N × HCl using the general procedure 2. The title compound was isolated by preparative HPLC with gradient elution (B/A: 5/95 → 100/0). Yield – 12 mg (61%) of yellow solid. ^1^H NMR (400 MHz, CD_3_OD) δ 7.38 (d, *J* = 15.7 Hz, 1H, CH=), 7.00 (d, *J* = 2.0 Hz, 1H, H_Ar_), 6.95–6.86 (m, 2H, H_Ar_), 6.81–6.70 (m, 3H, H_Ar_), 6.35 (d, *J* = 15.7 Hz, 1H, CH=), 4.05–3.94 (m, 2H, CH_2_), 3.82 (s, 2H, CH_2_), 3.74 (s, 3H, OCH_3_), 3.28 (t, *J* = 7.0 Hz, 2H, CH_2_), 3.11–3.00 (m, 2H, CH_2_), 2.58–2.48 (m, 2H, CH_2_), 1.66–1.47 (m, 4H, CH_2_), 1.47–1.28 (m, 4H, CH_2_). ^13^C NMR (101 MHz, CD_3_OD) δ 141.8, 114.7, 121.8, 116.5, 116.8, 114.2, 118.1, 118.2, 72.4, 70.4, 68.4, 58.7, 60.7, 55.8, 53.8, 49.0, 40.1, 57.2, 59.2, 55.3, 27.5, 30.1, 28.4, 27.7 (^13^C signals of protonated carbons were derived from HSQC spectrum). ESI-MS, positive mode: *m*/*z* (rel. int., %) = 441 (100) [M + H]^+^. HRMS (*m*/*z*): [*M*+H]^+^ calcd for C_25_H_33_N_2_O_5_, 441.2384; found, 441.2392.

##### Compound **41**

To compound **15**, 8-(7-methoxy-2,3-dihydrobenzo­[1,4-*f*]­thiazepin-4­(5*H*)-yl)­octan-1-amine **23** (39 mg, 0.12 mmol) in 1 mL 1,2-dichloroethane, aldehyde **3** (62 mg, 0.16 mmol) in 1 mL 1,2-dichloroethane and sodium triacetoxyborohydride (36 mg, 0.17 mmol) were added. The reaction mixture was stirred at rt for 3 h. After the solvent was removed *in vacuo*, 10 mL water and 10 mL DCM were added. The organic solution was separated, the aqueous solution extracted with DCM (2 × 5 mL). The combined organic solutions were dried over Na_2_SO_4_. The filtrate was evaporated, and the product isolated by flash chromatography using the cartridge Biotage HP-Sfär Silica HC Duo 20 μm, 10 g SiO_2_; gradient 5 to 50% methanol in DCM. Yield – 30 mg (27%) of yellow solid. ^1^H NMR (400 MHz, CDCl_3_) δ 7.44 (d, *J* = 8.4 Hz, 1H, H_Ar_), 6.92 (dd, *J* = 8.3, 2.2 Hz, 1H, H_Ar_), 6.86 (d, *J* = 2.2 Hz, 1H, H_Ar_), 6.82–6.74 (m, 2H, CH =, H_Ar_), 6.69 (dd, *J* = 8.4, 2.8 Hz, 1H, H_Ar_), 6.49 (d, *J* = 15.8 Hz, 1H, CH=), 6.33–6.24 (m, 1H, H_Ar_), 4.11 (s, 2H, CH_2_), 3.79 (s, 3H, OCH_3_), 3.35–3.30 (m, 2H, CH_2_), 2.71–2.62 (m, 2H, CH_2_), 2.40–2.31 (m, 2H, CH_2_), 1.53–1.42 (m, 2H, CH_2_), 1.41–1.36 (m, 2H, CH_2_), 1.36–1.20 (m, 10H), 0.99 (2 × s, 18H, CH_3_), 0.20 (2 × s, 12H, CH_3_). ^13^C NMR (101 MHz, CDCl_3_) δ 159.1, 146.8, 145.0, 139.8, 133.5, 131.0, 128.2, 128.2, 127.7, 121.0, 119.8, 118.8, 116.9, 116.9, 111.9, 59.6, 58.4, 55.3, 53.4, 52.1, 40.0, 30.2, 29.6, 27.5, 27.4, 27.3, 27.2, 26.9, 25.9, 18.4, −4.1. LC-MS, positive mode: *m*/*z* (rel. int., %) = 699 (100) [M + H]^+^.

Compound **9** was obtained from compound **41** (30 mg, 0.04 mmol), 10 mg (0.17 mmol) KF and 24 mg (0.17 mmol) Et_3_N × HCl using the general procedure 2. The title compound was isolated by preparative HPLC with gradient elution (B/A: 5/95 → 100/0). Yield – 13 mg (54%) of yellow solid. ^1^H NMR (400 MHz, CD_3_OD) δ 7.56 (d, *J* = 8.5 Hz, 1H, H_Ar_), 7.18 (d, *J* = 2.8 Hz, 1H, H_Ar_), 7.03–6.94 (m, 2H, H_Ar_), 6.84 (dd, *J* = 8.3, 2.0 Hz, 1H, H_Ar_), 6.80–6.73 (m, 2H, CH =, H_Ar_), 6.08 (dd, *J* = 15.5, 7.7 Hz, 1H, CH=), 4.66 (s, 2H, CH_2_), 3.92 (t, *J* = 7.6 Hz, 2H, NCH_2_), 3.83 (s, 3H, OCH_3_), 3.78–3.56 (m, 2H, CH_2_), 3.22–2.89 (m, 6H, CH_2_), 1.85–1.66 (m, 4H, CH_2_), 1.44–1.25 (m, 7H, CH_2_). ^13^C NMR (101 MHz, CD_3_OD) δ 164.2, 150.4, 149.2, 144.7, 138.1, 131.3, 123.4, 118.9, 117.0, 116.4, 59.0, 58.7, 55.4, 43.3, 32.4, 32.3, 30.0, 29.9, 27.7, 27.4, 25.7. ESI-MS, positive mode: *m*/*z* (rel. int., %) = 471 (100) [M + H]^+^. HRMS (*m*/*z*): [*M*+H]^+^ calcd for C_27_H_39_N_2_O_3_S, 471.2676; found, 471.2674.

##### Compound **42**

Aldehyde **15** (61 mg, 0.19 mmol), amine **26** (45 mg, 0.15 mmol) were dissolved in 2 mL DCE. Then sodium triacetoxyborohydride (44 mg, 0.21 mmol) was added, and the obtained suspension was stirred for 24 h at rt. The suspension was filtered, and the filtrate was concentrated *in vacuo* to give a yellow oil. Then the oil was dissolved in DCM (10 mL), washed with sat. aq. NaHCO_3_ (2 × 5 mL) and dried over Na_2_SO_4_. The title compound was isolated by means of flash chromatography Biotage HP-Sfär Silica HC Duo 20 μm 10 g SiO_2_, gradient 5 to 100% MeOH in DCM. Yield – 32 mg (32%) of yellow oil. ^1^H NMR (400 MHz, CDCl_3_) δ 6.98–6.90 (m, 1H, H_Ar_), 6.87 (dd, *J* = 6.5, 2.2 Hz, 2H, H_Ar_), 6.80–6.76 (m, 2H, H_Ar_), 6.73–6.68 (m, 1H, H_Ar_), 6.47 (d, *J* = 15.9 Hz, 1H, CH), 6.10 (dt, *J* = 15.9, 7.0 Hz, 1H, CH), 4.05–3.99 (m, 2H, CH_2_), 3.86 (s, 2H, CH_2_), 3.76 (s, 3H, OCH_3_), 3.51 (d, *J* = 7.1 Hz, 2H, CH_2_), 3.18–3.10 (m, 2H, CH_2_), 2.76–2.68 (m, 1H, CH_2_), 2.54–2.45 (m, 1H, CH_2_), 2.07–1.99 (m, 2H, CH_2_), 1.67–1.60 (m, 2H, CH_2_), 1.57–1.47 (m, 2H, CH_2_), 1.34–1.22 (m, 6H, CH_2_), 1.00 (s, 9H, CH_3_), 0.99 (s, 9H, CH_3_), 0.210 (s, 6H, CH_3_), 0.207 (s, 6H, CH_3_). ^13^C NMR (101 MHz, CDCl_3_) δ 155.4, 153.6, 147.2, 146.9, 135.6, 133.9, 132.1, 131.4, 129.9, 123.2, 121.3, 121.1, 120.0, 119.2, 116.2, 113.2, 69.1, 57.6, 57.4, 55.6, 54.8, 53.2, 52.2, 29.4, 29.3, 27.3, 26.8, 25.9, 25.1, 18.5, −4.1. LC-MS, positive mode: *m*/*z* (rel. int., %) = 683 (100) [M + H]^+^. HRMS (*m*/*z*): [*M*+H]^+^ calcd for C_39_H_67_N_2_O_4_Si_2_, 683.4634; found, 683.4642.

Compound **10** was obtained from compound **42** (32 mg, 0.05 mmol), 11 mg (0.19 mmol) KF and 26 mg (0.19 mmol) Et_3_N × HCl using the general procedure 2. The title compound was isolated by preparative HPLC with gradient elution (B/A: 5/95 → 100/0). Yield – 7 mg (33%) of yellow solid. ^1^H NMR (400 MHz, CD_3_OD) δ 7.06 (d, *J* = 8.7 Hz, 1H, H_Ar_), 7.01–6.90 (m, 3H, H_Ar_), 6.84 (dd, *J* = 8.3, 2.1 Hz, 1H, H_Ar_), 6.79–6.72 (m, 2H, CH =, H_Ar_), 6.14–6.01 (m, 1H, CH=), 4.49–4.42 (m, 2H, NCH_2_), 4.39–4.02 (m, 2H, CH_2_), 3.91 (t, *J* = 7.6 Hz, 2H, CH_2_), 3.78 (s, 3H, OCH_3_), 3.74–3.55 (m, 2H, CH_2_), 3.25–3.09 (m, 4H, CH_2_), 1.85–1.65 (m, 4H, CH_2_), 1.45–1.26 (m, 8H, CH_2_). ^13^C NMR (101 MHz, CD_3_OD) δ 157.7, 155.1, 147.9, 146.7, 142.2, 128.8, 125.0, 123.1, 120.8, 118.1, 117.4, 116.4, 114.5, 114.0, 58.3, 57.9, 56.4, 56.2, 52.9, 47.9, 29.8, 29.7, 27.4, 27.3, 25.2, 24.9, 9.2. ESI-MS, positive mode: *m*/*z* (rel. int., %) = 455 (100) [M + H]^+^. HRMS *m*/*z* calcd for C_24_H_33_BN_3_O_3_S [M + H]^+^, 455.2904; found, 454.2922.

##### Compound **43**

To 4-(7-methoxy-2,3-dihydrobenzo­[1,4-*f*]­thiazepin-4­(5H)-yl)­butan-1-amine 19 (100 mg, 0.37 mmol) in 2 mL 1,2-dichloroethane, (*E*)-3-(3,4-bis­((*tert*-butyldimethylsilyl)­oxy)­phenyl)­acrylaldehyde 15 (191 mg, 0.49 mmol) in 3 mL 1,2-dichloroethane and sodium cyanoborohydride (60 mg, 0.96 mmol) were added. The reaction mixture was stirred at 60 °C overnight and concentrated *in vacuo*. The product was isolated by flash chromatography using the cartridge Biotage HP-Sfär Silica HC Duo 20 μm, 25 g SiO_2_; gradient 5 to 100% EtOAc in hexane. Yield – 50 mg (15%) of yellowish oil. ^1^H NMR (500 MHz, CDCl_3_) δ 7.47 (dd, *J* = 8.5, 2.1 Hz, 1H, H_Ar_), 6.97–6.84 (m, 3H, H_Ar_), 6.81–6.75 (m, 2H, H_Ar_), 6.45 (d, *J* = 15.7 Hz, 1H, CH=), 6.20–6.04 (m, 1H, CH=), 4.39–4.26 (m, 2H, CH_2_), 3.80.(s, 3H, OCH_3_), 3.51–3.39 (m, 2H, CH_2_), 2.82 (d, *J* = 12.2 Hz, 4H, CH_2_), 2.67–2.59 (m, 2H, CH_2_), 2.02–1.68 (m, 6H, CH_2_), 1.02–0.93 (m, 18H, CH_3_), 0.22–0.15 (m, 12H, CH_3_). ^13^C NMR (101 MHz, CDCl_3_) δ 147.7, 147.1, 137.1, 134.4, 132.6, 129.5, 121.3, 121.2, 120.2, 119.6, 118.5, 118.2, 56.5, 55.7, 26.1, 18.6, −3.9. ^11^B NMR (128 MHz, CDCl_3_) δ −21.46. ESI-MS, positive mode: *m*/*z* (rel. int., %) = 682 (100) [M + H]^+^. HRMS *m*/*z* calcd for C_36_H_61_BN_3_O_3_SSi_2_ [M + H]^+^, 682.4067; found, 682.4059.

Compound **11** was obtained in the course of cleavage of TBDMS groups in compound **43** (9 mg, 13 μmol) was performed in MeOH using 3 mg (53 μmol) KF together with 7 mg (53 μmol) Et_3_N × HCl. The reaction mixture was stirred overnight at rt. After MeOH was evaporated, the title compound was isolated by preparative HPLC with gradient elution (B/A: 20/80 → 100/0). Yield – 5 mg (85%). ^1^H NMR (400 MHz, CD_3_OH) δ 7.41 (d, *J* = 8.4 Hz, 1H, H_Ar_), 6.92–6.86 (m, 2H, H_Ar_), 6.78–6.72 (m, 3H, H_Ar_), 6.53 (d, *J* = 16.1 Hz, 2H, CH=), 6.09–6.00 (m, 2H, CH=), 4.08 (s, 2H, CH_2_), 3.76 (s, 3H, OCH_3_), 3.28–3.24 (m, 2H, CH_2_), 2.81–2.67 (m, 4H, CH_2_), 2.42 (t, *J* = 7.2 Hz, 2H, CH_2_), 1.69 (d, *J* = 7.1 Hz, 2H, CH_2_), 1.61–1.54 (m, 2H, CH_2_), 1.34–1.29 (m, 2H, CH_2_). ^13^C NMR (101 MHz, CD_3_CN) δ 160.9, 146.3, 145.8, 138.0, 135.2, 129.7, 120.5, 119.9, 118.8, 116.3, 114.0, 56.4, 51.8, 23.4, 22.3. ^11^B NMR (128 MHz, CD_3_CN) δ −20.6. ESI-MS, positive mode: *m*/*z* (rel. int., %) = 454 (100) [M + H]^+^. HRMS *m*/*z* calcd for C_24_H_33_BN_3_O_3_S [M + H]^+^, 454.2334; found, 454.2343.

Compound **44** was obtained from 6-(7-methoxy-2,3-dihydrobenzo­[1,4-*f*]­oxazepin-4­(5*H*)-yl)­hexan-1-amine **25** (100 mg, 0.34 mmol), (*E*)-3-(3,4-bis­((*tert*-butyldimethylsilyl)­oxy)­phenyl)­acrylaldehyde **15** (173 mg, 0.44 mmol) and sodium cyanoborohydride (56 mg, 0.89 mmol) using procedure described for compound **43**. The product was isolated by flash chromatography using Biotage HP-Sfär Silica HC Duo 20 μm, 25 g; gradient 5 to 100% EtOAc in hexane. Yield – 66 mg (27%) of yellowish oil. ^1^H NMR (400 MHz, Chloroform-*d*) δ 7.47 (d, *J* = 8.5 Hz, 1H, H_Ar_), 6.97–6.83 (m, 3H, H_Ar_), 6.83–6.75 (m, 2H, H_Ar_), 6.52 (d, *J* = 15.3 Hz, 1H, CH=), 6.13 (dt, *J* = 15.3, 7.3 Hz, 1H, CH=), 4.34 (s, 2H, NCH_2_), 3.81 (s, 3H, OCH_3_), 3.54–3.42 (m, 2H, CH_2_), 2.89–2.79 (m, 4H, CH_2_), 2.61–2.51 (m, 2H, CH_2_), 1.68 (s, 4H, CH_2_), 1.40–1.28 (m, 4H, CH_2_), 0.99 (2 × s, 18H, CH_3_), 0.25–0.12 (m, 12H, CH_3_). ^13^C NMR (101 MHz, CD_3_OD) δ 161.3, 148.5, 148.1, 137.7, 135.1, 131.4, 129.3, 122.3, 121.5, 120.3, 120.0, 119.1, 115.6, 61.5, 56.1, 52.9, 32.7, 27.5, 26.5, 23.6, 20.9, 19.3, 19.3, 14.4, 14.4, 13.5, −3.8. ^11^B NMR (128 MHz, CD_3_OD) δ −43.6. ESI-MS, positive mode: *m*/*z* (rel. int., %) = 710 (100) [M + H]^+^. HRMS *m*/*z* calcd for C_38_H_65_BN_3_O_3_SSi_2_ [M + H]^+^, 710.4380; found, 710.4383.

Compound **12** was prepared from compound **44** (6 mg, 9 μmol) in MeOH according the general procedure 2. The title compound was isolated by preparative HPLC with gradient elution (B/A: 10/90 → 100/0). Yield – 3.4 mg (83%). ^1^H NMR (400 MHz, CD_3_OD) δ 7.56 (d, *J* = 8.5 Hz, 1H, H_Ar_), 7.19 (d, *J* = 2.8 Hz, 1H, H_Ar_), 6.99 (dd, *J* = 8.5, 2.8 Hz, 1H, H_Ar_), 6.91 (d, *J* = 2.0 Hz, 1H, H_Ar_), 6.77 (dd, *J* = 8.1, 2.0 Hz, 1H, H_Ar_), 6.73 (d, *J* = 8.1 Hz, 1H, H_Ar_), 6.56 (d, *J* = 15.8 Hz, 1H, CH=), 6.11–6.01 (m, 1H, CH=), 4.65 (s, 2H, CH_2_), 3.84 (s, 3H, OCH_3_), 3.67–3.56 (m, 2H, CH_2_), 3.50–3.39 (m, 1H, NH), 3.22–2.90 (m, 4H, CH_2_), 2.89–2.70 (m, 2H, CH_2_), 1.95–1.55 (m, 6H, CH_2_), 1.40 (s, 4H, CH_2_). ^13^C NMR (101 MHz, CD_3_OD) δ 161.7, 147.2, 146.6, 138.5, 135.6, 129.6, 120.7, 120.3, 119.9, 118.6, 116.5, 116.4, 116.3, 114.1, 56.9, 56.2, 53.7, 52.6, 33.4, 28.4, 27.4, 27.1, 27.0, 26.3, 26.2, 25.0. ^11^B NMR (128 MHz, CD_3_OD) δ −21.2. ESI-MS, positive mode: *m*/*z* (rel. int., %) = 482 (100) [M + H]^+^. HRMS *m*/*z* calcd for C_26_H_37_BN_3_O_3_S [M + H]^+^, 482.2648; found, 482.2665.

Compound **45** was obtained from 8-(7-methoxy-2,3-dihydrobenzo­[1,4-*f*]­thiazepin-4­(5*H*)-yl)­octan-1-amine **23** (160 mg, 0.49 mmol), (*E*)-3-(3,4-bis­((*tert*-butyldimethylsilyl)­oxy)­phenyl)­acrylaldehyde **15** (253 mg, 0.64 mmol) and sodium cyanoborohydride (70 mg, 1.11 mmol) using the procedure described above for compound **43**. The product was isolated by flash chromatography using the cartridge Biotage HP-Sfär Silica HC Duo 20 μm, 25 g SiO_2_; gradient 5 to 100% EtOAc in hexane. Yield – 115 mg (30%) of yellowish oil. ^1^H NMR (400 MHz, CDCl_3_) δ 7.44 (dd, *J* = 8.4, 1.3 Hz, 1H, H_Ar_), 6.91–6.82 (m, 1H, H_Ar_), 6.85–6.73 (m, 2H, H_Ar_), 6.73–6.57 (m, 1H, H_Ar_), 6.66–6.57 (m, 1H, H_Ar_), 6.51 (d, *J* = 15.7 Hz, 1H), 6.07 (dt, *J* = 15.3, 7.3 Hz, 1H), 4.14–4.07 (m, 2H, NCH_2_), 3.78 (s, 3H, OCH_3_), 3.36–3.27 (m, 2H, CH_2_), 2.86–2.77 (m, 2H, CH_2_), 2.73–2.58 (m, 2H, CH_2_), 2.39–2.31 (m, 2H, CH_2_), 1.53–1.41 (m, 2H, CH_2_), 1.32–1.17 (m, 6H, CH_2_), 0.98 (2 × s, 18H, CH_3_), 0.19 (2 × s, 12H, CH_3_). ^13^C NMR (101 MHz, CDCl_3_) δ 171.3, 159.2, 147.8, 147.1, 137.6, 133.7, 129.3, 127.9, 121.3, 120.3, 119.5, 117.9, 117.1, 112.2, 60.5, 59.6, 58.4, 55.8, 55.5, 52.4, 52.1, 30.2, 29.4, 29.2, 27.3, 26.8, 26.0, 21.2, 18.6, 14.3, −3.9. ^11^B NMR (128 MHz, CDCl_3_) δ −20.0. ESI-MS, positive mode: *m*/*z* (rel. int., %) = 739 (100) [M + H]^+^. HRMS *m*/*z* calcd for C_40_H_69_BN_3_O_3_SSi_2_ [M + H]^+^, 738.4693; found, 738.4710.

Compound **13** was prepared from compound **45** (15 mg, 22 μmol) in MeOH according to the general procedure 2. The title compound was isolated by preparative HPLC with gradient elution (B/A: 10/90 → 100/0). Yield – 3.1 mg (30%) of yellowish oil. ^1^H NMR (400 MHz, CD_3_OD) δ 7.56 (d, *J* = 8.5 Hz, 1H, H_Ar_), 7.20–7.16 (m, 1H, H_Ar_), 6.98 (dd, *J* = 8.5, 2.8 Hz, 1H, H_Ar_), 6.91 (d, *J* = 2.0 Hz, 1H, H_Ar_), 6.80–6.63 (m, 2H, H_Ar_), 6.55 (d, *J* = 15.9 Hz, 1H, CH=), 6.26 (br. s, 1H, NH), 6.06 (dt, *J* = 15.9, 7.9 Hz, 1H, CH=), 4.66 (s, 2H, CH_2_), 3.83 (s, 3H, CH_3_), 3.78–3.56 (m, 3H, CH_2_), 3.48–3.38 (m, 1H, CH_2_), 3.23–2.91 (m, 4H, CH_2_), 2.86–2.64 (m, 2H, CH_2_), 1.88–1.64 (m, 4H, CH_2_), 1.43–1.24 (m, 8H, CH_2_). ^13^C NMR (101 MHz, CD_3_OD) δ 161.6, 147.1, 146.5, 138.4, 135.5, 133.7, 129.7, 129.5, 120.6, 120.3, 119.9, 118.6, 116.5, 116.4, 114.2, 56.8, 56.2, 54.2, 53.7, 52.7, 33.4, 29.9, 29.8, 28.4, 27.7, 27.6, 27.4, 26.3, 25.1. ESI-MS, positive mode: *m*/*z* (rel. int., %) = 739 (100) [M + H]^+^. HRMS *m*/*z* calcd for C_28_H_41_BN_3_O_3_S [M + H]^+^, 510.2961; found, 510.2976.

### Cell Culture

HEK-293 cells stably expressing WT RyR2 and R-CEPIA1*er* as well as HEK-293 cells stably expressing only WT RyR2 were generously donated by Dr. Takashi Murayama, Juntendo University School of Medicine, Tokyo. The cells were cultured in Dulbecco’s Modified Eagle medium (DMEM, Thermo Fisher Scientific, Darmstadt, Germany) supplemented with 10% fetal bovine serum (FBS, Thermo Fisher Scientific, Darmstadt, Germany), 15 μg/mL Blasticidine (Invivogen, Toulouse, France) and 100 μg/mL Hygromycin (Merck KGaA, Darmstadt, Germany) in a humidified 5% CO_2_ incubator at 37 °C. HEK 293-RyR2-R-CEPIA1er cell medium additionally contained 400 μg/mL of G418 (Thermo Fisher Scientific, Darmstadt, Germany) To induce the expression of WT RyR2, 2 μg/mL Doxycycline (Merck KGaA, Darmstadt, Germany) was added 24 h before experiments.

HL-1 cells (SCC065, Merck KGaA, Darmstadt, Germany) were cultivated in Claycomb’s Medium (51800C, Merck KGaA, Darmstadt, Germany), supplemented with Glutamax, 10% FBS, 0.9% Penicillin/Streptomycin and 0.1 mM Noradrenaline (Merck KGaA, Darmstadt, Germany) on fibronectin/gelatin (Merck KGaA, Darmstadt, Germany) coated culture bottles or plates 2 days before plating.

CHO Na_V_1.5-Duo cells (B’SYS GmbH, Witterswil, Switzerland) were cultivated in hyClone Ham’s Nutrient Mixtures F12 (Cytiva, Dreieich Germany) supplemented with 10% FBS, 0.9% Penicillin/Streptomycin 250 μg/mL G-418 and 1 μg/mL Puromycine in culture bottles or plates 2 days before plating.

### Time-Lapse [Ca^2+^]_ER_ Assay

The fluorescent plate reader [Ca^2+^]_ER_ assay was performed according to the slightly modified protocol of Murayama & Kurebayashi.^46^ HEK-293 WT RyR2 R-CEPIA1*er* cells (50,000 cells per well) were cultivated for 24 h in black-walled, clear-bottom 96-well microplates (Corning, Amsterdam, The Netherlands) covered with fibronectin (10 μg/mL, Roche, Mannheim, Germany) in a humidified incubator at 37 °C and 5% CO_2_. After the cells were inducted with Doxycycline for 24 h, changes in ER calcium concentrations [Ca^2+^]_ER_ were measured on a FlexStation 3 Multimode Microplate Reader at 37 °C. The 1000 × stock solutions of the test compounds were prepared in DMSO. Briefly, the culture medium was removed and 100 μL of Tyrode’s solution containing 2 mM CaCl_2_ was added to the cells. The time courses were recorded in each well in a Flex mode for 5 min. Fluorescence was evoked by the 535 nm excitation wavelength and collected in a bottom-read mode at 585 nm, cutoff—570 nm, 10 flashes per read. The data were recorded every 10 s. 100 μL of 0–300 μM solution of a test compound in Tyrode’s solution containing 2 mM CaCl_2_ was injected in each well (pipet height—130 μL, speed 16 μL/s) at the 90 s time point. *F*/*F*_0_, the ratio of the averaged fluorescence intensities between the initial (before injection compound *F*_0_) and the last (*F*) 100 s of the read out were taken for the analysis. The 0.1 v/v% DMSO and 2 mM CaCl_2_ Tyrode’s solution was used as a control. For dose response curves, cells were pretreated with 4 mM caffeine for 10 min. The dose response curves were calculated using the software package GraphPad Prism version 8.3.1. (GraphPad Software, Inc.). All data are presented as mean ± S. in 8–16 independent experiments. *Y* = Bottom + (Top-Bottom)/(1 + 10^∧^((LogEC_50_ – X)*HillSlope)) was used where HillSlope describes the steepness of the curve, Top and Bottom are plateaus in the units of the *Y* axis.

### Cells Preparation for Imaging Experiments

Cells were seeded in CELLview Slides (Greiner Bio-One, Frickenhausen, Germany); 8000 cells per well or in 35 mm glass bottom dish (Cellvis, USA); well 14 mm, glass thickness #1.5, coated with fibronectin, 30,000 cells per well. The induction of HEK-293 RyR2 R-CEPIA*1er* cells was done by 24 h incubation in doxycycline solution (0.1 μg/mL).

The CHO Na_V_1.5 cells were seeded on fibronectin coated coverslips and fixed with glyoxal according to the Richter et al. protocol.^76^ The immunostaining of cells was done with anti-Na_V_1.5 antibody produced in rabbit (Merck KGaA, Darmstadt, Germany), which were stained with Star Red (Abberior GmbH, Göttingen, Germany). For the nucleus staining, we used 300 nM DAPI. The cells were mounted in Mowiol.

### Fluorescence Microscopy

Confocal imaging was performed on a Leica SP8 (Leica Microsystems, Mannheim, Germany) inverted confocal microscope equipped with an HC PL APO CS2 63x/1.40 oil objective. All live-cell images were acquired using a 700 Hz bidirectional scanner, and a 92.26 × 92.26-μm field of view (1024 × 1024 pixels and 90 nm pixels), pinhole of 95.6 μm diameter (1 AU), pixel dwell time—325 ns. R-CEPIA was excited with 561 nm laser (2% intensity) and detected with a HyD detector in the 600 nm -630 nm range. FLIPR Calcium 6 was excited with 488 nm laser (1% intensity) and detected with HyD detector in the 510 nm -550 nm range. Star Red was excited with 633 nm laser (1% intensity) and detected with a HyD detector in the 650–690 nm range. DAPI was excited with 405 nm laser (1% intensity) and detected with a HyD detector in the 420–470 nm range.

### Caffeine Assay on HL-1 Cells

HL-1 cells (50000 per well) were cultivated for 24 h in black-walled, clear-bottom 96-well microplates (Corning, Amsterdam, The Netherlands) covered with fibronectin/gelatin (Merck KGaA, Darmstadt, Germany) in a humidified incubator at 37 °C and 5% CO_2_.

Changes in Ca^2+^ concentration were measured on a FlexStation 3 Multimode Microplate Reader using FLIPR 6 Calcium Assay Kit from Molecular Devices (Molecular Devices LLC, San Jose, USA) at 37 °C. The 1000 × stock solutions of the test compounds were prepared in DMSO. 100 μL solution of test compound in Tyrode’s solution containing FLIPR Calcium 6 dye was incubated for 2 h at 37 °C and 5% CO_2_ in a humidified incubator. The compounds were not washed away before fluorescence measurements. The time courses were recorded in each well for 1 min. Fluorescence was evoked at 485 nm excitation wavelength and read out in a bottom-read mode at 525 nm, cutoff—515 nm, 6 flashes per read. Data were recorded every 1.5 s. To initiate Ca^2+^ influx, 25 μL of 50 mM caffeine solution in Tyrode’s solution containing 2 mM CaCl_2_ was injected in each well (pipet height—105 μL, speed 125 μL/s) at 10 s time point, resulting in 10 mM final concentration in the well. The differences between the caffeine induced peak minus basal fluorescence, Δ*F*_Caff_, were taken for the analysis. The Tyrode’s solution +0.1 v/v% DMSO was used as a control. The dose response curves were calculated using the software package GraphPad Prism version 8.3.1. All data are presented as mean ± SD from 6 independent experiments. *Y* = Bottom + (Top – Bottom)/(1 + 10^∧^((LogEC_50_ – *X*)*HillSlope)) was used where HillSlope describes the steepness of the curve, Top and Bottom are plateaus in the units of the *Y* axis.

**Isolation of Microsomal Membrane Vesicles from mouse hearts** was done according to procedure described earlier.^14^ All animal procedures were performed in accordance with national and institutional regulations. Sacrificing rodents for subsequent preparation of tissue did not require specific authorization or notification (§7 Abs. Two Satz 3 TierSchG).

### SERCA2 Activity Measurements

Mouse ventricular microsomes (4–5 μg of total protein) were diluted in 100 μL of the assay buffer used for measurement of SERCA activity (described below) and incubated at room temperature for 10 min prior to the experiments. The measurements of SERCA2 activity were made at 25 °C by using a NADH -coupled ATPase assay as described in Radnai et al. with slight modifications.^51^ Briefly, the assay buffer contained 60 mM MOPS, 120 mM KCl, 6 mM MgCl_2_, 1 mM EGTA, 5 mM NaN_3_, 0.5 mM phosphoenolpyruvate and 0.67 mM CaCl_2_, pH 7.0. Before the reaction was started by the injection of 10 μL ATP (resulting in 2 mM final concentration in the well), 5 units/mL of both lactate dehydrogenase and pyruvate kinase, 0.5 mM NADH, and 1 μM Ca^2+^ ionophore A-23187 (Sigma, C-7522) were added to the diluted sample with or without the tested compound (volume −150 μL). The 0.67 mM Ca/EGTA buffer has a free Ca^2+^ concentration of about 2 μM. The NADH fluorescence decrease was recorded each 2 s for 5 min course on a FlexStation 3 Multi-Mode Microplate Reader in 96-well glass bottom plates (MatTek Corporation; Cat. No. PBK96G-1.5-5-F). Fluorescence was evoked by 380 nm excitation wavelength and collected in a bottom-read mode at 425 nm, cutoff—470 nm, 4 flashes per read. ATP was injected at 60 s, speed—125 μL/s, pipet height—140 μL. The data were analyzed with computer software GraphPad Prism version 8.3.1 (GraphPad Software, Inc.). The difference in signal decrease with and without microsomes was taken as an indicator for the additional ATPase activity performed by SERCA2. The obtained values for maximal SERCA2 activity were normalized for total protein concentration. Maximal SERCA2 activity values are reported as percentages of control (0.1% DMSO) values. Microsomal samples that were kept on ice until usage and set to 100%.

### FLIPR Membrane Potential Dye Assay

Chinese hamster ovary (CHO, 50000 per well) cells stably expressing Na_V_1.5 (hH1a) α subunit were cultivated for 24 h in black-walled, clear-bottom 96-well microplates (Corning, Amsterdam, The Netherlands) in a humidified incubator at 37 °C and 5% CO_2_.

Na^+^ ion channel activities were measured on a Molecular Devices FlexStation 3 Multi-Mode Microplate Reader using FLIPR Membrane Potential Assay Kit (FMP, Molecular Devices LLC, München, Germany) at 37 °C according to the protocol of the manufacturer. In brief, the 1000 × stock solutions of the test compounds were prepared in DMSO. 100 μL solution of test compound in Tyrode’s solution containing FLIPR Membrane Potential Blue dye was incubated for 30 min at 37 °C and 5% CO_2_ in a humidified incubator. The compounds were not washed away before fluorescence measurements. The time courses were recorded in each well for 1.5 min. Fluorescence was evoked at 530 nm excitation wavelength and read out in a bottom-read mode at 565 nm (cutoff 550 nm). Data were recorded every 5 s, exposure (medium)—3 flashes, excitation bandwidth—10 nm, emission bandwidth—15 nm. 100 μL of 200 μM Veratridine in Tyrode’s solution was injected in each well (speed 16 μL/s) at 30 s time point, resulting in 100 μM final concentration in the well. The ratio between the Veratridine induced peak minus basal fluorescence (ratio = maximum/minimum) were taken for the analysis. Minimum was determined between reads 1 and 8, and maximum was determined between reads 6 and 20. The Tyrode’s solution +0.1 v/v% DMSO was used as a control. The Na^+^ channel blocker Tetracaine, an intracellular Na^+^ channel blocker (100 μM), was used as the negative control. The dose response curves were calculated using the software package GraphPad Prism version 8.3.1. All data are presented as mean ± SD from 3 independent experiments. *Y* = Bottom + (Top – Bottom)/(1 + 10^∧^((LogEC_50_ – *X*)*HillSlope)) was used where HillSlope describes the steepness of the curve, Top and Bottom are plateaus in the units of the *Y* axis.

### Statistical Analysis

Normality and log normality tests were performed using the Shapiro-Wilk test. The significance between means were tested using unpaired Student’s *t* test, nonparametric Mann–Whitney test and one-way ANOVA. The Dunnett test was performed to compare every mean to a control (0.1% DMSO) mean. Results are represented as mean ± SD or mean ± SEM. A value of *P* < 0.05 was considered significant.

### Western Blot Analysis

RyR2, SERCA2a and Na_V_1.5 protein levels were determined by Western blot analysis. 8 μL cell lysate was mixed with 2 μL NuPAGE LDS Sample buffer (Thermo Fisher) and heated at 70 °C for 10 min. The samples (100–150 μg protein) were loaded onto a 3–8% gradient Tris-acetate-gel or 4–20% SDS- Tris-glycine gel and the gel was run for 1 h at 150 V in running buffer. After that the gel was equilibrated for 10 min in 20% EtOH, transferred onto a nitrocellulose membrane and blotted with the iBlot 2 system (dry blot) for 10 min at 25 V. Membranes were blocked with 5% skim milk for 30 min at room temperature and incubated with primary antibodies against RyR2 (Life PA5–87416 polyclonal, 1:1000), SERCA2a (Badrilla A010–20 rabbit polyclonal, 1:1000) or Na_V_1.5 (Sigma, polyclonal, 1:200) overnight at 4 °C with moderate shaking. Binding of the primary antibody was detected by Horseradish peroxidase (HRP)-conjugated secondary antibody (1 h, rt). The detection was done with the Western Lightning Plus ECL Kit (PerkinElmer LAS GmbH, Germany, Rodgau) and the Amersham Imager 600.

### Cytotoxicity

Cell viability was assessed using CytoTox-Glo Cytotoxicity Assay according to the manufacturing procedure (Promega GmbH). Briefly, HL-1 cells were cultivated for 24 h in black-walled, clear-bottom 96-well microplates (Corning) covered with fibronectin/gelatin in DMEM, containing different concentrations of the tested compound (10,000 cells/well). Luminescence of a luminogenic peptide substrate was measured before and after addition of the lysis reagent (digitonin). The viable cell contribution was determined by a subtractive method. 0.1% DMSO treated cell were taken as a control.

**Membrane permeability** was assessed using PermeaPad Plate according to the manufacturing procedure (innoME GmbH, Espelkamp, Germany). Briefly, 200 μL (*V*_A_) water was added into acceptor plate. 200 μL (*V*_D_) of 100–500 μM test compound in water was added directly to the well membranes of the donor plate. The donor plate contains a biomimetic membrane for simulating passive mass transfer through different barriers in the body. Then the donor plate was placed into the acceptor plate wells and incubated at room temperature for 24 h in dark. To determine peak absorbance of test compounds, absorbance spectra of acceptor solutions and initial solutions for each test compound were read out. The Permeability Rate (*P*_e_) was calculated using the formula:

*P*_e_ = *C* × −ln (1 – OD_A_/OD_B_) cm/s, where *OD*_A_ is the absorbance of acceptor solution, *OD*_B_ is the absorbance of the standard solution; if the compound is able to permeabilize the membrane and fully reach equilibrium, 250 μM will be the final concentration of solution in the donor and acceptor wells. The coefficient *C* was calculated using the formula:

$$C=\frac{V_{D}\times V_{A}}{\left(V_{D}+V_{A}\right)\times \mathrm{Area}\times \mathrm{time}}$$

where *V*_A_—acceptor volume (cm^3^), *V*_D_—donor volume (cm^3^), membrane area 0.24 cm^2^, time is 86,400 s.

## Supplementary Material



## Notes

Western blot analysis (Figures S1 and S2); fluorescence measurements of caffeine addition to HL-1, HEK RyR2 WT, and HEK RyR2 R-CEPIA1er cells (Figure S3); CA fluorescence at various pH (Figure S4); CA fluorescence at caffeine assay conditions (Figure S5); comparison of the fluorescence properties of CA and compound **9** (Figure S6); Plots of NADH consumption in the presence of compound **11**, **12**, and **13** (Figures S7–S9); effects of compounds **9**, **11**–**13** on kinetic rate of NADH–NAD^+^ conversion (Figure S10); molecular docking of CDN1163 in the human E2.ATP SERCA2a (Figure S11); molecular docking of compounds **9**, **11**, and **12** in the human E2.ATP SERCA2a (Figure S12); confocal microscopy images of CHO Na_V_1.5 Duo cells (Figure S13); FLIPR membrane potential assay on CHO Na_V_1.5 Duo cells (Figure S14); cell viability measurements (Figure S15); SERCA2 activity measurements on microsomal membrane vesicles derived from HEK-293T cells (Figure S16); Scheme S1; ^1^H, ^13^C, and ^11^B NMR spectra (Figures S17–S60); HPLC spectra (Figures S61–S73) (PDF)

## Abbreviations

CA: caffeic acid
CHO: Chinese hamster ovary cells
DCM: Dichloromethane
DMEM: Dulbecco’s modified eagle medium
ER: endoplasmic reticulum
HATU: *O*-(7-azabenzotriazol-1-yl)-*N,N,N*′*,N*′-tetramethyluronium hexafluorphosphat
HBTU: 2-(1*H*-benzotriazol-1-yl)-1,1,3,3-tetramethyluronium hexafluorophosphate
HEK-293: human embryonic kidney 293 cells
HF: heart failure
HL-1: atrial muscle cells
NADH: nicotinamide adenine dinucleotide
Na_V_1.5: cardiac voltage-gated sodium channel
RMSD: root mean squared deviation
hRyR1: human ryanodine receptor 1
RyR2: ryanodine receptor 2
SERCA2a: sarco/endoplasmic reticulum Ca^2+^-ATPase 2a
SER: sarcoendoplasmic reticulum
TBDMS: *tert*-butyldimethylsilyl
WT: wild type
