# Supplementary material for: Multitargeted Caffeic Acid Derivatives Inhibit Cardiac RyR2- and NaV1.5- Channels but Stimulate SERCA2a Pump Activity
Source: ACS Omega. 2025 Sep 17;10(38):43831–54. doi: 10.1021/acsomega.5c04399 (PMC12489660; doi:10.1021/acsomega.5c04399)
Supplement: Supplementary file 1 [file ao5c04399_si_001.pdf]

SUPPORTING INFORMATION

Multi-targeted Caffeic Acid Derivatives inhibit cardiac RyR2- and $Na_V1.5$- channels but stimulate SERCA2a pump activity

Gyuzel Y. Mitronova[1,3*], Christine Quentin[1], Vladimir N. Belov[1], Kamila A. Kiszka[1], Jörg W. Wegener[3,4], Stephan E. Lehnart[2,3]

[1] Department of NanoBiophotonics, Max Planck Institute for Multidisciplinary Sciences, Am Fassberg 11, 37077 Göttingen, Germany

[2] Department of Cardiology & Pulmonology, Heart Research Center Göttingen, University Medical Center Göttingen, Robert-Koch-Str 42a, 37075 Göttingen, Germany

[3] DZHK (German Centre for Cardiovascular Research), Partner Site Lower Saxony, 37075 Göttingen, Germany

[4] Department of Translational Pharmacology, Medical School OWL, Bielefeld University, Universitätsstraße 25, 33615 Bielefeld, Germany

*Corresponding Author:
Gyuzel Y. Mitronova (G.Y.M); E-Mail: gyuzel.mitronova@mpinat.mpg.de;

SUPPLEMENTARY FIGURES AND SCHEMES

Figure S1. Western blot analysis of HEK293 and HL-1 cells. 5

Figure S2. Protein expression patterns. Western blots using anti-SERCA2a and anti-$Na_V1.5$........ 5

Figure S3. Addition of 10 mM caffeine to HL-1, HEK RyR2 WT and HEK RyR2 R-CEPIA1er, cells........ 6

Figure S4. Fluorescence of CA (33 μM) at various pH. 6

Figure S5. CA fluorescence does not affect Caffeine assay results........ 6

Figure S6. Comparison of the fluorescence properties of CA and compound **9** 7

Figure S7. Plots of NADH consumption (a) in the presence of compound **11**. 7

Figure S8. Plots of NADH consumption (a) in the presence of compound **12** 8

Figure S9. Plots of NADH consumption (a) in the presence of compound **13** 8

Figure S10. Compounds **9**, **11** - **13** evoke enhancements in the kinetic rate of NADH - NAD+ conversion in a dose-dependent manner. 9

Figure S11. CDN1163 in the human E2.ATP SERCA2a. 10

Figure S12. Compounds **9**, **11** and **12** in the human E2 SERCA2a 11

Figure S13. Confocal microscopy images of CHO $Na_V1.5$ Duo cells........ 12

Figure S14. FLIPR membrane potential assay on CHO $Na_V1.5$ Duo cells 12

Figure S15. Cell viability after 24 h........ 13

Figure S16. SERCA2 activity measurements on microsomal membrane vesicles derived from HEK-293T cells........ 13

Scheme S1. 14

NMR SPECTRA........ 15

Figure S17. 1H and 13C spectra of compound **1** 15

Figure S18. ^{1}H and ^{13}C spectra of compound **28**. 16

Figure S19. ^{1}H and ^{13}C spectra of compound **2**. 17

Figure S20. ^{1}H and ^{13}C spectra of compound **29**. 18

Figure S21. ^{1}H and ^{13}C spectra of compound **17**. 19

Figure S22. ^{1}H and ^{13}C spectra of compound **30**........ 20

Figure S23. ^{1}H and ^{13}C spectra of compound **18**. 21

Figure S24. ^{1}H spectrum of compound **30**. 22

Figure S25. ^{1}H and ^{13}C spectra of compound **3**........ 23

Figure S26. ^{1}H spectrum of compound **32**........ 24

Figure S27. ^{1}H and ^{13}C spectra of compound **21** 25

Figure S28. ^{1}H spectrum of compound **33**........ 26

Figure S29. ^{1}H spectrum of compound **4** 26

Figure S30. ^{1}H and ^{13}C spectra of compound **34** ... 27
Figure S31. ^{1}H spectrum of compound **5** ... 28
Figure S32. ^{1}H spectrum of compound **35** ... 28
Figure S33. ^{1}H and ^{13}C spectra of compound **22** ... 29
Figure S34. ^{1}H and ^{13}C spectra of compound **23** ... 30
Figure S35. ^{1}H spectrum of compound **37** ... 31
Figure S36. ^{1}H and ^{13}C spectra of compound **24** ... 32
Figure S37. ^{1}H and ^{13}C spectra of compound **25** ... 33
Figure S38. ^{1}H and ^{13}C spectra of compound **26** ... 34
Figure S39. ^{1}H and ^{13}C spectra of compound **38** ... 35
Figure S40. ^{1}H and ^{13}C spectra of compound **6** ... 36
Figure S41. ^{1}H and ^{13}C spectra of compound **39** ... 37
Figure S42. ^{1}H and ^{13}C spectra of compound **7** ... 38
Figure S43. ^{1}H and ^{13}C spectra of compound **40** ... 39
Figure S44. ^{1}H and gHSQCAD spectra of compound **8** ... 40
Figure S45. ^{1}H and ^{13}C spectra of compound **41** ... 41
Figure S46. ^{1}H and ^{13}C spectra of compound **9** ... 42
Figure S47. ^{1}H and ^{13}C spectra of compound **42** ... 43
Figure S48. ^{1}H and ^{13}C spectra of compound **10** ... 44
Figure S49. ^{1}H and ^{13}C spectra of compound **43** ... 45
Figure S50. ^{11}B spectrum of compound **43** ... 46
Figure S51. ^{1}H and ^{13}C spectra of compound **43** ... 47
Figure S52. ^{11}B spectrum of compound **11** ... 48
Figure S53. ^{1}H and ^{13}C spectra of compound **44** ... 49
Figure S54. ^{11}B spectrum of compound **44** ... 50
Figure S55. ^{1}H and ^{13}C spectra of compound **12** ... 51
Figure S56. ^{11}B spectrum of compound **12** ... 52
Figure S57. ^{1}H and ^{13}C spectra of compound **45** ... 53
Figure S58. ^{11}B spectrum of compound **46** ... 54
Figure S59. ^{1}H and ^{13}C spectra of compound **13** ... 55
Figure S60. ^{11}B spectrum of compound **13** ... 56
HPLC TRACES ... 57
Figure S61. HPLC trace of compound **1** ... 57
Figure S62. HPLC trace of compound **2** ... 58
Figure S63. HPLC trace of compound **3** ... 58
Figure S64. HPLC trace of compound **4** ... 58

Figure S65. HPLC trace of compound **5.** t_R = 3.3 min, 100 % (B/A: 30/70 → 100/0 in 14 min, flow 1.2 ml/min, 254 nm). 59
Figure S66. HPLC trace of compound **6** 59
Figure S67. HPLC trace of compound **7** 60
Figure S68. HPLC trace of compound **8** 60
Figure S69. HPLC trace of compound **9** 61
Figure S70. HPLC trace of compound **10** 61
Figure S71. HPLC trace of compound **11** 62
Figure S72. HPLC trace of compound **12** 62
Figure S73. HPLC trace of compound **13** 63
NOTES 63
ABBREVIATIONS 63
REFERENCE 64

SUPPLEMENTARY FIGURES AND SCHEMES

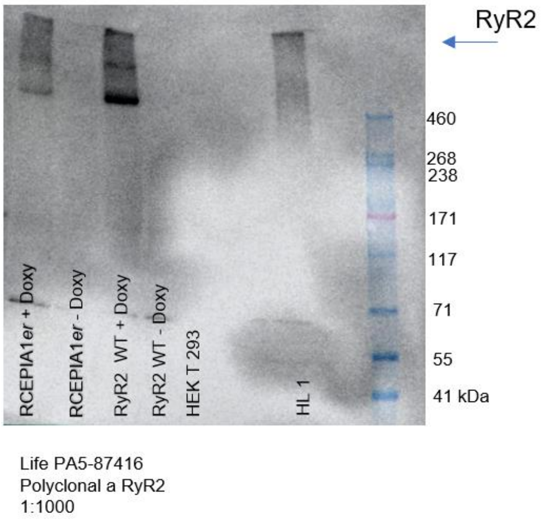

Figure S1. Western blot analysis of HEK293 and HL-1 cells. HEK293 RyR2 R-CEPIA1er and HEK293 RyR2 WT cells were analyzed before and after doxycycline treatment (Doxy – doxycycline).

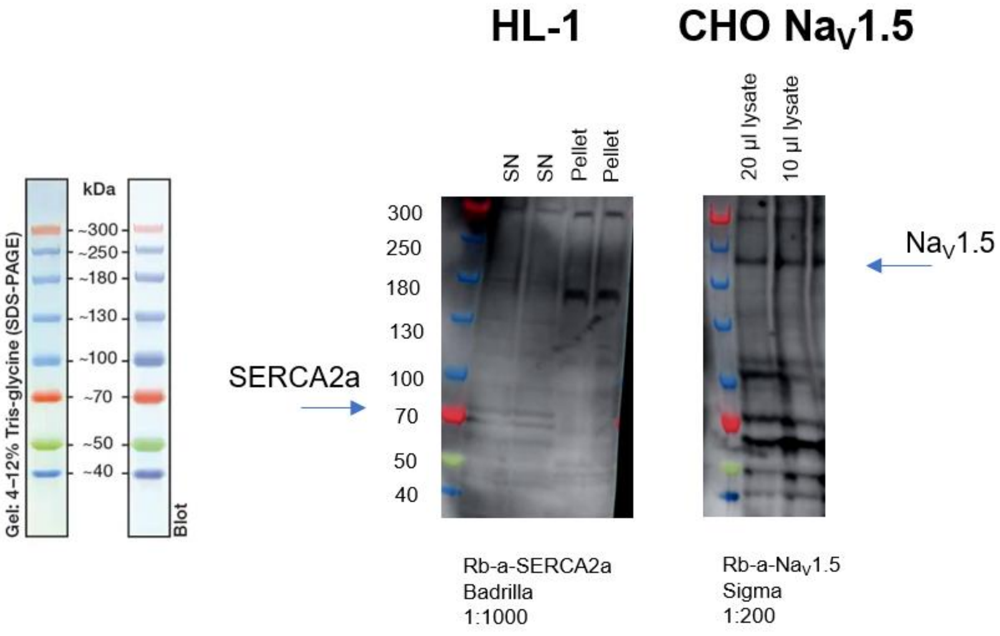

Figure S2. Protein expression patterns. Western blots using anti-SERCA2a and anti-$Na_V1.5$.

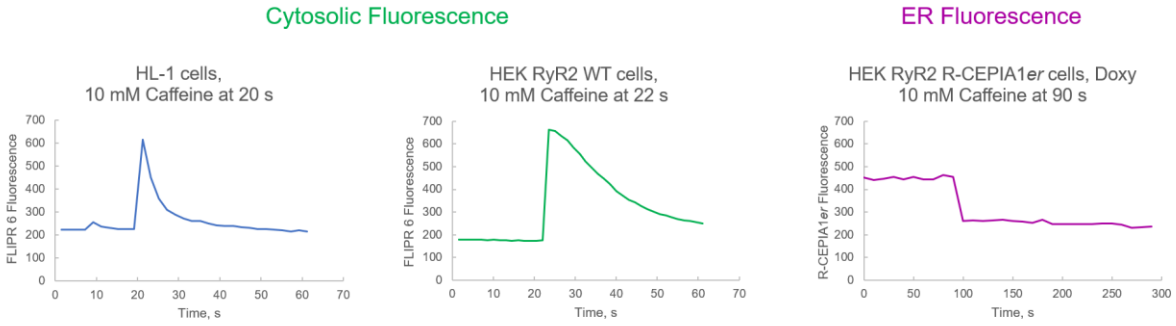

Figure S3. Addition of 10 mM caffeine to HL-1, HEK RyR2 WT and HEK RyR2 R-CEPIA1er cells.

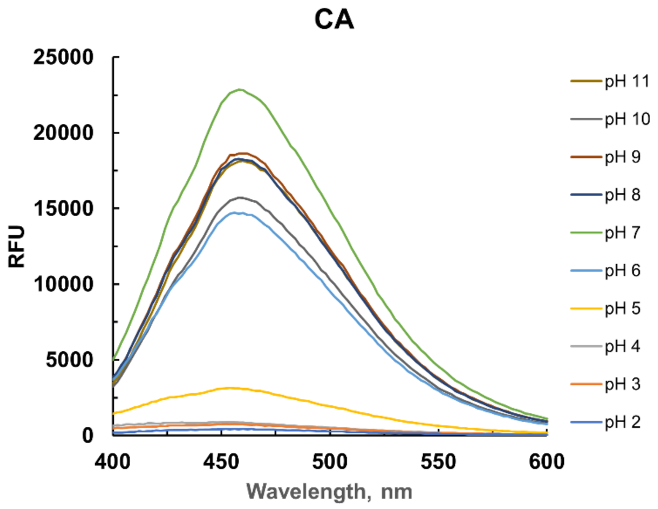

Figure S4. Fluorescence of CA (33 μM) at various pH.

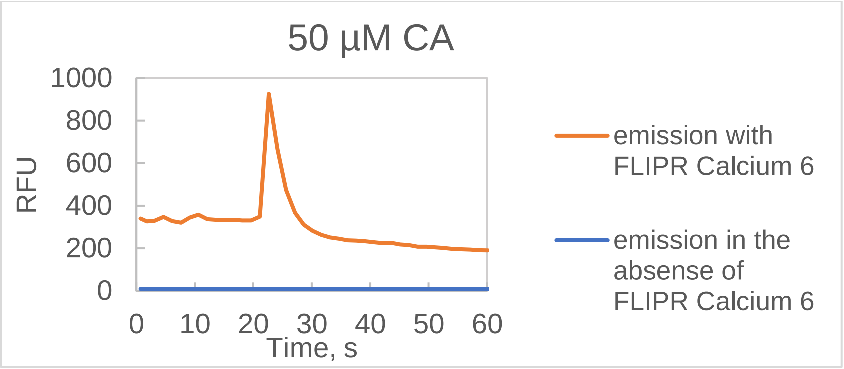

Figure S5. CA fluorescence does not affect Caffeine assay results.

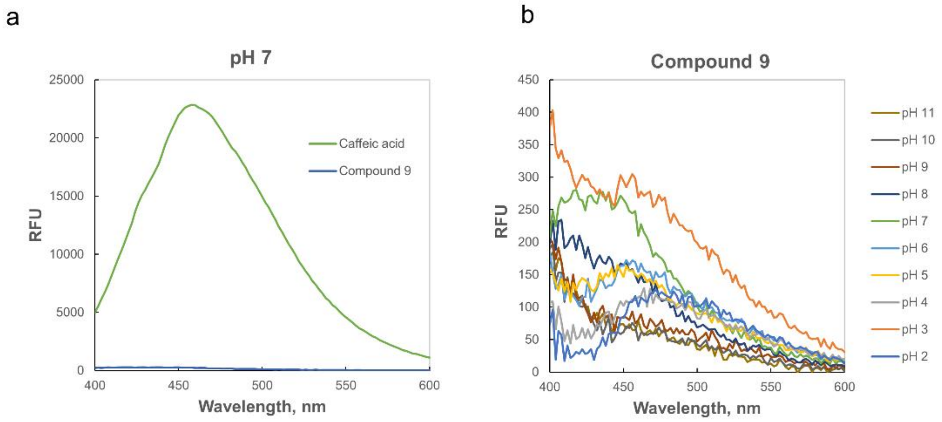

Figure S6. (a) Comparison of the fluorescence properties of CA and compound 9 (33 μM). (b) Fluorescence of 9 (33 μM) at various pH.

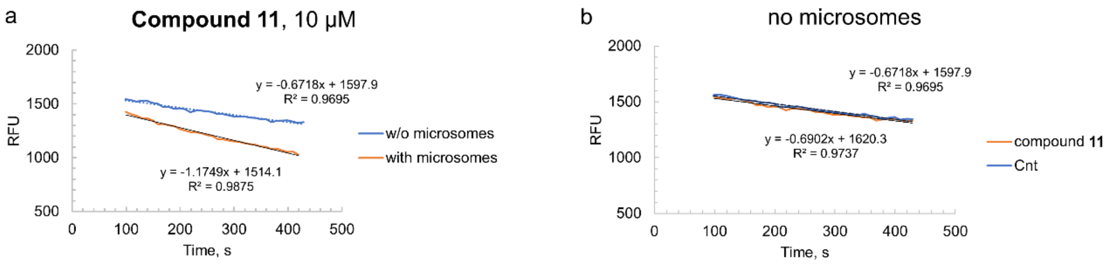

Figure S7. Plots of NADH consumption (a) in the presence of compound 11 (10 μM) with and without mouse cardiac vesicles; (b) with and without compound **11** (no microsomes). Cnt – control sample (0.1 v/v% DMSO).

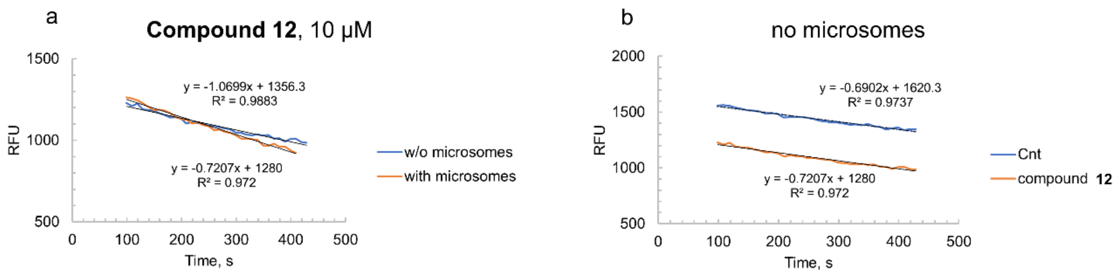

Figure S8. Plots of NADH consumption (a) in the presence of compound **12** (10 µM) with and without mouse cardiac vesicles; (b) with and without compound **12** (no microsomes). Cnt – control sample (0.1 v/v% DMSO).

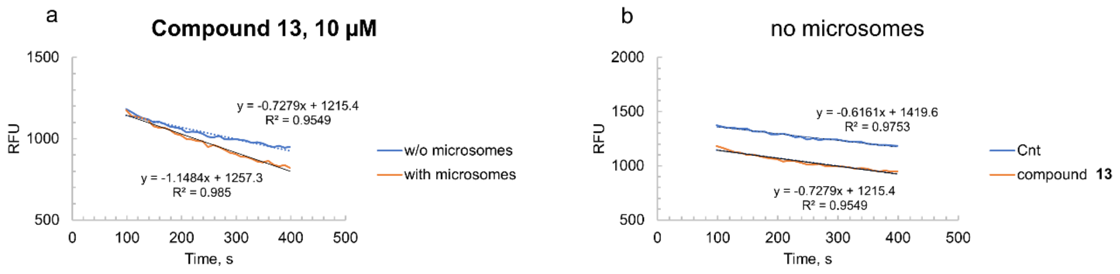

Figure S9. Plots of NADH consumption (a) in the presence of compound **13** (10 µM) with and without mouse cardiac vesicles; (b) with and without compound **13** (no mouse SR). Cnt – control sample (0.1 v/v% DMSO).

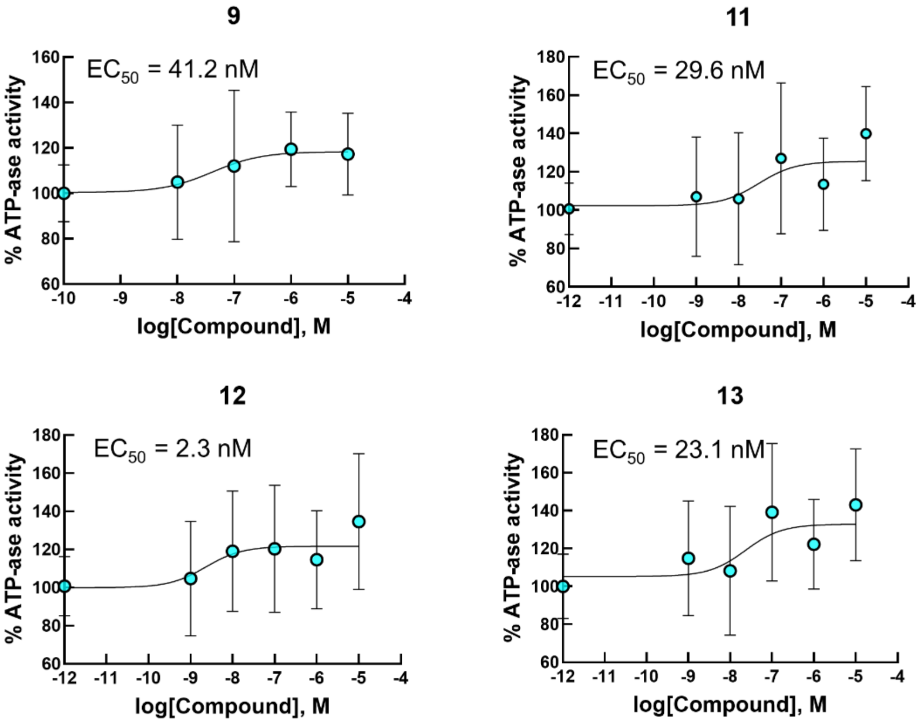

Figure S10. Compounds **9**, **11** - **13** evoke enhancements in the kinetic rate of NADH - NAD^+ conversion in a dose-dependent manner. 200 µM free Ca^{2+} concentration was used. The effects of compounds are expressed as a percentage of the control effect. Data represented as mean ± S.D., n = 9 – 24/ 5 - 6 mice.

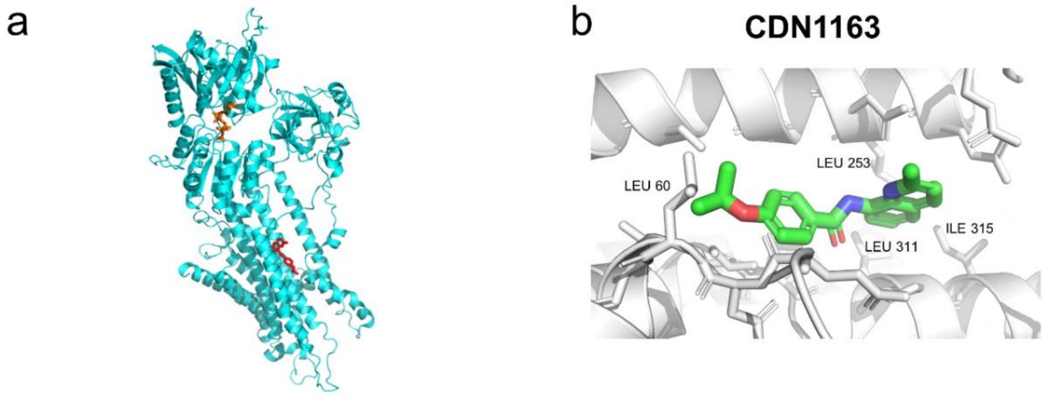

Figure S11. (a) CDN1163 (red) in the human E2.ATP SERCA2a (pdb 7BT2).[1] ATP molecule in the N domain of SERCA2a is indicated in orange. Molecular docking was done using DiffDock-L (Neurosnap Inc., Delaware, USA). DiffDock Confidences are moderate: -0.65. The SMINA Minimized Affinitiy of -7.18 kcal/mol indicates that the potential ligand-protein complexes are stable. The RMSDs of 1.08 Å meets the criterion for the accurate prediction. Data visualization was done using Pymol, Molecular Graphics System, Version 3.1.2 (Schrödinger, LLC, New York, USA). (b) Predicted binding modes of CDN1163. Carbon atoms are indicated in gray (for SERCA2a) or green (for compounds), nitrogen atoms are in blue and oxygens in red.

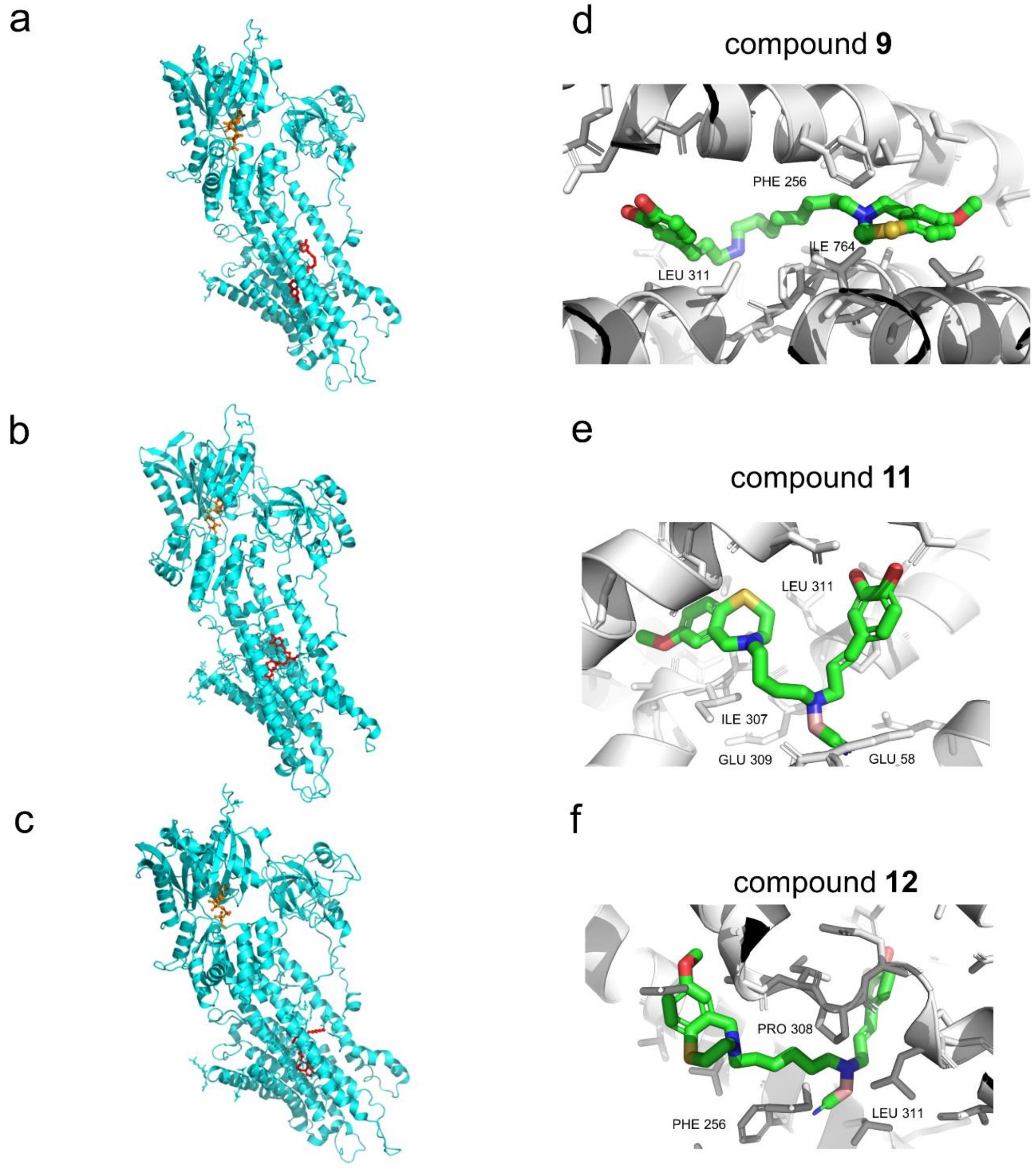

Figure S12. (a, b, c) Compounds **9**, **11** and **12** (red) in the human E2 SERCA2a (pdb 7BT2).[1] Molecular docking was done using DiffDock-L (Neurosnap Inc., Delaware, USA). DiffDock Confidences are moderate: -0.64 for compound **9**, -0.99 for compound **11** and -1.2 for compound **12**. The RMSDs for compounds **9** (1.99), **11** (1.45), **12** (1.56) meet the criterion for the accurate prediction. The SMINA Minimized Affinities of -7.49 kcal/mol (compound **9**) and -7.45 kcal/mol (com-pound **11**) indicate that the potential ligand-protein complexes are stable. The calculated SMINA Minimized Affinity for the compound **12** in SERCA2a (-8.15 kcal/mol) show that this complex is highly stable. Data visualization was done using Pymol, Molecular Graphics System, Version 3.1.2 (Schrödinger, LLC, New York, USA). (d, e, f) Predicted binding modes of compounds **9**, **11** and **12**. Carbon atoms are indicated in gray (for SERCA2a) or green (for compounds), nitrogen atoms are in blue, oxygens in red and sulfur in yellow.

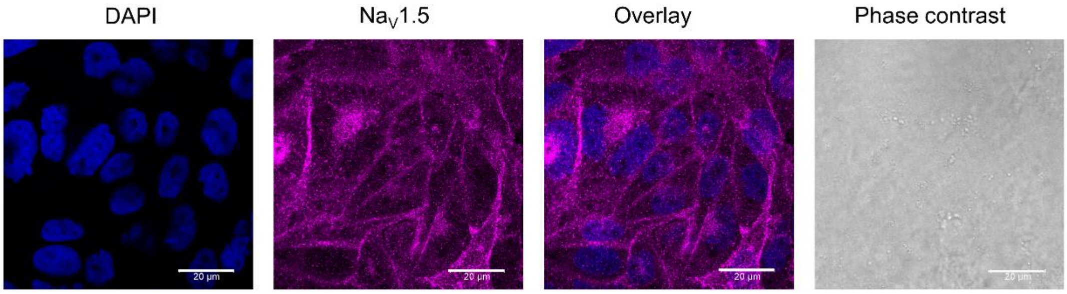

Figure S13. Confocal microscopy images of CHO $Na_V1.5$ Duo cells stained with DAPI (blue) and anti-$Na_V1.5$ antibodies (magenta). Scale bar: 20 µm. The cells were imaged on confocal Leica SP8 microscope as described in the Materials and Methods section.

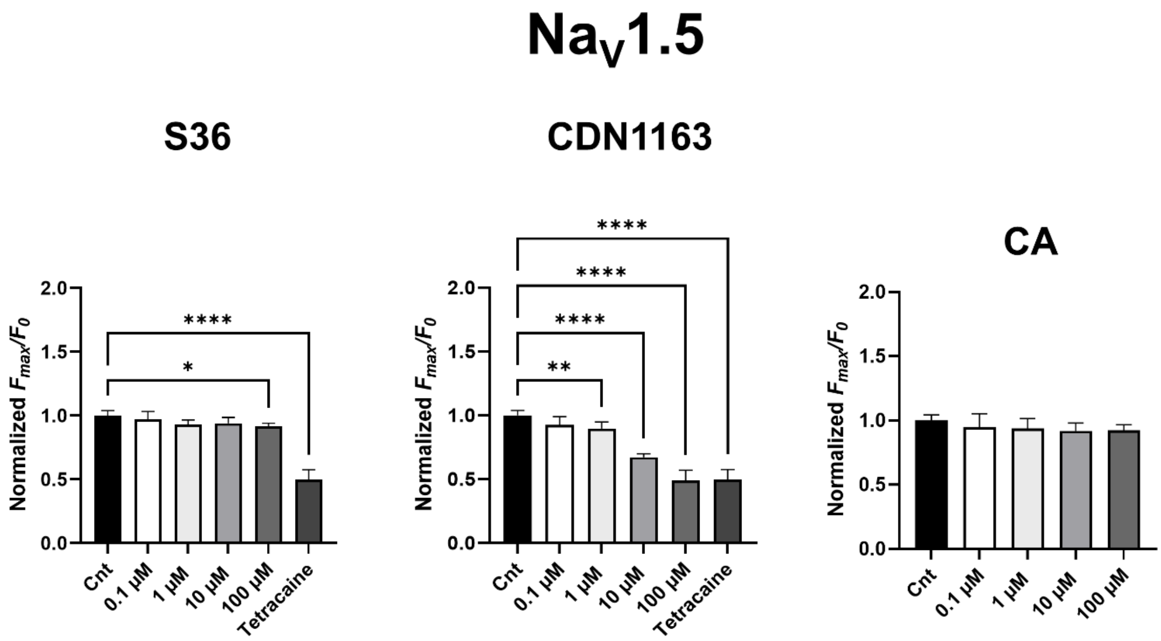

Figure S14. FLIPR membrane potential assay on CHO $Na_V1.5$ Duo cells. The data were normalized to the control (Cnt, 0.1 v/v% DMSO). Values represent the mean ± S.D., * $P < 0.05$, ** $P < 0.005$, **** $P < 0.0001$ vs. control by one-way ANOVA test, n = 3 - 6.

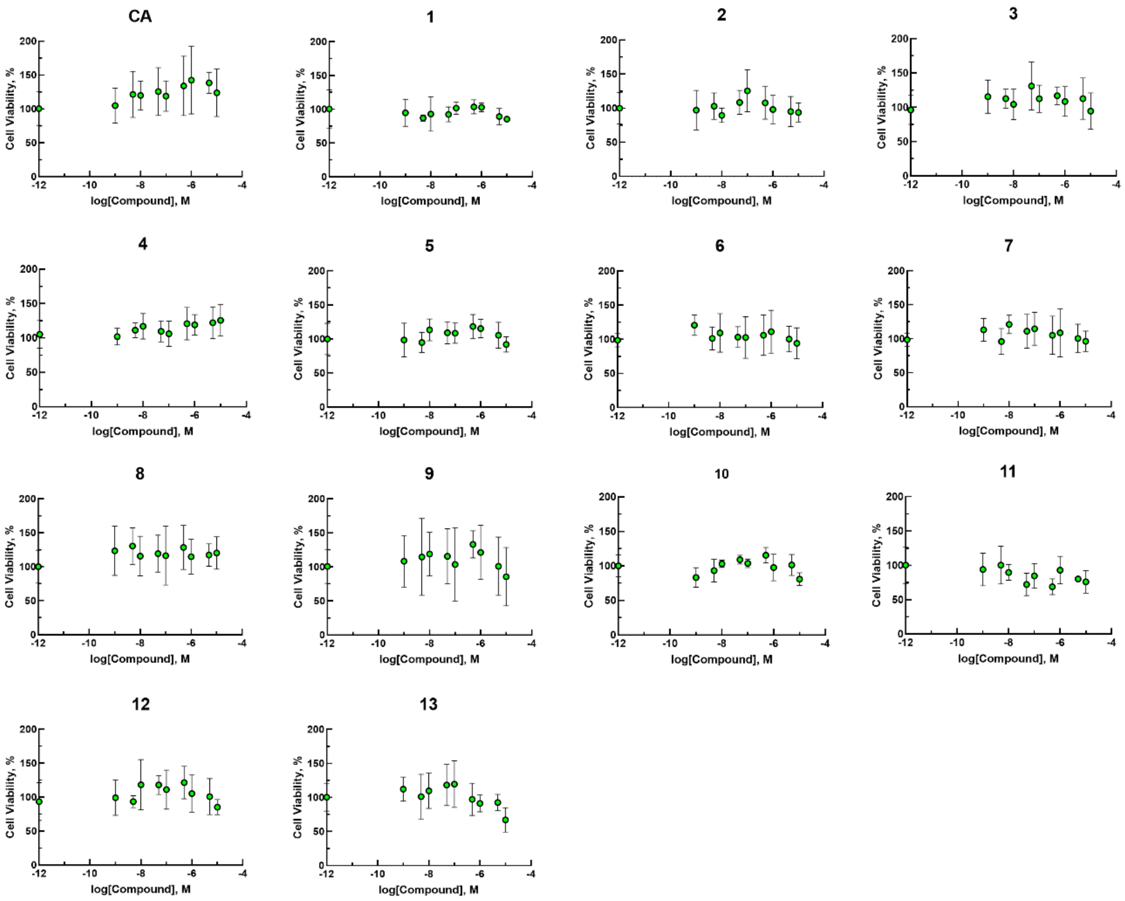

Figure S15. Cell viability after 24 h incubation with test compounds in the concentration range of 1 nM – 10 μM. HL cells were treated with various concentrations of the tested compounds (24 h) and assayed using CytoTox-Glo™ Cytotoxicity kit. The effect from non-treated cells (incubation in DMEM for 24 h) was taken as a 100% cell viability. Data represent as mean ± S.D., n = 3 – 6 independent measurements.

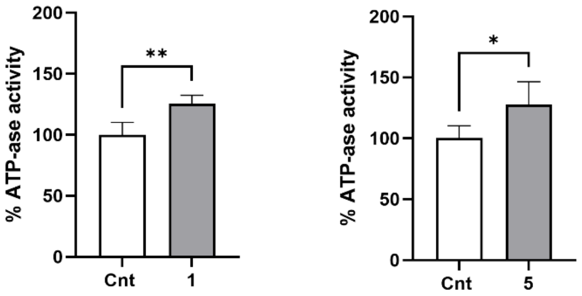

Figure S16. SERCA2 activity measurements on microsomal membrane vesicles derived from HEK-293T cells. Effect of 10 μM **1** and **5** on SERCA2 activity. The rate of ATP consumption was normalized to the 0.1 v/v% DMSO. Values represent the mean ± S.D., * $P < 0.05$, ** $P < 0.001$ vs. control (Cnt, 0.1 v/v% DMSO) by unpaired t test; n = 4.

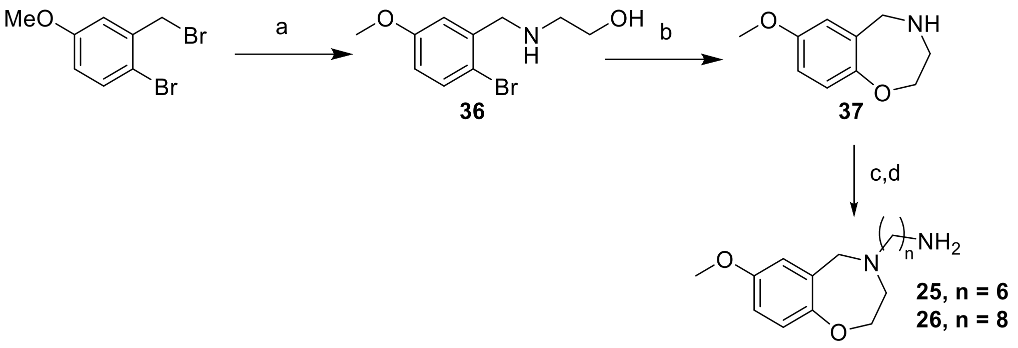

Scheme S1. Reagents and conditions: (a) 2-aminoethanol, $NaHCO_3$, THF, rt; (b) CuI, K_2CO_3, *i*PrOH, reflux; (c) *N*-(6-bromohexyl)phthalimide or *N*-(8-bromooctyl)phthalimide, K_2CO_3, 1,4-dioxane, reflux; (d) N_2H_2, EtOH, rt.

NMR SPECTRA

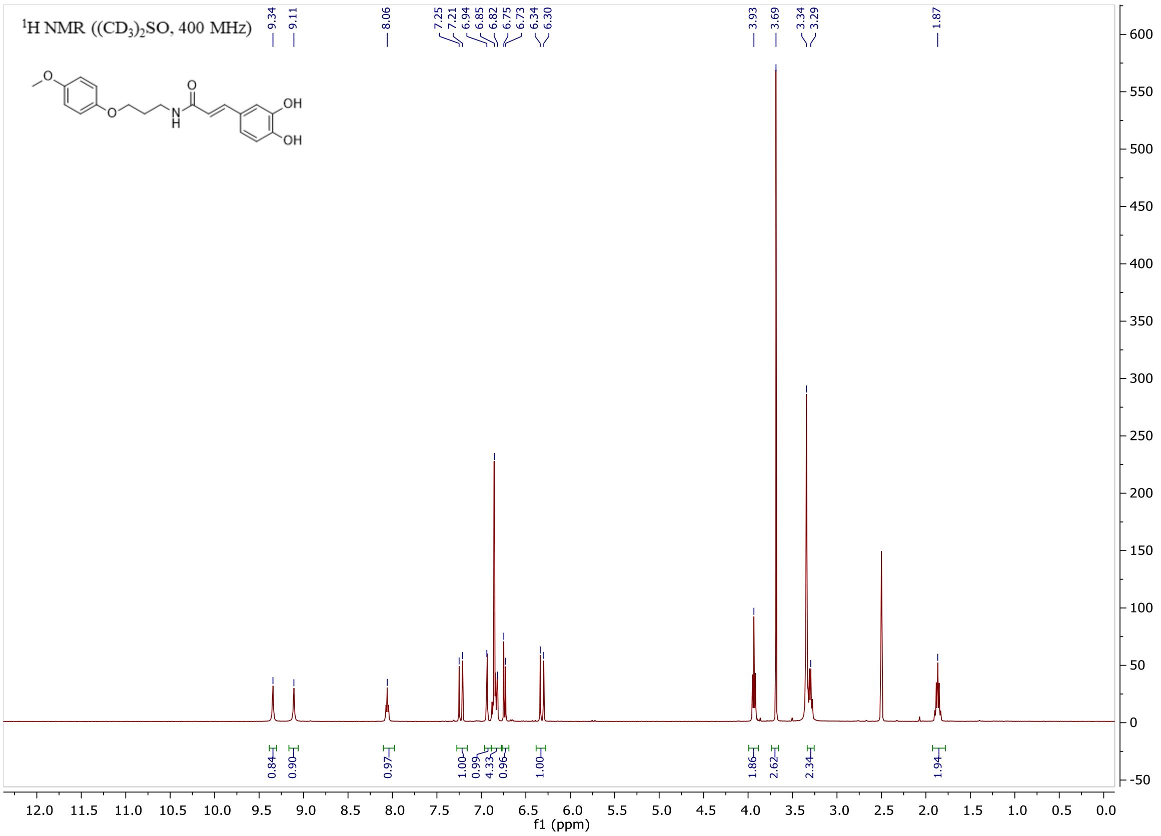

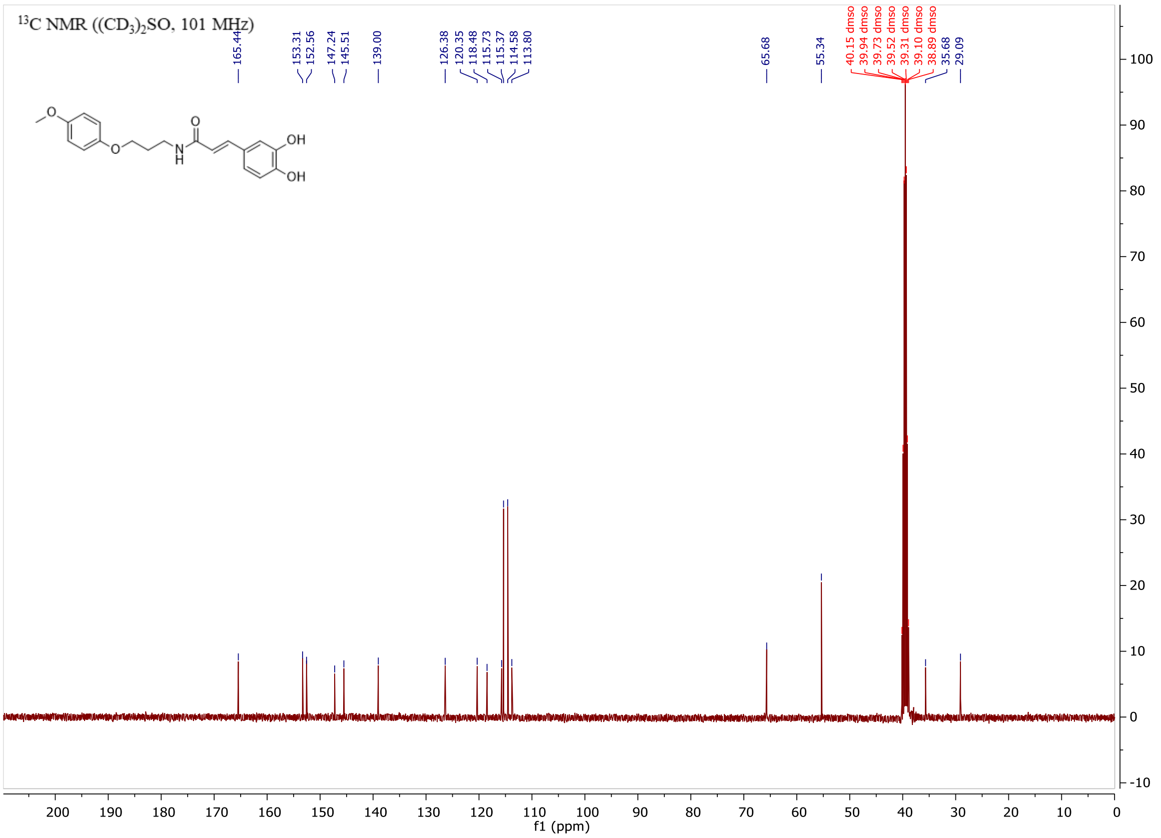

Figure S17. 1H and 13C spectra of compound **1**.

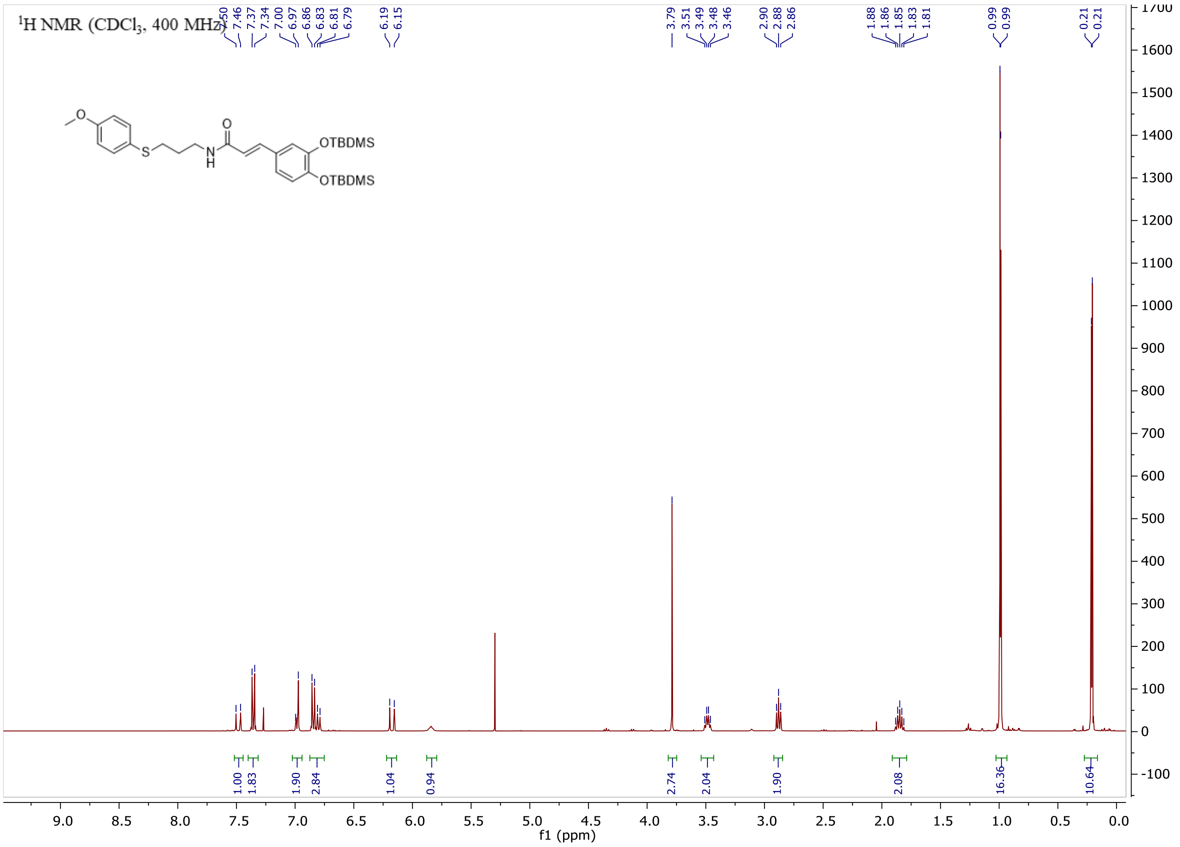

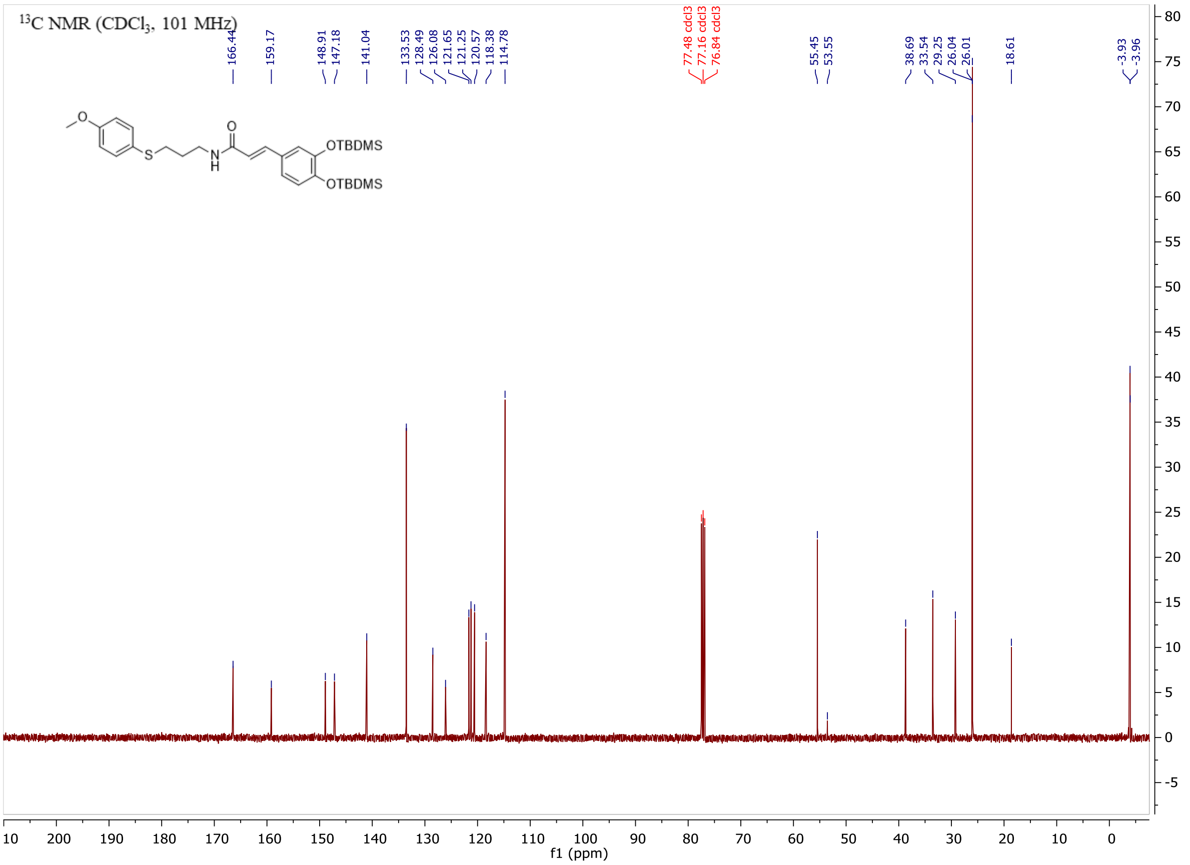

Figure S18. ¹H and ¹³C spectra of compound **28**.

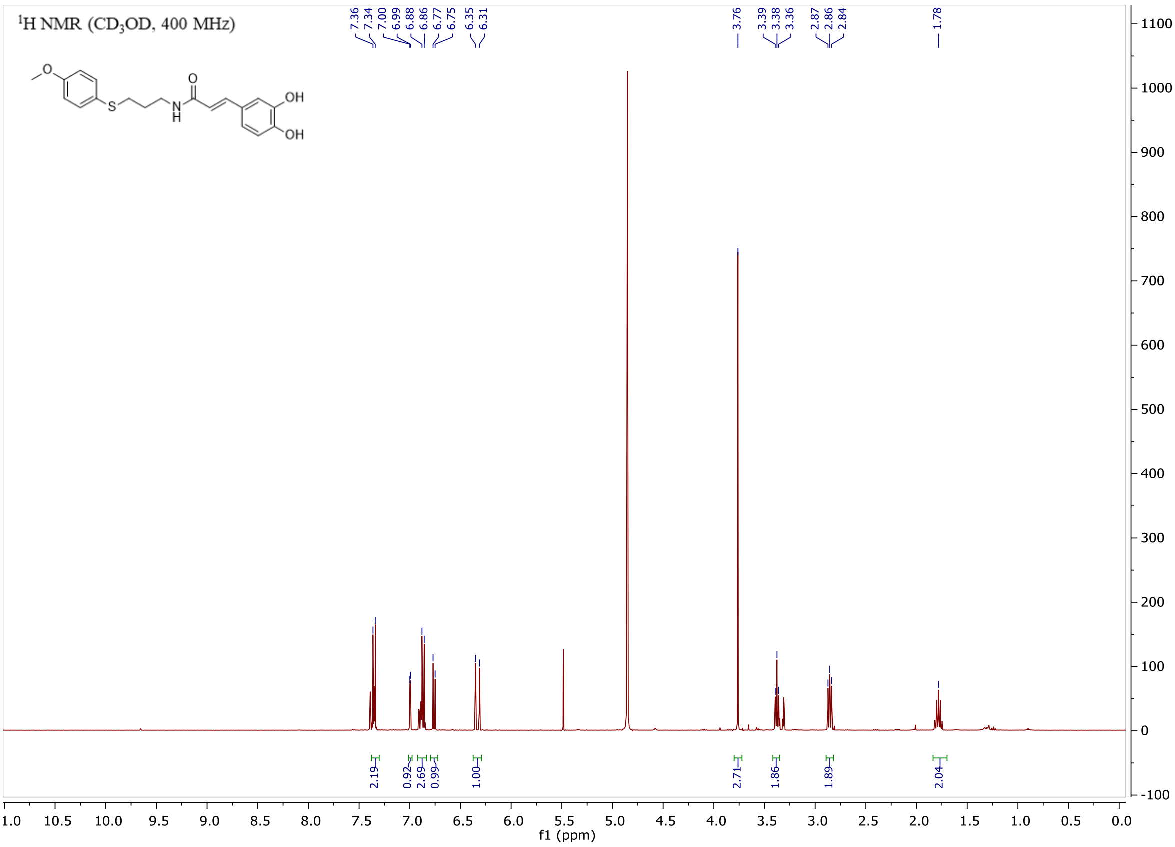

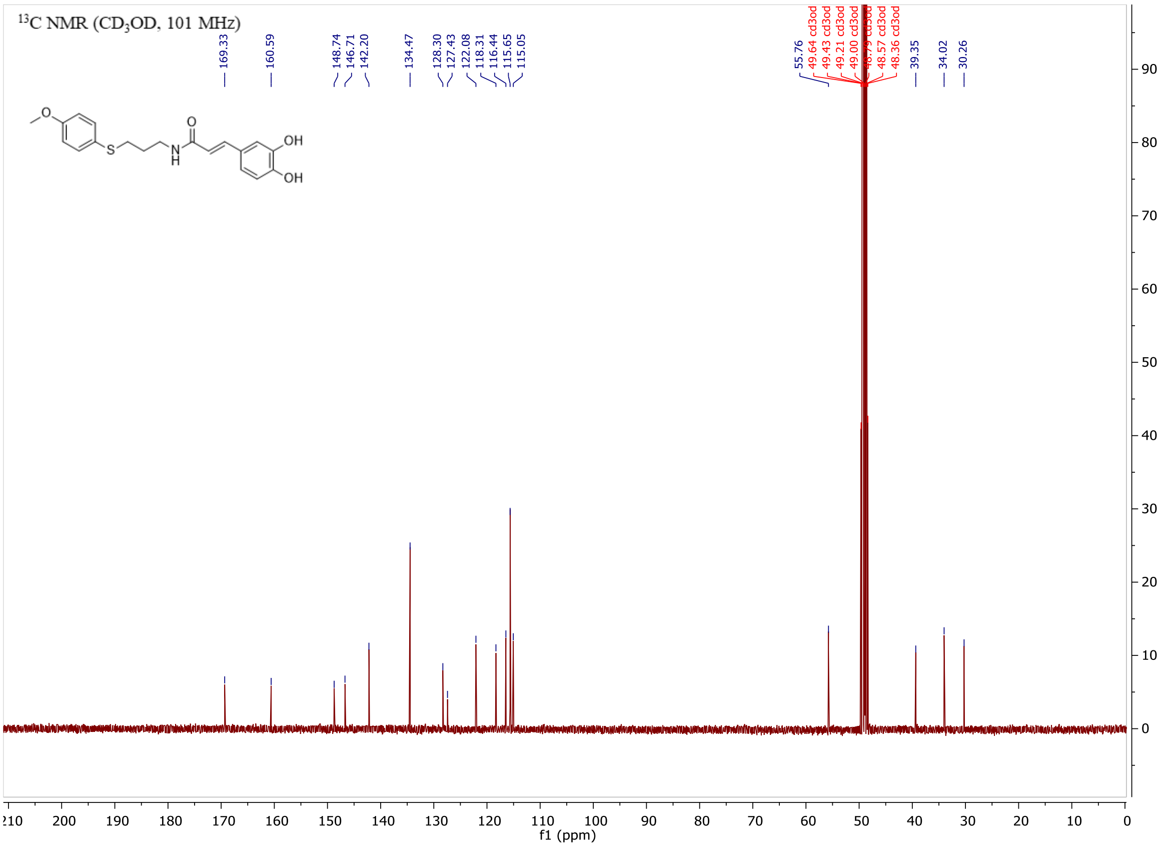

Figure S19. ¹H and ¹³C spectra of compound **2**.

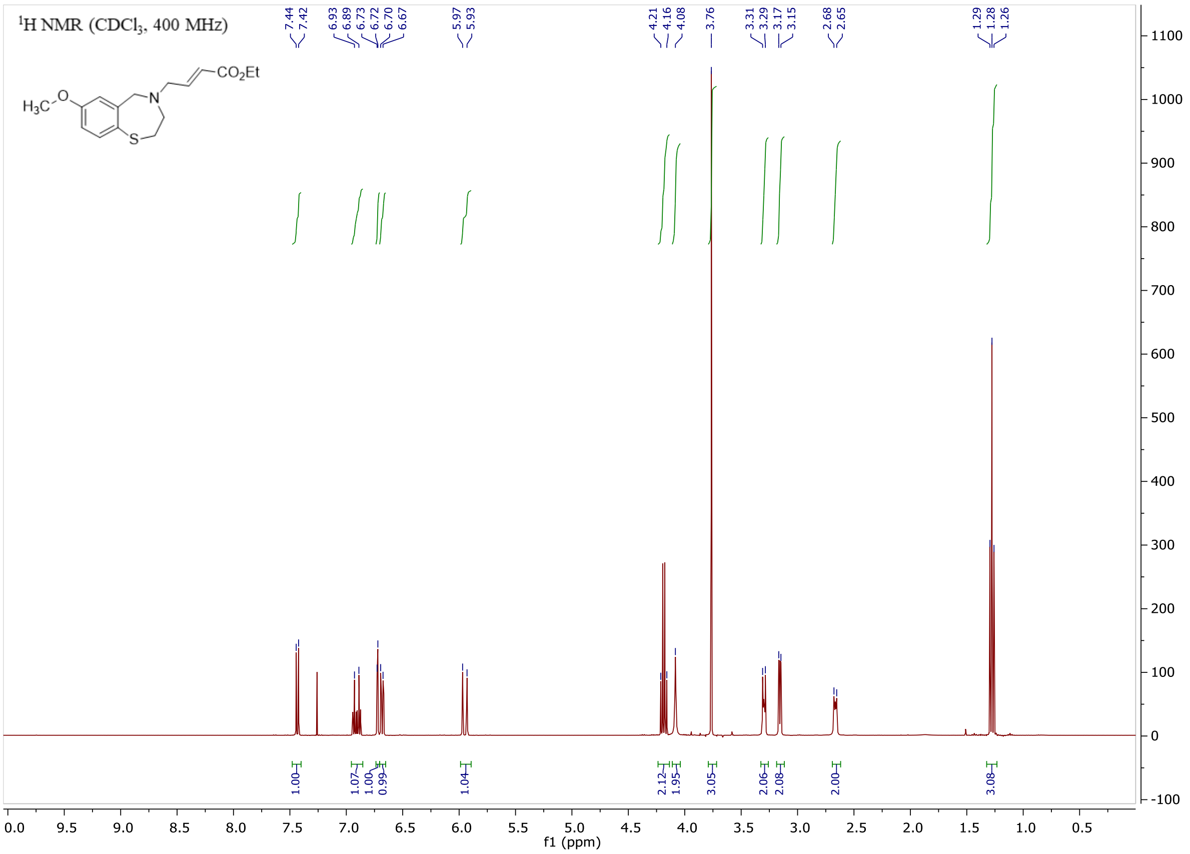

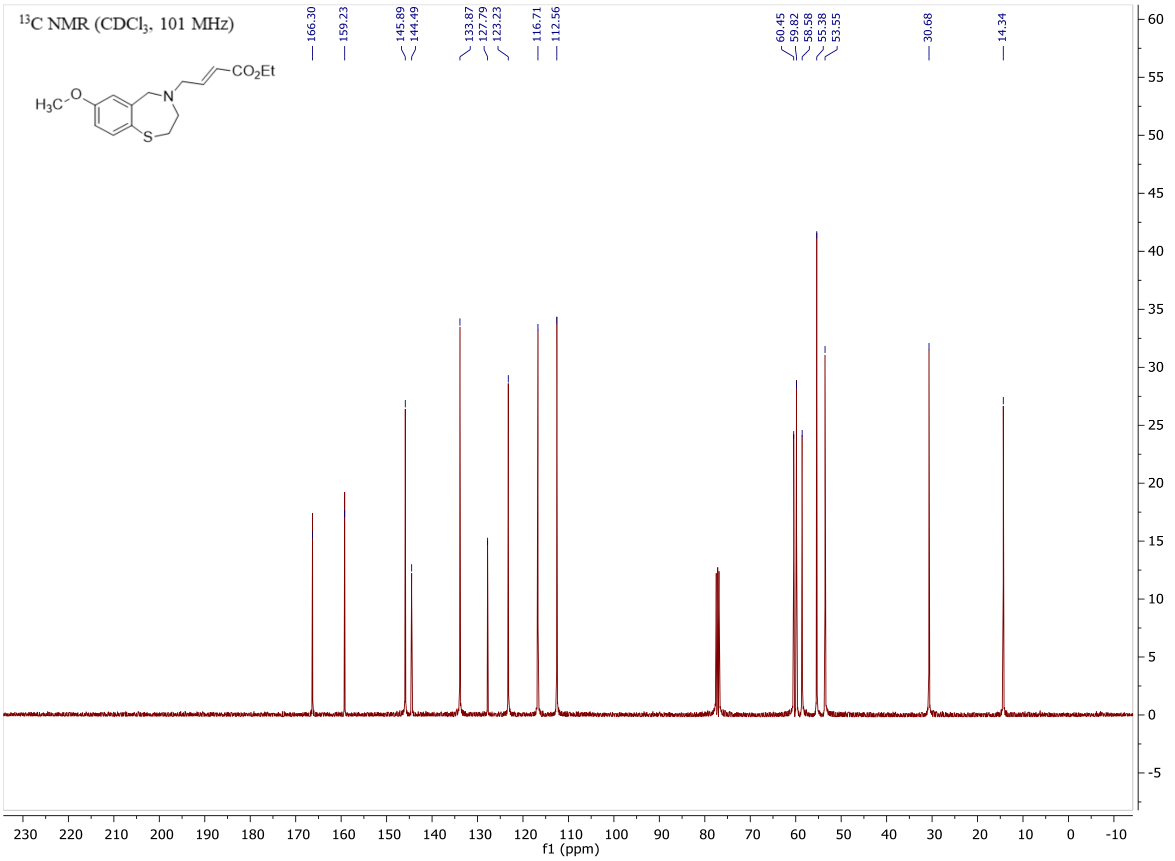

Figure S20. ^{1}H and ^{13}C spectra of compound **29**.

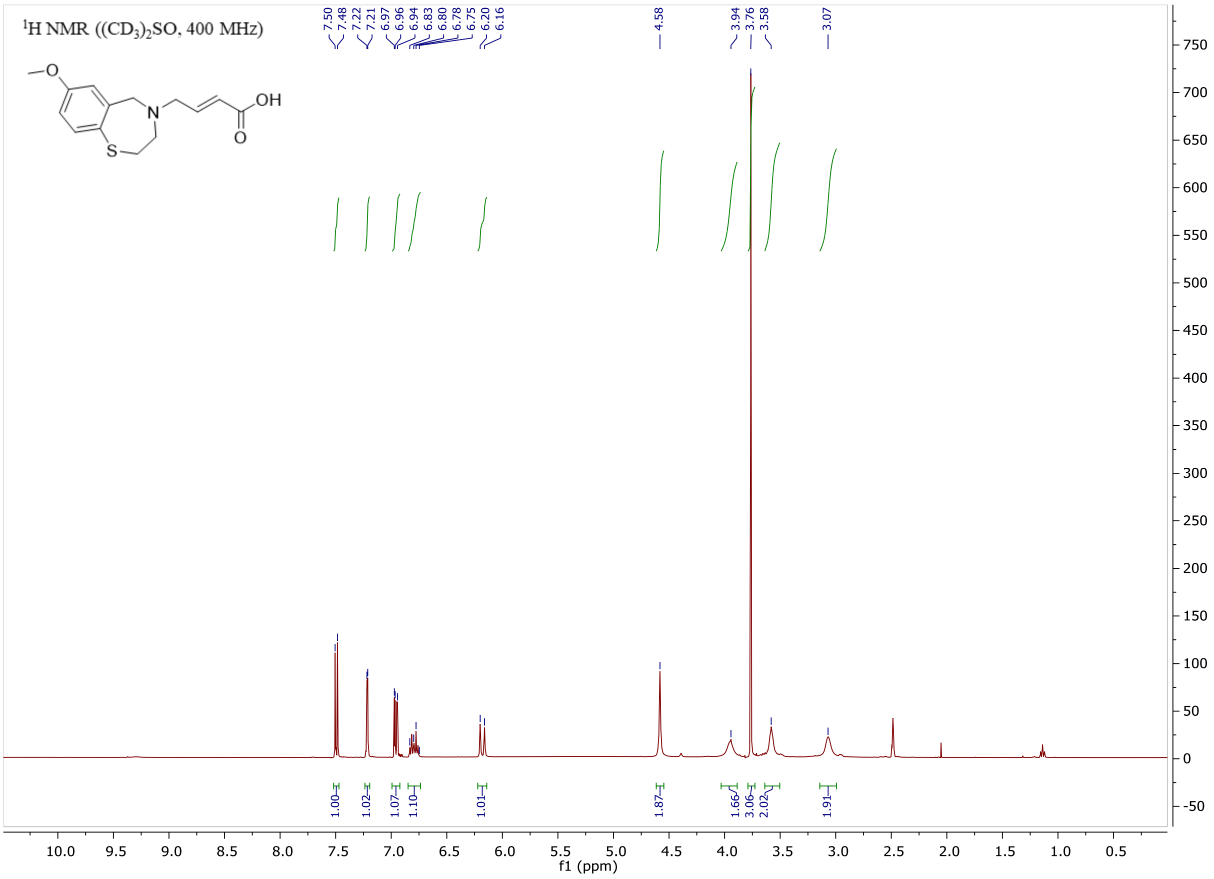

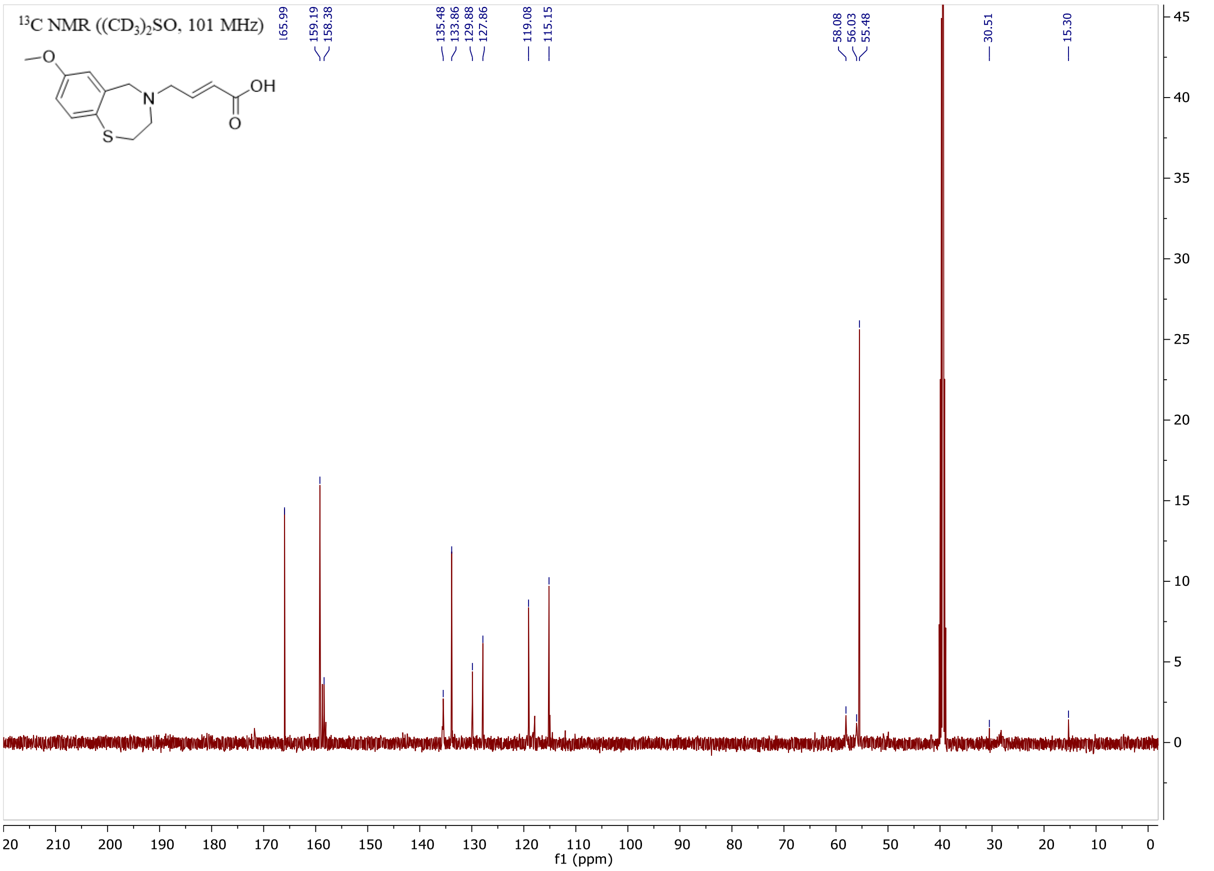

Figure S21. ¹H and ¹³C spectra of compound **17**.

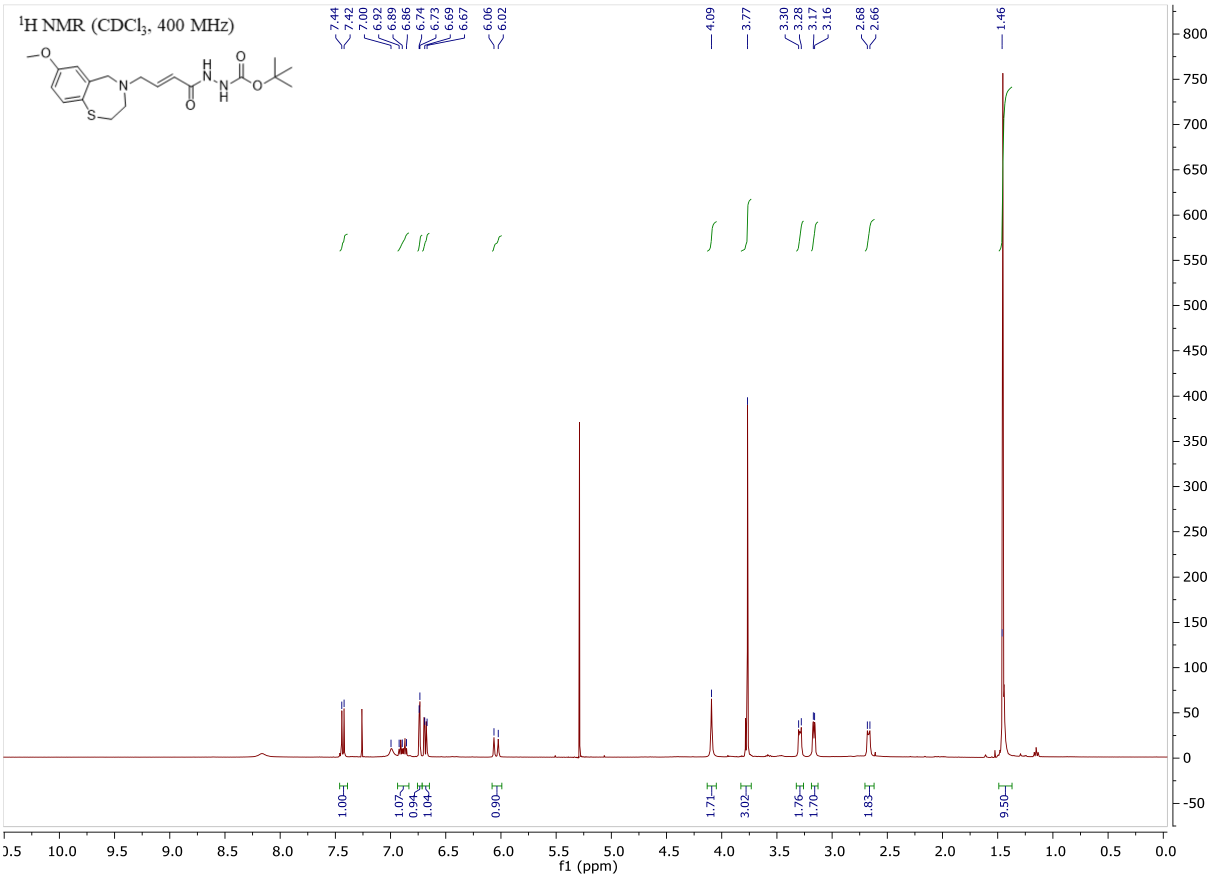

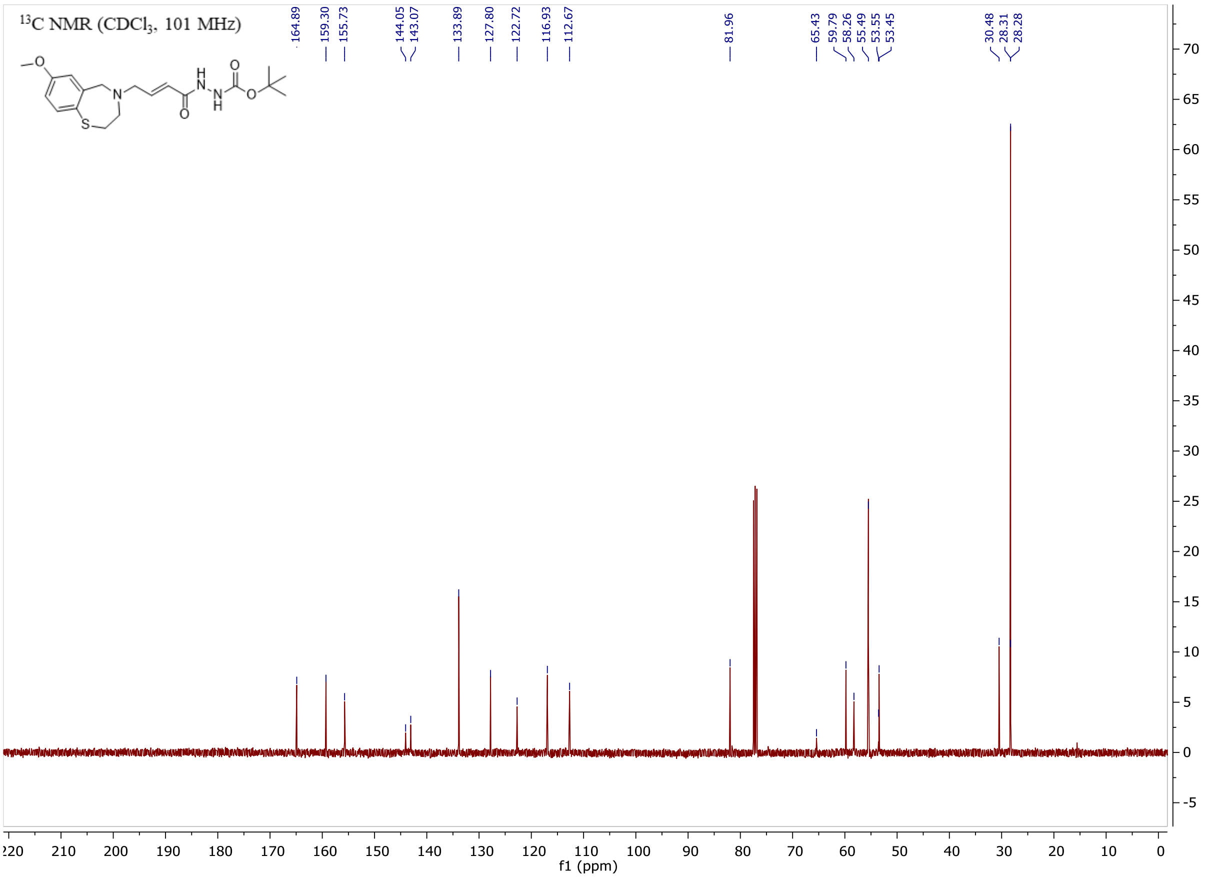

Figure S22. ^{1}H and ^{13}C spectra of compound **30**.

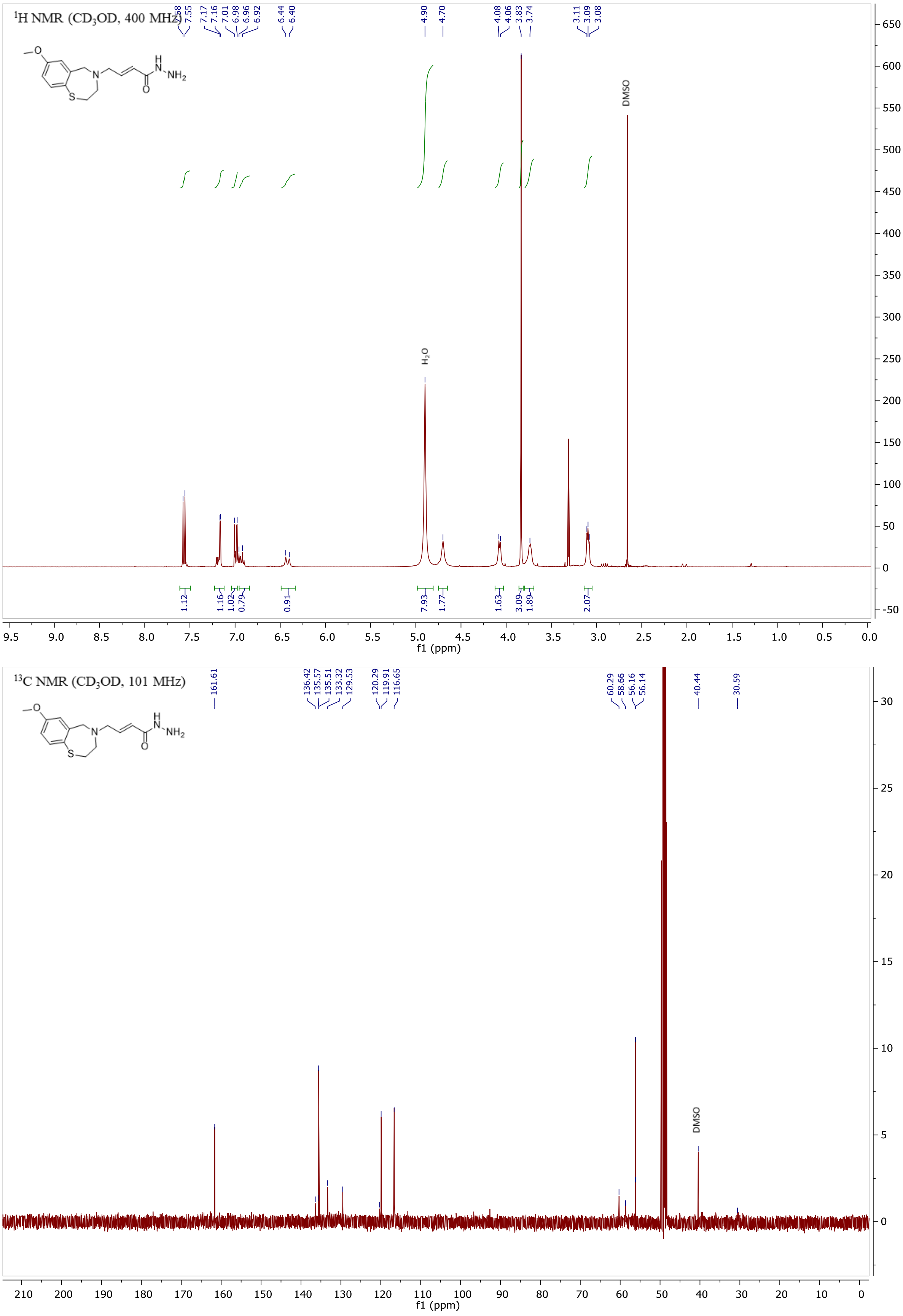

Figure S23. 1H and 13C spectra of compound **18**.

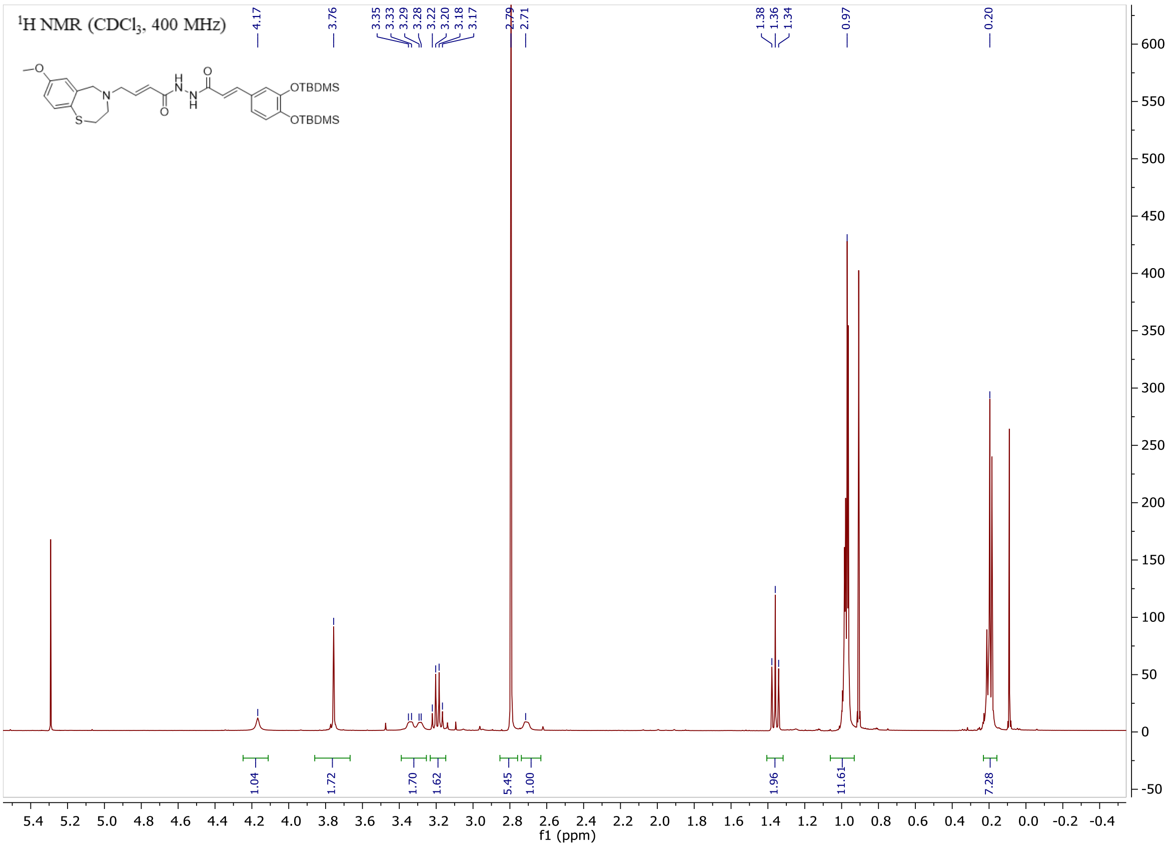

Figure S24. ¹H spectrum of compound **30**.

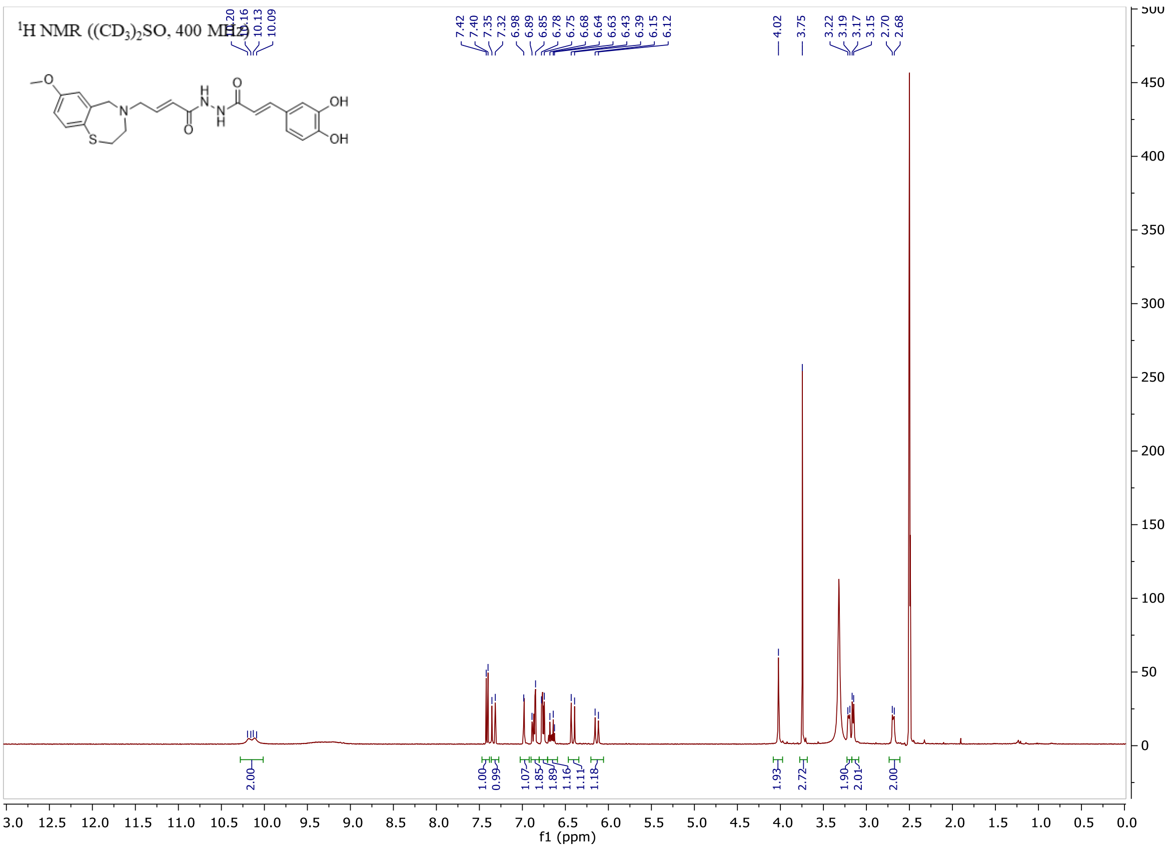

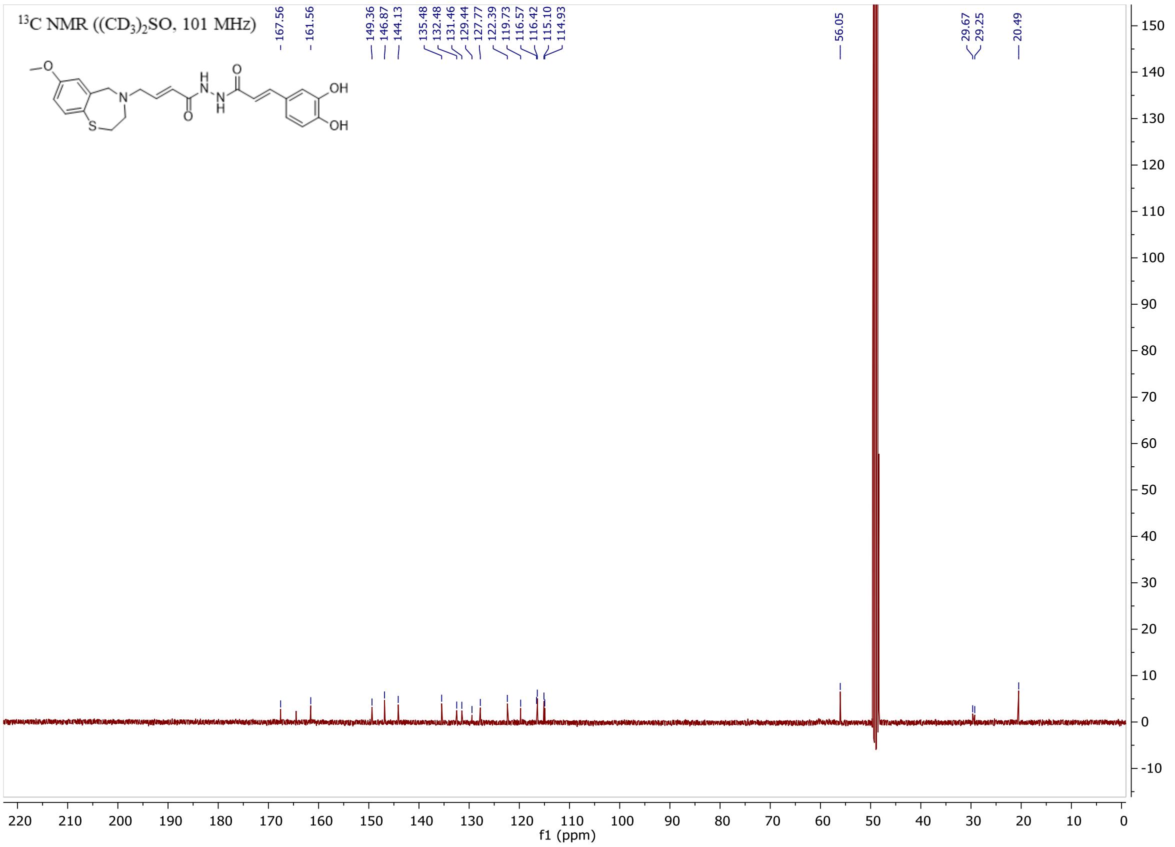

Figure S25. ¹H and ¹³C spectra of compound **3.**

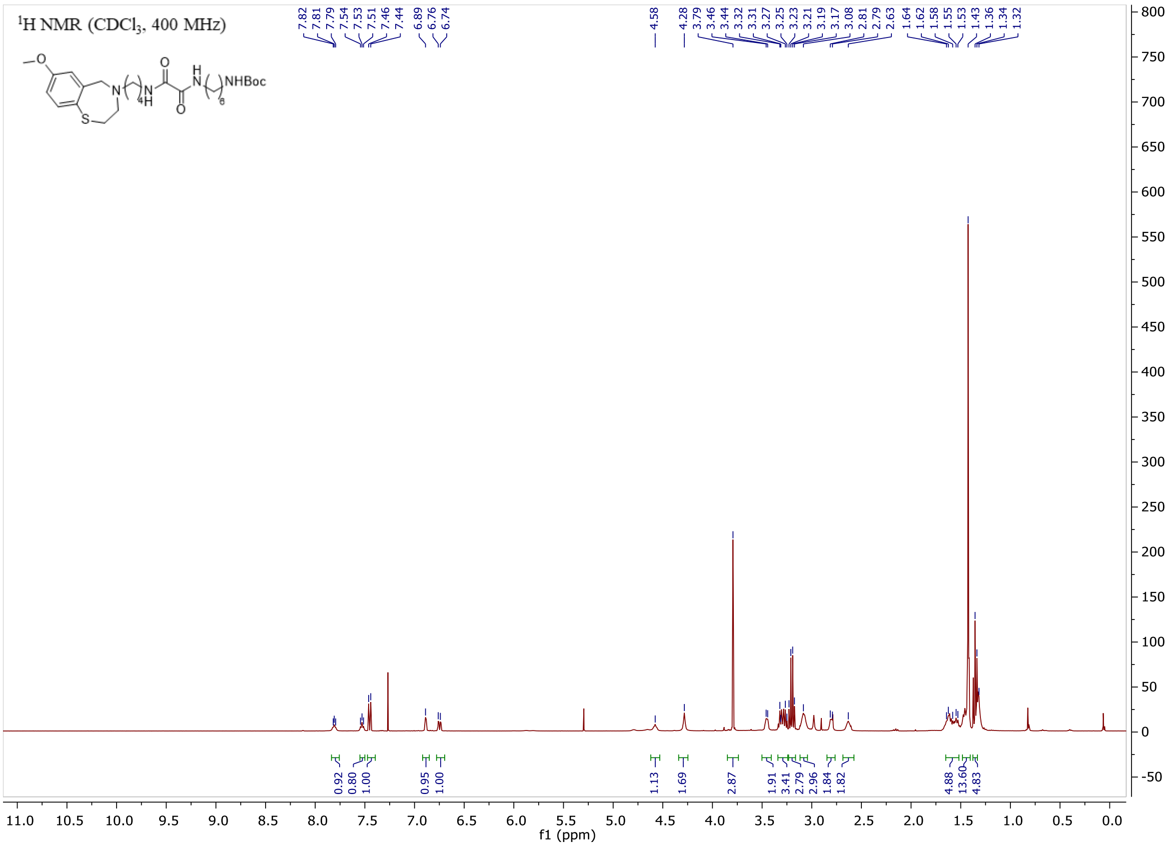

Figure S26. ^{1}H spectrum of compound **32.**

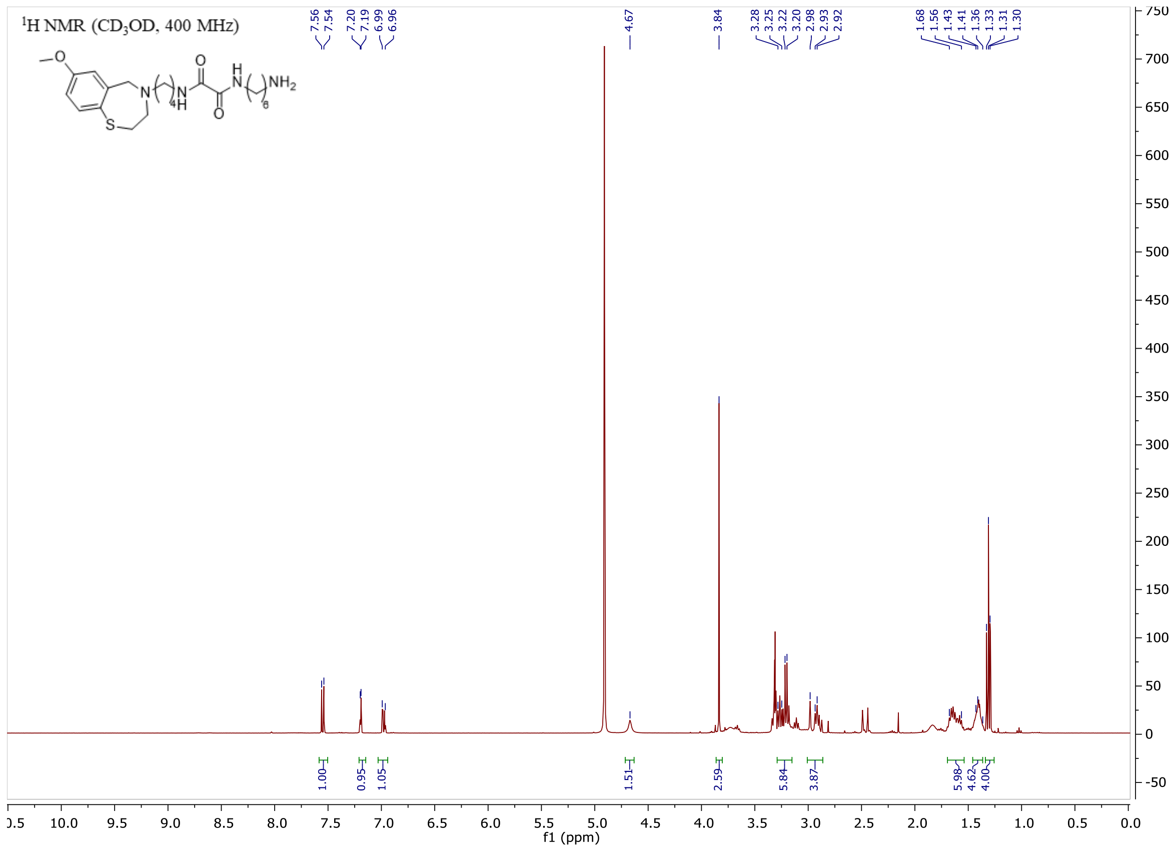

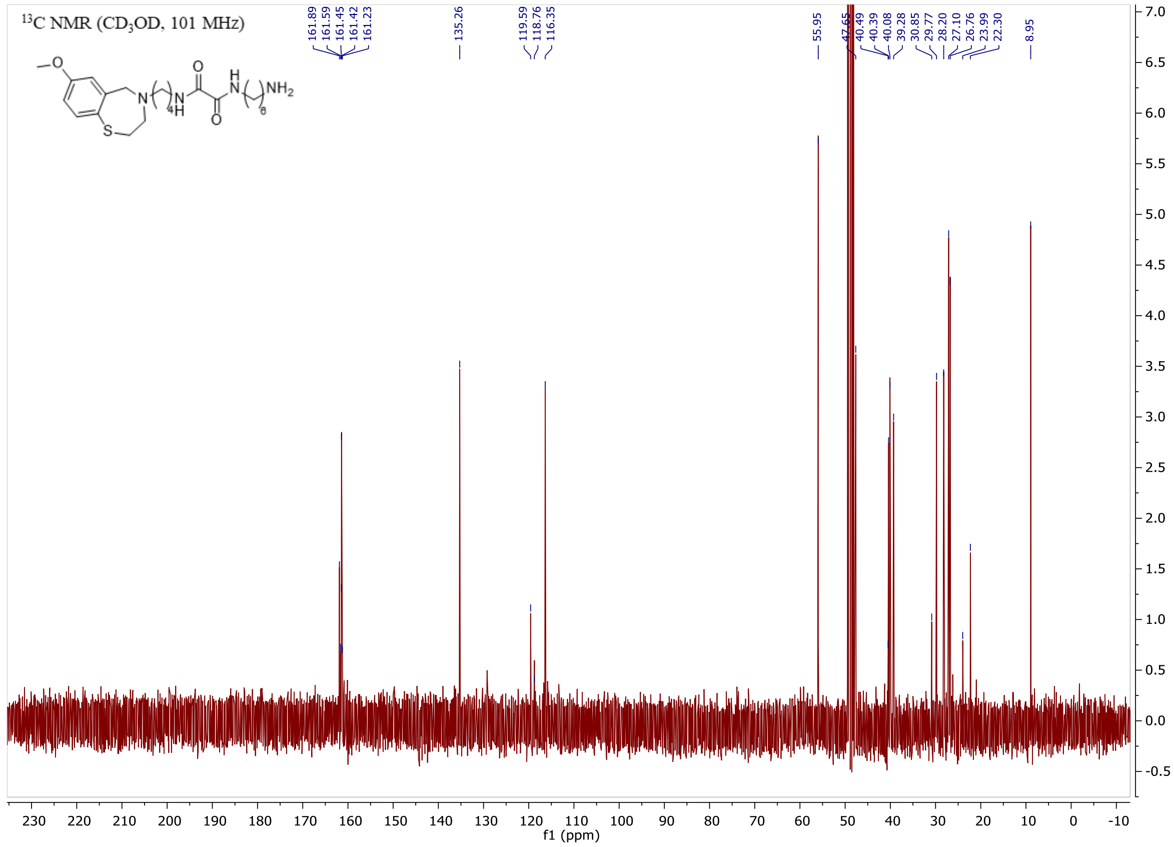

Figure S27. ^{1}H and ^{13}C spectra of compound **21.**

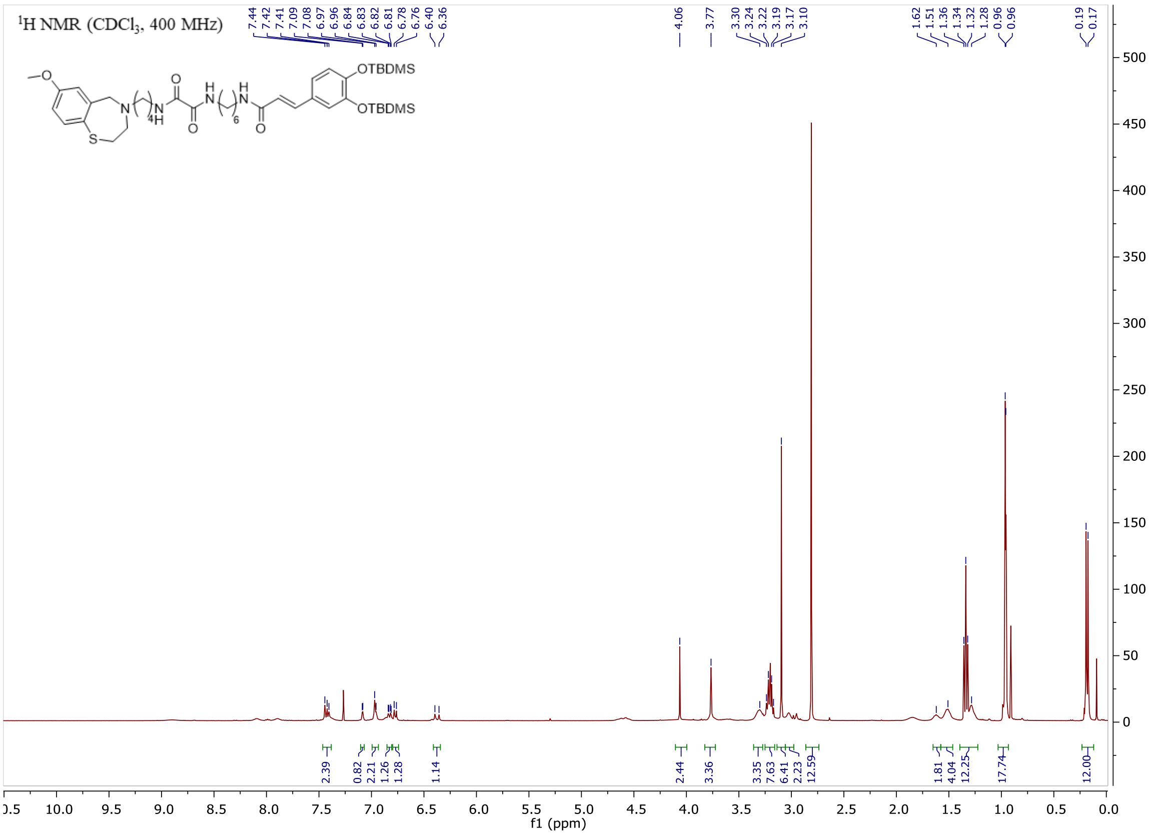

Figure S28. ^{1}H spectrum of compound **33.**

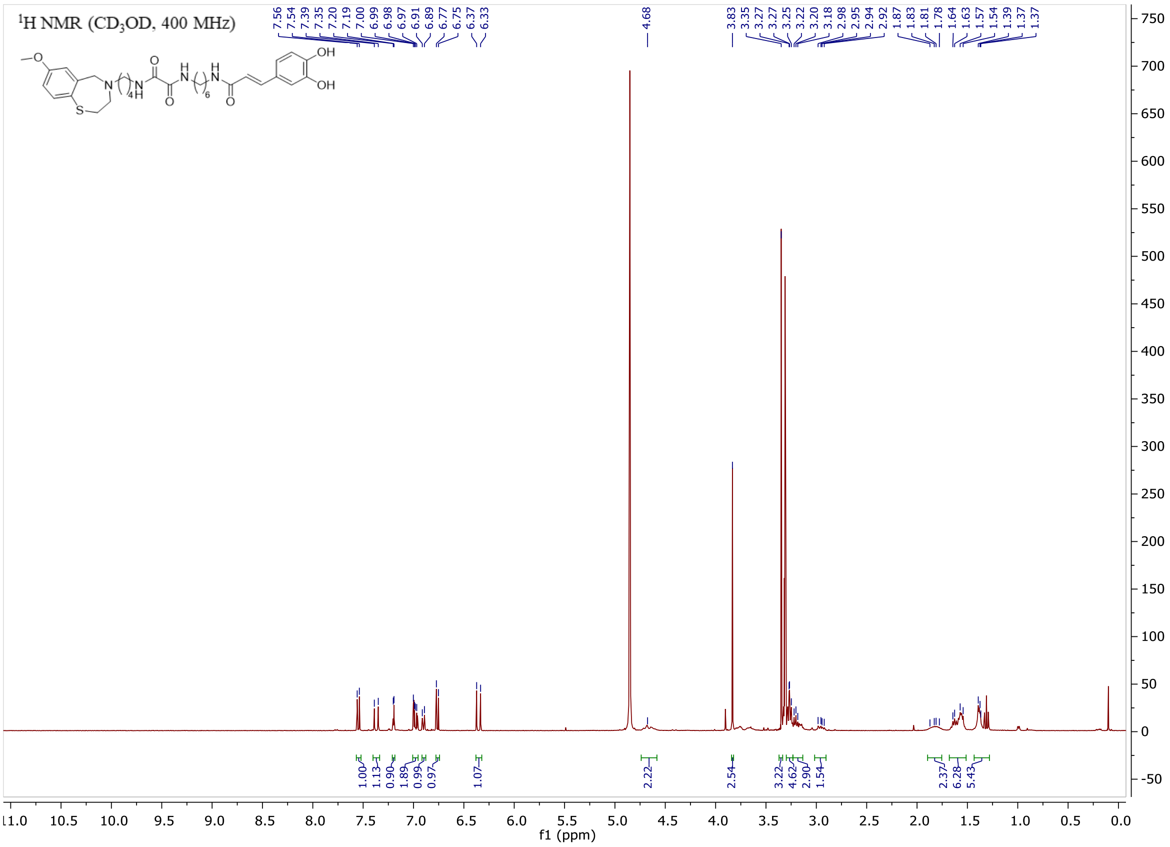

Figure S29. ^{1}H spectrum of compound **4.**

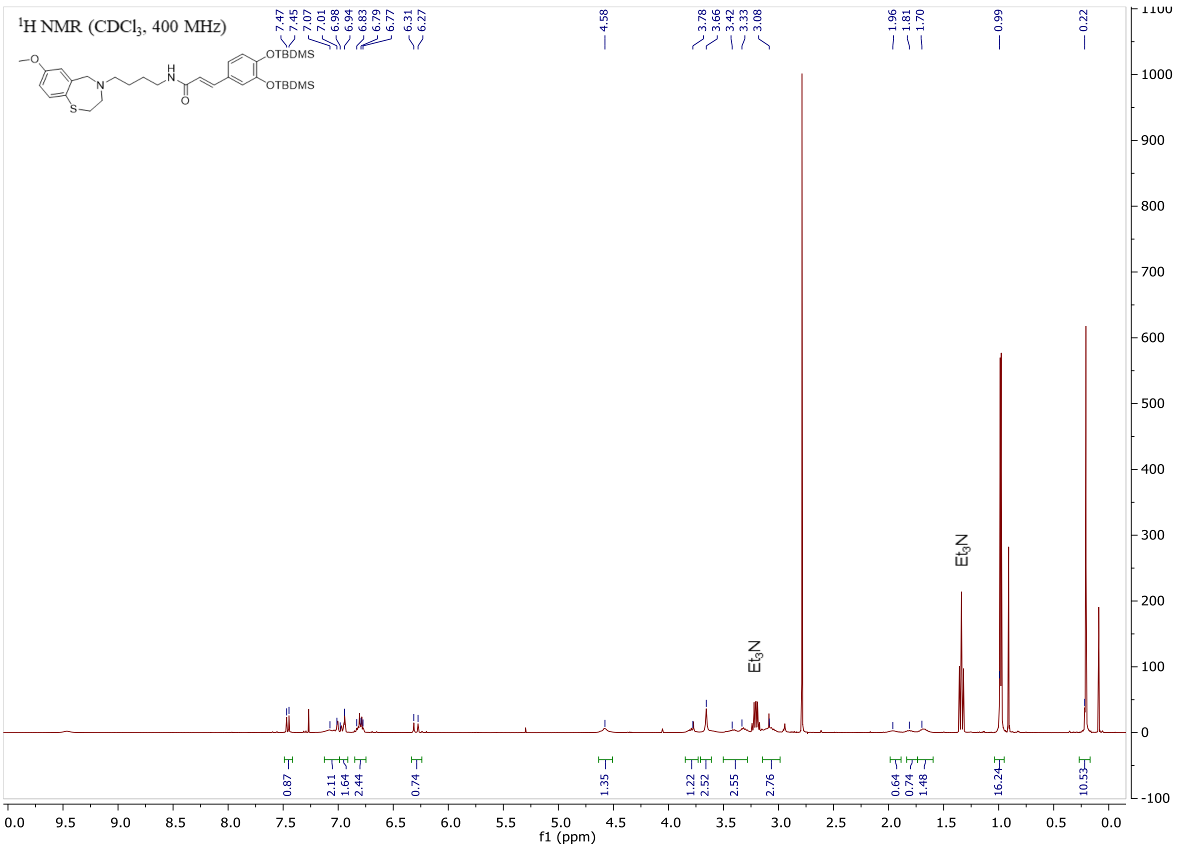

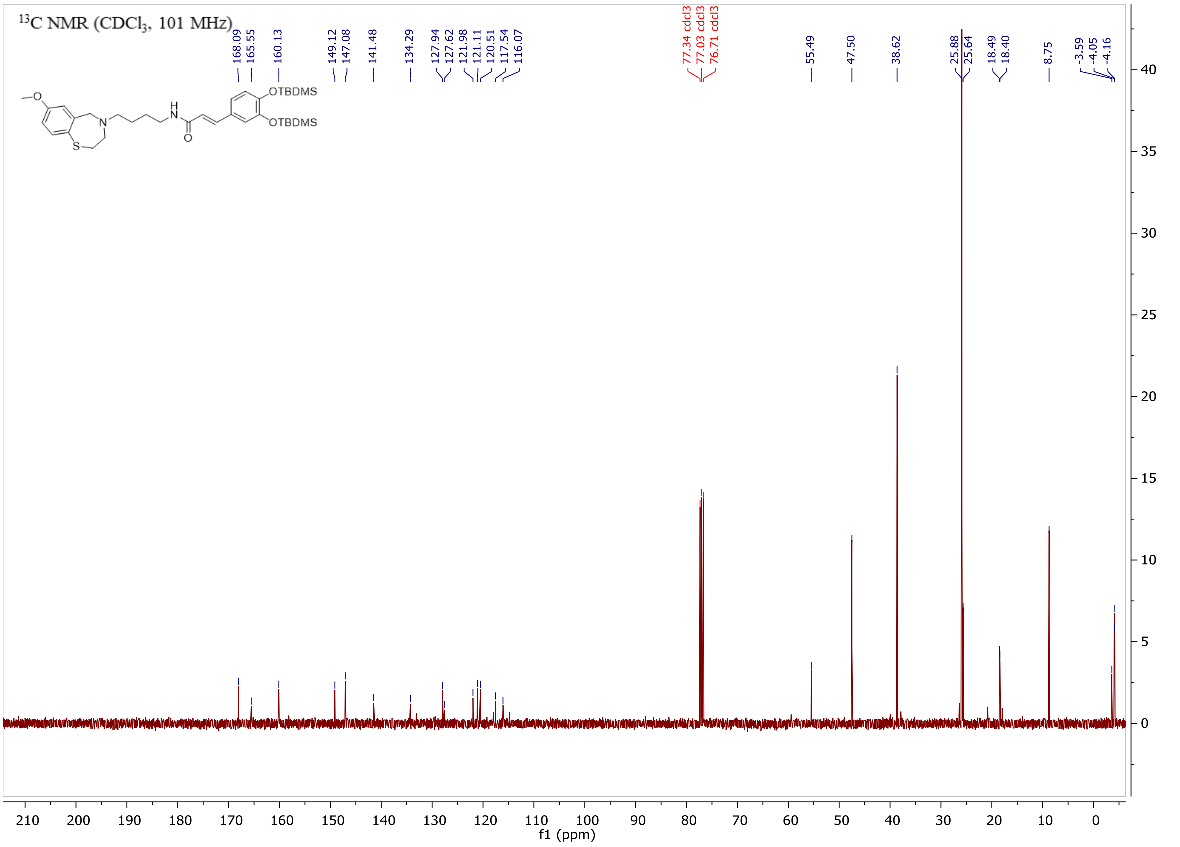

Figure S30. ^{1}H and ^{13}C spectra of compound **34**.

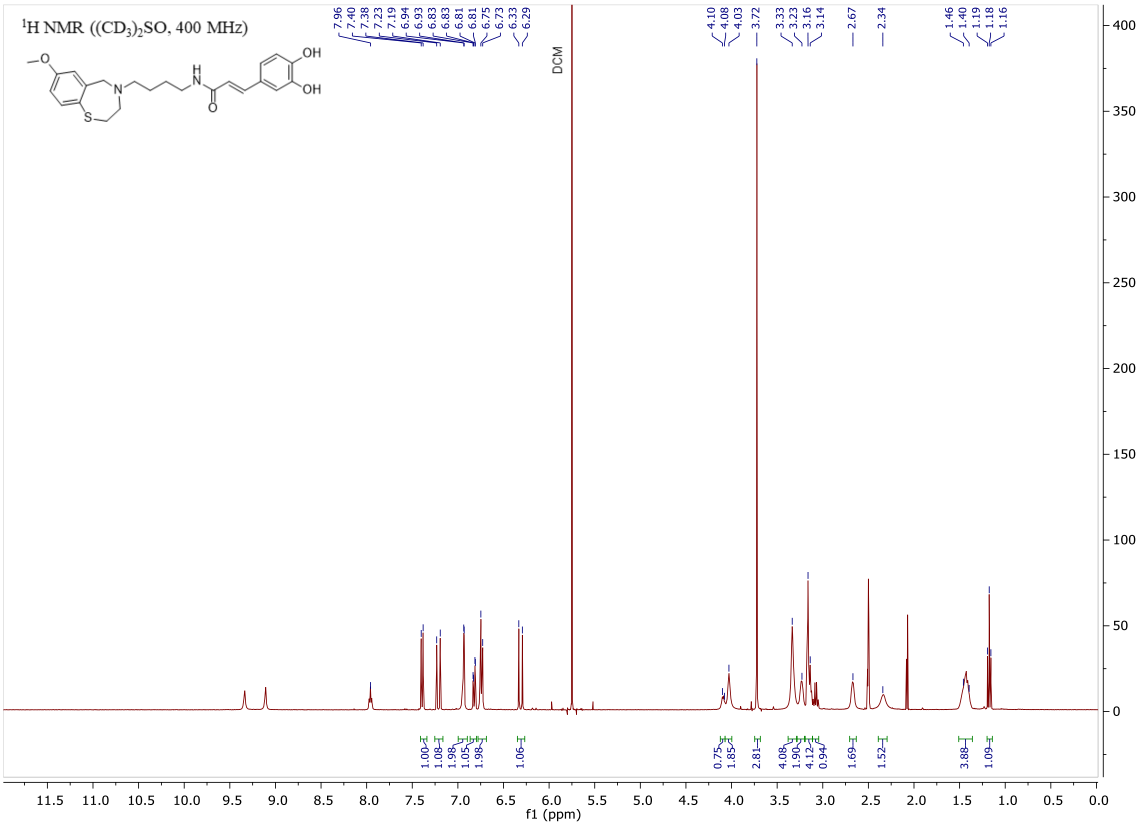

Figure S31. ^{1}H spectrum of compound **5.**

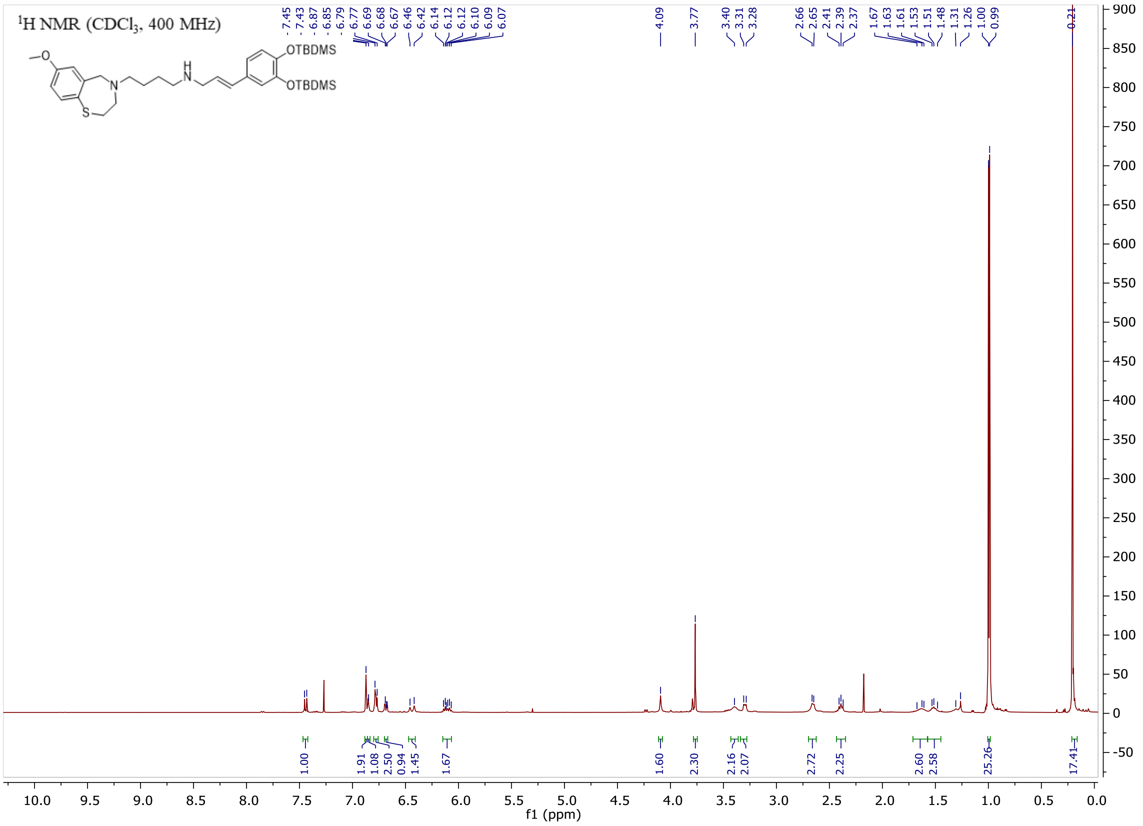

Figure S32. ^{1}H spectrum of compound **35.**

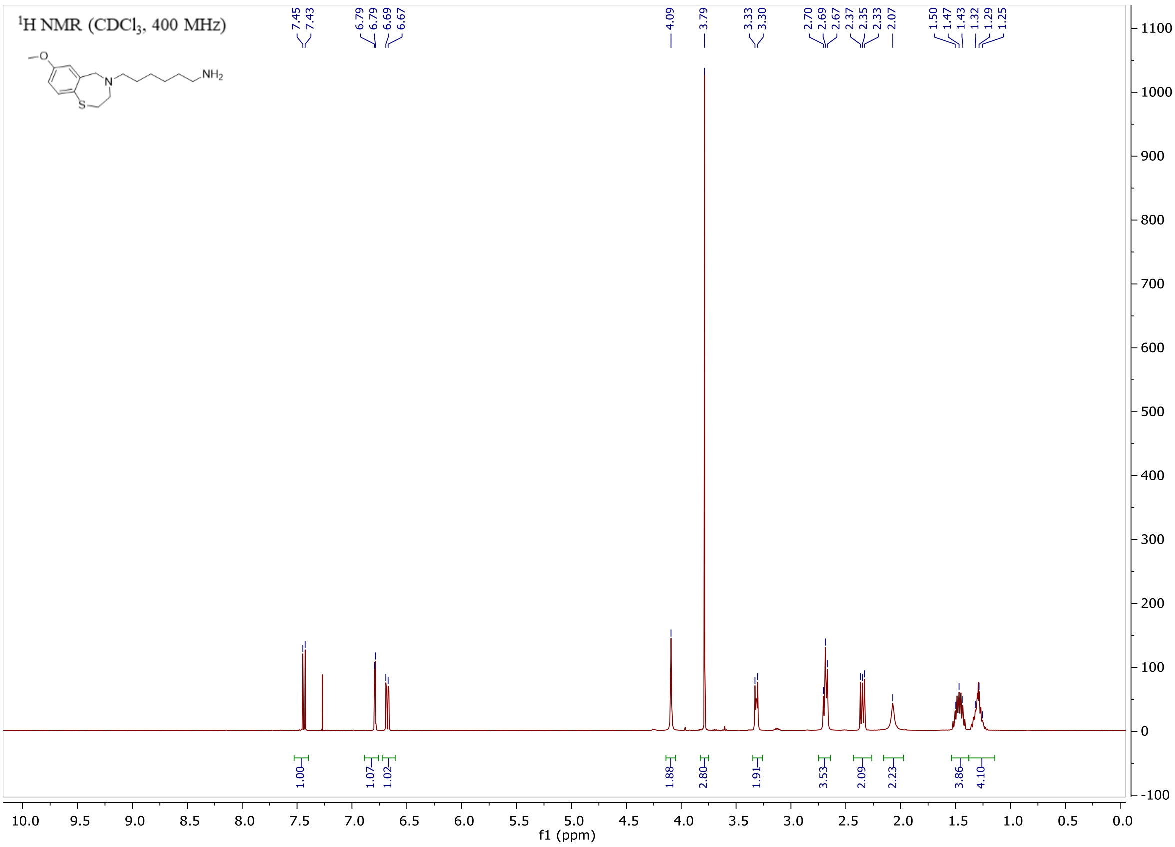

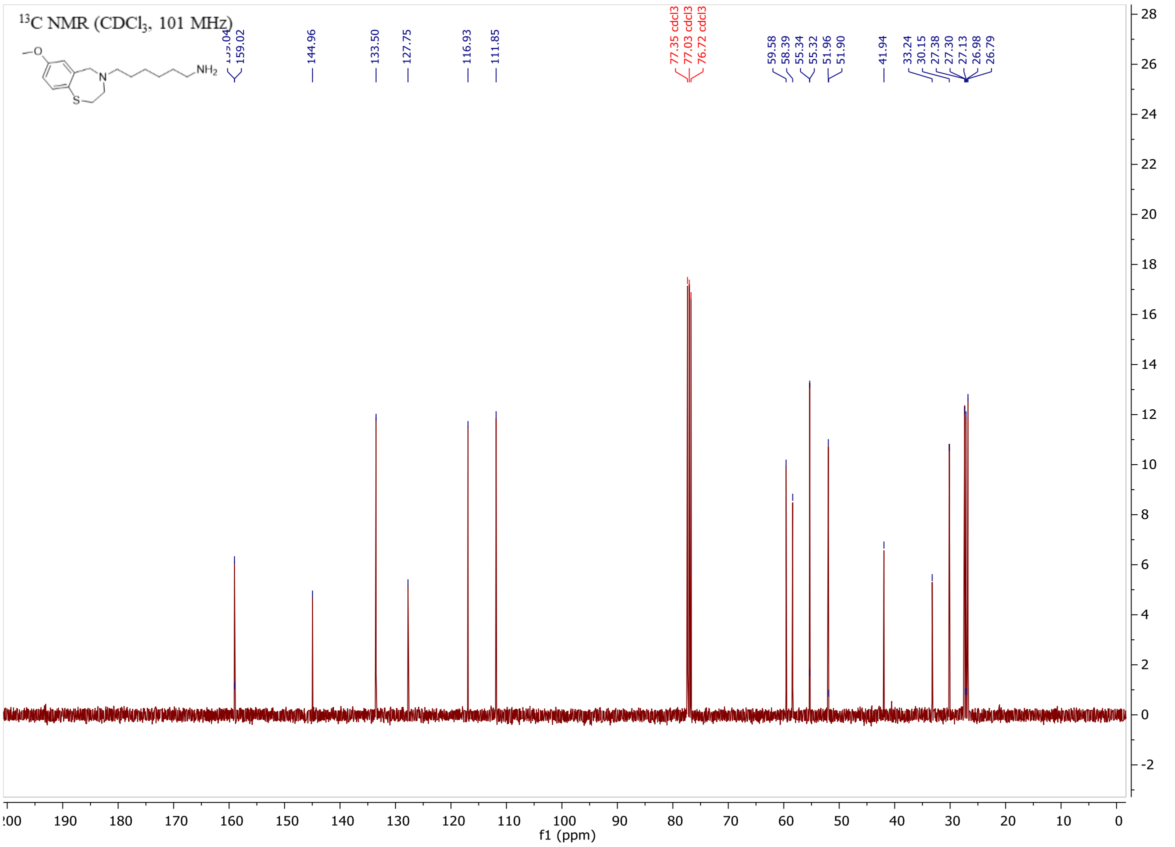

Figure S33. ^{1}H and ^{13}C spectra of compound **22.**

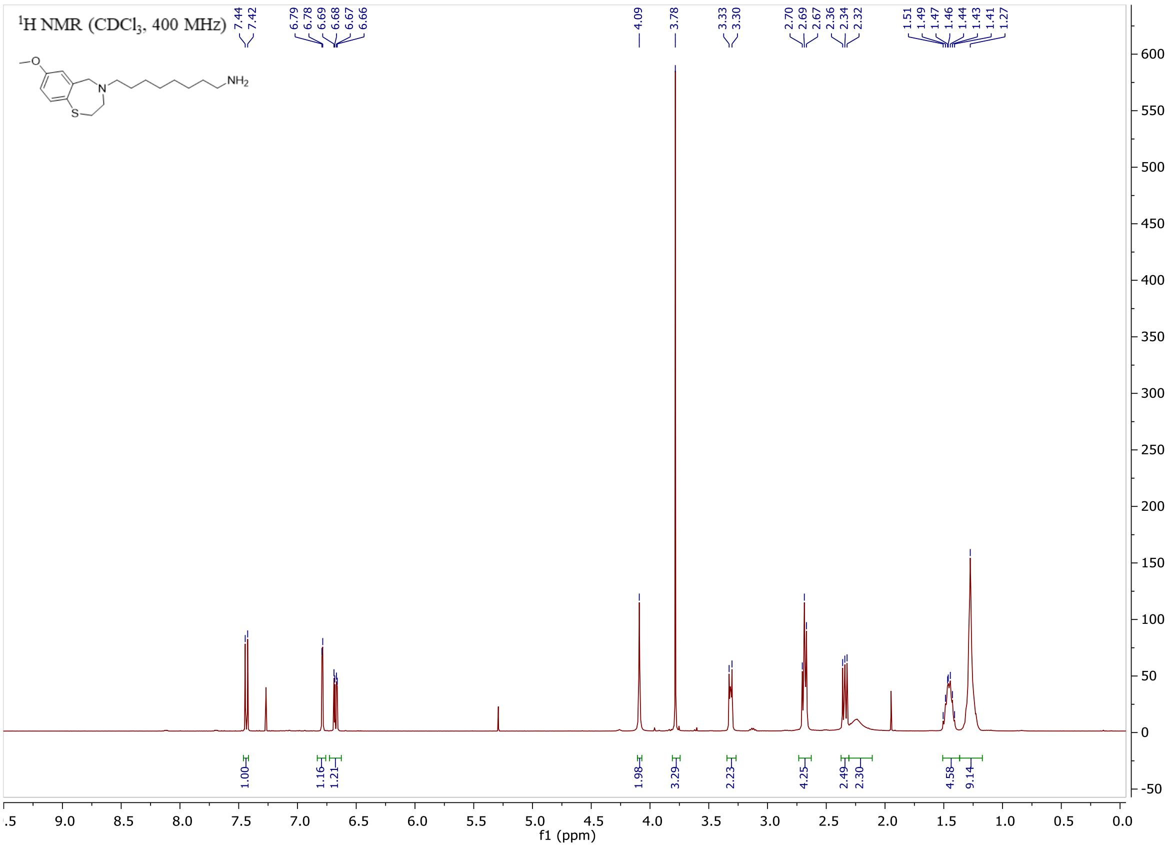

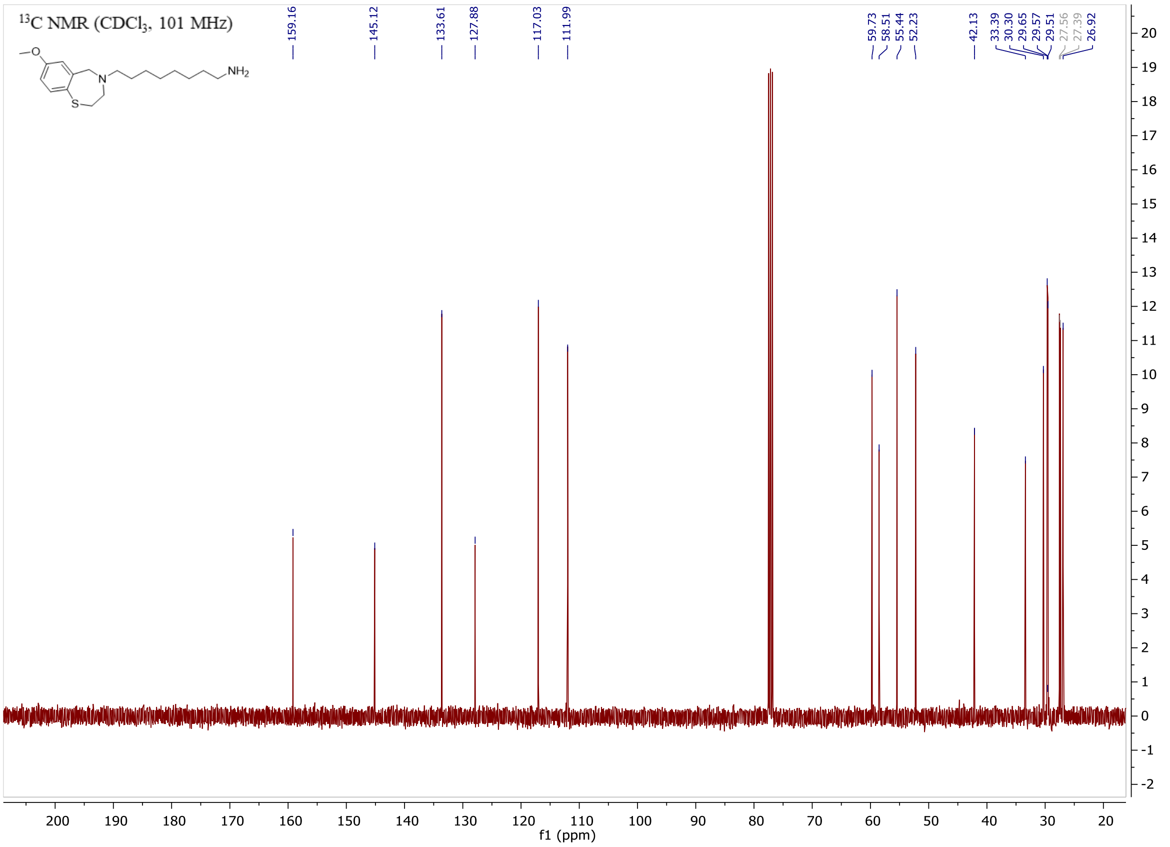

Figure S34. ¹H and ¹³C spectra of compound **23.**

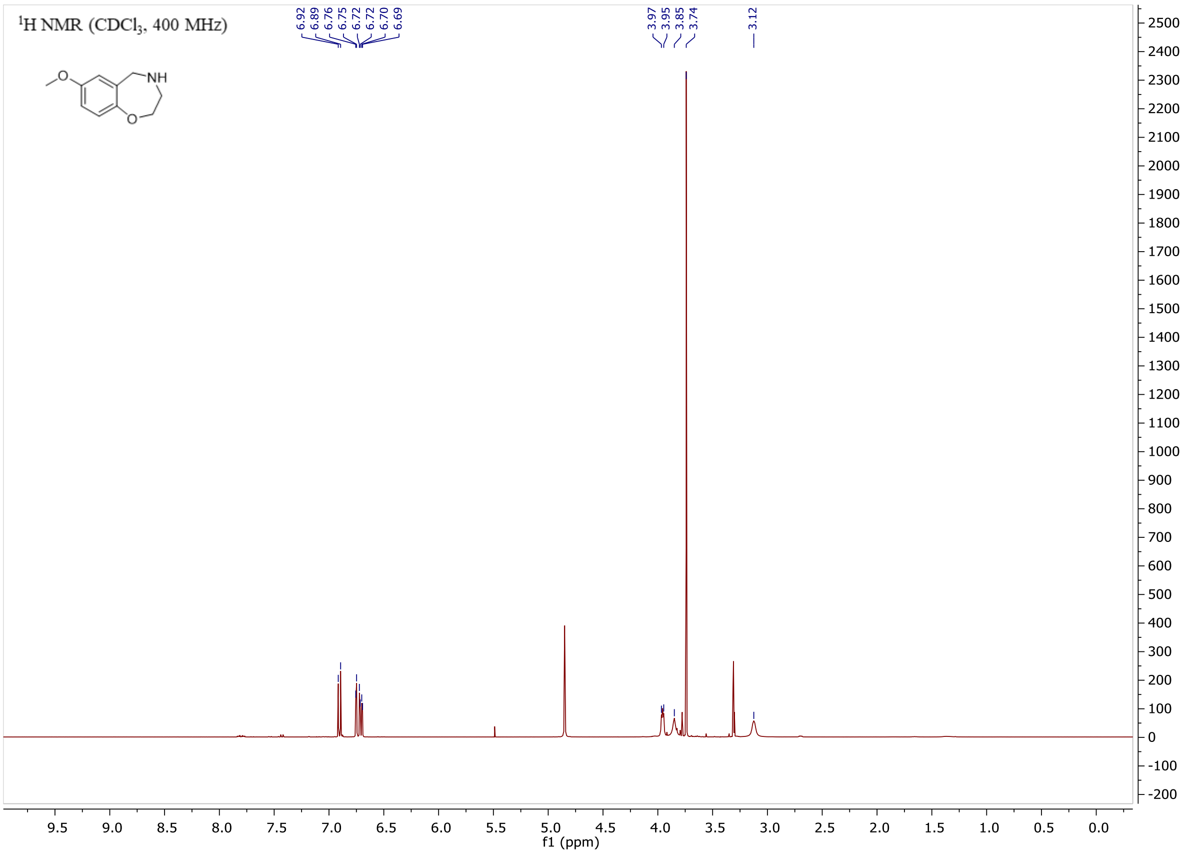

Figure S35. ¹H spectrum of compound **37.**

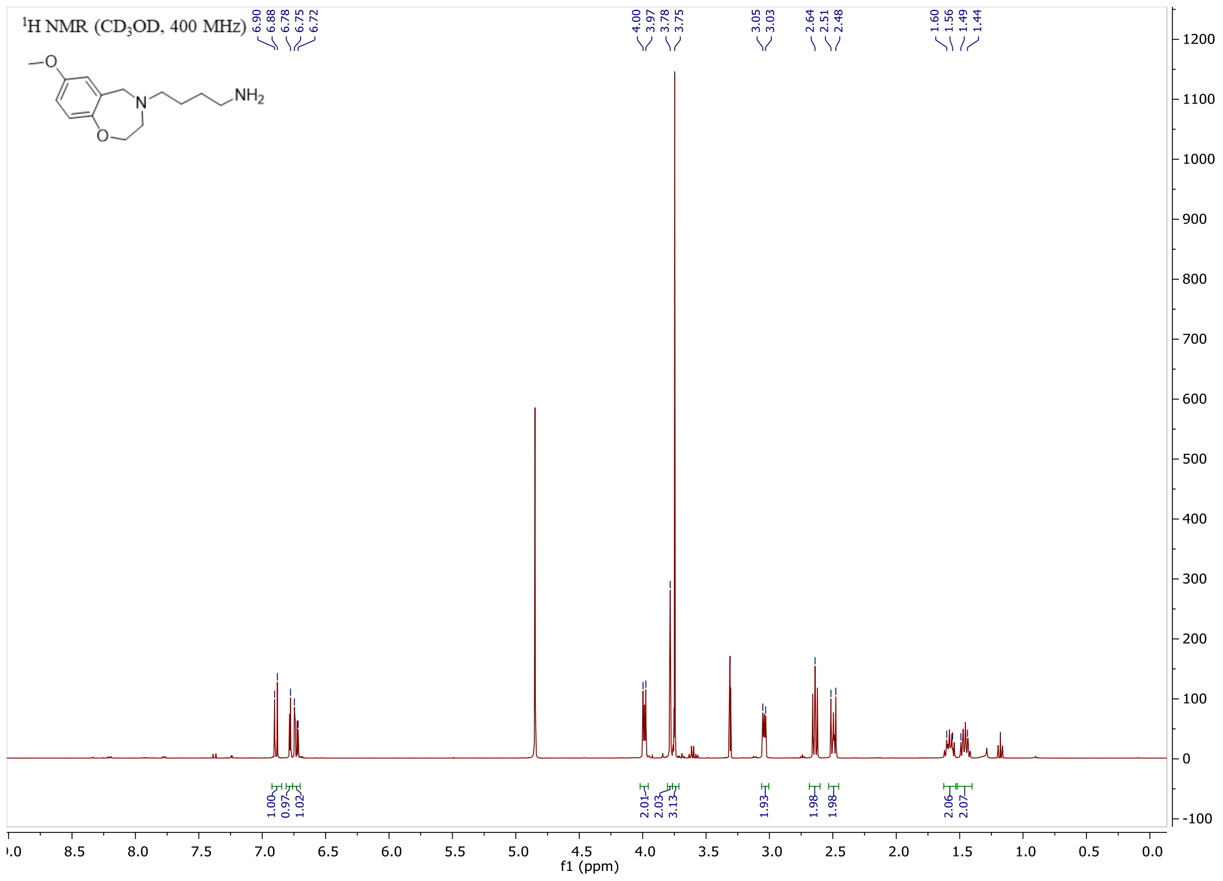

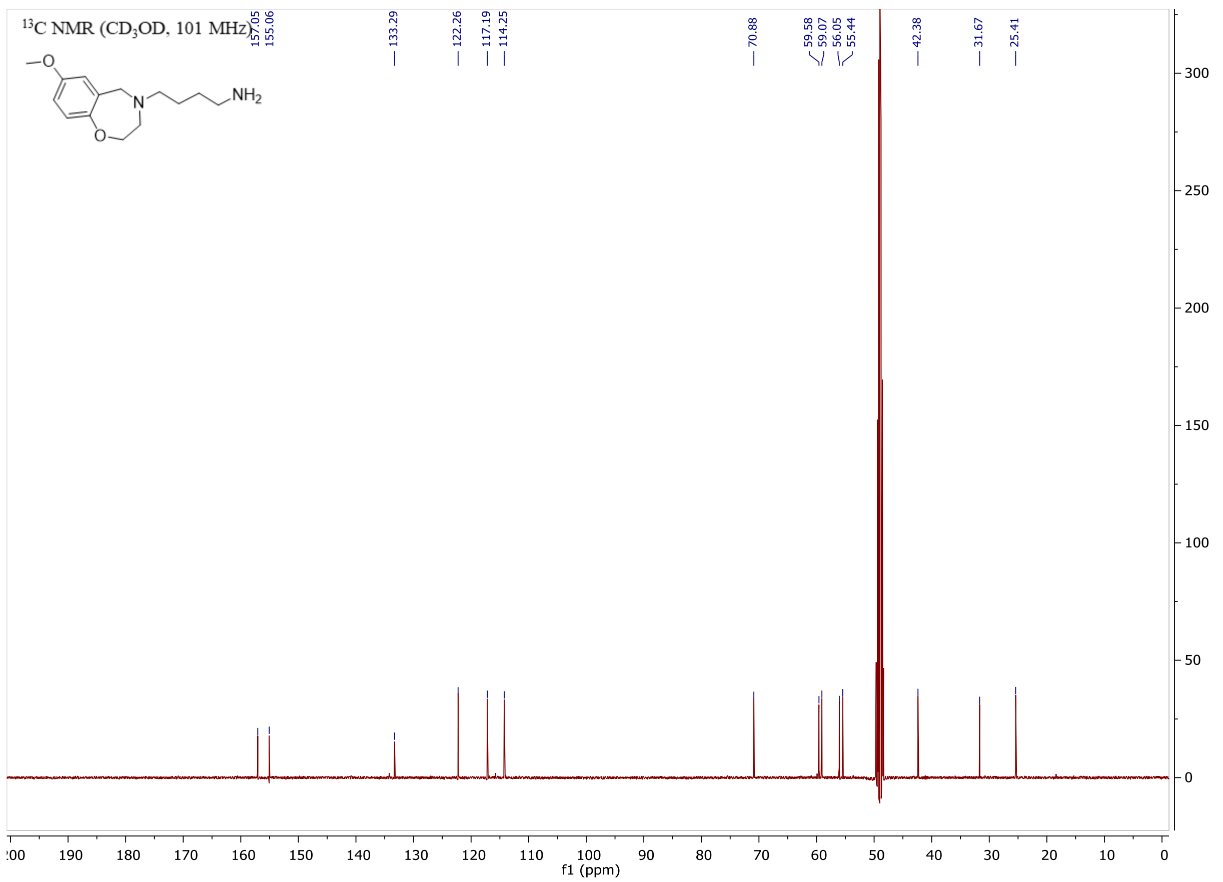

Figure S36. ^{1}H and ^{13}C spectra of compound **24.**

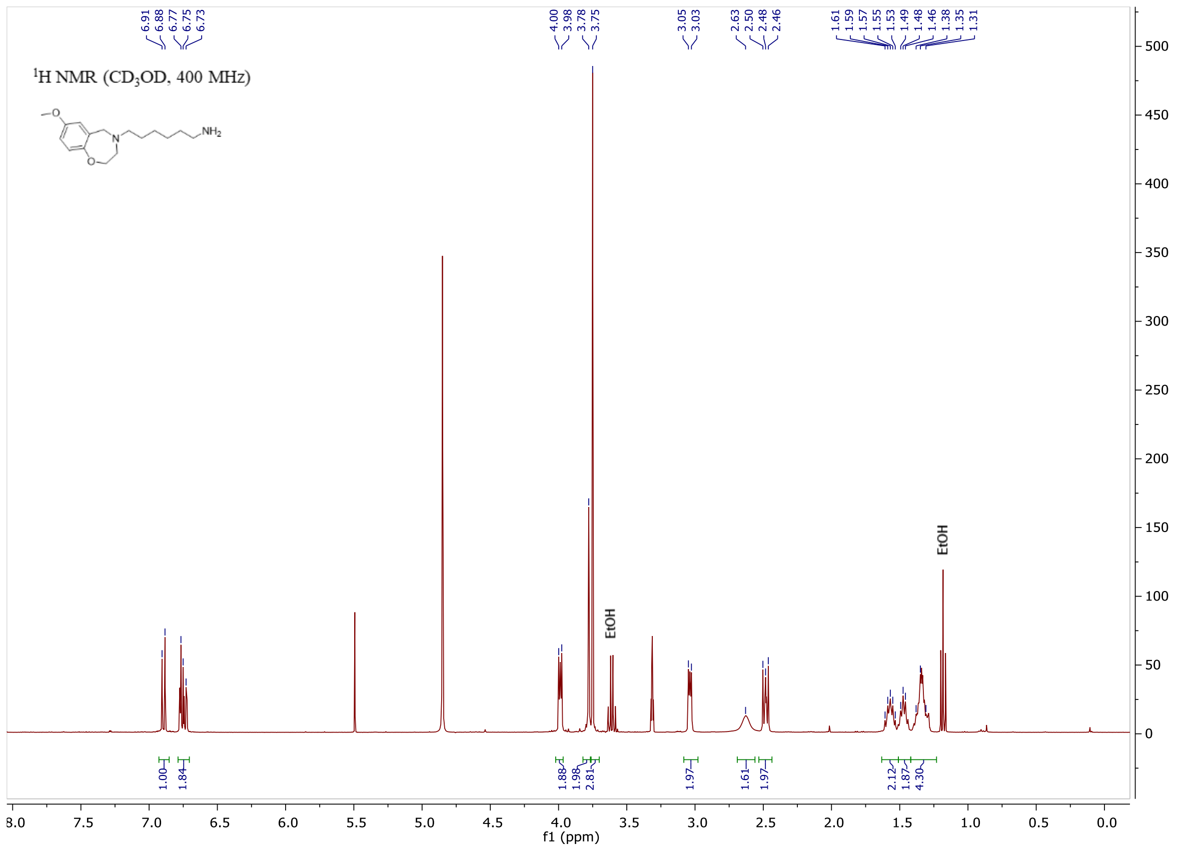

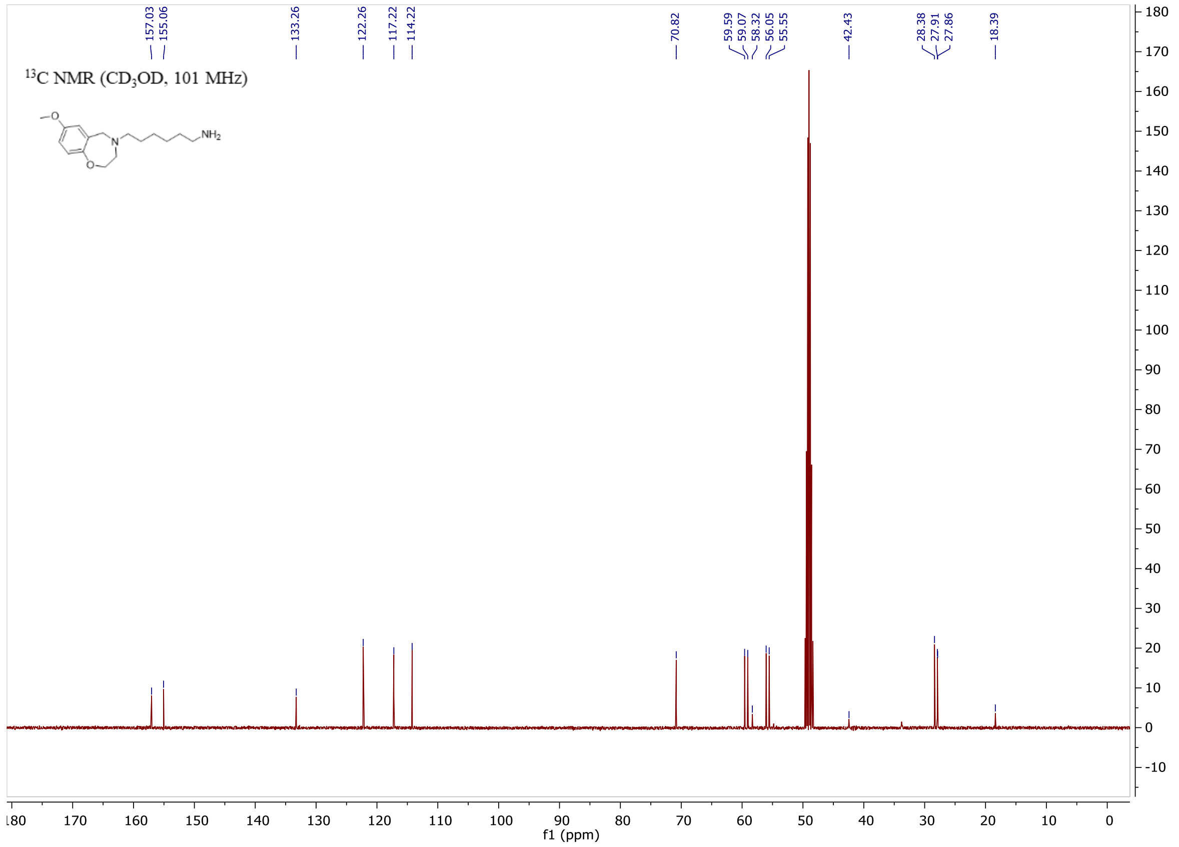

Figure S37. ^{1}H and ^{13}C spectra of compound **25.**

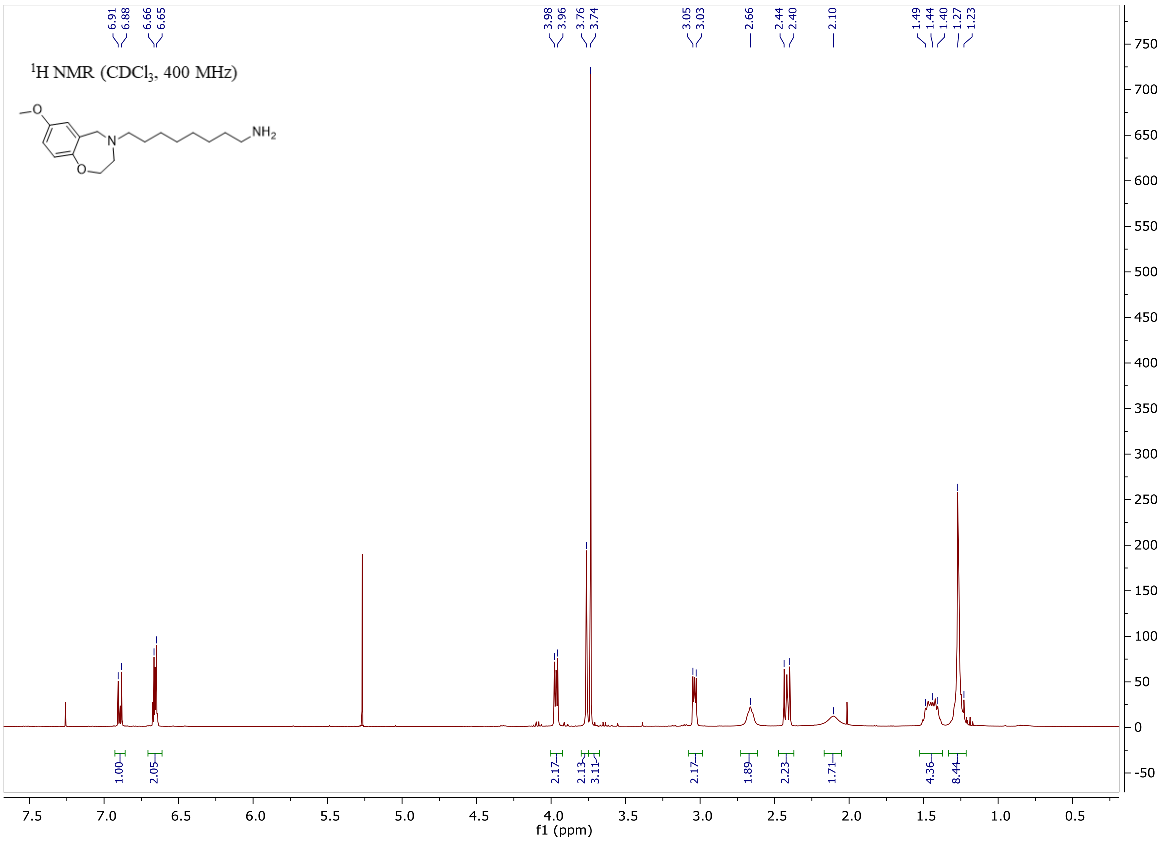

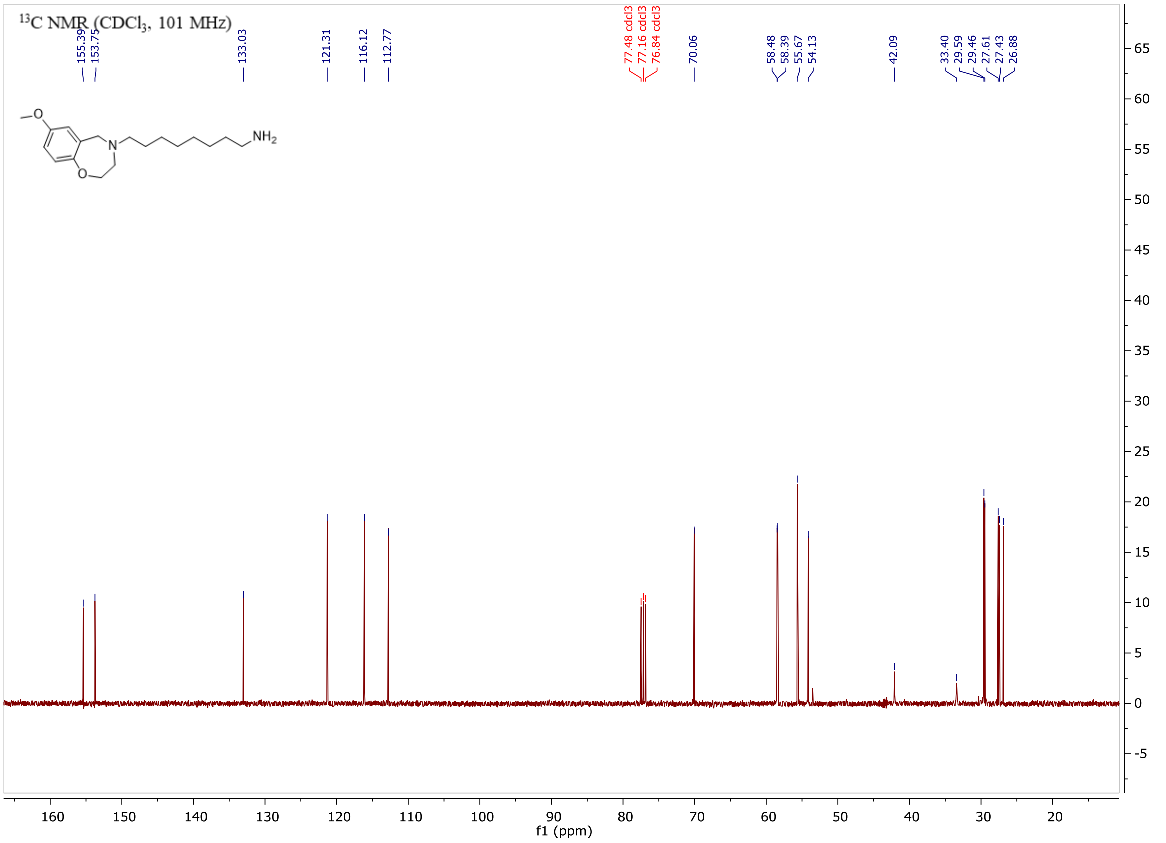

Figure S38. ^{1}H and ^{13}C spectra of compound **26.**

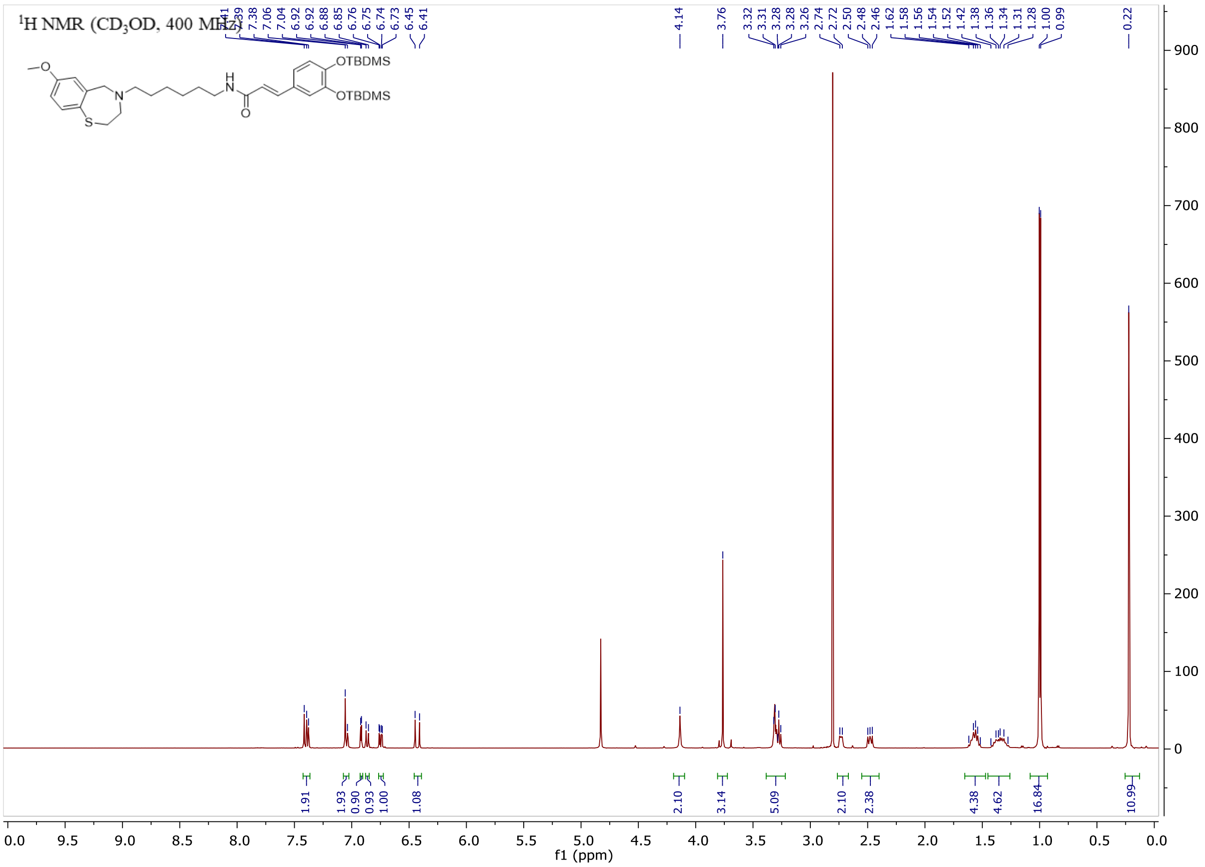

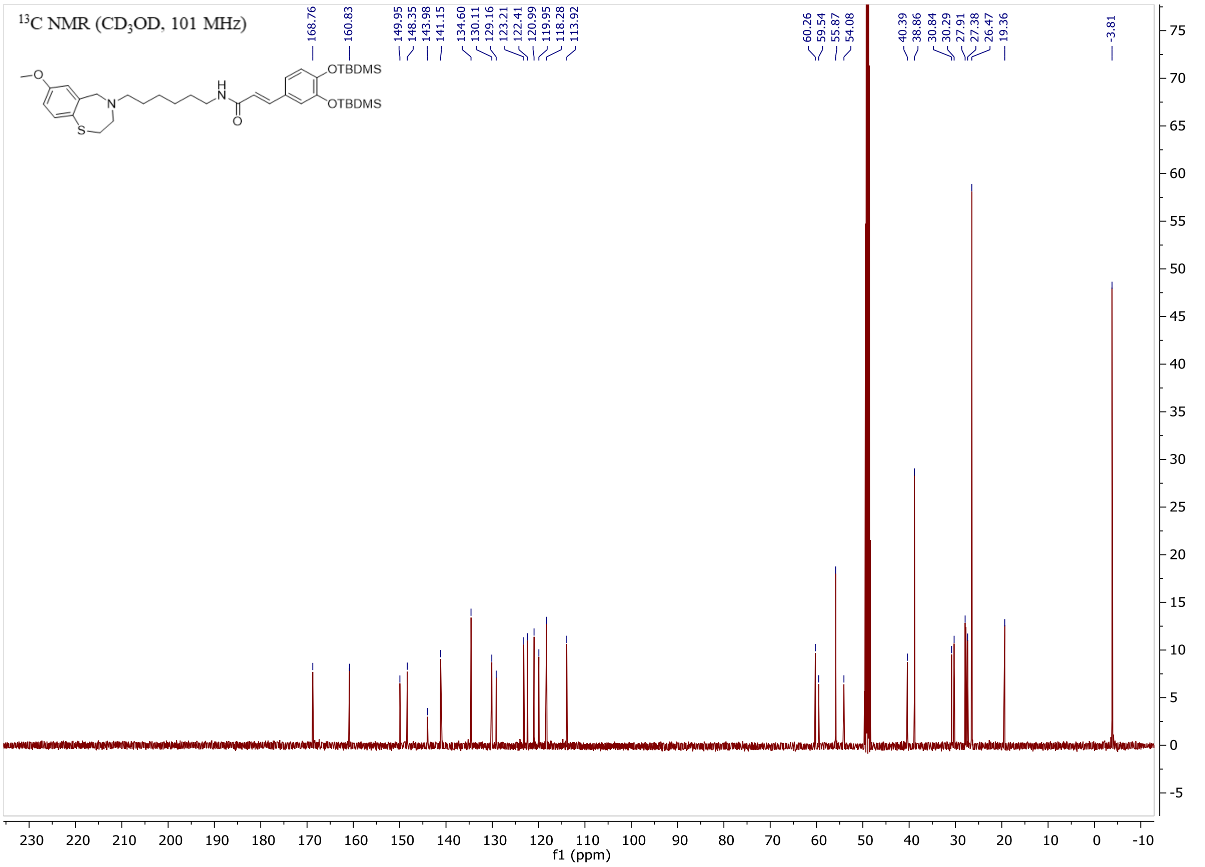

Figure S39. ^{1}H and ^{13}C spectra of compound **38.**

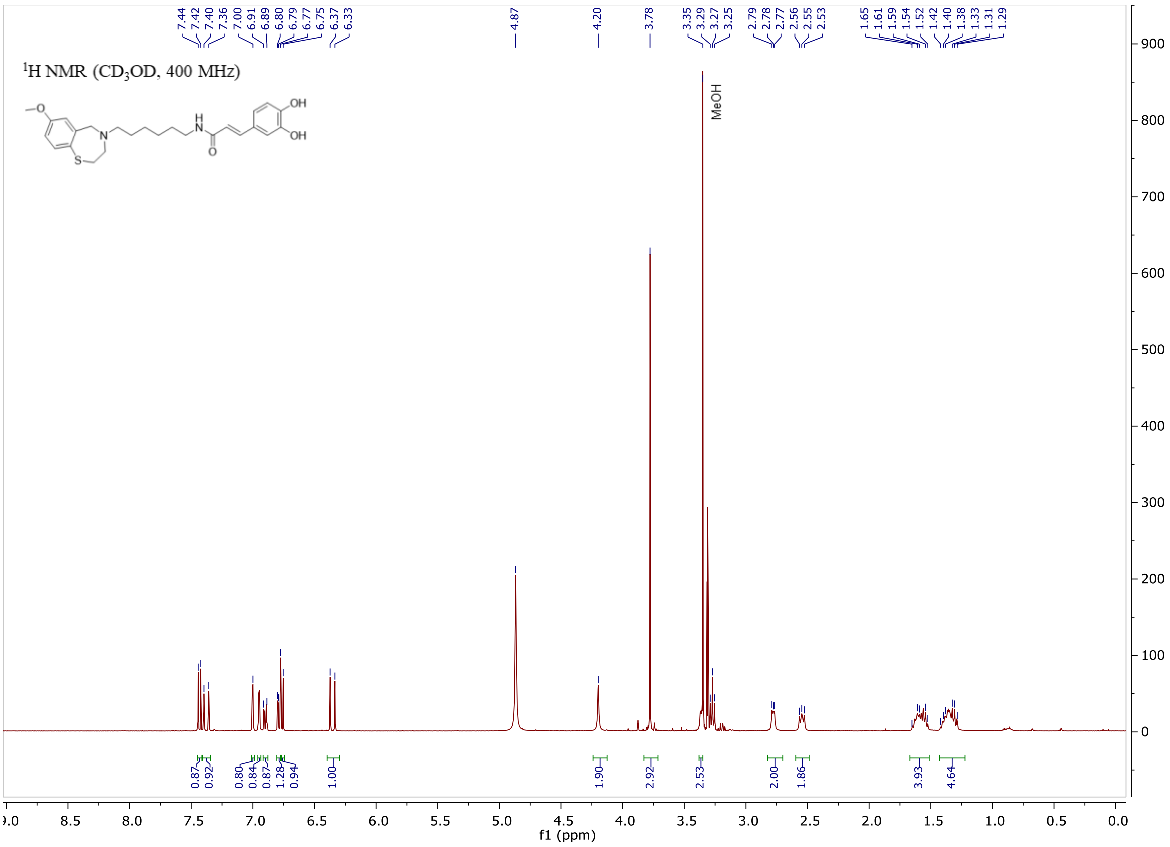

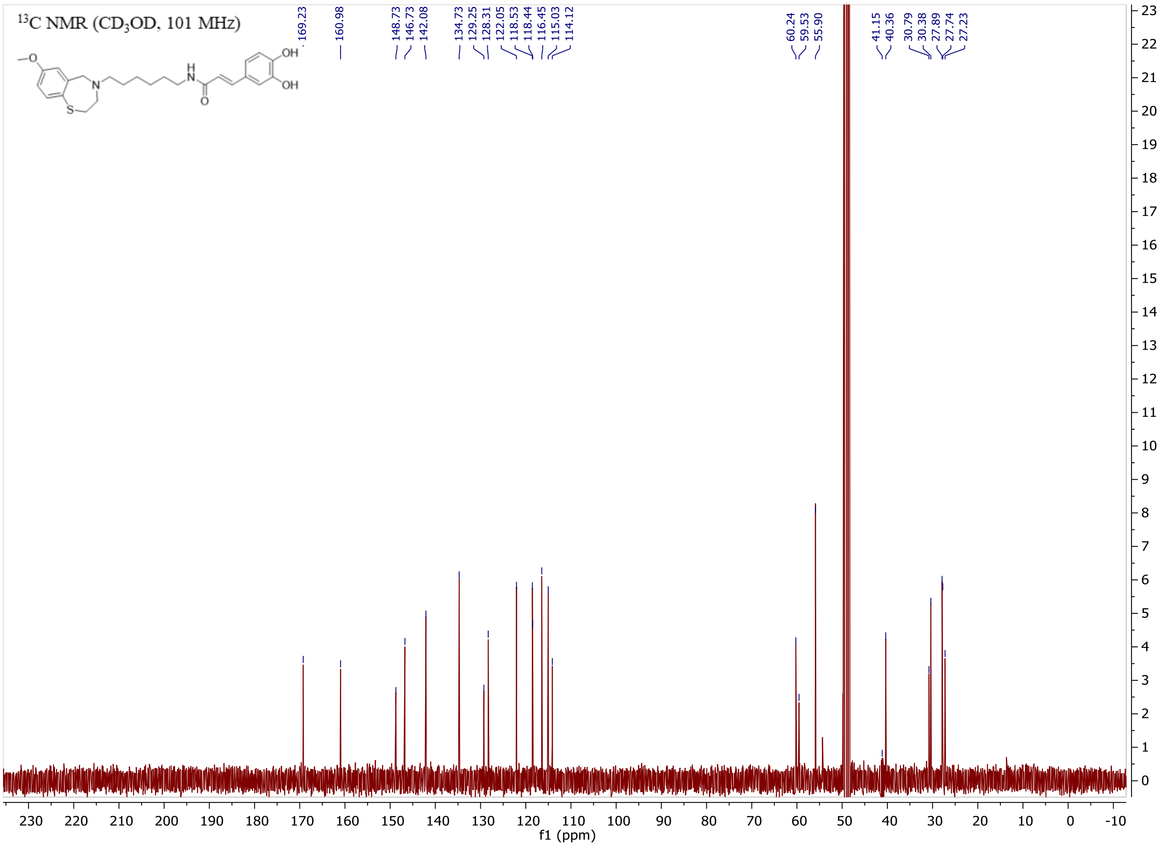

Figure S40. ^{1}H and ^{13}C spectra of compound **6.**

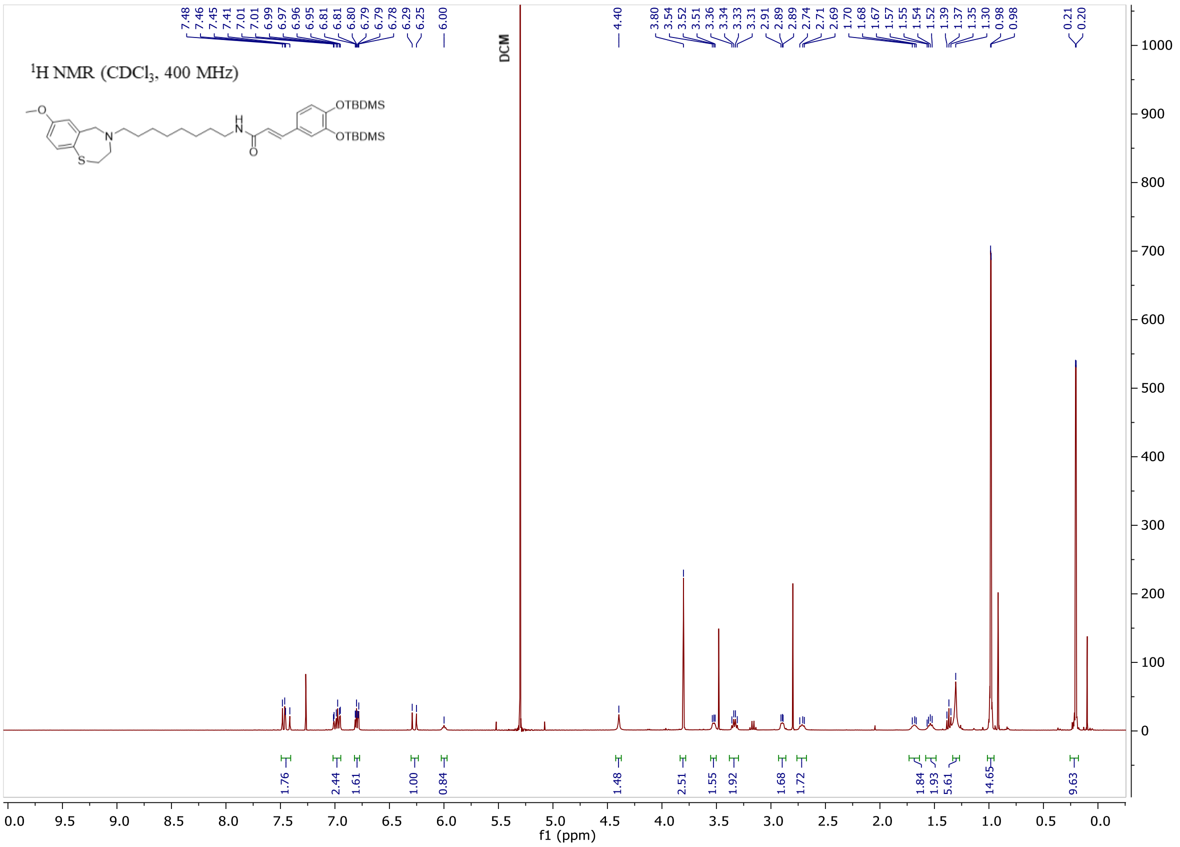

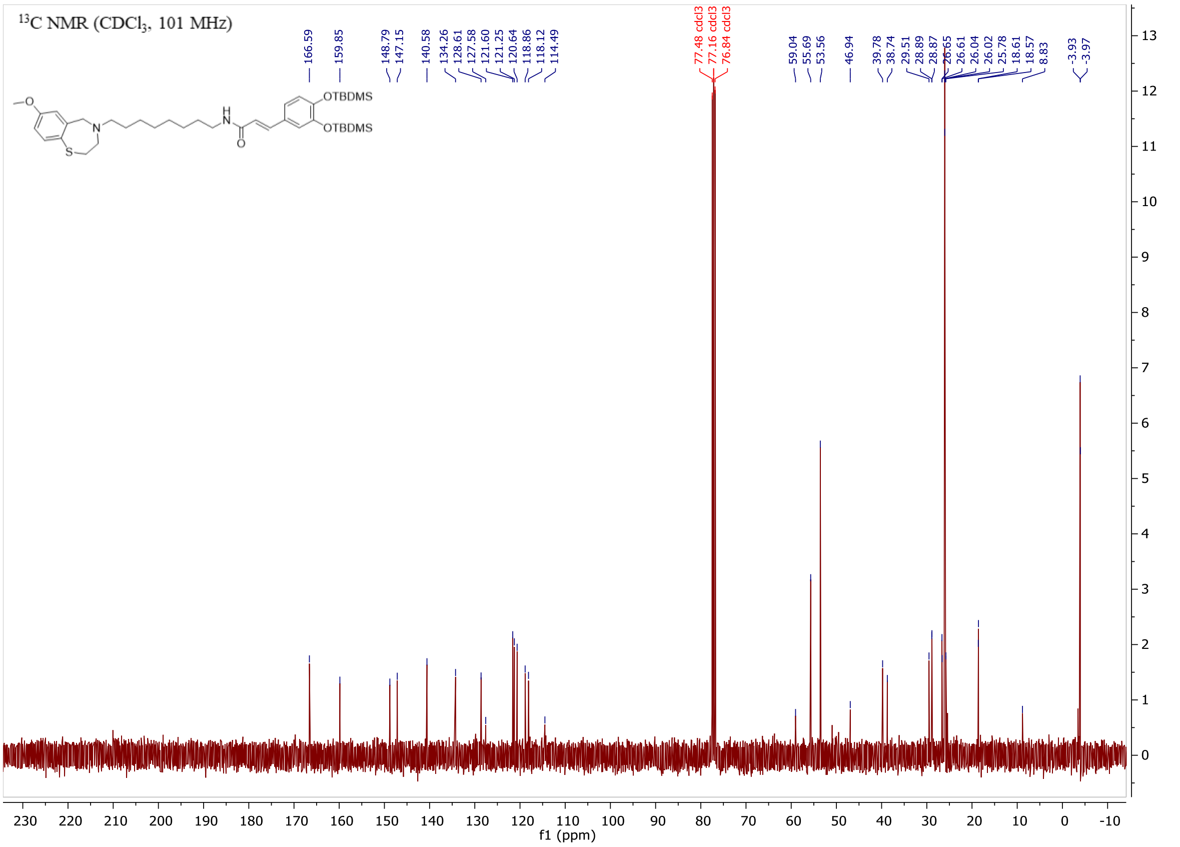

Figure S41. ¹H and ¹³C spectra of compound **39.**

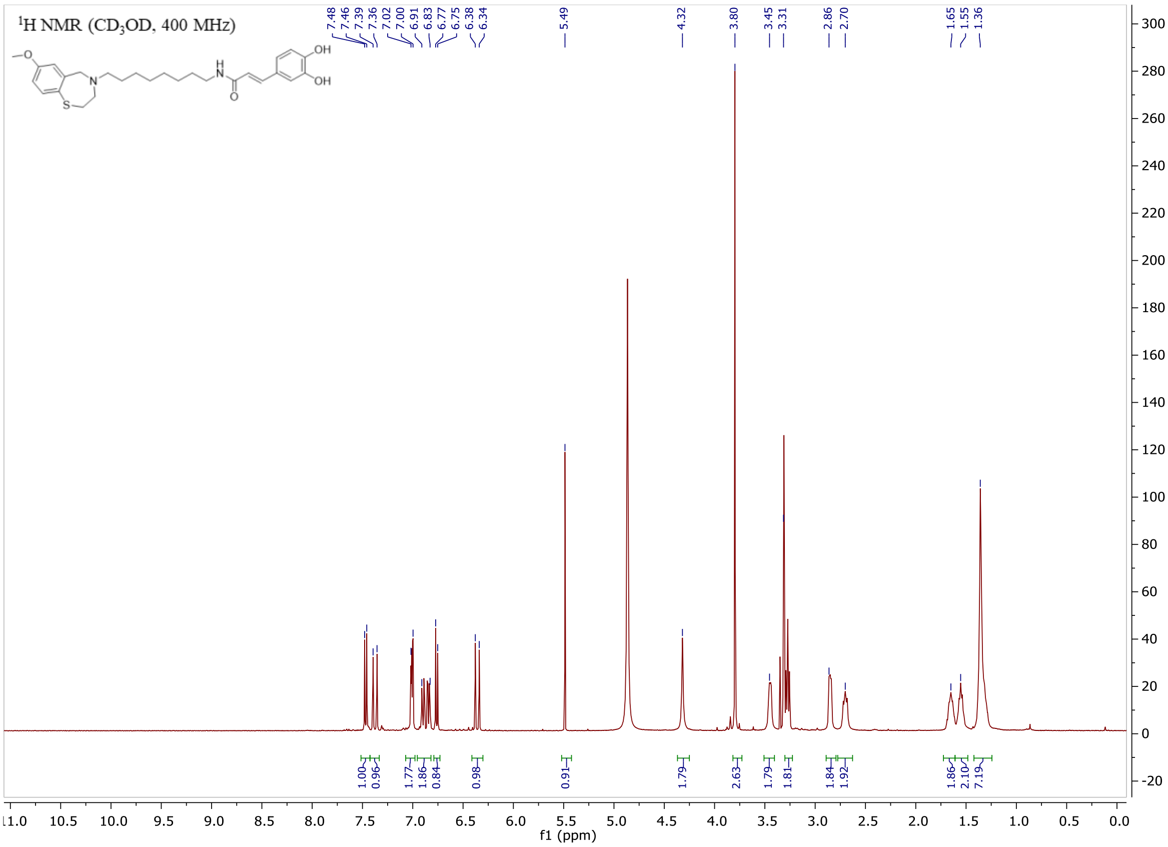

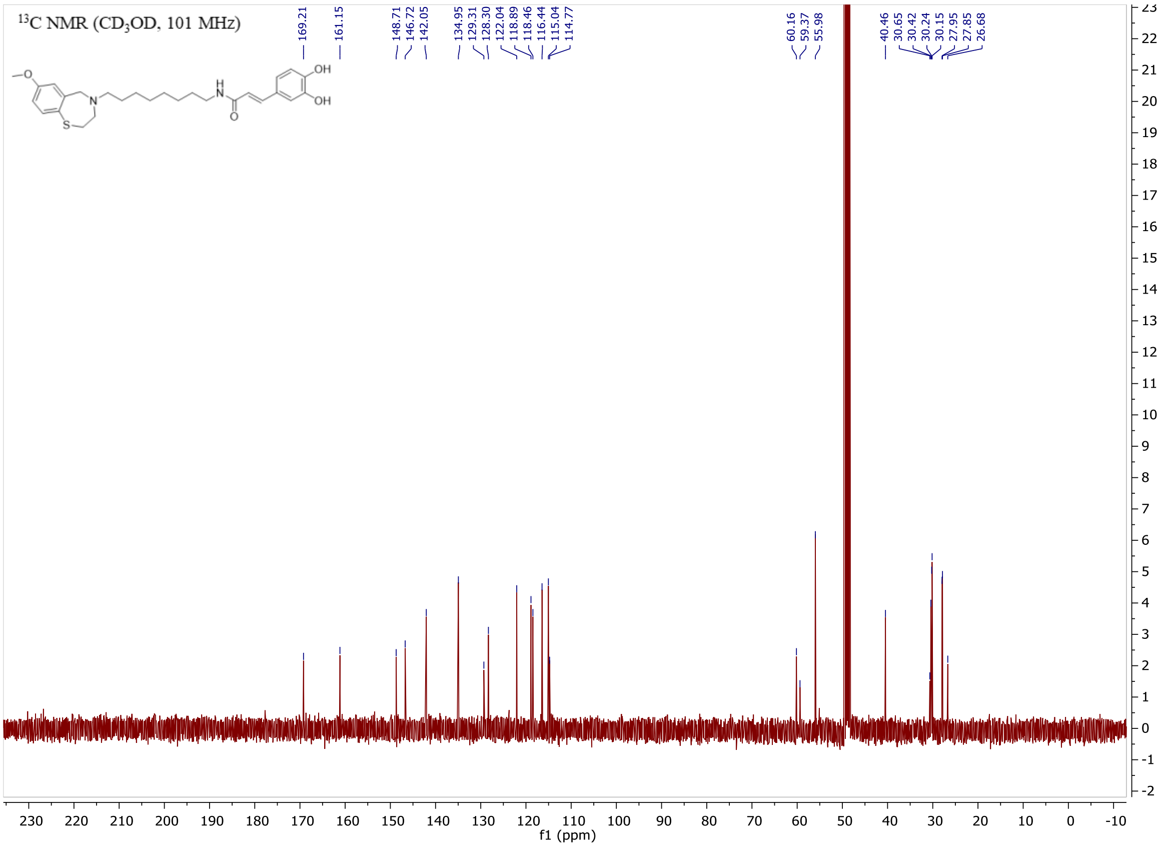

Figure S42. ^{1}H and ^{13}C spectra of compound **7.**

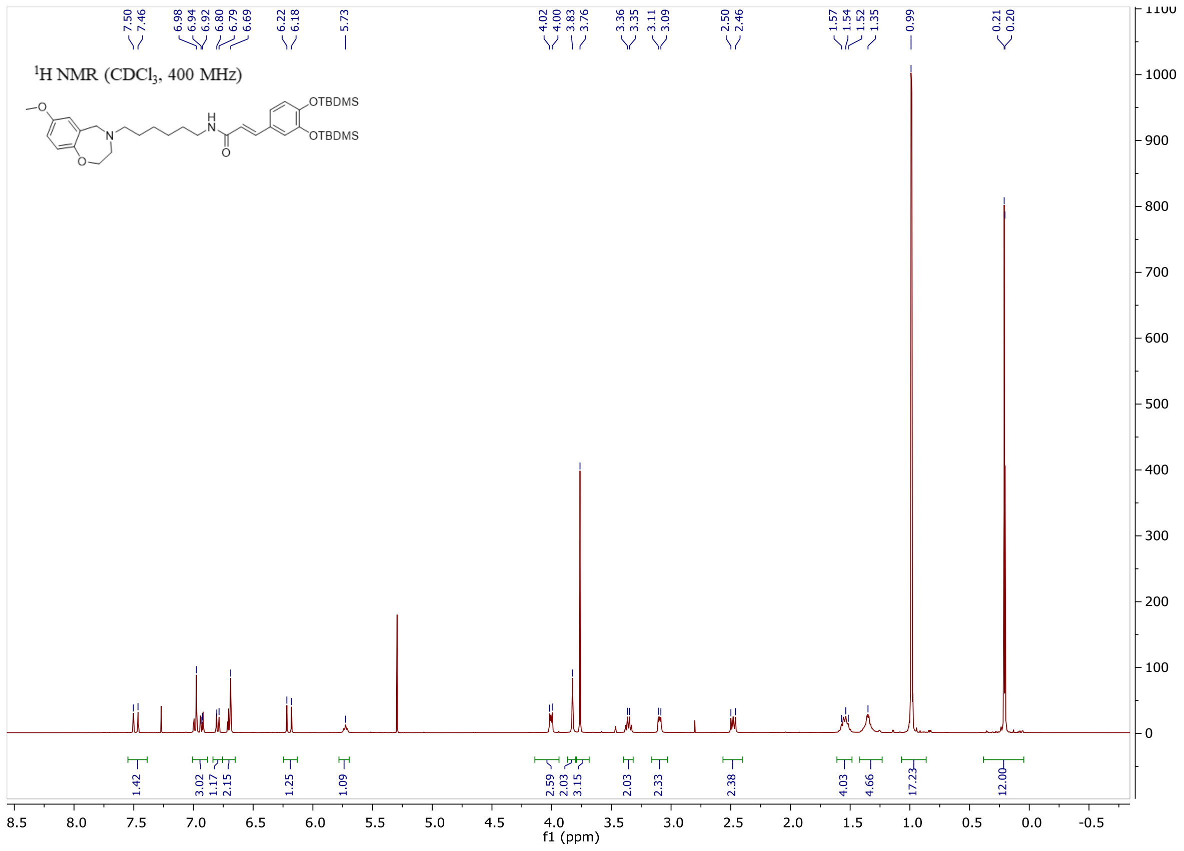

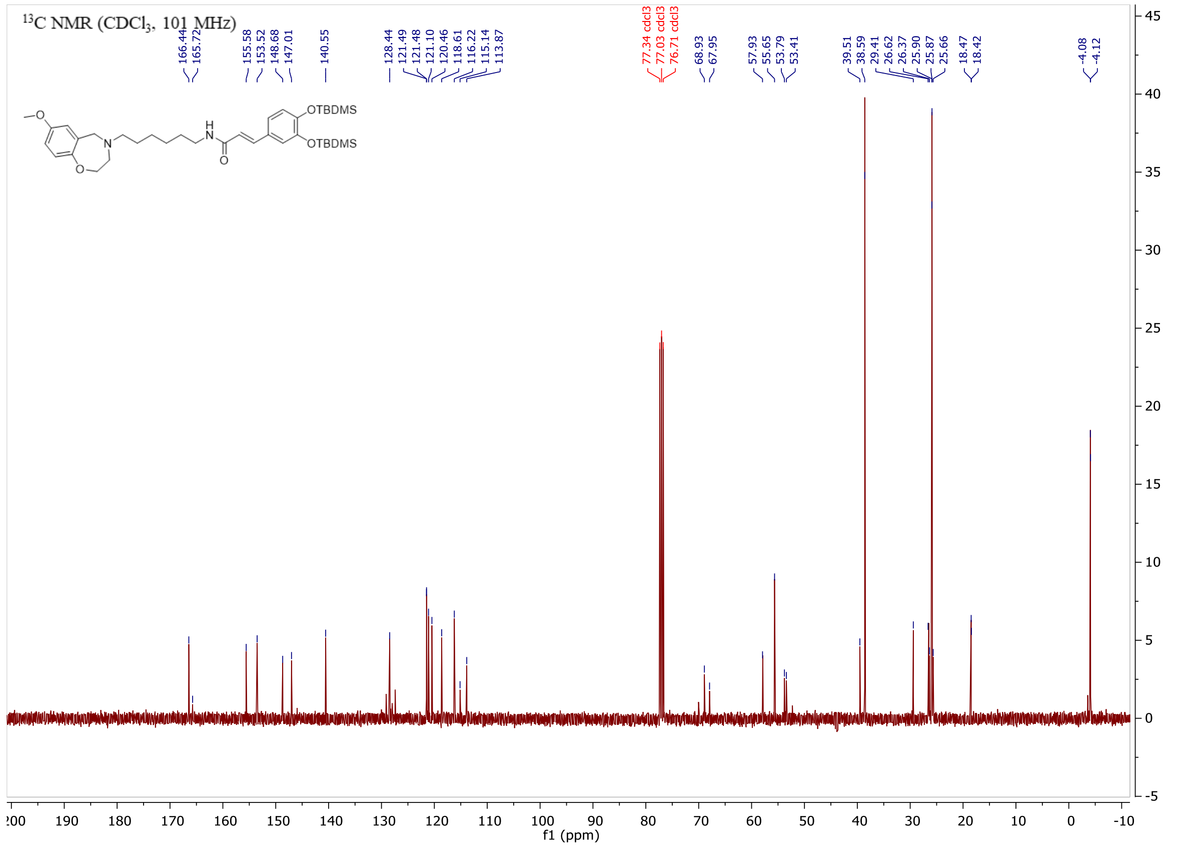

Figure S43. ^{1}H and ^{13}C spectra of compound **40.**

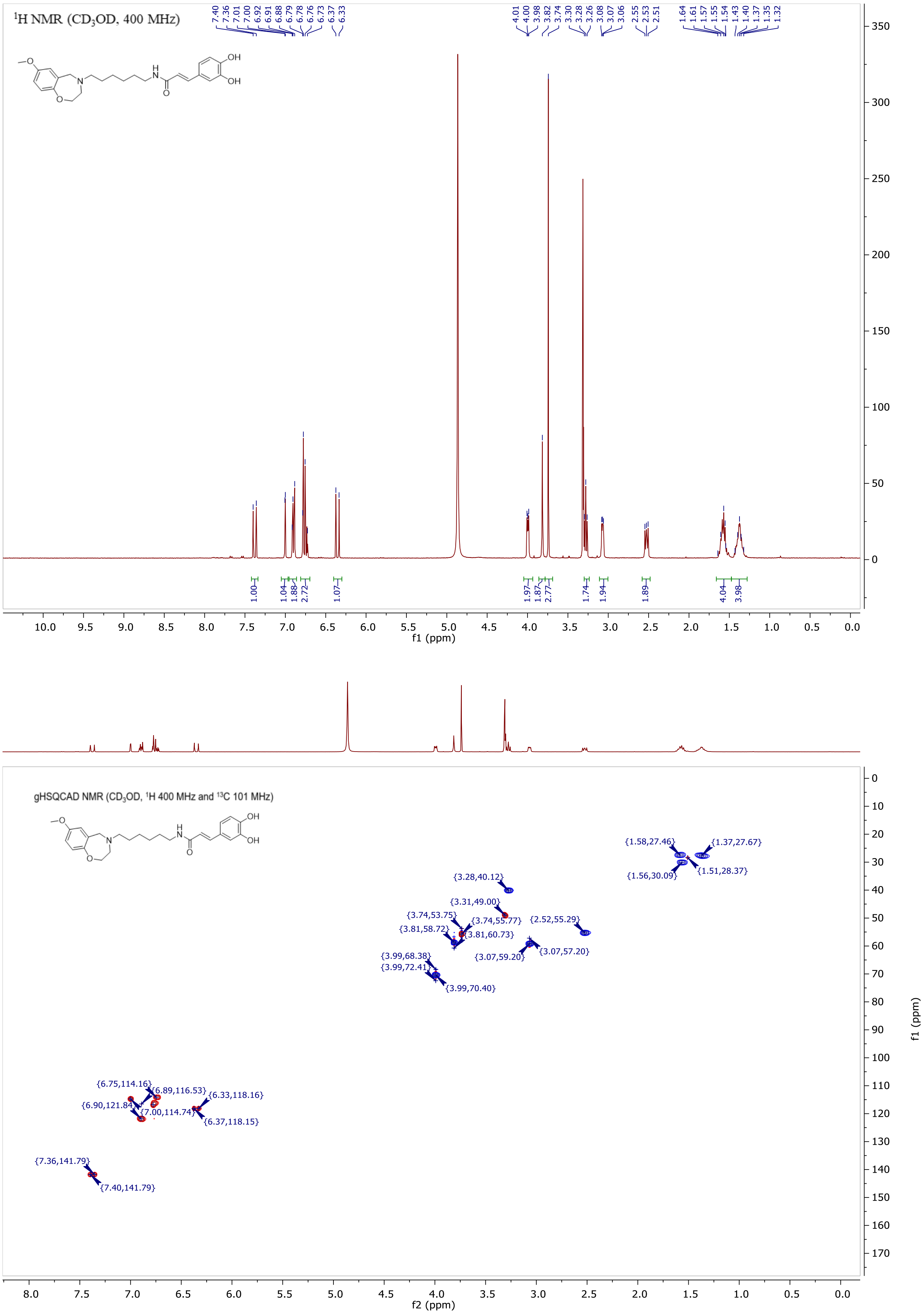

Figure S44. ^{1}H and gHSQCAD spectra of compound **8.**

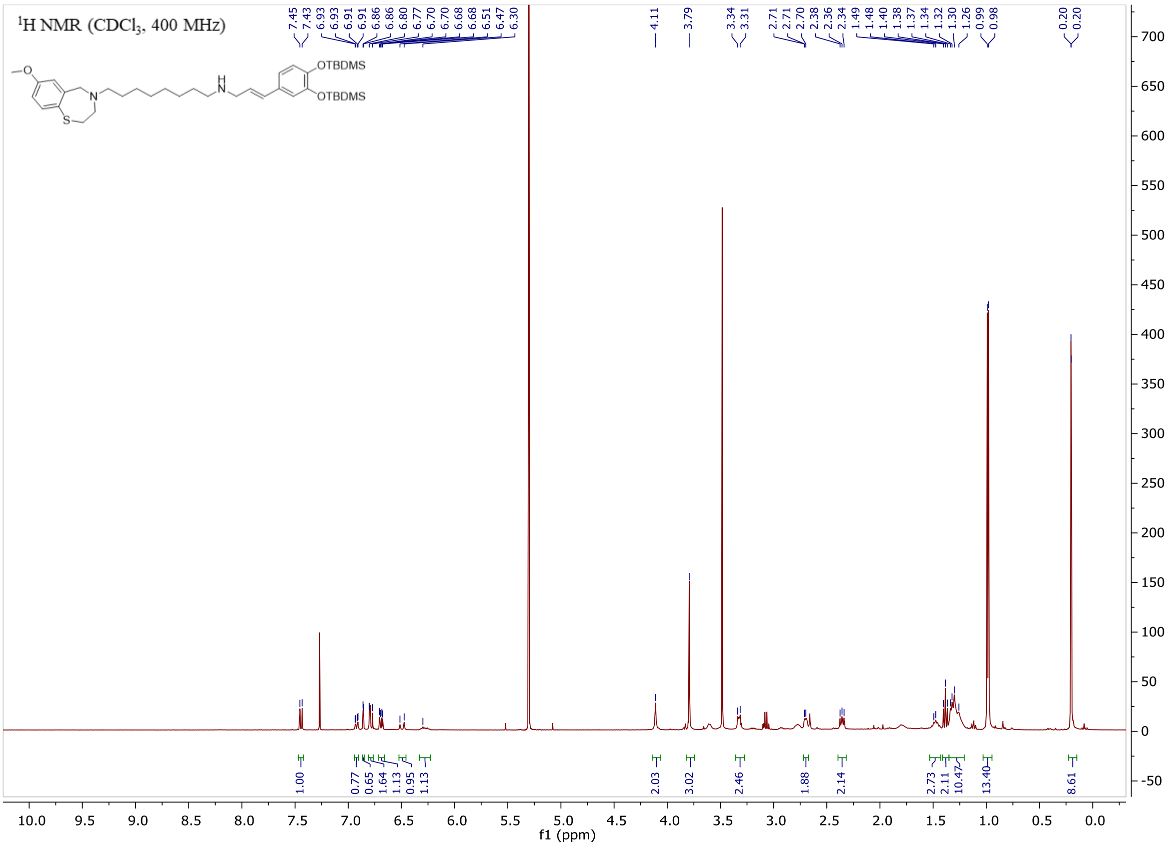

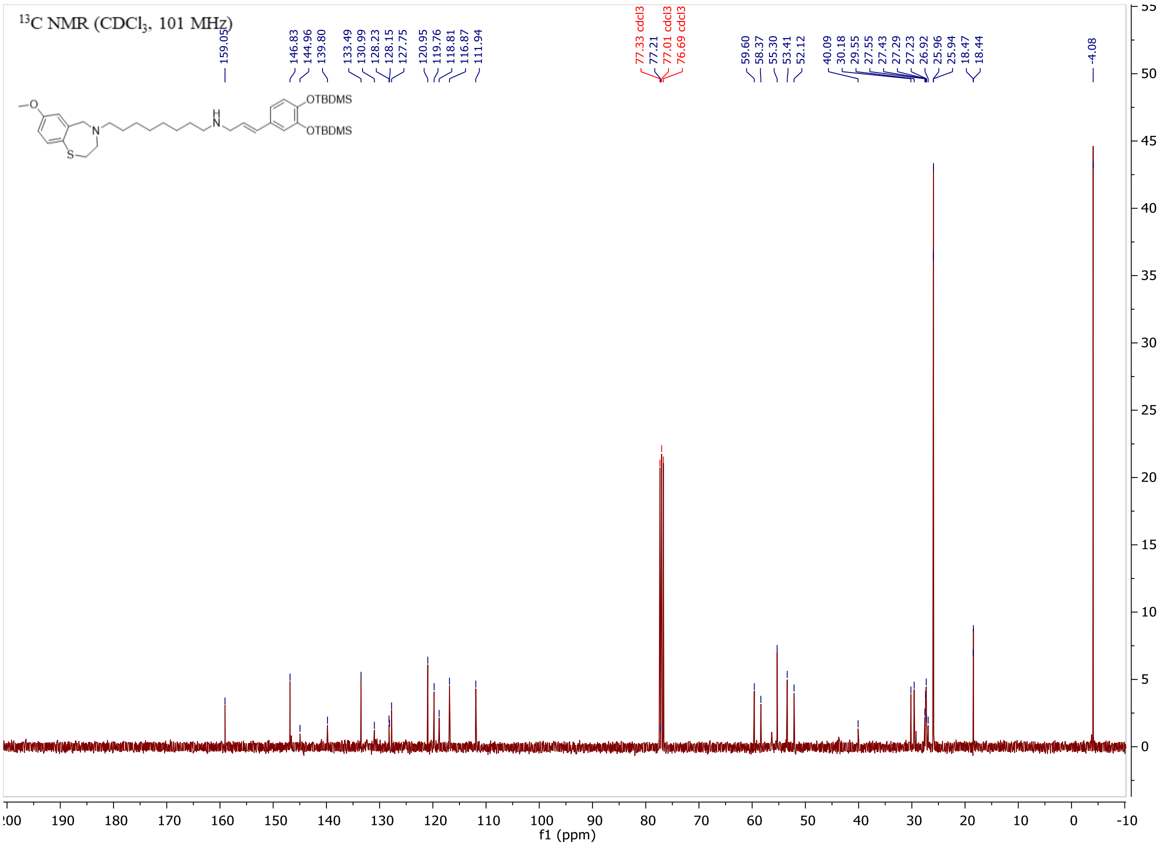

Figure S45. ^{1}H and ^{13}C spectra of compound **41.**

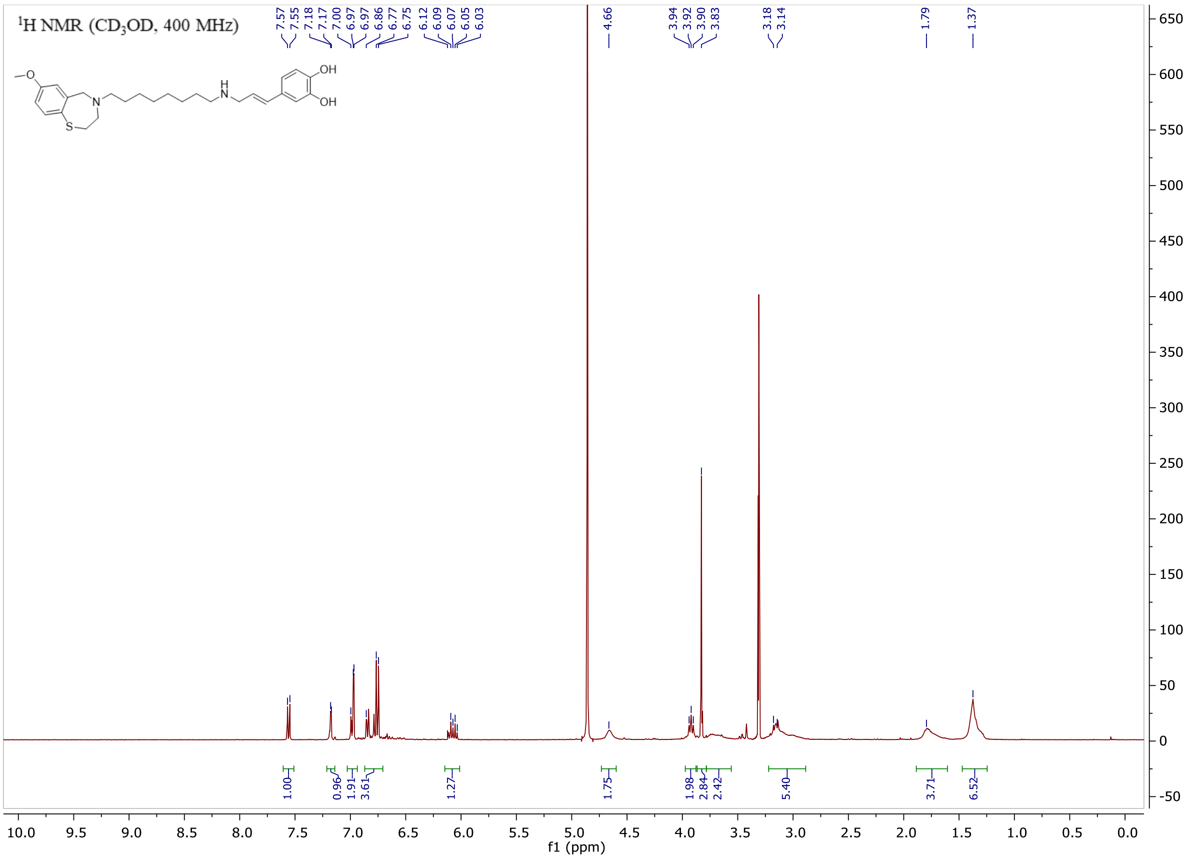

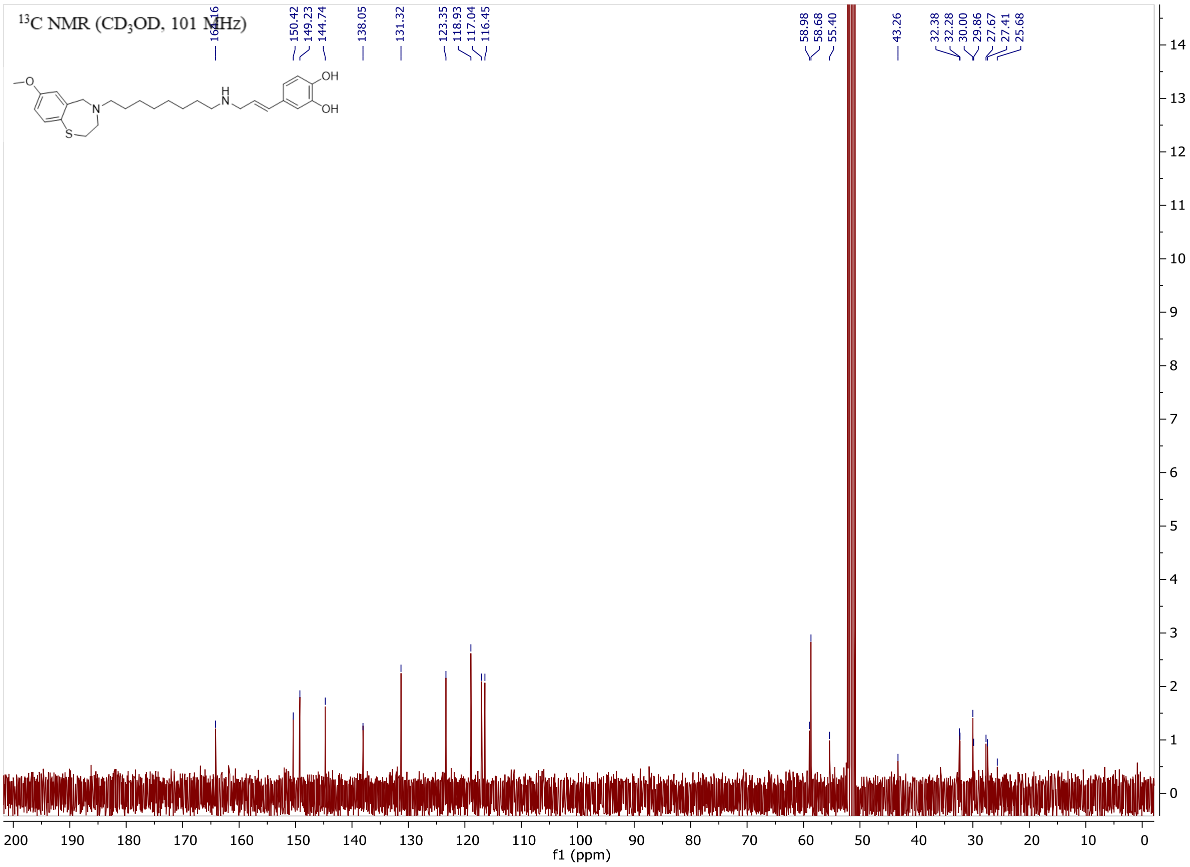

Figure S46. ¹H and ¹³C spectra of compound **9.**

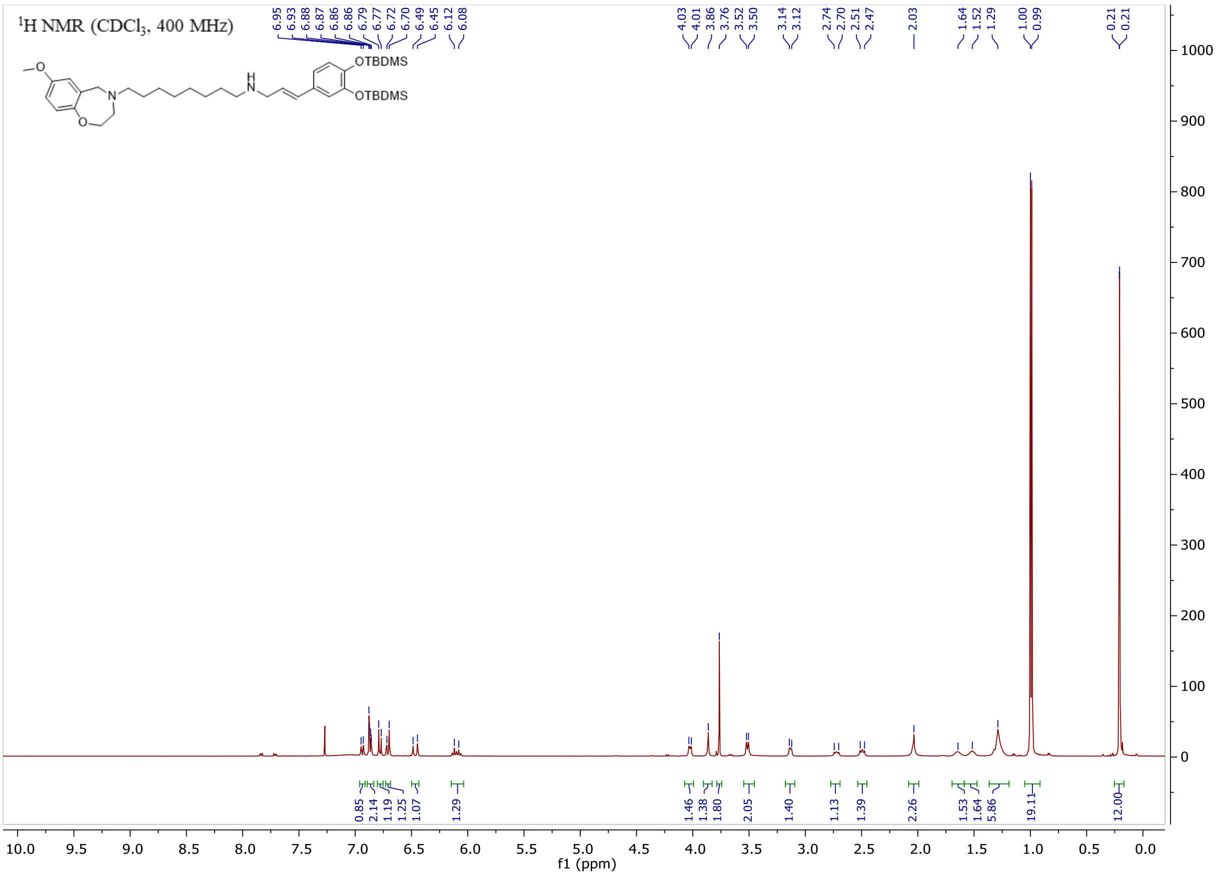

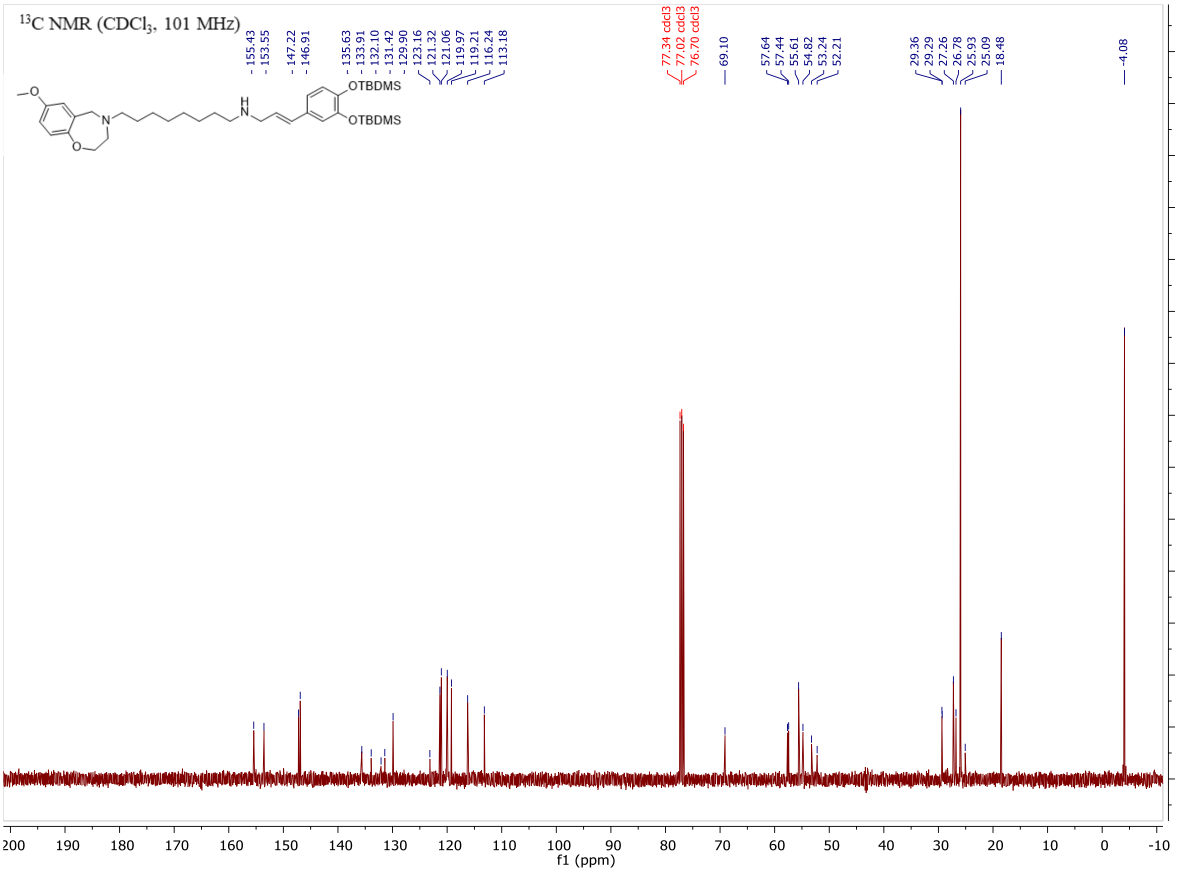

Figure S47. ^{1}H and ^{13}C spectra of compound **42.**

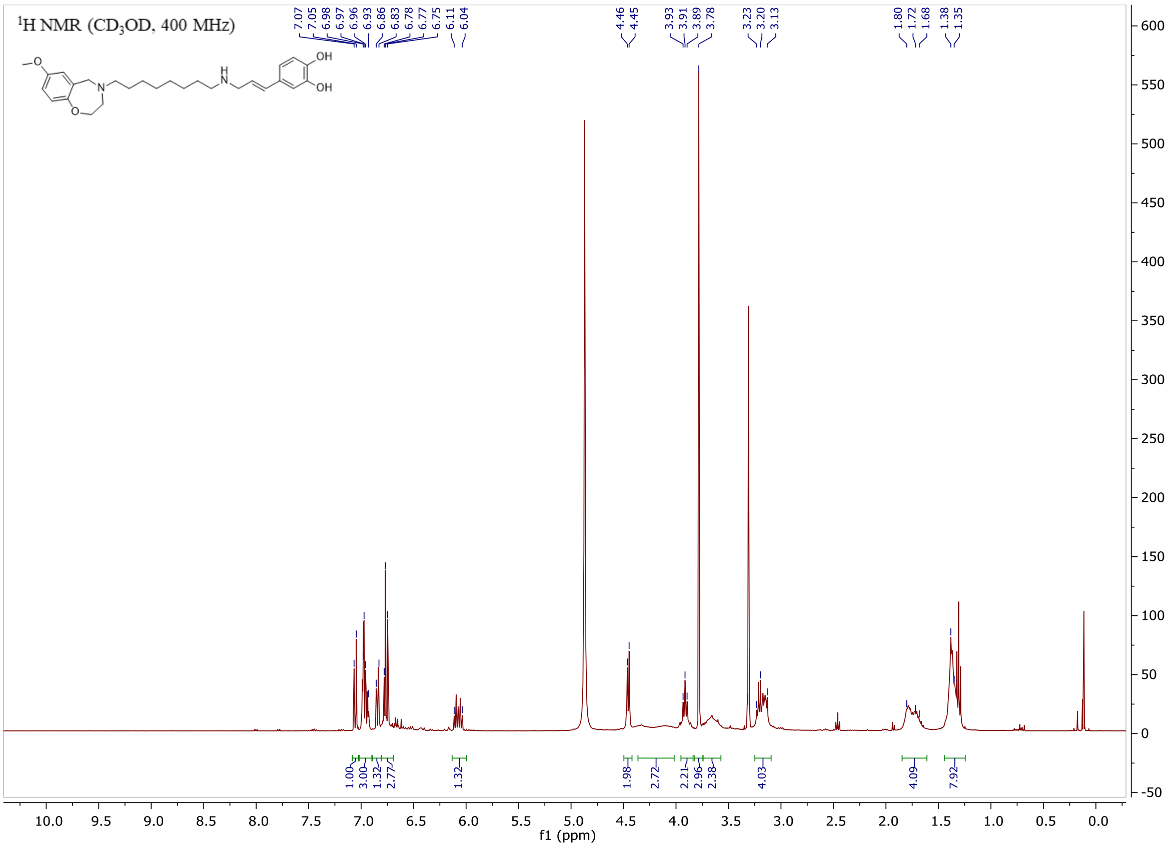

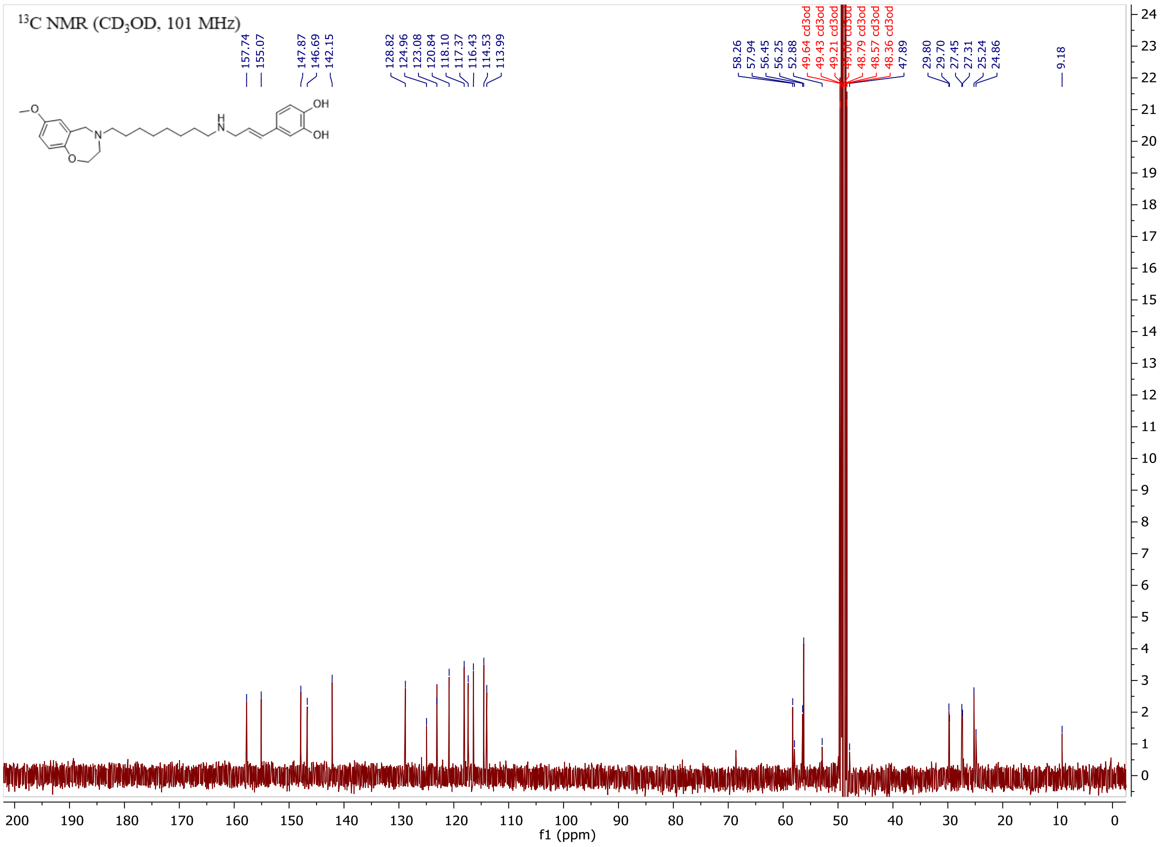

Figure S48. ^{1}H and ^{13}C spectra of compound **10.**

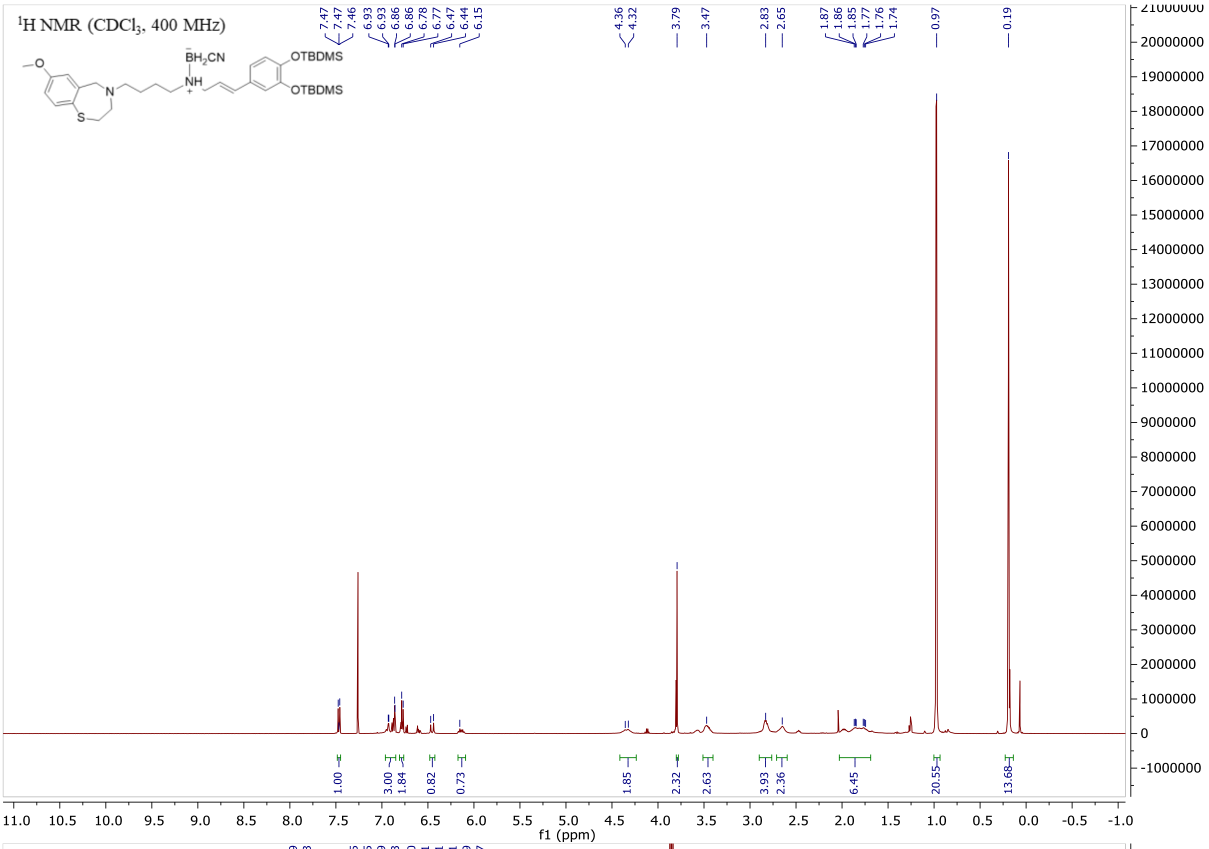

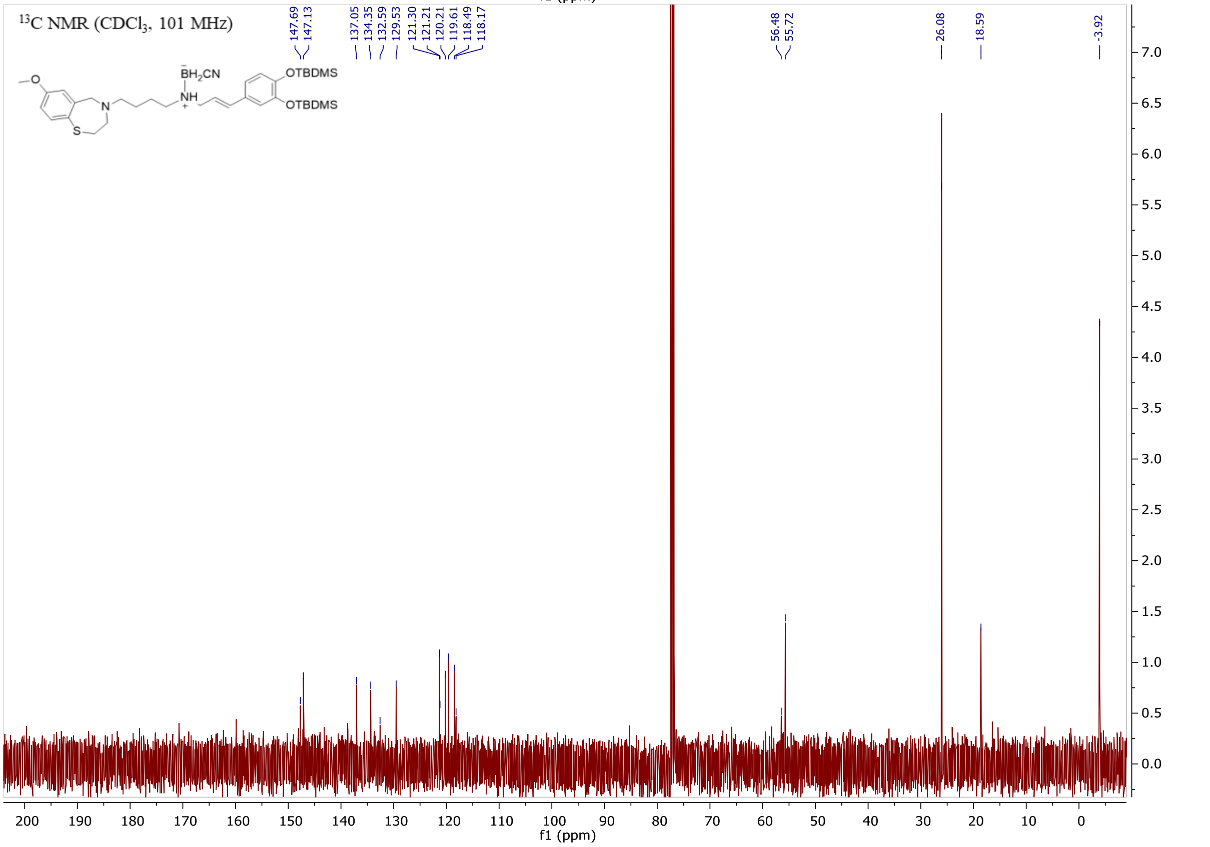

Figure S49. ^{1}H and ^{13}C spectra of compound **43.**

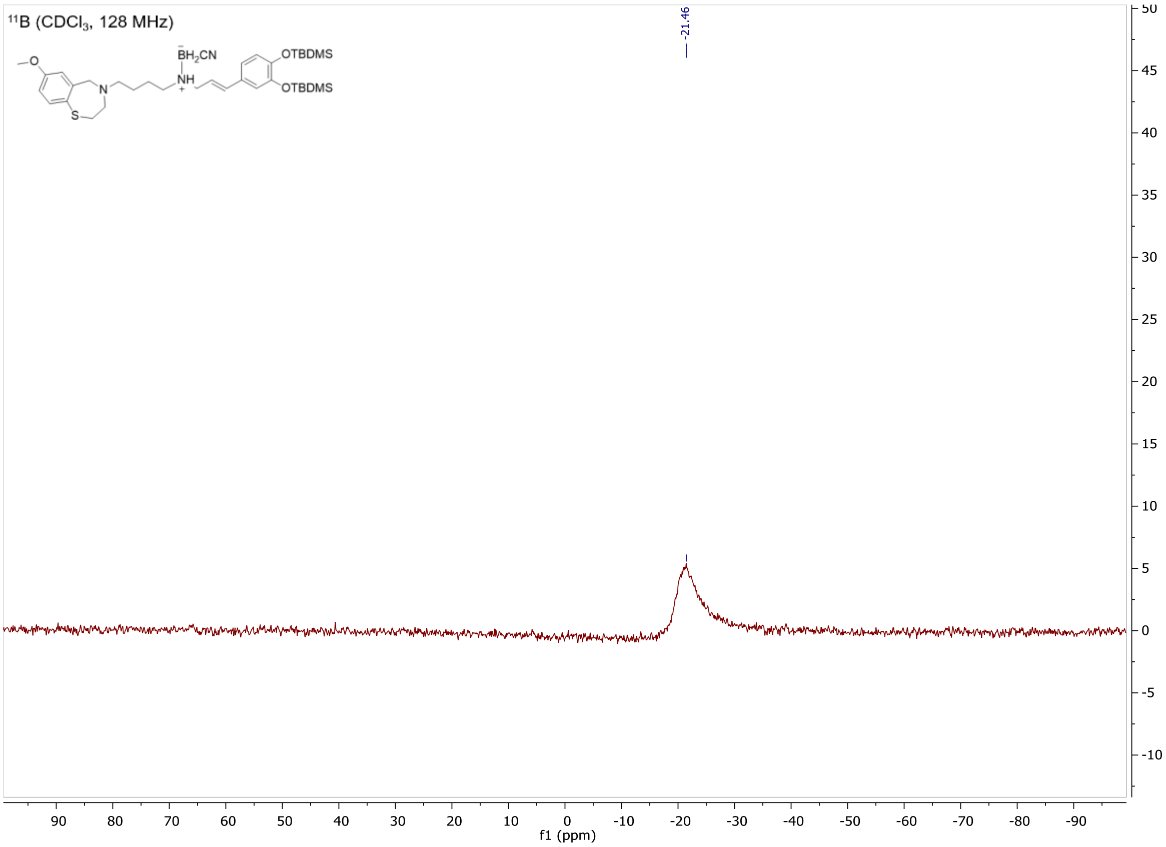

Figure S50. ¹¹B spectrum of compound **43.**

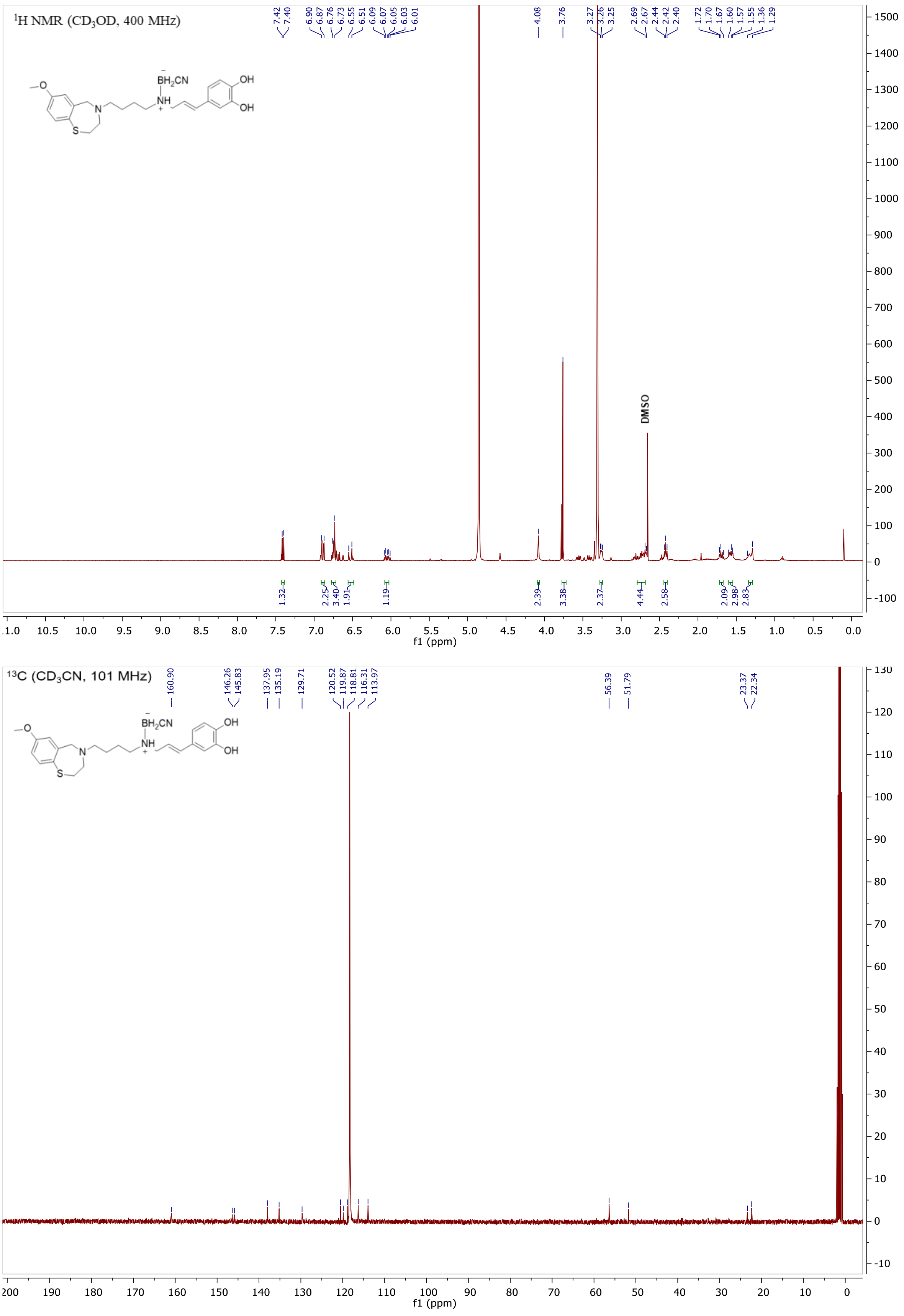

Figure S51. ^{1}H and ^{13}C spectra of compound **43.**

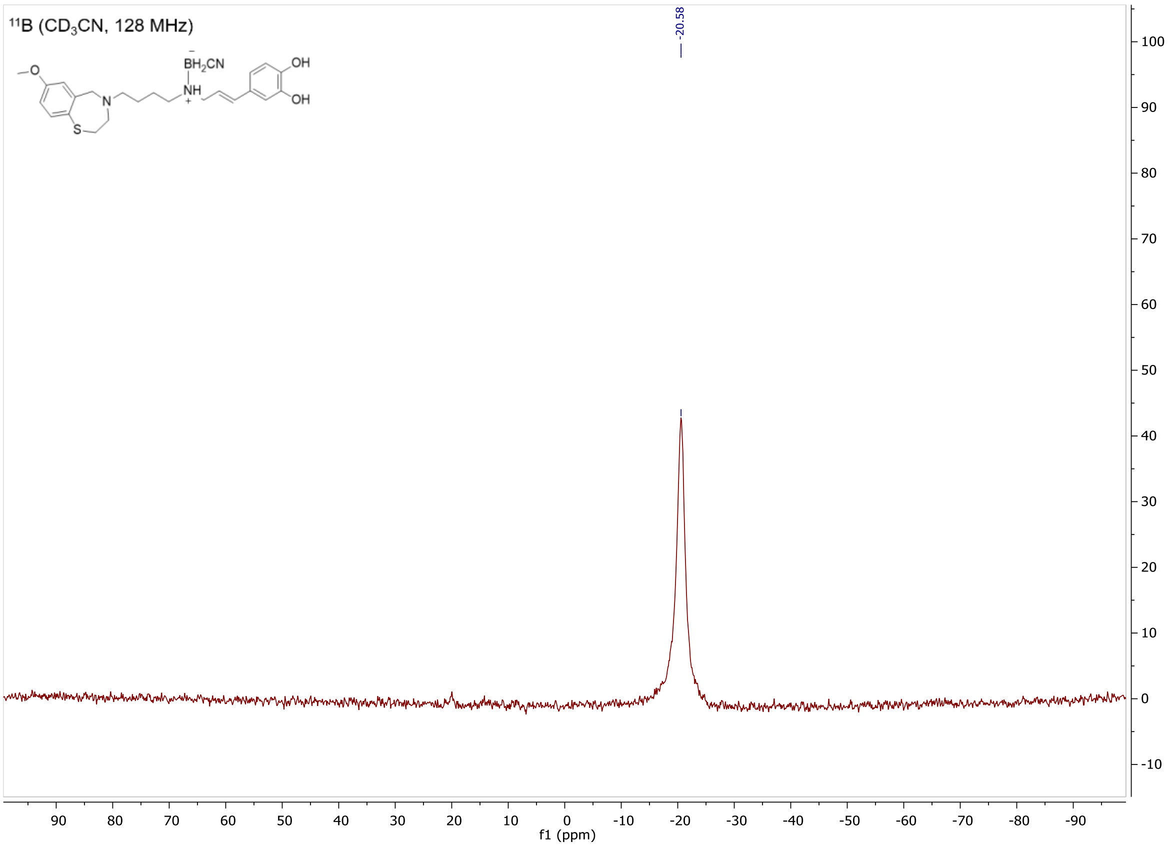

Figure S52. ^{11}B spectrum of compound **11.**

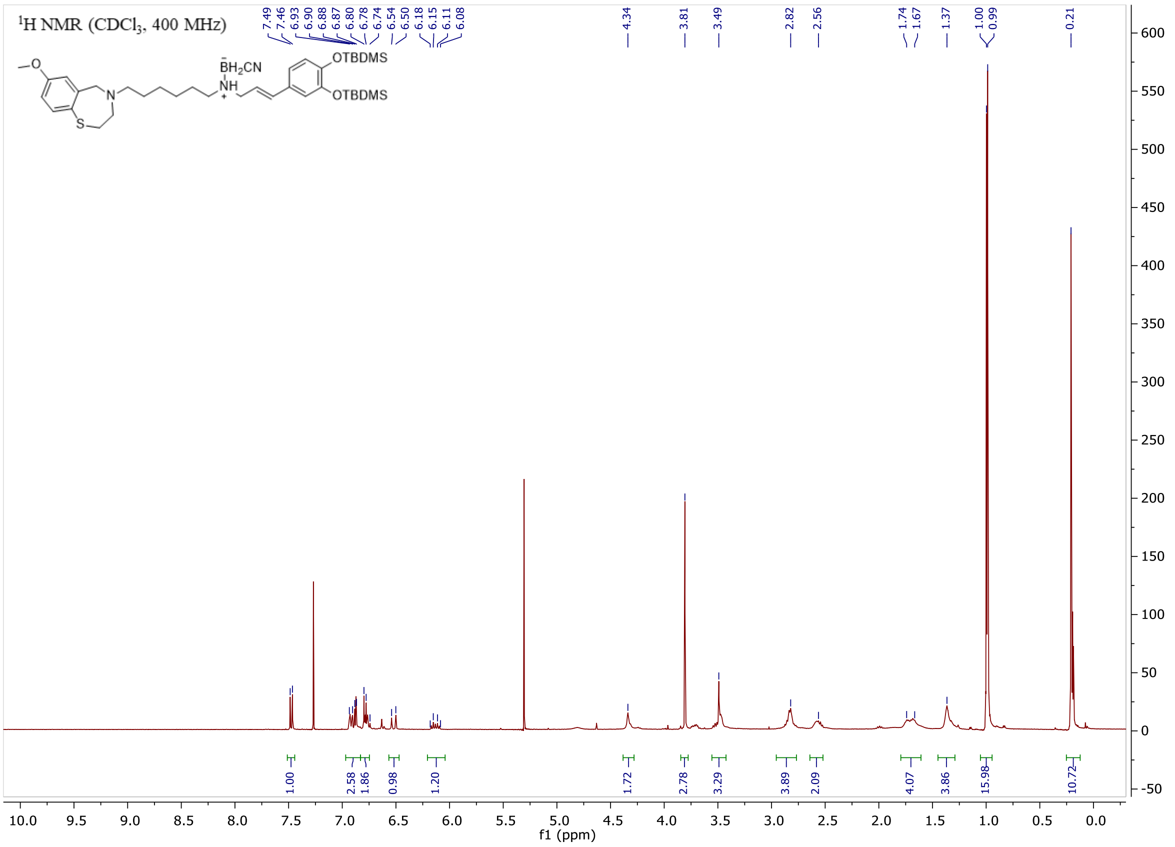

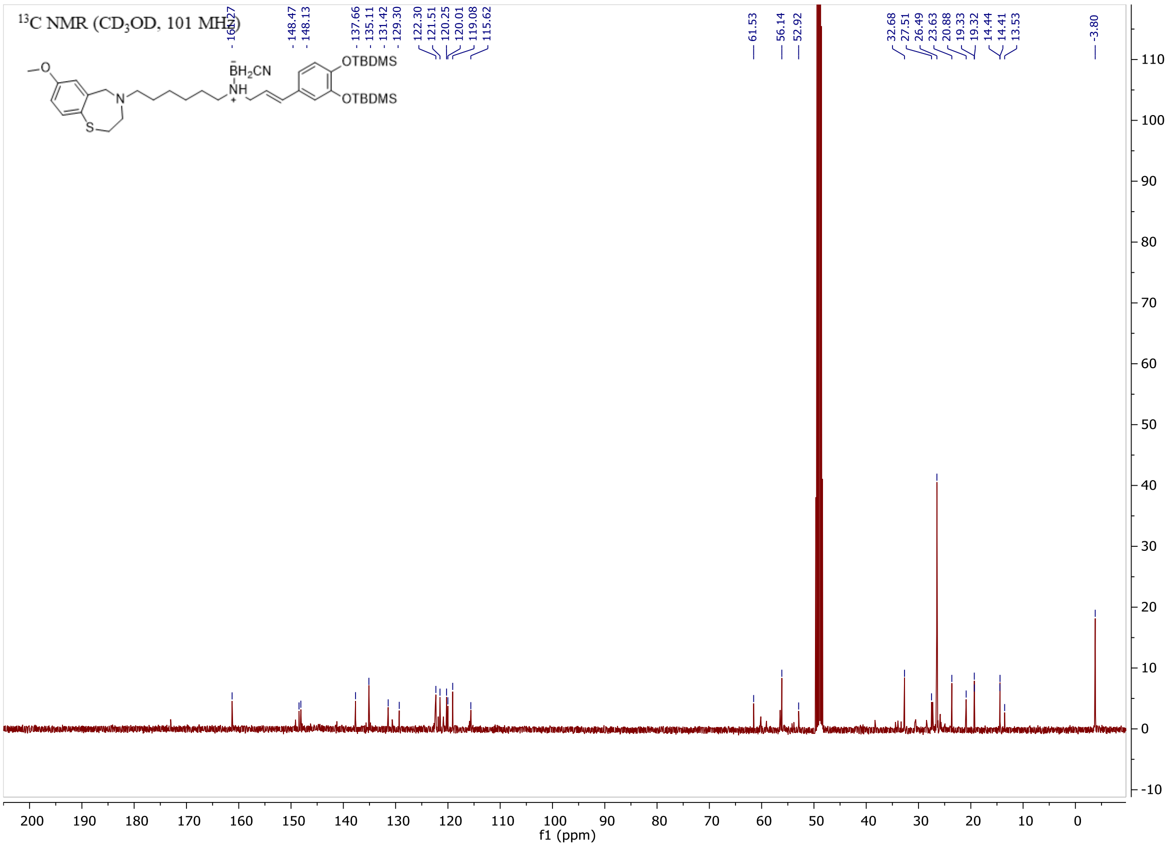

Figure S53. 1H and 13C spectra of compound **44.**

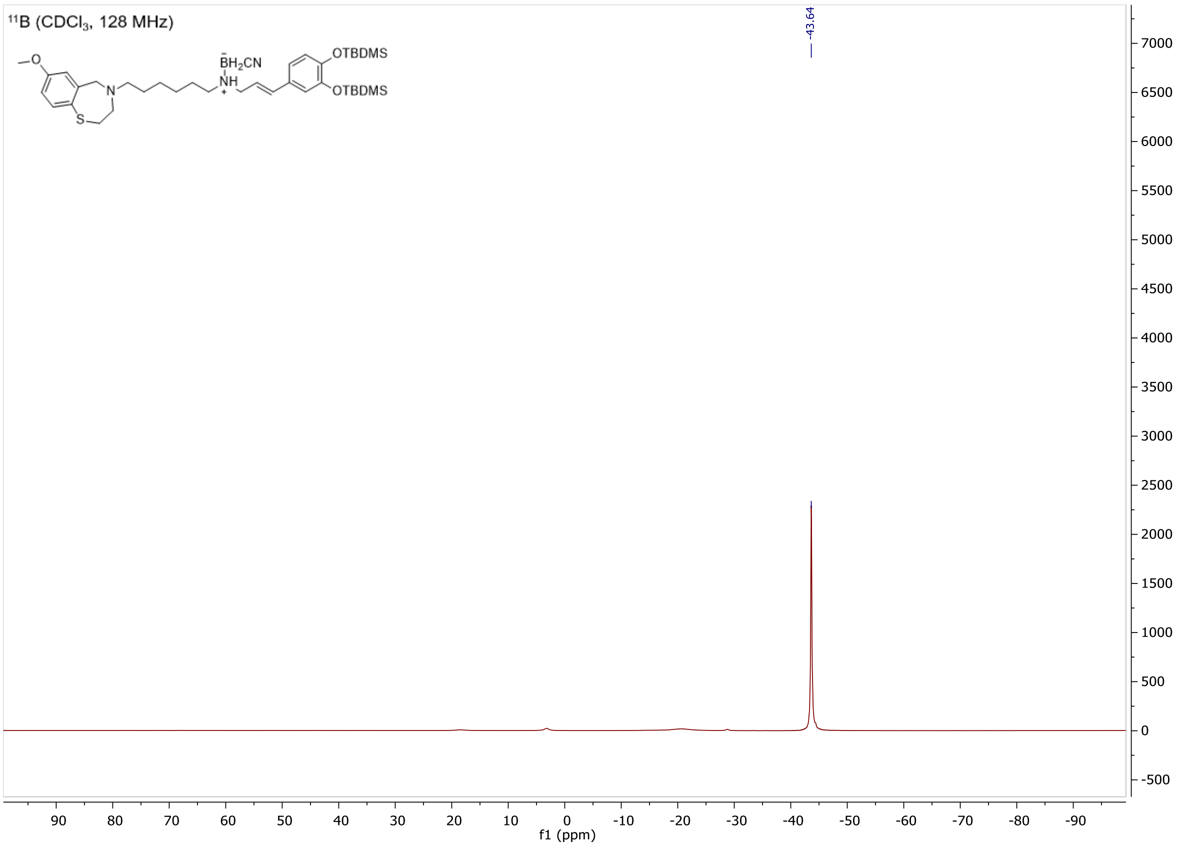

Figure S54. ^{11}B spectrum of compound **44.**

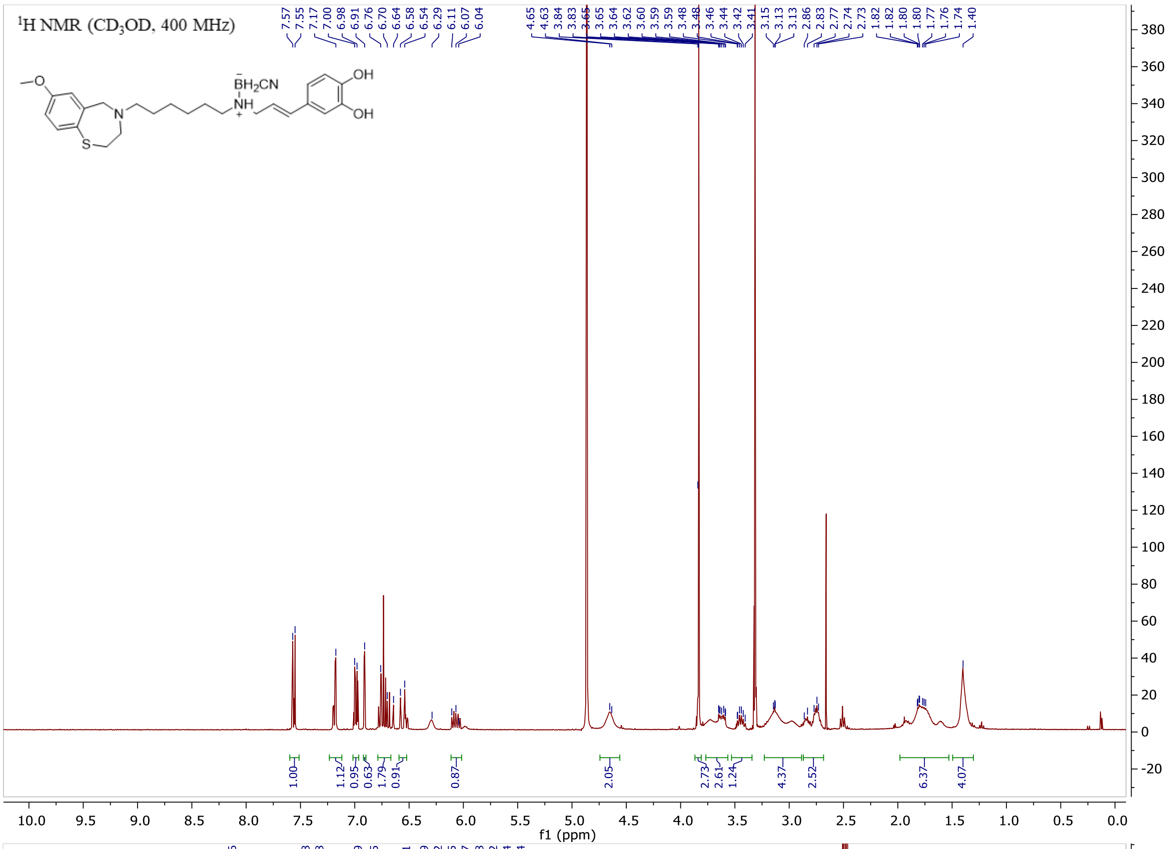

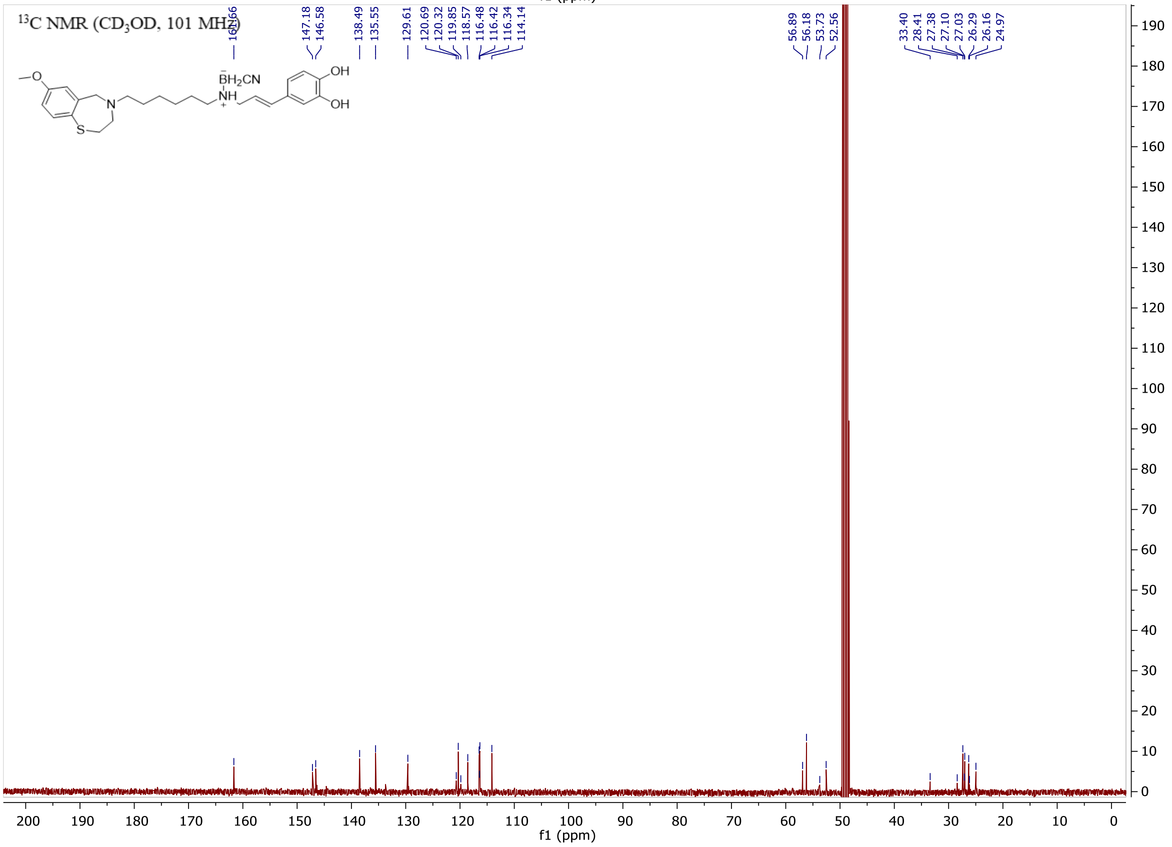

Figure S55. ^{1}H and ^{13}C spectra of compound **12.**

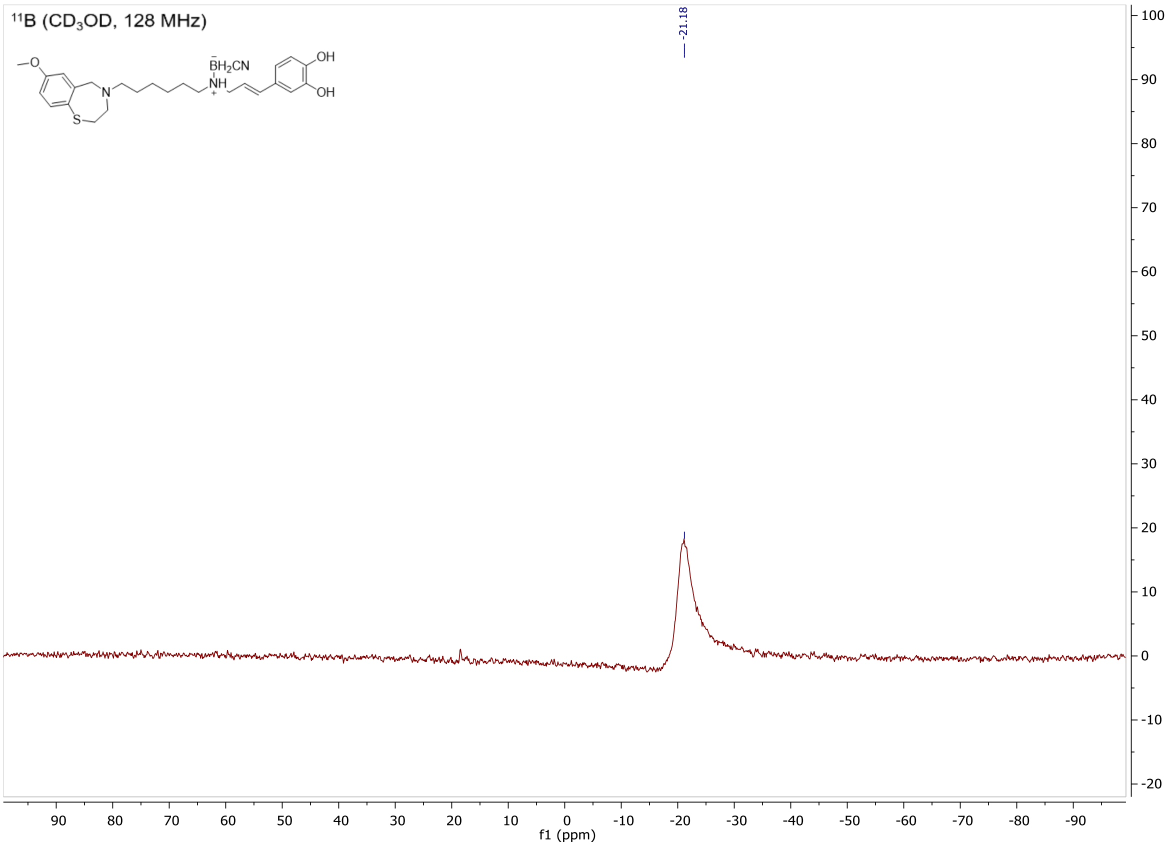

Figure S56. ^{11}B spectrum of compound **12.**

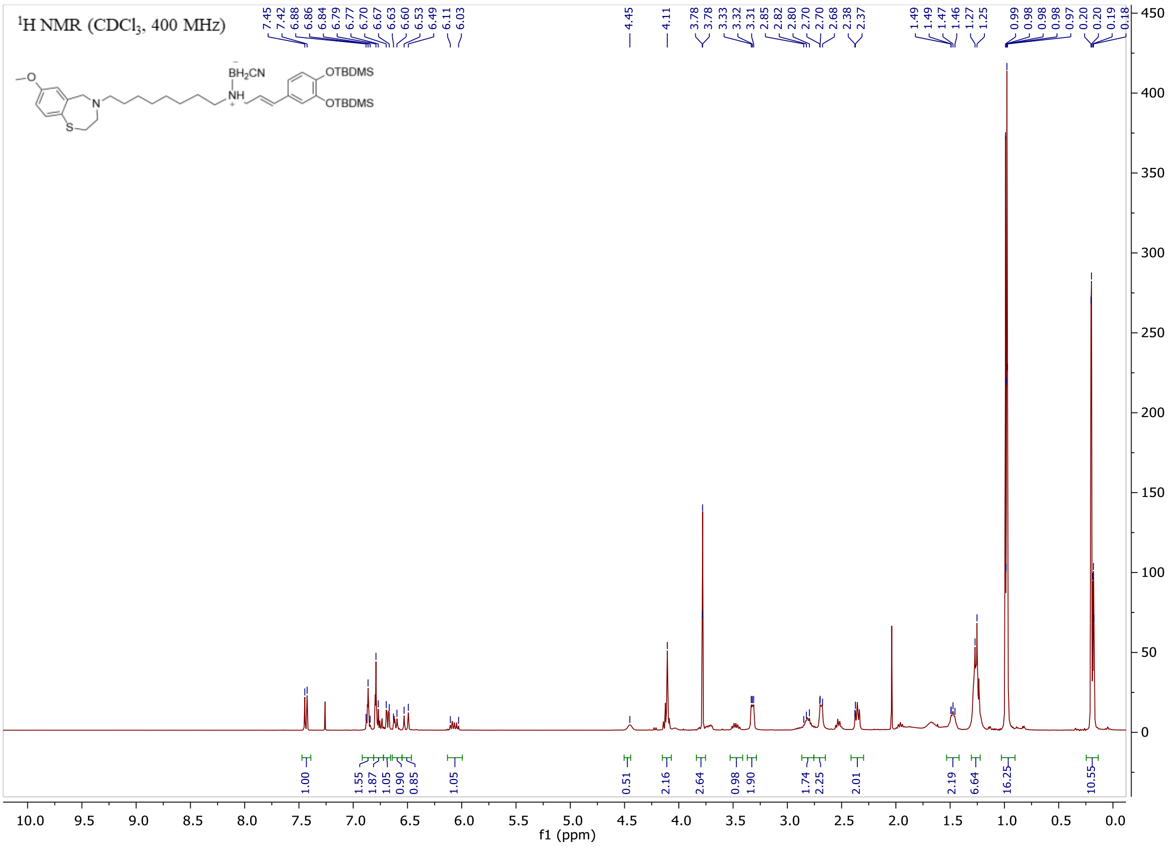

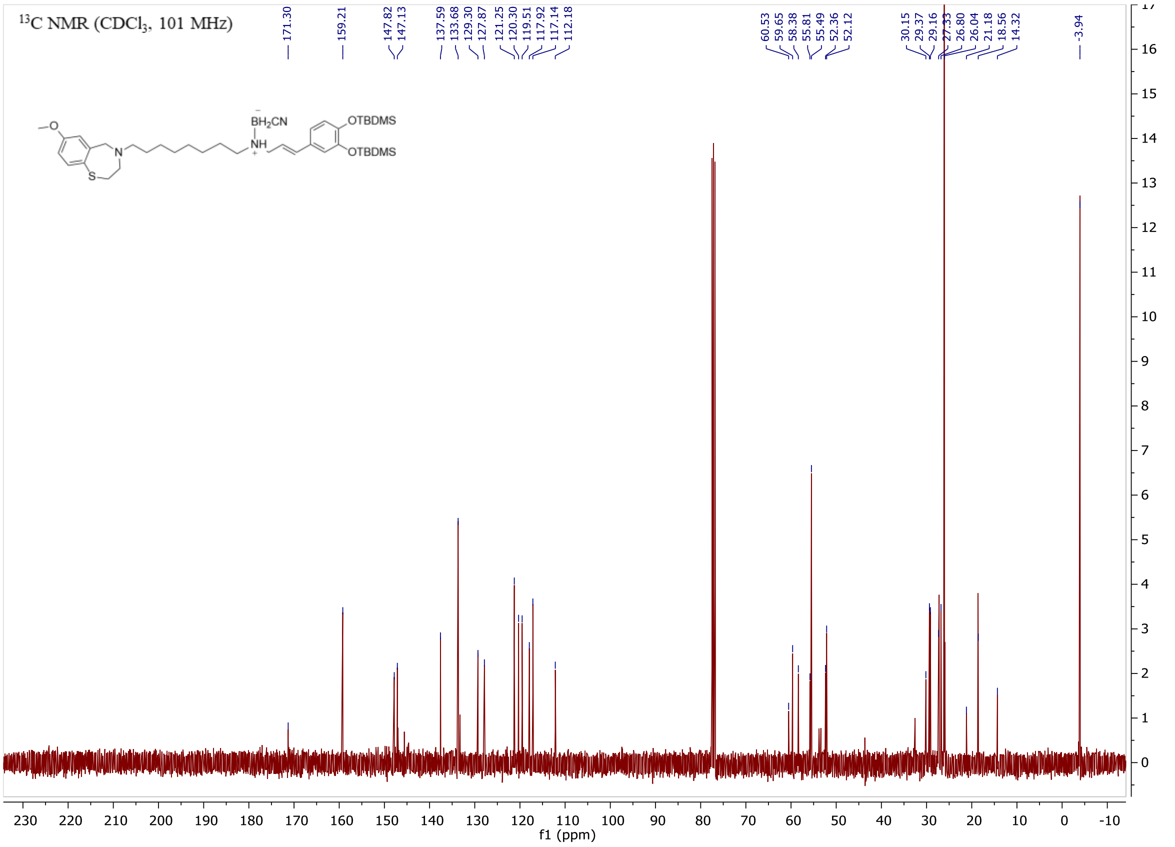

Figure S57. ^{1}H and ^{13}C spectra of compound **45.**

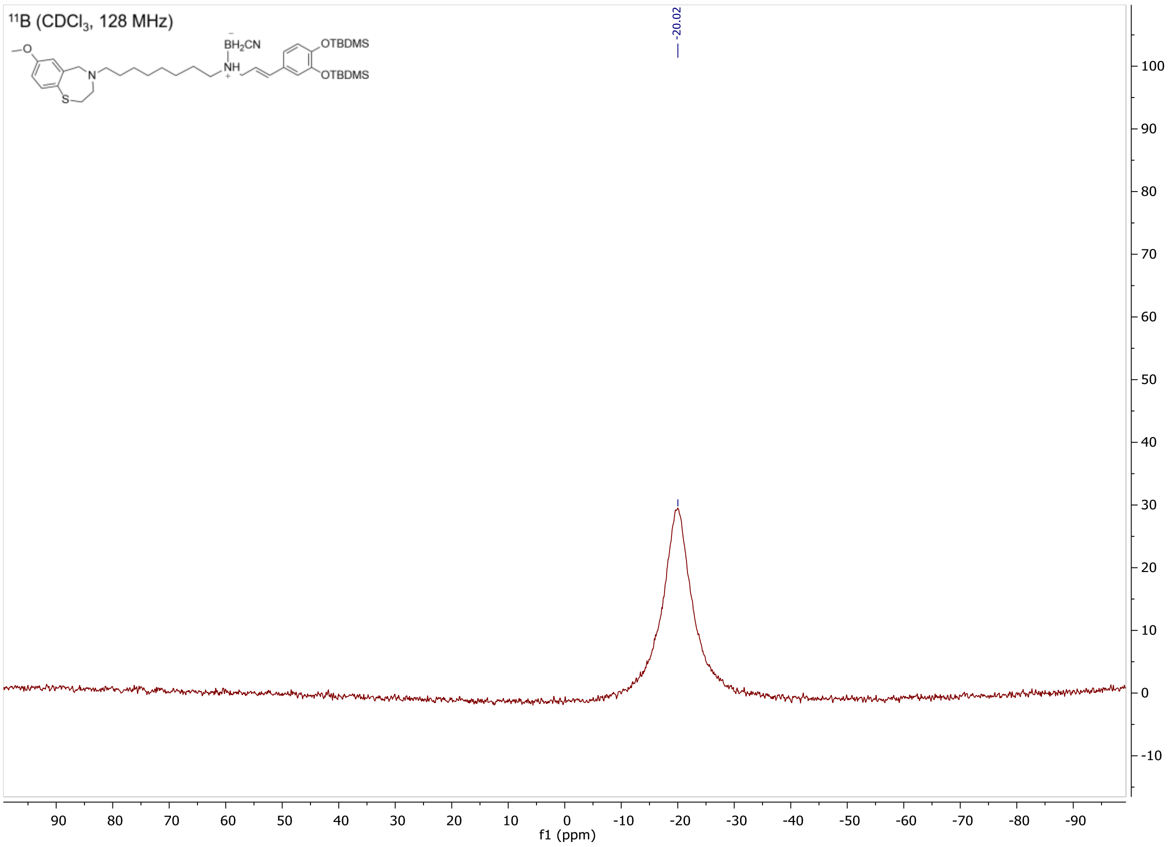

Figure S58. ^{11}B spectrum of compound **46.**

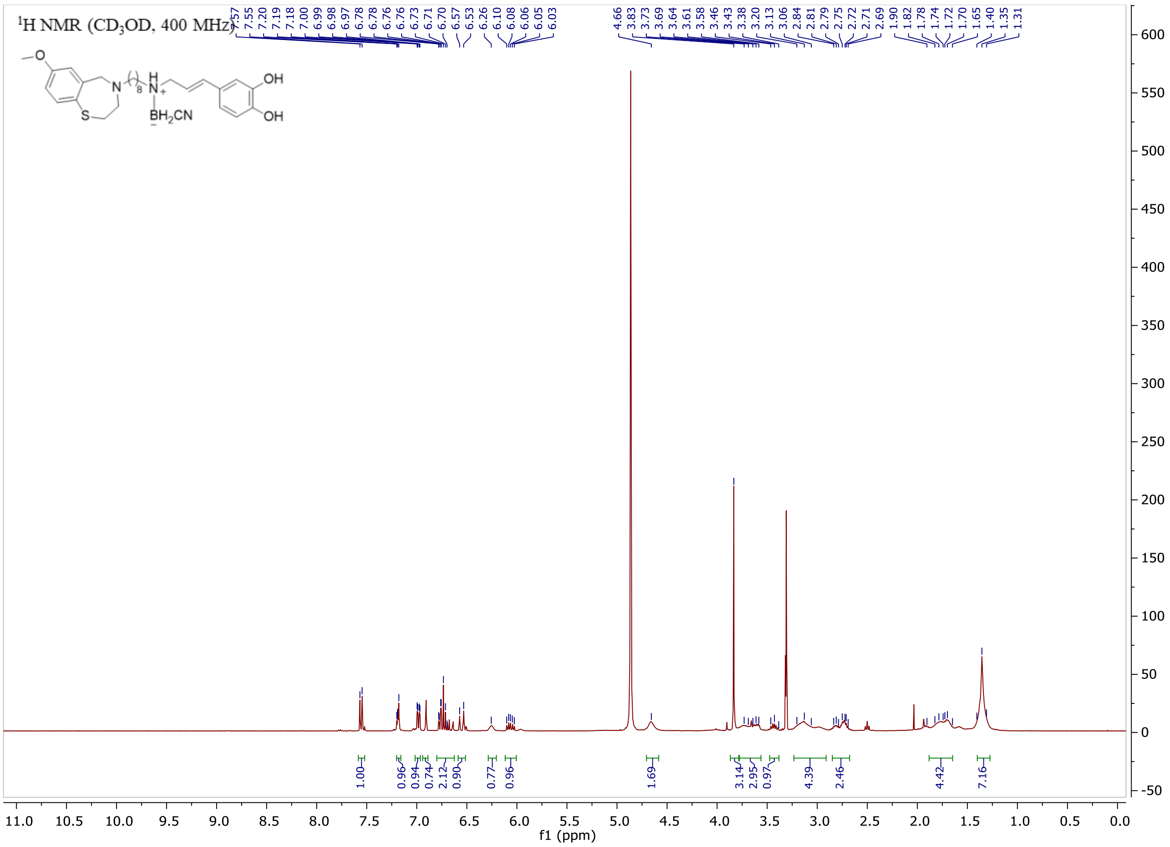

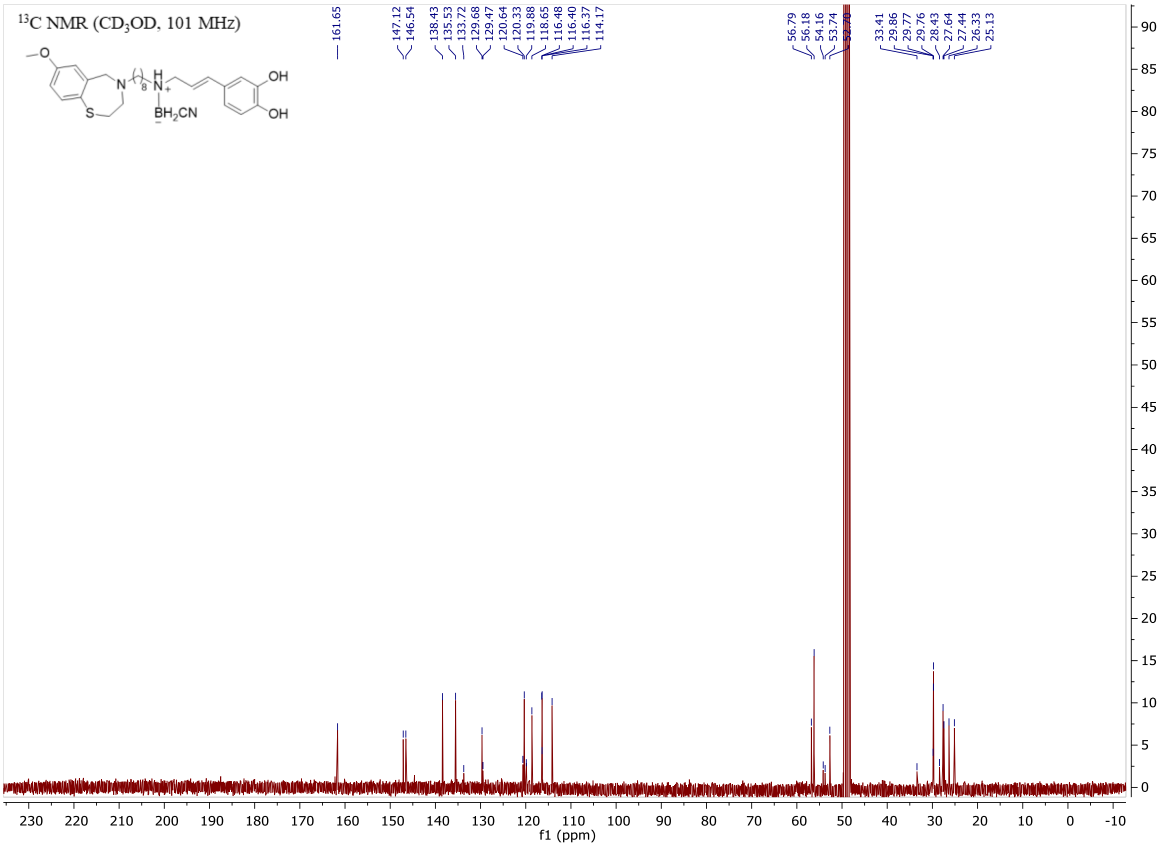

Figure S59. ^{1}H and ^{13}C spectra of compound **13.**

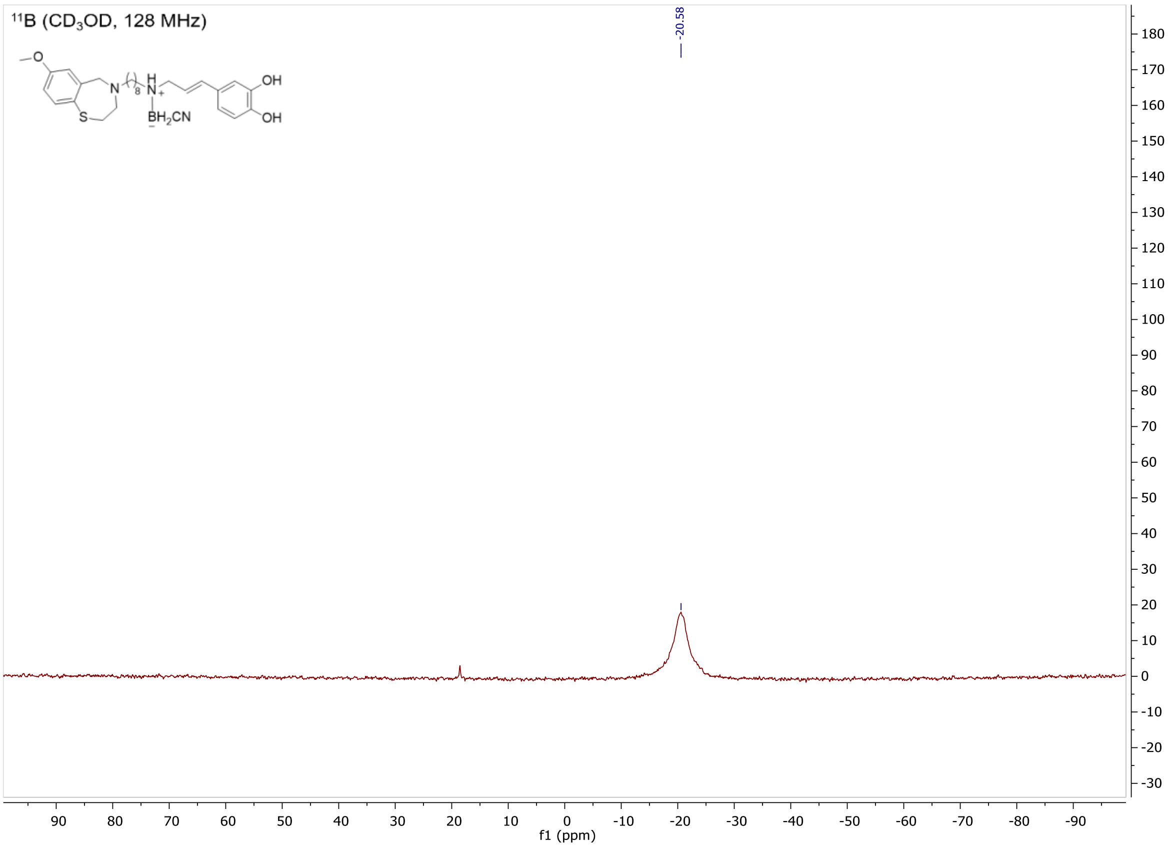

Figure S60. ^{11}B spectrum of compound **13.**

HPLC TRACES

Analytical HPLC was performed with Knauer AZURA liquid chromatography system. Analytical column: Knauer Eurosphere II 100-5, C18-H, 5 µm, 150×4.6 mm (unless stated otherwise); solvent A: H_2O + 0.1% v/v TFA, solvent B: MeCN + 0.1% v/v TFA; temperature 25 °C.

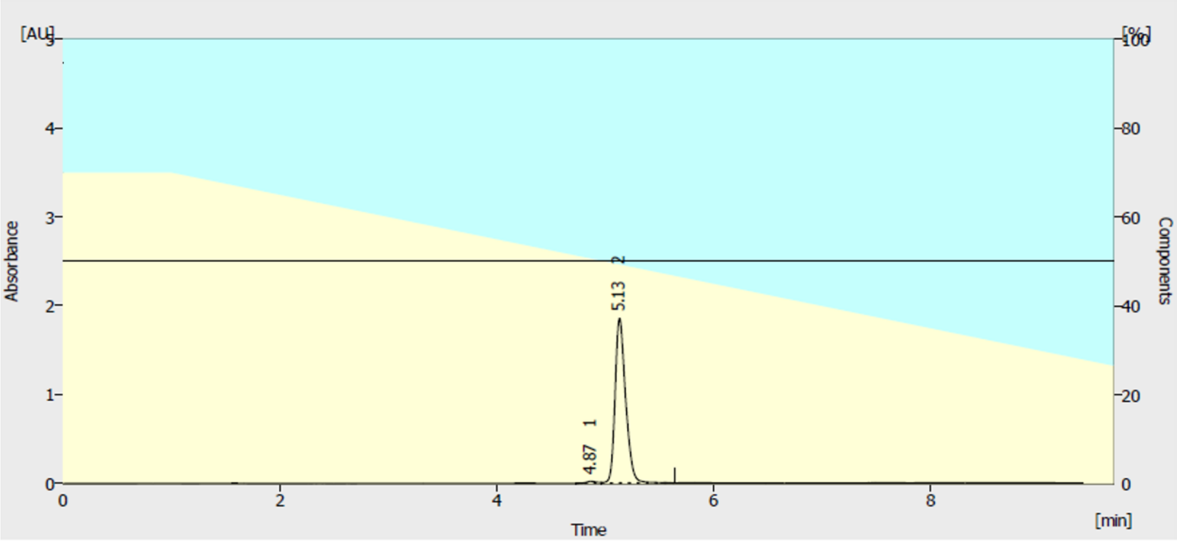

	Reten. Time [min]	Area [mAU.s]	Height [mAU]	Area [%]	Height [%]	W05 [min]	PDA Peak Purity	Compound Name
1	4.868	132.144	20.548	1.0	1.1	0.11	978	
2	5.132	12498.417	1853.622	99.0	98.9	0.10	853	
	Total	12630.561	1874.171	100.0	100.0			

Figure S61. HPLC trace of compound **1**. t_R = 5.1 min, 99 % (B/A: 30/70 → 100/0 in 14 min, flow 1.2 ml/min, 254 nm).

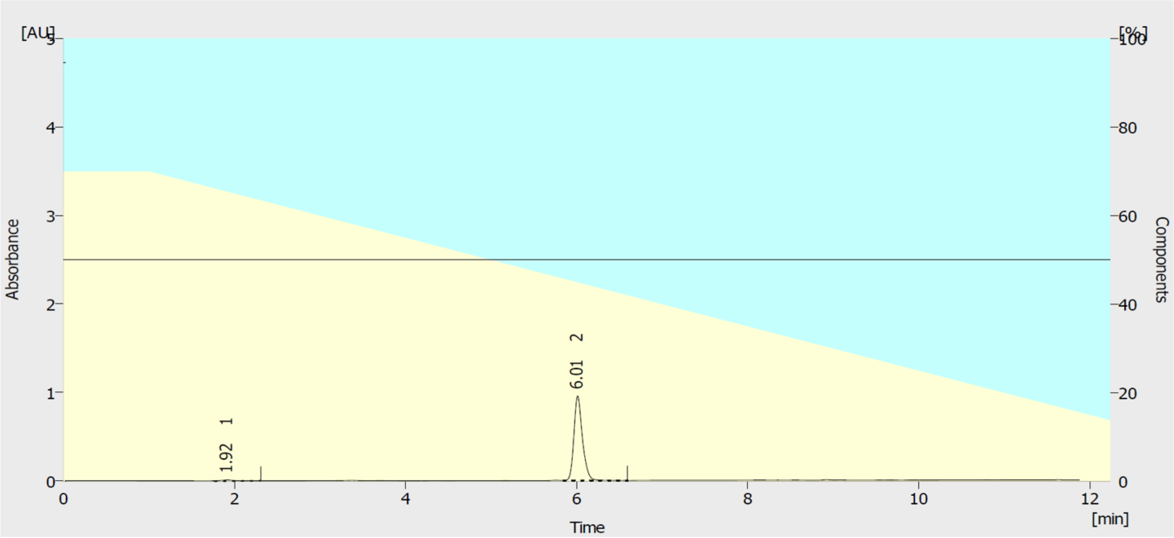

	Reten. Time [min]	Area [mAU.s]	Height [mAU]	Area [%]	Height [%]	W05 [min]	PDA Peak Purity	Compound Name
1	1.917	1873.126	13.531	22.1	1.4	0.19	446	
2	6.012	6597.418	955.554	77.9	98.6	0.10	652	
	Total	8470.545	969.084	100.0	100.0			

Figure S62. HPLC trace of compound **2.** t_R = 6.0 min, 99 % (B/A: 30/70 → 100/0 in 14 min, flow 1.2 ml/min, 254 nm).

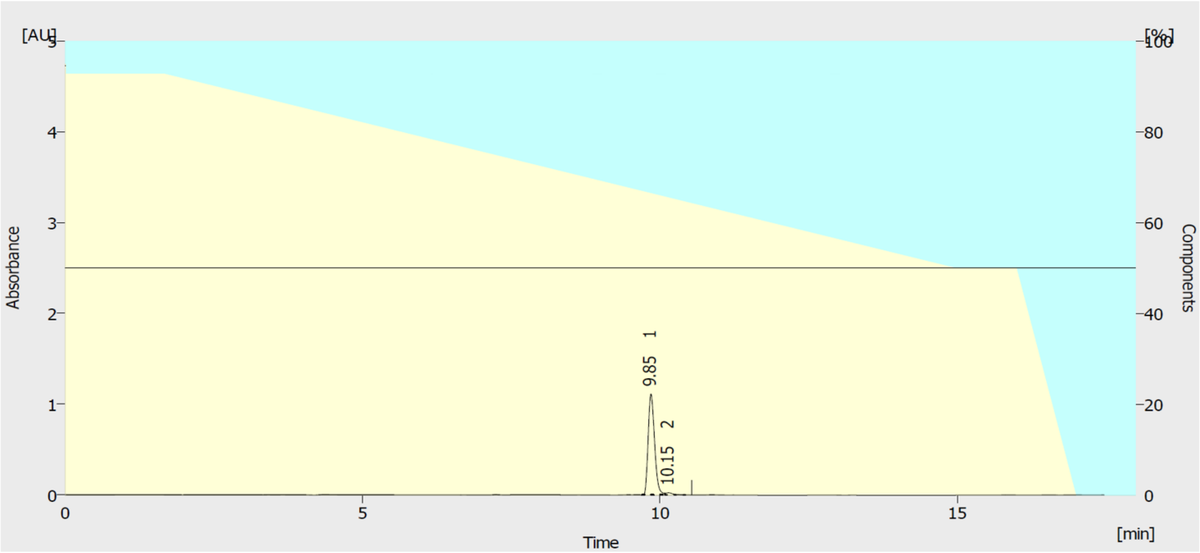

	Reten. Time [min]	Area [mAU.s]	Height [mAU]	Area [%]	Height [%]	W05 [min]	PDA Peak Purity	Compound Name
1	9.850	8276.871	1111.757	97.6	97.9	0.12	878	
2	10.145	201.914	23.550	2.4	2.1	0.13	978	
	Total	8478.786	1135.307	100.0	100.0			

Figure S63. HPLC trace of compound **3**. t_R = 9.9 min, 99 % (B/A: 10/90 → 50/50 in 14 min, flow 1.2 ml/min, 254 nm).

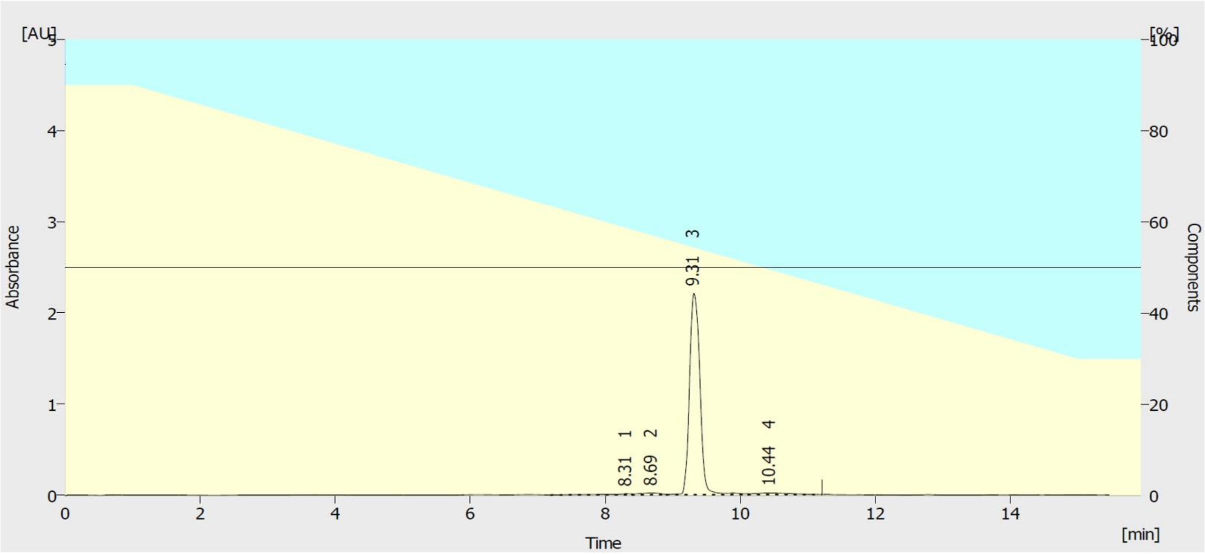

	Reten. Time [min]	Area [mAU.s]	Height [mAU]	Area [%]	Height [%]	W05 [min]	PDA Peak Purity	Compound Name
1	8.310	306.051	12.564	1.2	0.6	0.17	963	
2	8.690	414.594	19.277	1.7	0.9	0.38	995	
3	9.313	23775.598	2208.328	94.8	97.8	0.17	719	
4	10.442	593.678	16.786	2.4	0.7	0.62	959	
	Total	25089.921	2256.955	100.0	100.0			

Figure S64. HPLC trace of compound **4**. t_R = 9.3 min, 98 % (B/A: 10/90 → 70/30 in 14 min, flow 1.2 ml/min, 254 nm).

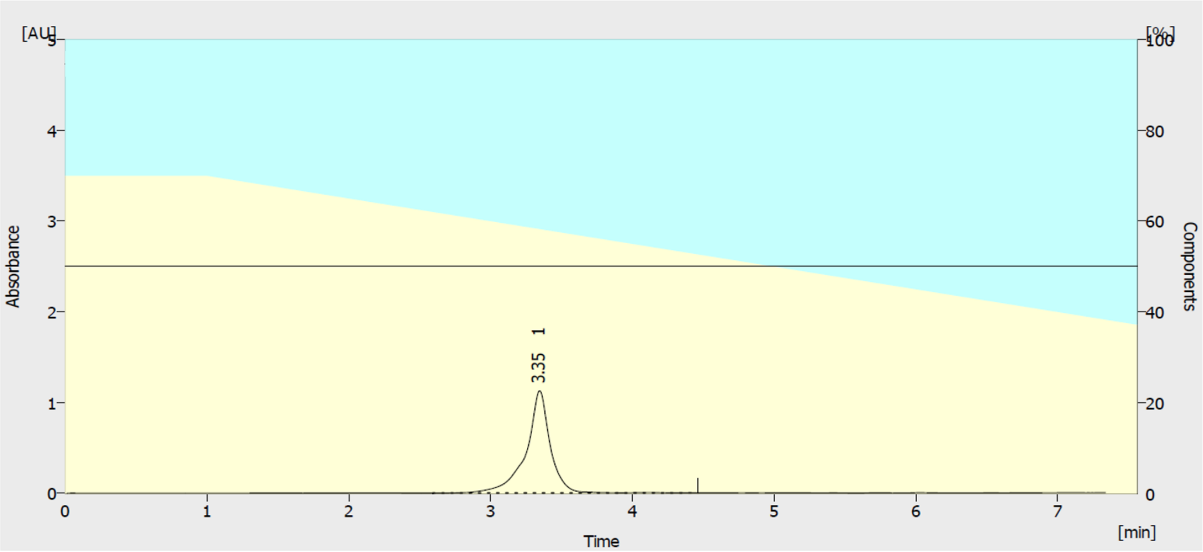

	Reten. Time [min]	Area [mAU.s]	Height [mAU]	Area [%]	Height [%]	W05 [min]	PDA Peak Purity	Compound Name
1	3.347	13340.689	1126.717	100.0	100.0	0.14	691	
	Total	13340.689	1126.717	100.0	100.0			

Figure S65. HPLC trace of compound **5.** t_R = 3.3 min, 100 % (B/A: 30/70 → 100/0 in 14 min, flow 1.2 ml/min, 254 nm).

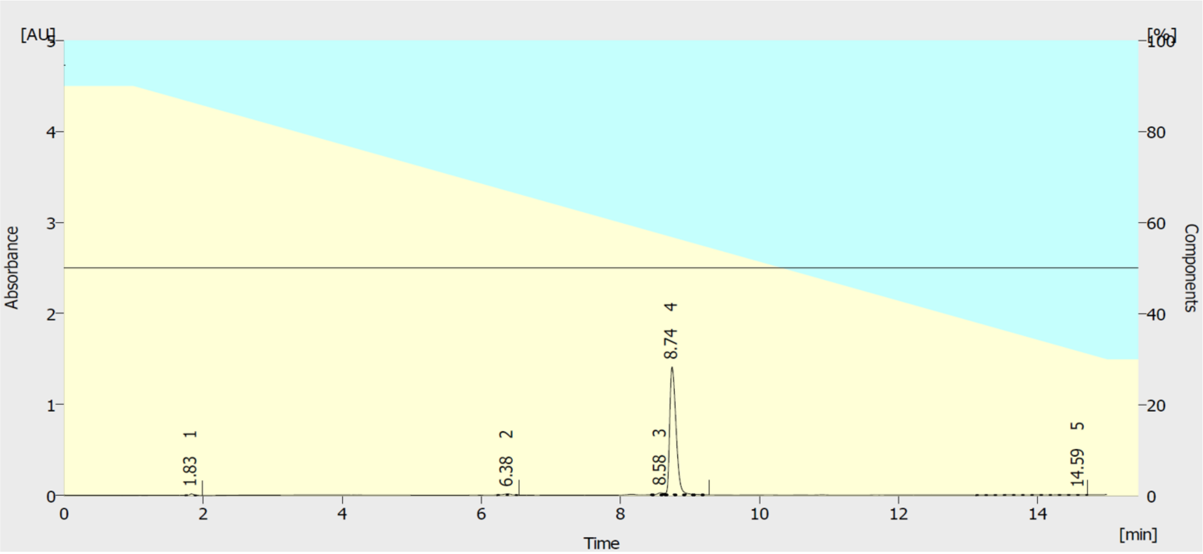

	Reten. Time [min]	Area [mAU.s]	Height [mAU]	Area [%]	Height [%]	W05 [min]	PDA Peak Purity	Compound Name
1	1.903	26.495	5.253	0.3	0.5	0.07	970	
2	6.403	7745.955	1108.742	98.1	98.6	0.11	667	
3	7.203	69.174	5.563	0.9	0.5	0.18	997	
4	7.560	57.864	5.252	0.7	0.5	0.19	991	
	Total	7899.488	1124.810	100.0	100.0			

Figure S66. HPLC trace of compound **6.** t_R = 8.7 min, 98 % (B/A: 10/90 → 70/30 in 14 min, flow 1.2 ml/min, 254 nm).

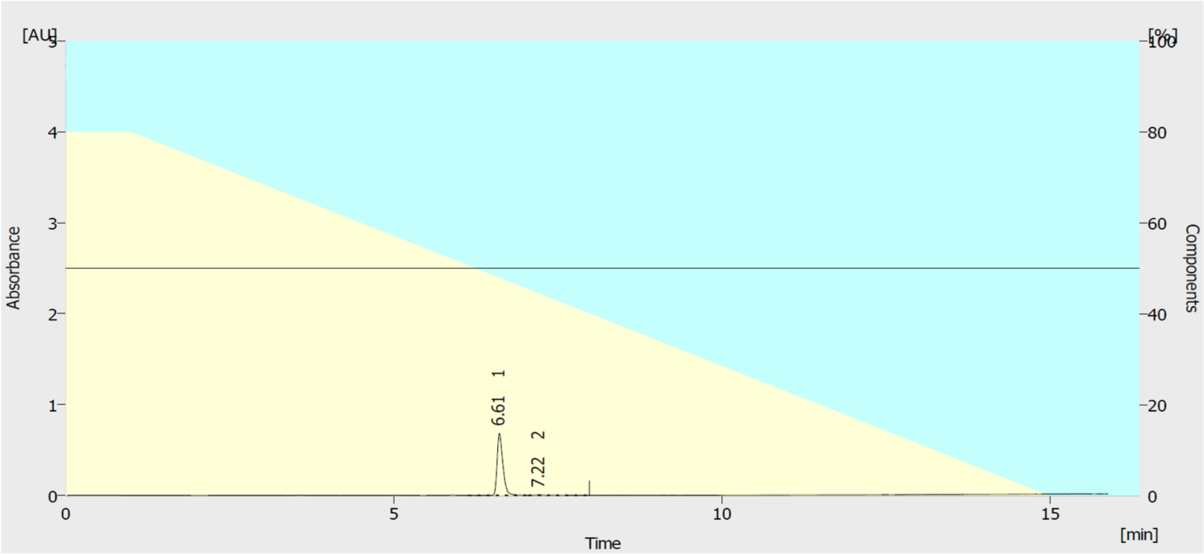

Figure S67. HPLC trace of compound **7.** t_R = 8.7 min, 99 % (B/A: 20/80 → 70/30 in 14 min, flow 1.2 ml/min, 254 nm).

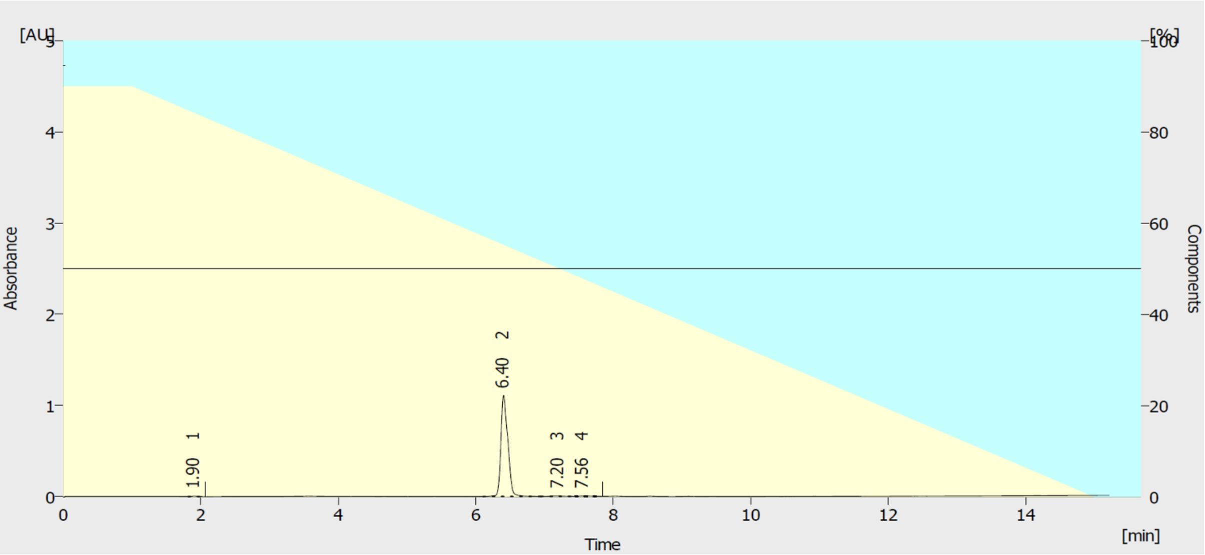

	Reten. Time [min]	Area [mAU.s]	Height [mAU]	Area [%]	Height [%]	W05 [min]	PDA Peak Purity	Compound Name
1	1.903	26.495	5.253	0.3	0.5	0.07	970	
2	6.403	7745.955	1108.742	98.1	98.6	0.11	667	
3	7.203	69.174	5.563	0.9	0.5	0.18	997	
4	7.560	57.864	5.252	0.7	0.5	0.19	991	
	Total	7899.488	1124.810	100.0	100.0			

Figure S68. HPLC trace of compound **8.** t_R = 6.4 min, 98 % (B/A: 10/90 → 100/0 in 14 min, flow 1.2 ml/min, 254 nm).

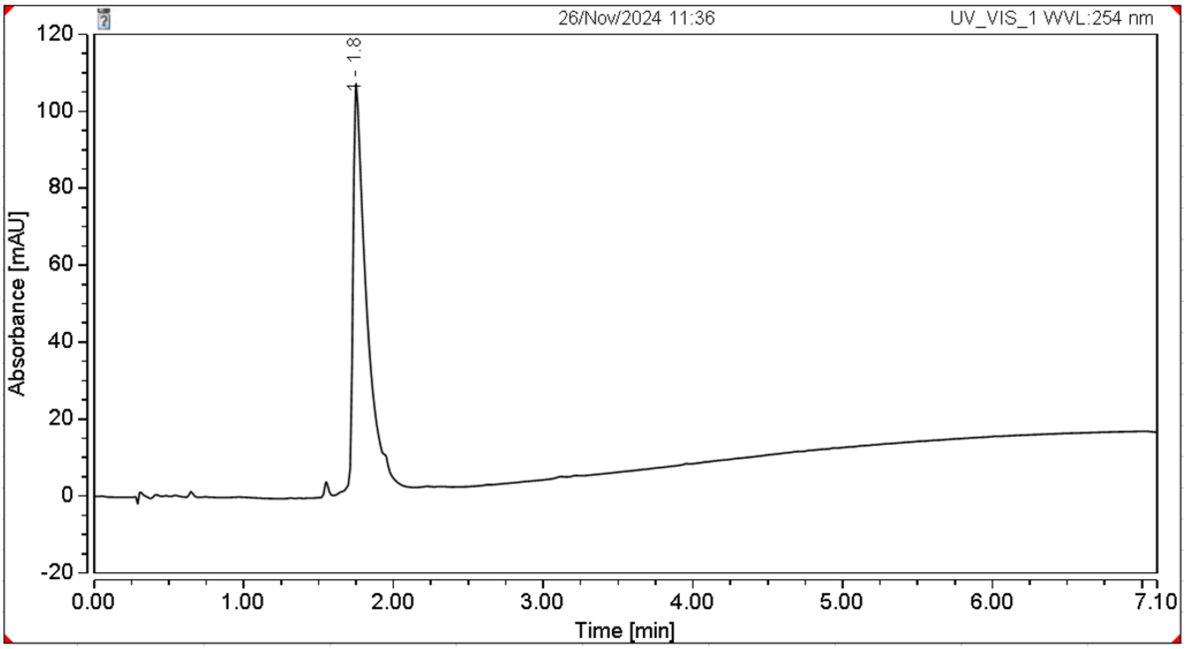

Figure S69. HPLC trace of compound **9.** Analytical column: Phenomenex Kinetex C18, C18-H, 1.7 µm, 50×3 mm; solvent A: H_2O + 0.1% v/v FA, solvent B: MeCN + 0.1% v/v FA; temperature 25 °C. t_R = 1.8 min, 99 % (B/A: 20/80 → 100/0 in 4 min, flow 0.8 ml/min, 254 nm).

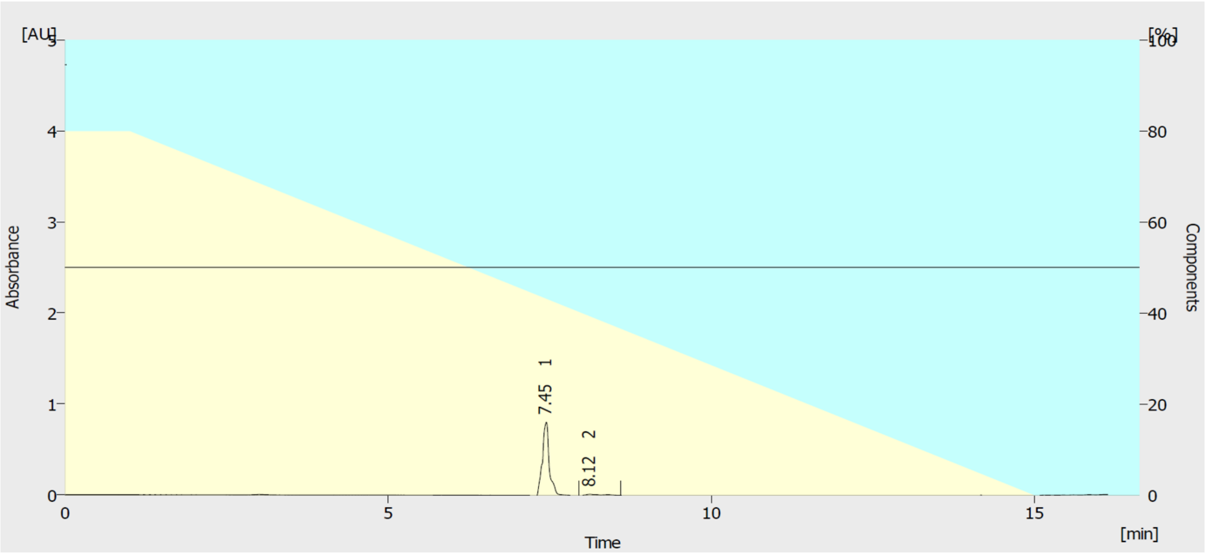

	Reten. Time [min]	Area [mAU.s]	Height [mAU]	Area [%]	Height [%]	W05 [min]	PDA Peak Purity	Compound Name
1	7.447	5991.304	804.446	95.9	98.2	0.09	599	
2	8.123	253.583	14.878	4.1	1.8	0.22	839	
	Total	6244.888	819.324	100.0	100.0			

Figure S70. HPLC trace of compound **10.** t_R = 7.5 min, 98 % (B/A: 20/80 → 100/0 in 14 min, flow 1.2 ml/min, 254 nm).

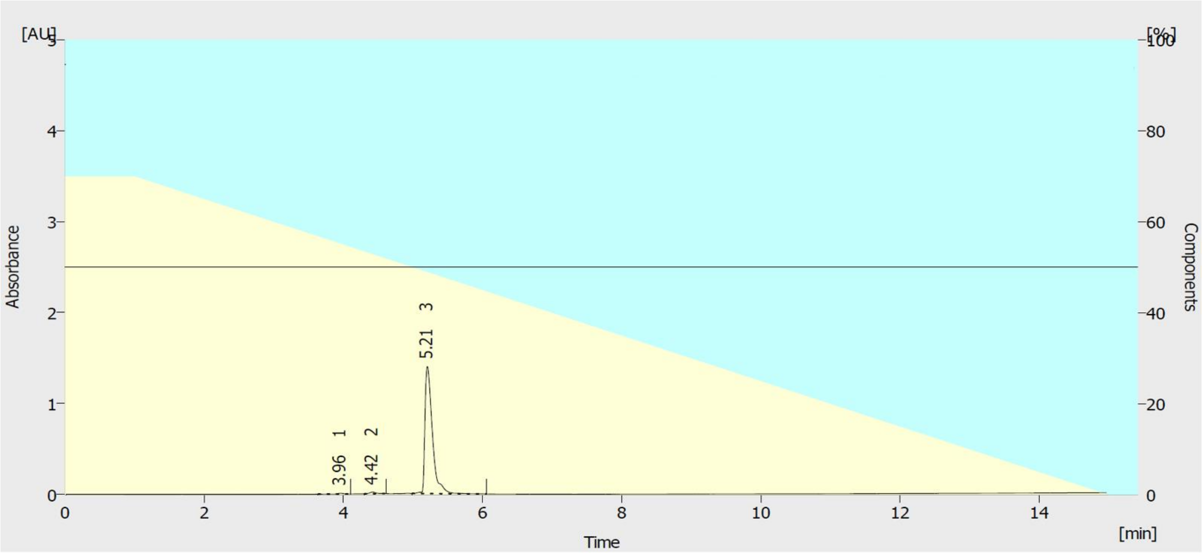

	Reten. Time [min]	Area [mAU.s]	Height [mAU]	Area [%]	Height [%]	W05 [min]	PDA Peak Purity	Compound Name
1	3.958	51.049	8.504	0.5	0.6	0.09	994	
2	4.420	113.761	18.739	1.1	1.3	0.09	989	
3	5.208	10391.958	1397.864	98.4	98.1	0.11	782	
	Total	10556.767	1425.107	100.0	100.0			

Figure S71. HPLC trace of compound **11.** t_R = 5.2 min, 98 % (B/A: 30/70 → 100/0 in 14 min, flow 1.2 ml/min, 254 nm).

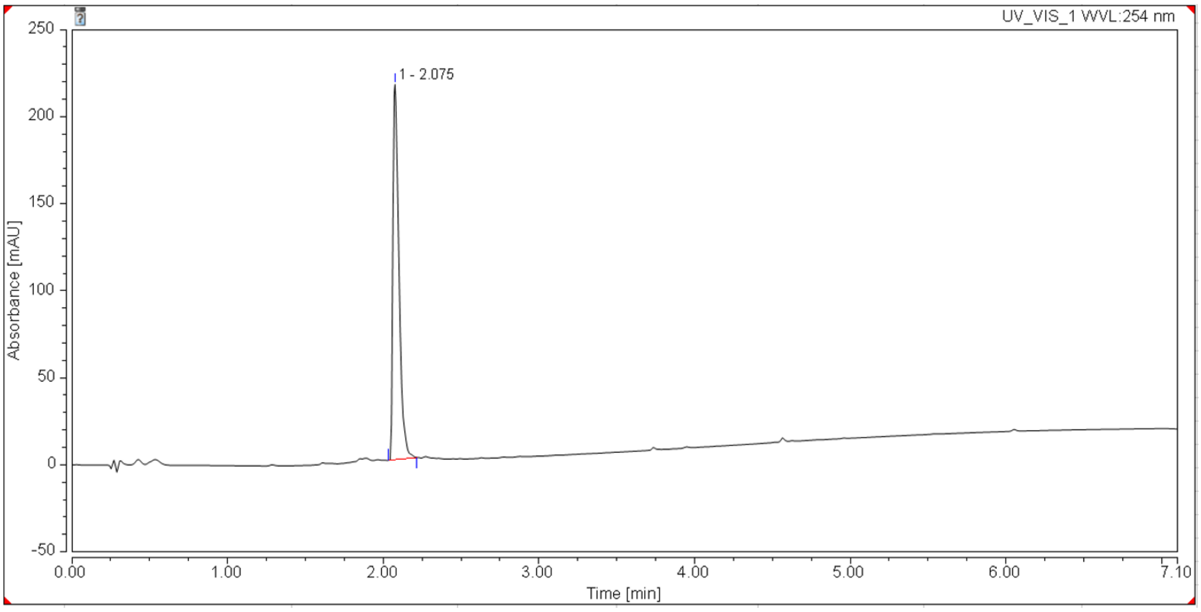

Figure S72. HPLC trace of compound **12.** Analytical column: Phenomenex Kinetex C18, C18-H, 1.7 µm, 50×3 mm; solvent A: H_2O + 0.1% v/v FA, solvent B: MeCN + 0.1% v/v FA; temperature 25 °C. t_R = 2.1 min, 100 % (B/A: 20/80 → 100/0 in 4 min, flow 0.8 ml/min, 254 nm).

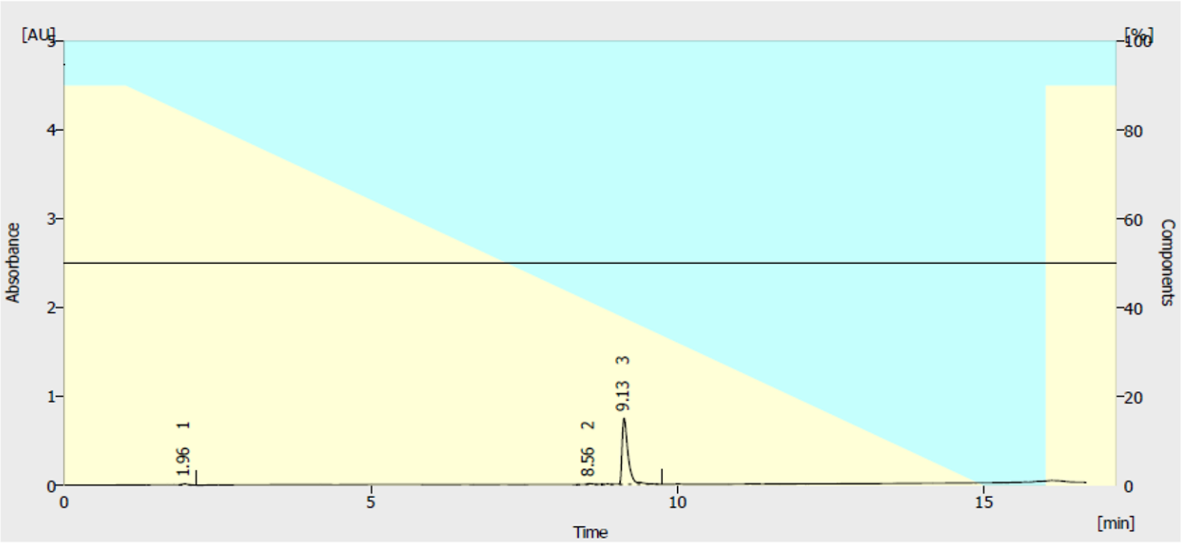

	Reten. Time [min]	Area [mAU.s]	Height [mAU]	Area [%]	Height [%]	W05 [min]	PDA Peak Purity	Compound Name
1	1.960	66.678	11.593	1.3	1.5	0.09	964	
2	8.558	110.470	11.835	2.1	1.5	0.11	999	
3	9.130	5091.934	745.250	96.6	97.0	0.10	849	
	Total	5269.082	768.678	100.0	100.0			

Figure S73. HPLC trace of compound **13.** t_R = 9.1 min, 97 % (B/A: 10/90 → 100/0 in 14 min, flow 1.2 ml/min, 254 nm).

NOTES

The TOC graphic contains the heart image generated by Gyuzel Mitronova using AI models (NightCafe).

ABBREVIATIONS

CA - caffeic acid

CHO - Chinese hamster ovary cells

DCM - Dichloromethane

DMEM - Dulbecco's modified eagle medium

ER - endoplasmic reticulum

HATU – *O*-(7-azabenzotriazol-1-yl)-*N,N,N',N'*-tetramethyluronium hexafluorphosphat

HBTU – 2-(1*H*-benzotriazol-1-yl)-1,1,3,3-tetramethyluronium hexafluorophosphate

HEK-293 – human embryonic kidney 293 cells

HF – heart failure

HL-1 – atrial muscle cells

NADH - nicotinamide adenine dinucleotide

$Na_V1.5$ – cardiac voltage-gated sodium channel

hRyR1 – human ryanodine receptor 1,

RMSD - root mean squared deviation

RyR2 – ryanodine receptor 2

SERCA2a – sarco/endoplasmic reticulum Ca^{2+}-ATPase 2a

SER - sarcoendoplasmic reticulum

TBDMS - tert-butyldimethylsilyl

WT - wild type

REFERENCE

1. Kabashima, Y.; Ogawa, H.; Nakajima, R.; Toyoshima, C., What ATP binding does to the Ca2+ pump and how nonproductive phosphoryl transfer is prevented in the absence of Ca2+. *PNAS* **2020,** *117* (31), 18448-18458.
